# An integrative analysis of genome-wide association study and regulatory SNP annotation datasets identified candidate genes for bipolar disorder

**DOI:** 10.1186/s40345-019-0170-z

**Published:** 2020-02-03

**Authors:** Xin Qi, Yan Wen, Ping Li, Chujun Liang, Bolun Cheng, Mei Ma, Shiqiang Cheng, Lu Zhang, Li Liu, Om Prakash Kafle, Feng Zhang

**Affiliations:** Key Laboratory of Trace Elements and Endemic Diseases of National Health and Family Planning Commission, School of Public Health, Health Science Center, Xi’an Jiaotong University, No. 76 Yan Ta West Road, Xi’an, 710061 People’s Republic of China

**Keywords:** Bipolar disorder, Regulatory SNP, Genome-wide association studies

## Abstract

**Background:**

Bipolar disorder (BD) is a complex mood disorder. The genetic mechanism of BD remains largely unknown.

**Methods:**

We conducted an integrative analysis of genome-wide association study (GWAS) and regulatory SNP (rSNP) annotation datasets, including transcription factor binding regions (TFBRs), chromatin interactive regions (CIRs), mature microRNA regions (miRNAs), long non-coding RNA regions (lncRNAs), topologically associated domains (TADs) and circular RNAs (circRNAs). Firstly, GWAS dataset 1 of BD (including 20,352 cases and 31,358 controls) and GWAS dataset 2 of BD (including 7481 BD patients and 9250 controls) were integrated with rSNP annotation database to obtain BD associated SNP regulatory elements and SNP regulatory element-target gene (E–G) pairs, respectively. Secondly, a comparative analysis of the two datasets results was conducted to identify the common rSNPs and also their target genes. Then, gene sets enrichment analysis (FUMA GWAS) and HumanNet-XC analysis were conducted to explore the functional relevance of identified target genes with BD.

**Results:**

After the integrative analysis, we identified 52 TFBRs target genes, 44 TADs target genes, 55 CIRs target genes and 21 lncRNAs target genes for BD, such as *ITIH4* (*P*_*dataset1*_ = 6.68 × 10^−8^, *P*_*dataset2*_ = 6.64 × 10^−7^), *ITIH3* (*P*_*dataset1*_ = 1.09 × 10^−8^, *P*_*dataset2*_ = 2.00 × 10^−7^), *SYNE1* (*P*_*dataset1*_ = 1.80 × 10^−6^, *P*_*dataset2*_ = 4.33 × 10^−9^) and *OPRM1* (*P*_*dataset1*_ = 1.80 × 10^−6^, *P*_*dataset2*_ = 4.33 × 10^−9^).

**Conclusion:**

We conducted a large-scale integrative analysis of GWAS and 6 common rSNP information datasets to explore the potential roles of rSNPs in the genetic mechanism of BD. We identified multiple candidate genes for BD, supporting the importance of rSNP in the development of BD.

## Background

Bipolar disorder (BD) is a common and often life-threatening mood disorder, which is characterized by recurrent manic, depressive or mixed states episodes (Judd et al. 2005). The prevalence of BD is more than 1%, and it is one of the important causes of disability among young adults (Merikangas et al. 2011; Alonso et al. 2010). BD can lead to cognitive and functional impairment and raised mortality, particularly death caused by suicide (Martinez-Aran et al. 2007). The suicide rate in BD patients is 20–30 times higher than in the general populations (Pompili et al. 2013). BD brings heavy burdens to BD patients and society, including direct costs of treatment and indirect costs (Dilsaver 2011).

BD is a complex disorder with strong genetic factors (Mühleisen et al. 2014). The estimated heritability of BD ranged from 60 to 80% (Craddock and Forty 2006). Multiple genome-wide association studies (GWAS) of BD has been conducted. For instance, a large-scale GWAS of 7481 individuals with BD and 9250 controls identified a new susceptibility variant in *ODZ4* gene (Psychiatric et al. 2011). *TRANK1*, *LMAN2L* and *PTGFR* were also identified by GWAS as the candidate genes for BD (Chen et al. 2011). Although GWAS has successfully identified multiple susceptibility genes associated with BD, there is still a challenge to clarify the roles of genomic regulatory elements in the development of BD. Recent studies observed that the significant SNPs detected by GWAS were enriched in non-coding regulatory genomic loci, for instance, expression quantitative traits (eQTLs) (Vernot et al. 2012). However, limited efforts have been paid to explore the roles of genomic regulatory loci in the genetic mechanism of BD.

Regulatory single nucleotide polymorphisms (rSNPs) are a group of regulatory genomic loci, which can produce new regulatory elements, such as transcription factor binding regions (TFBRs) and chromatin interactive regions (CIRs) (Wu et al. 2009). SNPs involved in transcription factor binding sites (TFBSs) or that affect TF-DNA binding affinity were considered to be predominant rSNPs (Riva 2012). It is similar to the effect of SNPs on protein structure and function, and the functional effect of rSNPs has been widely studied in recent years (Munkhtulga et al. 2010). Previous studies found that rSNPs played a vital role in the molecular mechanism of complex diseases (Marco et al. 2006). For example, Marco et al. (2006) identified a gain-of-function rSNP in a non-coding region of alpha thalassemia, which could interfere the normal activation of the downstream alpha-like globin genes through producing new promoterlike element. Integrating GWAS dataset and rSNP annotation information has the potential to provide novel clues for clarifying the genetic mechanism of human complex diseases (Macintyre et al. 2010). For instance, Macintyre et al. (2010) identified 11 rSNPs with disrupted impact on TF binding site from disease- and trait-associated GWAS SNPs. The functional relevance of the 9 of 11 rSNPs had been reported by previous studies (Macintyre et al. 2010). The rSNPs (rs4150275 and rs17655) of *ERCC5* gene were also found to be associated with chronic obstructive pulmonary disease by combing GWAS and regulatory SNPs annotation information (Yeo et al. 2018).

To identify novel BD associated genetic loci, we performed an integrative analysis of GWAS and rSNP annotation database for BD. GWAS dataset 1 of BD and GWAS dataset 2 of BD were integrated with rSNP annotation database to obtain BD associated SNP regulatory elements and SNP regulatory element-target gene (E–G) pairs, respectively. Then, a comparative analysis of the two datasets results was conducted to identify the common rSNPs and their target genes. Additionally, the identified BD associated genes were subjected to enrichment analysis and HumanNet-XC analysis to explore the functional relevance of the identified genes with BD.

## Materials and methods

### GWAS dataset 1 of BD

A recent large-scale GWAS summary dataset of BD was driven from the Psychiatric Genomics Consortium (PGC) (Stahl et al. 2019). The detailed information of the dataset and analysis procedure can be found in this published study (Stahl et al. 2019). Briefly, 20,352 cases and 31,358 controls of European descent were included in this GWA study. A standardized quality control, imputation and analysis were performed in this published study according to the PGC “ricopili” pipeline (Stahl et al. 2019; Ripke S. Ricopili: a tool for visualizing regions of interest in select GWAS data sets 2014). The default criteria for retaining genotyped SNPs and subjects includes: SNP missingness < 0.05 (before sample removal) and SNP missingness < 0.02 (after sample removal); subject SNP missingness < 0.02; autosomal heterozygosity deviation (|F_het_| < 0.2); difference in SNP missingness between cases and controls < 0.02; and SNP Hardy–Weinberg equilibrium (*P* > 10^−6^ in controls or *P* > 10^−10^ in cases). The software of IMPUTE2/SHAPEIT was used to perform genotype imputation (Howie et al. 2011; Delaneau et al. 2012). SNPs with imputation marker INFO score ≥ 0.6 and allele frequencies ≥ 0.01 and ≤ 0.99 were retained after imputation. Individual relatedness checks and principle component analysis (PCA) was also conducted in this study. After linkage disequilibrium (LD) pruning (r^2^ > 0.02) and frequency filtering (minor allele frequency (MAF) > 0.05), 24,498 autosomal SNPs were finally retained and used to calculate the IBS (identity by state) matrix of relatedness checks. Individuals related to another (with pi_hat > 0.2) were detected and one member of each pair was removed at random after preferentially retaining cases over controls.

### GWAS dataset 2 of BD

Another GWAS dataset of BD was driven from PGC (Psychiatric et al. 2011). The detailed information, including sample characteristics, experimental design and statistical analysis, was performed in the previous study (Psychiatric et al. 2011). In brief, this GWAS dataset contained 7481 subjects with BD and 9250 controls. SNP genotyping was performed using commercial platforms, such as Affymetrix 500 K, 5.0, 6.0 and Illumina HumanHap 500. Imputation was performed using BEAGLE 3.0 against the HapMap Phase 2 as Ref. (Browning and Browning 2007). After quality control, 2,415,422 autosomal SNPs with MAF ≥ 1% and imputation quality score r^2^ > 0.3 were analyzed using logistic regression.

### rSNP annotation dataset

The regulatory features of SNPs in human genome are annotated by the rSNPBase 3.1 database (http://rsnp3.psych.ac.cn/) (Guo and Wang 2018). rSNPBase 3.1 contains 119,630,196 rSNP annotation items, including human SNP-related regulatory elements as well as their target regulatory genes (Guo and Wang 2018). There are 6 common types of regulatory elements annotated by the rSNPBase 3.1, including transcription factor binding regions (TFBRs), chromatin interactive regions (CIRs), mature microRNA regions (miRNAs), long non-coding RNA regions (lncRNAs), topologically associated domains (TADs) and circular RNAs (circRNAs). They used genomic proximity to detect the relationship between the included regulatory elements and genes from Ensembl (GRCh37) (Aken et al. 2016).

### Integrative analysis of GWAS and rSNP annotation information

**Fig. 1:**
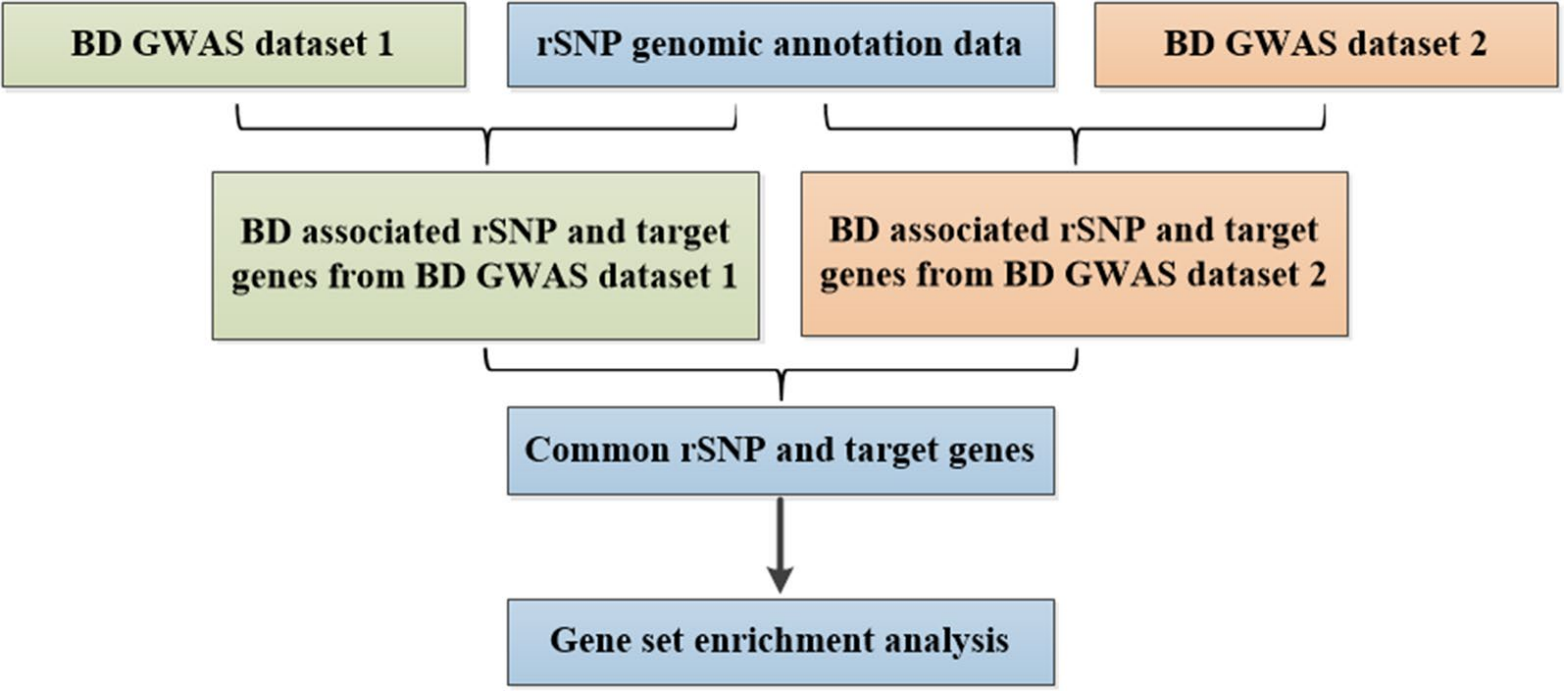
Flowchart

To integrate the GWAS dataset of BD with the rSNP functional annotation information, the SNPs with GWAS *P* value < 10^−5^ were selected from GWAS dataset 1 and GWAS dataset 2, respectively. The selected SNPs were then annotated by rSNPBase 3.1 to obtain BD associated SNP regulatory elements as well as their target genes (including TFBRs, CIRs, miRNAs, lncRNAs and TADs). Then, a comparative analysis of the two datasets results was conducted to identify the common rSNPs and their target genes (except for circRNAs). A flowchart for this study was showed in Fig. 1.

### Functional analysis of identified target genes

The gene sets enrichment analysis and HumanNet-XC analysis were conducted to explore the functional relevance of the target genes of identified rSNPs with BD. The gene sets enrichment analysis was implemented by FUMA GWAS (Functional Mapping and Annotation of Genome-Wide Association Studies, https://fuma.ctglab.nl/) (Watanabe et al. 2017). HumanNet-XC (Functional gene network extended by Co-citation) (http://www.inetbio.org/humannet/) were found to show the best performance in ranking disease-linked gene sets with minimal literature-dependent biases (Hwang et al. 2018). The gene-set analysis of HumanNet-XC were applied to the target genes of identified rSNP for exploring the disease-association with annotated disease genes from DisGeNET and DISEASES (Piñero et al. 2017; Pletscher-Frankild et al. 2015). The candidate genes for BD were selected according to their closeness to the guide genes, and the putative disease gene network was explored with an interactive network viewer (Hwang et al. 2018).

## Results

### BD associated rSNP and their target genes

GWAS dataset 1 study identified a group of rSNPs for BD, including 218 rSNP for TFBRs, 1200 rSNP for TADs, 624 rSNP for CIRs and 130 rSNP for lncRNAs, corresponding to 507, 362, 591 and 142 target regulatory genes, respectively (Additional file 1: Table S1). 1751 of these rSNPs had been demonstrated as eQTLs in the previous literature. For circRNA region, 2354 rSNPs were identified for BD. In GWAS dataset 2 study, we detected 24 rSNP for TFBRs, 192 rSNP for TADs, 54 rSNP for CIRs and 15 rSNPs for lncRNAs, corresponding to 77, 56, 71 and 21 target regulatory genes, respectively. Among these rSNPs, 200 rSNPs had been identified as eQTLs in published studies (Additional file 2: Table S2).

After comparing GWAS dataset 1 and GWAS dataset 2 study results, we identified 52 common target genes for TFBRs, 44 common target genes for TADs, 55 common target genes for CIRs and 21 common target genes for lncRNAs, respectively (Additional file 3: Table S3). Irrespective of different regulatory elements, 85 target regulatory genes of the identified rSNPs were found for BD, such as *ITIH4* (*P*_*dataset1*_ = 6.68 × 10^−8^, *P*_*dataset2*_ = 6.64 × 10^−7^), *ITIH3* (*P*_*dataset1*_ = 1.09 × 10^−8^, *P*_*dataset2*_ = 2.00 × 10^−7^), *SYNE1* (*P*_*dataset1*_ = 1.80 × 10^−6^, *P*_*dataset2*_ = 4.33 × 10^−9^), *OPRM1* (*P*_*dataset1*_ = 1.80 × 10^−6^, *P*_*dataset2*_ = 4.33 × 10^−9^) and *HDAC2* (*P*_*dataset1*_ = 6.58 × 10^−6^, *P*_*dataset2*_ = 3.35 × 10^−8^) (Additional file 3: Table S3). The top 20 genes were listed in Table 1.

**Table 1:** List of the top 20 common target genes shared by of BD GWAS dataset 1 and dataset 2

Gene	SNP-associated regulatory elements	BD GWAS dataset 1		BD GWAS dataset 2	
		SNP	P_dataset1_	SNP	P_dataset2_
IPCEF1	TADs	rs9371601	1.80 × 10^−6^	rs9371601	4.33 × 10^−9^
OPRM1	TADs	rs9371601	1.80 × 10^−6^	rs9371601	4.33 × 10^−9^
SYNE1	TADs	rs9371601	1.80 × 10^−6^	rs9371601	4.33 × 10^−9^
RGS17	TADs	rs215005	6.04 × 10^−6^	rs214952	3.19 × 10^−8^
TFB1M	TADs	rs215005	6.04 × 10^−6^	rs214952	3.19 × 10^−8^
CCDC170	TADs	rs9371601	1.80 × 10^−6^	rs551900	3.35 × 10^−8^
ENSG00000235652	TADs	rs548985	6.58 × 10^−6^	rs551900	3.35 × 10^−8^
HDAC2	TADs	rs548985	6.58 × 10^−6^	rs551900	3.35 × 10^−8^
KMT2D	CIRs	rs10783301	3.32 × 10^−6^	rs10875914	8.27 × 10^−8^
ENSG00000258101	CIRs, TFBRs	rs10459221	4.20 × 10^−7^	rs10459232	8.81 × 10^−8^
RHEBL1	CIRs, TFBRs	rs7969091	3.25 × 10^−7^	rs7969091	8.91 × 10^−8^
ENSG00000257346	CIRs, lncRNAs	rs11168839	2.89 × 10^−6^	rs11168839	1.24 × 10^−7^
PRKAG1	CIRs, TFBRs	rs2304275	1.45 × 10^−6^	rs10875915	1.82 × 10^−7^
ENSG00000257913	CIRs, TFBRs	rs2293445	6.51 × 10^−6^	rs2293445	1.96 × 10^−7^
VPRBP	TADs, CIRs, TFBRs	rs2302417	4.93 × 10^−9^	rs736408	2.00 × 10^−7^
ITIH3	TADs, CIRs, TFBRs	rs4481150	1.09 × 10^−8^	rs736408	2.00 × 10^−7^
ENSG00000272822	CIRs, TFBRs	rs1054442	3.27 × 10^−6^	rs10783299	2.53 × 10^−7^
RNU6-940P	CIRs, TFBRs	rs1054442	3.27 × 10^−6^	rs10783299	2.53 × 10^−7^
WNT1	CIRs, TFBRs	rs1054442	3.27 × 10^−6^	rs10783299	2.53 × 10^−7^
LMAN2L	TFBRs	rs72809838	6.47 × 10^−9^	rs2271893	4.59 × 10^−7^

*TFBRs* transcription factor binding regions, *CIRs* chromatin interactive regions, long *lncRNAs* non-coding RNAs regions, *TADs* topologically associated domains

### Gene sets enrichment analysis

Additional file 4: Table S4 summarized the gene sets enrichment analysis results of 85 common target genes detected in the two datasets studies. We detected 5 significant immunologic signatures signals, such as GSE19401_PLN_VS_PEYERS_PATCH_FOLLICULAR_DC_UP (*P* value = 2.64 × 10^−6^, adjusted *P* = 1.29 × 10^−2^), GSE29618_BCELL_VS_PDC_DN (*P* value = 4.74 × 10^−5^, adjusted *P* = 4.96 × 10^−2^) and GSE43955_TH0_VS_TGFB_IL6_TH17_ACT_CD4_TCELL_10H_UP (*P* value = 4.97 × 10^−5^, adjusted *P* = 4.96 × 10^−2^).

### HumanNet-XC disease gene prediction

HumanNet-XC tool was used to further explore the diseases closely related to the identified 85 target regulatory genes (Additional file 5: Figure S1). Among the 85 target regulatory genes, 53 genes were identified as guide genes and 2285 candidate genes within group connectivity of guide genes were found by HumanNet-XC (Additional file 6: Table S5, Additional file 7: Table S6). The results of annotated disease genes from DisGeNET and DISEASES were showed in Additional file 8: Table S7. Intriguingly, BD were found to be the most significant in the annotation genes from DisGeNET (*P* value = 7.41 × 10^−5^).

## Discussion

To explore the functional relevance of rSNP and their target genes in the pathogenesis of BD, we conducted an integrative analysis of GWAS and rSNPs annotation information. We identified a group of rSNP and their target genes for BD. Further functional analysis of the identified target genes support the importance of rSNPs in the genetic mechanism of BD.

Integrating BD GWAS and rSNP datasets observed association evidence between BD and the TADs and CIRs rSNPs of ITIH3, as well as the TFBRs and CIRs rSNP of ITIH4. ITIH4 has been confirmed to be a biomarker of neuroinflammation and neuroinflammation was involved in the development of BD (Yang et al. 2012; Najjar et al. 2013). Association analysis showed that rs2239547 (ITIH3/4-region) was significantly related with a history of suicide attempt in BD patients (Finseth et al. 2014). Besides, in a joint analysis of BD and schizophrenia, the ITIH3-ITIH4 region (rs2239547) reached genome-wide significance in the support for shared susceptibility (Ripke et al. 2011).

SYNE1 encodes spectrin repeat containing nuclear envelope protein 1, which is a part of a complex linking nucleoskeleton to cytoskeleton (Warren et al. 2005). The significant association between SYNE1 and BD have been observed in an samples of 1527 BD cases and 1579 controls, and a combined analysis of PGC-BD data (*P* = 2.9 × 10^−8^, OR = 1.104) (Green et al. 2013). Moreover, polymorphisms in SYNE1 conferred a greater risk of developing BD in the high genetic risk individuals (Gassó et al. 2016). Our study results suggested that the rSNP of SYNE1 may contributed to BD through TADs and TFBRs.

OPRM1 is a notable gene identified by this study. It has been demonstrated that OPRM1 was related to substance dependence and drug dependence (Glatt et al. 2007; Luo et al. 2003). The previous study showed that substance dependence had a close relationship with BD patients (Leventhal and Zimmerman 2010). Besides, a case–control study suggested that BD was significantly associated with a risk for substance use disorder and dependence, independent of psychiatric comorbidity (Wilens et al. 2008).

HDAC2 (histone deacetylase 2) is also identified for BD in our analysis. The inhibition of histone deacetylase had the effect on neuroprotective and neuroregenerative properties in animal models of brain diseases (Fischer et al. 2010). And the inhibition of histone deacetylase may supply a potential target in the treatment of BD (Machado-Vieira et al. 2011). In addition, it was found that HDAC2 were identified to be a risk gene for BD, and be involved in regulating early brain development (Xiang et al. 2017).

There are several issues need to be noted. First, the common 6 types of rSNP annotation information were collected from the rSNPBase 3.1. Some new regulatory elements are not included in the rSNPBase 3.1 database, for instance m6A (Harper et al. 1990). Second, a small part of subjects were overlapping between the two GWAS datasets of BD, which may affect the robust of our study results. Third, the functional annotation information of rSNP did not contain the biological effect of target genes in different tissue or cell types. Therefore, our rSNP analysis could not take tissue or cell types into account. Further studies are warranted to explore the roles of identified genes within different tissue and cell types. Forth, this study focused on the rSNP, which occupied a small part of whole genome. We think that using the genome-wide significance threshold (for example P < 5.0 × 10^−8^) is too strict and may miss bipolar disorder associated rSNPs. Therefore, we used the threshold of P < 1.0 × 10^−5^ in this study.

In conclusion, we conducted a large-scale integrative analysis of GWAS and 6 common rSNP information to explore the potential roles of rSNPs in the genetic mechanism of BD. We identified multiple rSNPs and candidate genes for BD. We hope that our study results are helpful for understanding the genetic mechanism of BD.

## Supplementary information

**Additional file 1: Table S1.** rSNP functional annotation analysis results of BD GWAS dataset 1.

**Additional file 2: Table S2.** rSNP functional annotation analysis results of BD GWAS dataset 2.

**Additional file 3: Table S3.** List of the common target genes shared by BD GWAS dataset 1 and dataset 2.

**Additional file 4: Table S4.** FUMA gene set enrichment analysis results.

**Additional file 5: Figure S1.** Based on the 85 common target genes as guide genes, we used HumanNet-XC to predict network-based disease gene. The upper panel shows the interactive network viewer, and a group of guide genes (green nodes), which can be annotated by their neighbors as putative candidate genes (blue nodes). The local subnetwork of the first ranked candidate, ITIH4, and its neighbors were highlighted. DISEASES and DisGeNET, serving to validate the specific prediction result, already annotate the retrieved gene ITIH4 for Bipolar Disorder. The lower panel shows the guide genes, including the statistical significance of within group connectivity of guide genes, and the observed network performance for guide gene recovery reported as receiver operating characteristic curve (ROC) curves. The area under the receiver operating characteristic curve (AUROC) indicated the predictive HumanNet-XC networks performance for a disease, which was based on the efficiency of guide gene recovery.

**Additional file 6: Table S5.** List of the candidate genes identified by HumanNet-XC analysis.

**Additional file 7: Table S6.** List of the guide genes identified by HumanNet-XC analysis

**Additional file 8: Table S7.** HumanNet-XC gene-set analysis results.

## Data Availability

Not applicable.
