# Supplementary material for: An integrative analysis of genome-wide association study and regulatory SNP annotation datasets identified candidate genes for bipolar disorder
Source: Int J Bipolar Disord. 2020 Feb 3;8:6. doi: 10.1186/s40345-019-0170-z (PMC6995798; doi:10.1186/s40345-019-0170-z)
Supplement: Supplementary file 1 — Additional file 1: Table S1. rSNP functional annotation analysis results of BD GWAS dataset 1. [file 40345_2019_170_MOESM1_ESM.docx]

Table S1. rSNP functional annotation analysis results of BD GWAS dataset 1

| **SNP** | **SNP-related regulatory elements** | **element-gene (E-G) pairs** | **eQTL^*^** | **P** |
| --- | --- | --- | --- | --- |
| rs9834970 | TADs | MOBP,CTDSPL,ENTPD3-AS1 | Y | 5.53E-14 |
| rs11129735 | circRNAs,TADs | MOBP,CTDSPL,ENTPD3-AS1 | N | 1.16E-11 |
| rs4072458 | TADs | MOBP,CTDSPL,ENTPD3-AS1 | N | 2.36E-11 |
| rs1553656 | TADs | MOBP,CTDSPL,ENTPD3-AS1 | N | 3.5E-11 |
| rs4789 | circRNAs,TADs | MOBP,CTDSPL,ENTPD3-AS1 | Y | 3.74E-11 |
| rs55644704 | TADs | DLEC1 | N | 8.87E-11 |
| rs111444407 | circRNAs |  | N | 2.4E-10 |
| rs3732386 | circRNAs,TADs | MOBP,CTDSPL,ENTPD3-AS1 | N | 3.32E-10 |
| rs3732385 | circRNAs,TADs | MOBP,CTDSPL,ENTPD3-AS1 | N | 3.34E-10 |
| rs7652637 | circRNAs,TADs | MOBP,CTDSPL,ENTPD3-AS1 | N | 3.34E-10 |
| rs3732384 | circRNAs,TADs | MOBP,CTDSPL,ENTPD3-AS1 | N | 3.34E-10 |
| rs4234258 | circRNAs,TADs | MOBP,CTDSPL,ENTPD3-AS1 | Y | 3.75E-10 |
| rs9811916 | circRNAs,TADs | MOBP,CTDSPL,ENTPD3-AS1 | Y | 4.44E-10 |
| rs4678909 | circRNAs,TADs | MOBP,CTDSPL,ENTPD3-AS1 | N | 4.57E-10 |
| rs112219496 | circRNAs |  | N | 5.85E-10 |
| rs1532965 | TADs | MOBP,CTDSPL,ENTPD3-AS1 | N | 6.25E-10 |
| rs12637912 | circRNAs,TADs | MOBP,CTDSPL,ENTPD3-AS1 | Y | 6.31E-10 |
| rs4624519 | TADs | MOBP,CTDSPL,ENTPD3-AS1 | Y | 7.93E-10 |
| rs8108777 | circRNAs |  | Y | 8.38E-10 |
| rs4808932 | circRNAs |  | Y | 8.62E-10 |
| rs10423874 | circRNAs |  | Y | 1.03E-09 |
| rs2011503 | circRNAs,CIRs | SUGP1,MAU2 | Y | 1.07E-09 |
| rs7628483 | CIRs | MLH1 | Y | 1.1E-09 |
| rs13314421 | CIRs | MLH1 | Y | 1.14E-09 |
| rs9863798 | circRNAs,TADs | TRANK1,GOLGA4 | N | 1.14E-09 |
| rs4239639 | circRNAs |  | Y | 1.31E-09 |
| rs7624498 | TADs | DLEC1,CCDC66,TRANK1 | Y | 1.34E-09 |
| rs1557723 | circRNAs,CIRs | SUGP1,MAU2 | Y | 1.41E-09 |
| rs756999 | circRNAs |  | Y | 1.51E-09 |
| rs2965190 | circRNAs |  | Y | 1.59E-09 |
| rs1989867 | circRNAs |  | Y | 1.65E-09 |
| rs9882911 | circRNAs,TADs | TRANK1,GOLGA4 | Y | 1.79E-09 |
| rs111492783 | circRNAs |  | N | 1.81E-09 |
| rs17807744 | circRNAs,TADs | TRANK1,GOLGA4 | Y | 2E-09 |
| rs4678912 | circRNAs,TADs | TRANK1,GOLGA4 | N | 2.08E-09 |
| rs2905422 | circRNAs |  | Y | 2.09E-09 |
| rs9883001 | circRNAs,TADs | TRANK1,GOLGA4 | N | 2.09E-09 |
| rs17195098 | circRNAs,TADs | TRANK1,GOLGA4 | N | 2.1E-09 |
| rs756997 | lncRNAs |  | Y | 2.18E-09 |
| rs9821223 | circRNAs,TADs | TRANK1,GOLGA4 | N | 2.19E-09 |
| rs4678911 | circRNAs,TADs | TRANK1,GOLGA4 | N | 2.24E-09 |
| rs9819304 | circRNAs,TADs | TRANK1,GOLGA4 | N | 2.25E-09 |
| rs6771655 | circRNAs,TADs | TRANK1,GOLGA4 | N | 2.28E-09 |
| rs2965195 | circRNAs |  | Y | 2.28E-09 |
| rs4678910 | circRNAs,TADs | TRANK1,GOLGA4 | N | 2.29E-09 |
| rs6550435 | TADs | MOBP,CTDSPL,ENTPD3-AS1 | N | 2.31E-09 |
| rs4678913 | circRNAs,TADs | TRANK1,GOLGA4 | N | 2.88E-09 |
| rs7257875 | CIRs | MAU2 | Y | 2.9E-09 |
| rs10422893 | CIRs | MAU2 | Y | 2.9E-09 |
| rs10744560 | circRNAs |  | Y | 2.92E-09 |
| rs138739237 | TADs | TRANK1,GOLGA4 | N | 2.98E-09 |
| rs56044734 | lncRNAs |  | Y | 3.06E-09 |
| rs55762233 | lncRNAs |  | Y | 3.2E-09 |
| rs10403273 | TFBRs | HAPLN4 | Y | 3.39E-09 |
| rs4298967 | circRNAs |  | Y | 3.54E-09 |
| rs8113794 | circRNAs |  | Y | 3.91E-09 |
| rs4808934 | CIRs | MAU2 | Y | 3.95E-09 |
| rs56149992 | circRNAs,TADs | DLEC1,CCDC66,TRANK1 | N | 4.06E-09 |
| rs17216041 | lncRNAs |  | Y | 4.2E-09 |
| rs12311439 | circRNAs |  | N | 4.3E-09 |
| rs8100097 | circRNAs |  | Y | 4.48E-09 |
| rs80146298 | circRNAs,CIRs | SUGP1,ENSG00000267629,TM6SF2 | N | 4.56E-09 |
| rs138321 | TADs,TFBRs | MIR4766,TNRC6B | Y | 4.69E-09 |
| rs56135441 | CIRs | MAU2 | Y | 4.7E-09 |
| rs56352336 | circRNAs |  | Y | 4.7E-09 |
| rs2965194 | circRNAs |  | Y | 4.81E-09 |
| rs76944848 | CIRs | MAU2 | N | 4.82E-09 |
| rs10774036 | circRNAs |  | N | 4.92E-09 |
| rs2302417 | TADs | VPRBP | Y | 4.93E-09 |
| rs56278919 | lncRNAs |  | Y | 4.94E-09 |
| rs17751061 | circRNAs |  | N | 5.14E-09 |
| rs56212061 | circRNAs |  | Y | 5.45E-09 |
| rs72999016 | circRNAs |  | N | 5.46E-09 |
| rs148264883 | circRNAs,TADs | DLEC1,CCDC66,TRANK1 | N | 5.56E-09 |
| rs884301 | circRNAs |  | N | 5.8E-09 |
| rs884303 | circRNAs |  | N | 5.87E-09 |
| rs4239193 | circRNAs |  | N | 6.25E-09 |
| rs75968099 | TADs | MOBP,CTDSPL,ENTPD3-AS1 | N | 6.44E-09 |
| rs72809838 | CIRs | LMAN2L | N | 6.47E-09 |
| rs4765913 | circRNAs |  | N | 6.57E-09 |
| rs55690320 | circRNAs,TADs,CIRs | DLEC1,CCDC66,MLH1,TRANK1,EPM2AIP1 | N | 6.75E-09 |
| rs2159100 | circRNAs |  | Y | 6.85E-09 |
| rs73068044 | circRNAs,TADs | TRANK1,GOLGA4 | N | 7.12E-09 |
| rs8100713 | circRNAs |  | Y | 7.34E-09 |
| rs12315711 | circRNAs |  | N | 7.5E-09 |
| rs4765905 | circRNAs |  | Y | 7.52E-09 |
| rs113145184 | CIRs | MAU2 | N | 7.82E-09 |
| rs57436187 | CIRs,TFBRs | MAU2,RFXANK | Y | 7.97E-09 |
| rs1006737 | circRNAs |  | Y | 8.14E-09 |
| rs876720 | circRNAs |  | N | 8.33E-09 |
| rs769087 | circRNAs |  | N | 8.83E-09 |
| rs12972691 | circRNAs |  | Y | 8.85E-09 |
| rs7646741 | circRNAs,TADs | NEK4 | Y | 9.52E-09 |
| rs2710323 | TADs | VPRBP | Y | 9.85E-09 |
| rs73496688 | TADs | TENM4,JRKL,JRKL-AS1,DLG2 | N | 1.05E-08 |
| rs56361249 | circRNAs |  | N | 1.07E-08 |
| rs55822665 | CIRs,TFBRs | MEF2B,ENSG00000267629,MEF2BNB,TM6SF2 | Y | 1.09E-08 |
| rs4481150 | circRNAs,TADs,CIRs | VPRBP,ITIH3 | Y | 1.09E-08 |
| rs56351161 | circRNAs |  | N | 1.12E-08 |
| rs9867455 | TADs | DLEC1,CCDC66,TRANK1 | Y | 1.13E-08 |
| rs112253053 | circRNAs,CIRs | SUGP1,ENSG00000267629,TM6SF2 | N | 1.15E-08 |
| rs12226877 | CIRs | FEN1 | Y | 1.17E-08 |
| rs11237821 | circRNAs |  | N | 1.17E-08 |
| rs12275195 | circRNAs |  | N | 1.18E-08 |
| rs758170 | circRNAs |  | N | 1.18E-08 |
| rs4808944 | circRNAs,CIRs | SUGP1,ENSG00000267629,TM6SF2 | Y | 1.18E-08 |
| rs10774035 | circRNAs |  | Y | 1.21E-08 |
| rs4515997 | circRNAs |  | N | 1.22E-08 |
| rs2315023 | circRNAs |  | Y | 1.22E-08 |
| rs72809828 | circRNAs,CIRs | LMAN2L | N | 1.22E-08 |
| rs2535627 | lncRNAs |  | Y | 1.3E-08 |
| rs67570263 | circRNAs |  | N | 1.3E-08 |
| rs72999059 | circRNAs |  | N | 1.31E-08 |
| rs9876421 | TADs | MOBP,CTDSPL,ENTPD3-AS1 | N | 1.52E-08 |
| rs329319 | circRNAs |  | Y | 1.54E-08 |
| rs6779665 | TADs,CIRs | DLEC1,CCDC66,MLH1,TRANK1 | Y | 1.57E-08 |
| rs7108878 | circRNAs |  | N | 1.58E-08 |
| rs11555053 | circRNAs |  | Y | 1.63E-08 |
| rs11130324 | circRNAs,TADs | NEK4 | Y | 1.68E-08 |
| rs2010506 | circRNAs |  | Y | 1.69E-08 |
| rs72999053 | circRNAs |  | N | 1.73E-08 |
| rs1024582 | circRNAs |  | Y | 1.74E-08 |
| rs11062170 | circRNAs |  | N | 1.76E-08 |
| rs4794563 | circRNAs |  | Y | 1.77E-08 |
| rs72809820 | CIRs | CNNM4 | N | 1.82E-08 |
| rs8110171 | circRNAs |  | Y | 1.84E-08 |
| rs3617 | circRNAs,TADs | VPRBP | Y | 1.84E-08 |
| rs11706780 | circRNAs,TADs | TRANK1,GOLGA4 | N | 1.9E-08 |
| rs7965923 | circRNAs |  | N | 1.92E-08 |
| rs71395455 | circRNAs,CIRs | EGLN1P1 | Y | 1.93E-08 |
| rs72809827 | circRNAs |  | N | 1.98E-08 |
| rs58361269 | circRNAs |  | N | 1.99E-08 |
| rs2271893 | circRNAs,CIRs,TFBRs | ANKRD36,,CNNM4,LMAN2L,APPBP2 | Y | 2.08E-08 |
| rs7612511 | circRNAs,TADs | PBRM1 | Y | 2.27E-08 |
| rs1511237 | circRNAs |  | N | 2.3E-08 |
| rs77427798 | TADs,CIRs | ZNF208,ZNF93,ENSG00000267268,ZNF431,ENSG00000267383,ENSG00000141979 | N | 2.31E-08 |
| rs4433593 | circRNAs |  | N | 2.32E-08 |
| rs12913702 | circRNAs |  | Y | 2.42E-08 |
| rs11237820 | circRNAs |  | N | 2.47E-08 |
| rs6769400 | TADs | DLEC1,CCDC66,TRANK1 | Y | 2.61E-08 |
| rs931913 | TADs | MOBP,CTDSPL,ENTPD3-AS1 | Y | 2.66E-08 |
| rs17150022 | circRNAs,TADs | DFNA5,CCDC126 | N | 2.7E-08 |
| rs77749926 | circRNAs |  | N | 2.7E-08 |
| rs79406027 | circRNAs |  | N | 2.71E-08 |
| rs12277834 | circRNAs |  | N | 2.71E-08 |
| rs7287625 | TADs | TNRC6B | Y | 2.71E-08 |
| rs7291189 | TADs | TNRC6B | Y | 2.74E-08 |
| rs968369 | TADs,TFBRs | TENM4,JRKL,JRKL-AS1,DLG2 | N | 2.74E-08 |
| rs73068054 | circRNAs,TADs | DLEC1,CCDC66,TRANK1 | N | 2.77E-08 |
| rs4808958 | circRNAs |  | Y | 2.81E-08 |
| rs12672003 | circRNAs,TADs | MPP6 | N | 2.87E-08 |
| rs77616520 | TADs,CIRs | ZNF208,ZNF93,ATP13A1,ENSG00000267268,ZNF431,ENSG00000267383,ENSG00000141979,TSSK6 | N | 3.01E-08 |
| rs35595120 | circRNAs,TADs,CIRs | DLEC1,CCDC66,MLH1,TRANK1,EPM2AIP1 | N | 3.01E-08 |
| rs12629699 | circRNAs,TADs | PBRM1 | Y | 3.04E-08 |
| rs4678552 | TADs | MOBP,CTDSPL,ENTPD3-AS1 | N | 3.04E-08 |
| rs80012185 | circRNAs,TADs | DFNA5,CCDC126 | N | 3.05E-08 |
| rs9882879 | TADs | MOBP,CTDSPL,ENTPD3-AS1 | N | 3.08E-08 |
| rs76790018 | TADs,CIRs | ZNF208,ZNF93,ATP13A1,ENSG00000267268,LPAR2,ZNF431,ENSG00000267383,ENSG00000141979,TSSK6 | N | 3.08E-08 |
| rs28576724 | TADs | DLEC1,CCDC66,TRANK1 | Y | 3.11E-08 |
| rs45556231 | TADs | ZNF208,ZNF431,ENSG00000267383,ENSG00000141979 | Y | 3.19E-08 |
| rs80195870 | circRNAs,TADs | DFNA5,CCDC126 | N | 3.29E-08 |
| rs13081155 | circRNAs |  | Y | 3.29E-08 |
| rs10433615 | circRNAs,TADs | PBRM1 | Y | 3.32E-08 |
| rs13231398 | TADs | IMMP2L | N | 3.36E-08 |
| rs138312 | circRNAs,TADs | TNRC6B | Y | 3.41E-08 |
| rs7395229 | CIRs | FEN1 | Y | 3.45E-08 |
| rs74654392 | circRNAs |  | N | 3.46E-08 |
| rs34067609 | TADs | ZNF208,ZNF431,ENSG00000267383,ENSG00000141979 | Y | 3.46E-08 |
| rs6976330 | circRNAs,TADs | DFNA5,CCDC126 | N | 3.51E-08 |
| rs2390982 | circRNAs,TADs | MPP6 | Y | 3.52E-08 |
| rs11509139 | circRNAs,TADs | DFNA5,CCDC126 | Y | 3.64E-08 |
| rs7256028 | circRNAs,CIRs,TFBRs | TSSK6 | Y | 3.65E-08 |
| rs113980591 | circRNAs |  | N | 3.65E-08 |
| rs174592 | circRNAs |  | Y | 3.66E-08 |
| rs16996127 | circRNAs,CIRs,TFBRs | POU6F1,SLC25A20,ATP13A1,NDUFA13,YJEFN3,PSME3,ENSG00000258674,PPP1CB,TSSK6 | Y | 3.67E-08 |
| rs1036215 | TADs | ZNF208,ZNF431,ENSG00000267383,ENSG00000141979 | Y | 3.68E-08 |
| rs3774349 | circRNAs,CIRs,TFBRs | ENSG00000252787,SNORD19 | Y | 3.91E-08 |
| rs75861367 | circRNAs,TADs | DFNA5,CCDC126 | N | 3.97E-08 |
| rs1108842 | CIRs,TFBRs,lncRNAs | EID1,ENSG00000272173,RNU7-1,CCDC88A,MGEA5,PBRM1,SPCS1,KCNIP2-AS1,TM9SF3,ENSG00000235883 | Y | 4.01E-08 |
| rs114829110 | circRNAs,TADs | DFNA5,CCDC126 | N | 4.03E-08 |
| rs146197383 | circRNAs,TADs | DFNA5,CCDC126 | N | 4.04E-08 |
| rs55927782 | TADs,CIRs | ZNF208,GMIP,ZNF431,ENSG00000267383,ENSG00000141979 | Y | 4.06E-08 |
| rs76473585 | circRNAs |  | N | 4.14E-08 |
| rs8111093 | circRNAs |  | Y | 4.32E-08 |
| rs6772646 | TADs | DLEC1,CCDC66,TRANK1 | Y | 4.33E-08 |
| rs6511027 | circRNAs |  | Y | 4.35E-08 |
| rs2245647 | circRNAs |  | Y | 4.39E-08 |
| rs10412176 | circRNAs |  | Y | 4.5E-08 |
| rs11668203 | circRNAs |  | Y | 4.54E-08 |
| rs10455979 | circRNAs |  | N | 4.6E-08 |
| rs3779463 | circRNAs,TADs | DFNA5,CCDC126 | Y | 4.6E-08 |
| rs114142225 | circRNAs,TADs | DFNA5,CCDC126 | N | 4.6E-08 |
| rs77112403 | circRNAs,TADs | DFNA5,CCDC126 | N | 4.61E-08 |
| rs960145 | circRNAs |  | N | 4.63E-08 |
| rs10486428 | circRNAs |  | Y | 4.7E-08 |
| rs111383872 | circRNAs,TADs | DFNA5,CCDC126 | N | 4.71E-08 |
| rs751857 | circRNAs,CIRs | MAU2 | Y | 4.74E-08 |
| rs79360559 | TFBRs | ENSG00000249568 | N | 4.76E-08 |
| rs2965184 | circRNAs,TFBRs | MIR640 | Y | 4.76E-08 |
| rs4678914 | circRNAs,TADs,CIRs | DLEC1,CCDC66,ENSG00000272127,TRANK1 | N | 4.81E-08 |
| rs1944449 | circRNAs |  | Y | 4.91E-08 |
| rs75575419 | circRNAs,TADs | DFNA5,CCDC126 | N | 4.91E-08 |
| rs138731899 | circRNAs,TADs | DFNA5,CCDC126 | N | 4.95E-08 |
| rs6949503 | circRNAs,TADs | DFNA5,CCDC126 | N | 4.99E-08 |
| rs77165503 | circRNAs,TADs | TENM4,JRKL,JRKL-AS1,DLG2 | N | 5.11E-08 |
| rs2577831 | circRNAs,TADs | PBRM1 | Y | 5.13E-08 |
| rs80200208 | circRNAs,CIRs | MIR640 | N | 5.14E-08 |
| rs8105642 | circRNAs |  | Y | 5.15E-08 |
| rs2060274 | circRNAs |  | Y | 5.15E-08 |
| rs8110250 | circRNAs |  | Y | 5.16E-08 |
| rs8101219 | TADs,CIRs | ZNF208,CILP2,GMIP,ZNF431,ENSG00000267383,ENSG00000141979 | Y | 5.24E-08 |
| rs77254326 | CIRs,TFBRs | RAP2B,ATP13A1,ENSG00000244268,NDUFA13,YJEFN3,SMURF2,ENSG00000258674,PPP1CB,TSSK6,BCL7B | N | 5.25E-08 |
| rs17149781 | circRNAs,TADs | MPP6 | N | 5.26E-08 |
| rs8113006 | TADs | ZNF208,ZNF431,ENSG00000267383,ENSG00000141979 | Y | 5.28E-08 |
| rs79265434 | circRNAs,CIRs | MPP6 | N | 5.31E-08 |
| rs754256 | circRNAs |  | Y | 5.37E-08 |
| rs2060275 | circRNAs |  | Y | 5.38E-08 |
| rs113527843 | TADs,CIRs | ZNF208,CILP2,GMIP,ZNF431,ENSG00000267383,ENSG00000141979 | N | 5.4E-08 |
| rs8100927 | TADs,CIRs | ZNF208,CILP2,GMIP,ZNF431,ENSG00000267383,ENSG00000141979 | Y | 5.41E-08 |
| rs11670761 | TADs,CIRs,TFBRs | ZNF208,ATP13A1,CILP2,YJEFN3,NDUFA13,ZNF431,ENSG00000267383,ENSG00000258674,ENSG00000141979 | Y | 5.43E-08 |
| rs4808968 | TADs,CIRs | ZNF208,CILP2,ZNF431,ENSG00000267383,ENSG00000141979 | Y | 5.43E-08 |
| rs116052126 | circRNAs,TADs | DFNA5,CCDC126 | N | 5.49E-08 |
| rs11669730 | TADs,CIRs,TFBRs | ZNF208,ATP13A1,CILP2,YJEFN3,NDUFA13,ZNF431,ENSG00000267383,ENSG00000258674,ENSG00000141979 | Y | 5.52E-08 |
| rs9714129 | circRNAs,TADs | PBRM1 | Y | 5.54E-08 |
| rs4808969 | TADs,CIRs | ZNF208,CILP2,ZNF431,ENSG00000267383,ENSG00000141979 | Y | 5.55E-08 |
| rs56280531 | circRNAs |  | Y | 5.56E-08 |
| rs75667002 | circRNAs |  | N | 5.57E-08 |
| rs1560687 | circRNAs |  | Y | 5.58E-08 |
| rs74394616 | circRNAs,CIRs | MPP6 | N | 5.66E-08 |
| rs2916075 | circRNAs |  | Y | 5.67E-08 |
| rs4808206 | TADs | ZNF208,ZNF431,ENSG00000267383,ENSG00000141979 | Y | 5.73E-08 |
| rs4808204 | circRNAs |  | Y | 5.77E-08 |
| rs4808970 | TADs,CIRs | ZNF208,ZNF93,ENSG00000267268,LPAR2,ZNF431,ENSG00000267383,ENSG00000141979 | Y | 5.79E-08 |
| rs6979037 | circRNAs,TADs | DFNA5,CCDC126 | Y | 5.82E-08 |
| rs113841472 | circRNAs |  | N | 5.84E-08 |
| rs4808951 | circRNAs |  | Y | 5.86E-08 |
| rs7250233 | circRNAs,CIRs | LPAR2,TSSK6 | Y | 5.88E-08 |
| rs11669558 | TADs | ZNF208,ZNF431,ENSG00000267383,ENSG00000141979 | Y | 5.88E-08 |
| rs76095338 | circRNAs |  | N | 5.89E-08 |
| rs2590838 | circRNAs,TADs | PBRM1 | Y | 5.94E-08 |
| rs75582668 | circRNAs |  | N | 5.96E-08 |
| rs78883953 | circRNAs,CIRs | TSSK6 | N | 5.97E-08 |
| rs7245689 | circRNAs,CIRs | TSSK6 | Y | 5.98E-08 |
| rs2916072 | circRNAs |  | Y | 6.02E-08 |
| rs56330647 | circRNAs,CIRs,TFBRs | ATP13A1,YJEFN3,ENSG00000258674,TSSK6 | Y | 6.05E-08 |
| rs3823198 | circRNAs |  | N | 6.09E-08 |
| rs34162609 | circRNAs |  | Y | 6.09E-08 |
| rs17684164 | CIRs,TFBRs | RAP2B,ATP13A1,ENSG00000244268,NDUFA13,YJEFN3,SMURF2,ENSG00000258674,PPP1CB,TSSK6,BCL7B | Y | 6.09E-08 |
| rs80007081 | CIRs,TFBRs | RAP2B,ATP13A1,ENSG00000244268,NDUFA13,YJEFN3,SMURF2,ENSG00000258674,PPP1CB,TSSK6,BCL7B | N | 6.1E-08 |
| rs2237326 | TADs | DFNA5,CCDC126 | Y | 6.1E-08 |
| rs2336147 | circRNAs,TADs | PBRM1 | Y | 6.18E-08 |
| rs74446114 | circRNAs |  | N | 6.24E-08 |
| rs11509873 | circRNAs,TADs | DFNA5,CCDC126 | N | 6.29E-08 |
| rs174599 | circRNAs |  | Y | 6.34E-08 |
| rs2966422 | TADs | IMMP2L | N | 6.35E-08 |
| rs12898460 | TADs,lncRNAs | PATL2,GPR176,WDR76 | Y | 6.35E-08 |
| rs2965181 | circRNAs |  | Y | 6.38E-08 |
| rs11671253 | circRNAs,CIRs | MAU2 | Y | 6.46E-08 |
| rs7652667 | CIRs | ITIH4 | Y | 6.48E-08 |
| rs147292290 | circRNAs |  | N | 6.51E-08 |
| rs58074958 | circRNAs |  | Y | 6.51E-08 |
| rs2305929 | circRNAs,CIRs,TFBRs,lncRNAs | HIST1H2AG,B9D1,RNU5A-1,HIST1H2BJ,RNVU1-6,ENSG00000233396,RBKS,MAPK7 | Y | 6.56E-08 |
| rs2071042 | CIRs,TFBRs,lncRNAs | ITIH4,PPP3CC | Y | 6.57E-08 |
| rs34667451 | circRNAs |  | Y | 6.58E-08 |
| rs9366029 | circRNAs |  | N | 6.58E-08 |
| rs78010159 | circRNAs |  | N | 6.65E-08 |
| rs4808205 | TADs,CIRs | ZNF208,GMIP,ZNF431,ENSG00000267383,ENSG00000141979 | Y | 6.68E-08 |
| rs79370636 | circRNAs,CIRs | YJEFN3,NDUFA13,ENSG00000258674,TSSK6 | N | 6.69E-08 |
| rs59945804 | TADs | DFNA5,CCDC126 | N | 6.7E-08 |
| rs2965179 | circRNAs,TFBRs | ENSG00000267234 | Y | 6.76E-08 |
| rs4765914 | circRNAs |  | Y | 6.79E-08 |
| rs4441609 | circRNAs,TADs | TRANK1,GOLGA4 | Y | 6.82E-08 |
| rs11707149 | TADs | MOBP,CTDSPL,ENTPD3-AS1 | N | 6.93E-08 |
| rs73205263 | TADs | IMMP2L | N | 6.95E-08 |
| rs61744761 | TADs | ZNF208,ZNF431,ENSG00000267383,ENSG00000141979 | Y | 6.95E-08 |
| rs2966420 | TADs | IMMP2L | N | 7.01E-08 |
| rs12665974 | circRNAs,TADs | DFNA5,CCDC126 | Y | 7.03E-08 |
| rs8108659 | circRNAs |  | Y | 7.04E-08 |
| rs955379 | circRNAs |  | N | 7.12E-08 |
| rs79210963 | circRNAs,TADs | MPP6 | N | 7.16E-08 |
| rs2966424 | TADs | IMMP2L | N | 7.42E-08 |
| rs174594 | circRNAs |  | Y | 7.47E-08 |
| rs7636849 | TADs | MOBP,CTDSPL,ENTPD3-AS1 | N | 7.52E-08 |
| rs13083798 | circRNAs,TADs,CIRs | PBRM1,VPRBP,SMIM4 | Y | 7.54E-08 |
| rs11670775 | TADs,CIRs,TFBRs | ZNF208,ATP13A1,CILP2,YJEFN3,NDUFA13,ZNF431,ENSG00000267383,ENSG00000258674,ENSG00000141979 | Y | 7.56E-08 |
| rs6769789 | circRNAs |  | Y | 7.62E-08 |
| rs2940384 | TADs | IMMP2L | N | 7.84E-08 |
| rs12154597 | circRNAs,TADs | DFNA5,CCDC126 | Y | 7.98E-08 |
| rs79971637 | circRNAs,TADs | DFNA5,CCDC126 | N | 8.04E-08 |
| rs75678193 | circRNAs,TADs | DFNA5,CCDC126 | N | 8.05E-08 |
| rs12290811 | circRNAs |  | Y | 8.15E-08 |
| rs2256332 | CIRs | NEK4 | Y | 8.17E-08 |
| rs11922961 | TFBRs | MUSTN1 | Y | 8.24E-08 |
| rs174578 | circRNAs |  | Y | 8.33E-08 |
| rs16955864 | TADs,TFBRs,lncRNAs | PCTP,ENSG00000262079 | N | 8.38E-08 |
| rs2916070 | circRNAs |  | Y | 8.41E-08 |
| rs12912934 | TFBRs,lncRNAs | UBE2Q2P1,LINC00933 | Y | 8.43E-08 |
| rs1014969 | TADs | VPRBP | Y | 8.45E-08 |
| rs763193 | circRNAs |  | N | 8.47E-08 |
| rs78176666 | circRNAs,CIRs | GMIP,TSSK6 | N | 8.65E-08 |
| rs12286448 | circRNAs,TADs | TENM4,JRKL,JRKL-AS1,DLG2 | Y | 8.66E-08 |
| rs75453386 | circRNAs,CIRs | TSSK6 | N | 8.66E-08 |
| rs111406321 | circRNAs,CIRs | TSSK6 | N | 8.68E-08 |
| rs7127580 | circRNAs,TADs | TENM4,JRKL,JRKL-AS1,DLG2 | N | 8.74E-08 |
| rs75525243 | circRNAs,CIRs | YJEFN3,ENSG00000258674,TSSK6 | N | 8.74E-08 |
| rs45631651 | circRNAs,CIRs | YJEFN3,ENSG00000258674,TSSK6 | Y | 8.77E-08 |
| rs75107726 | circRNAs,TADs | DFNA5,CCDC126 | N | 8.82E-08 |
| rs74950305 | CIRs | YJEFN3,ENSG00000258674,TSSK6 | N | 8.87E-08 |
| rs11633377 | circRNAs |  | Y | 9.2E-08 |
| rs62019463 | CIRs | CACYBP | Y | 9.24E-08 |
| rs79604156 | circRNAs,TADs | TENM4,JRKL,JRKL-AS1,DLG2 | N | 9.25E-08 |
| rs77162489 | circRNAs,TADs | DFNA5,CCDC126 | N | 9.25E-08 |
| rs3804640 | circRNAs |  | N | 9.27E-08 |
| rs79251484 | circRNAs,TADs | DFNA5,CCDC126 | N | 9.3E-08 |
| rs34395289 | TFBRs | LINC00933 | Y | 9.33E-08 |
| rs181957530 | circRNAs |  | N | 9.37E-08 |
| rs12905223 | CIRs,TFBRs | UBE2Q2P1,LINC00933 | Y | 9.38E-08 |
| rs11079161 | TADs,TFBRs | PCTP,ENSG00000262079 | Y | 9.38E-08 |
| rs17600551 | circRNAs |  | Y | 9.49E-08 |
| rs8102502 | TADs,CIRs | ZNF208,ZNF93,ATP13A1,ENSG00000267268,ZNF431,ENSG00000267383,ENSG00000141979,TSSK6 | Y | 9.5E-08 |
| rs174601 | circRNAs |  | Y | 9.5E-08 |
| rs36033486 | circRNAs |  | Y | 9.5E-08 |
| rs62019469 | circRNAs |  | Y | 9.53E-08 |
| rs12908549 | circRNAs |  | Y | 9.59E-08 |
| rs78436753 | circRNAs,TADs | DFNA5,CCDC126 | N | 9.59E-08 |
| rs56291054 | circRNAs |  | Y | 9.64E-08 |
| rs34487417 | circRNAs |  | Y | 9.66E-08 |
| rs906482 | TADs | MOBP,CTDSPL,ENTPD3-AS1 | Y | 9.73E-08 |
| rs12665866 | circRNAs,TADs | DFNA5,CCDC126 | N | 9.75E-08 |
| rs11637142 | CIRs,TFBRs | WDR73,ENSG00000259415 | Y | 9.8E-08 |
| rs12703284 | TADs | DENND2A | Y | 9.81E-08 |
| rs12912251 | TADs,lncRNAs | PATL2,GPR176,WDR76 | Y | 9.84E-08 |
| rs11719137 | circRNAs |  | Y | 9.92E-08 |
| rs35758837 | circRNAs |  | Y | 9.98E-08 |
| rs12899981 | circRNAs |  | Y | 1E-07 |
| rs11632465 | circRNAs |  | Y | 1.01E-07 |
| rs28522146 | TADs | TRANK1,GOLGA4 | N | 1.01E-07 |
| rs12202969 | circRNAs |  | Y | 1.02E-07 |
| rs62019464 | CIRs,TFBRs | WDR73,ENSG00000259415 | Y | 1.02E-07 |
| rs6445541 | circRNAs |  | Y | 1.02E-07 |
| rs56019197 | circRNAs |  | Y | 1.03E-07 |
| rs71395453 | CIRs,TFBRs | UBE2Q2P1,LINC00933 | Y | 1.03E-07 |
| rs9813869 | TADs | DLEC1,CCDC66,TRANK1 | Y | 1.03E-07 |
| rs9985296 | circRNAs,TADs | TRANK1,GOLGA4 | Y | 1.05E-07 |
| rs3803403 | circRNAs,CIRs | ZNF592 | Y | 1.06E-07 |
| rs2276823 | TFBRs | MUSTN1,ENSG00000243696 | Y | 1.06E-07 |
| rs11633267 | circRNAs |  | Y | 1.06E-07 |
| rs2940396 | TADs | IMMP2L | N | 1.07E-07 |
| rs12903120 | TADs,TFBRs | PATL2,GPR176,C15orf53,WDR76 | Y | 1.07E-07 |
| rs12275127 | circRNAs,TADs | TENM4,JRKL,JRKL-AS1,DLG2 | N | 1.08E-07 |
| rs4808962 | circRNAs |  | Y | 1.08E-07 |
| rs4447398 | circRNAs,TADs | STARD9,RTF1 | N | 1.1E-07 |
| rs56214994 | circRNAs |  | Y | 1.1E-07 |
| rs11632169 | circRNAs,TADs | STARD9,RTF1 | Y | 1.1E-07 |
| rs35102843 | TADs,TFBRs | PATL2,GPR176,C15orf53,WDR76 | N | 1.11E-07 |
| rs11915546 | CIRs | ITIH4 | Y | 1.12E-07 |
| rs12903134 | circRNAs |  | Y | 1.13E-07 |
| rs35726233 | circRNAs |  | Y | 1.14E-07 |
| rs1529745 | circRNAs,CIRs | DDX49,ASXL1 | Y | 1.14E-07 |
| rs174581 | circRNAs,CIRs | MIR1908 | Y | 1.15E-07 |
| rs10035291 | circRNAs |  | N | 1.15E-07 |
| rs1197546 | circRNAs,TADs | STARD9,RTF1 | N | 1.15E-07 |
| rs174580 | circRNAs,CIRs | MIR1908 | Y | 1.15E-07 |
| rs34117475 | CIRs | LINC00933 | Y | 1.17E-07 |
| rs6896235 | circRNAs |  | N | 1.18E-07 |
| rs12906474 | CIRs | LINC00933 | Y | 1.19E-07 |
| rs765090 | circRNAs |  | N | 1.19E-07 |
| rs2019065 | TADs | VPRBP | Y | 1.2E-07 |
| rs7163869 | TADs,TFBRs,lncRNAs | PATL2,GPR176,C15orf53,WDR76 | Y | 1.21E-07 |
| rs6871860 | circRNAs |  | N | 1.21E-07 |
| rs2336558 | circRNAs |  | Y | 1.22E-07 |
| rs11647445 | circRNAs |  | N | 1.22E-07 |
| rs11715347 | circRNAs |  | Y | 1.22E-07 |
| rs6895882 | circRNAs |  | N | 1.22E-07 |
| rs174577 | circRNAs |  | Y | 1.22E-07 |
| rs733591 | circRNAs |  | N | 1.22E-07 |
| rs34921234 | circRNAs |  | Y | 1.23E-07 |
| rs184582541 | circRNAs |  | Y | 1.24E-07 |
| rs3803405 | circRNAs,CIRs | ZNF592 | Y | 1.24E-07 |
| rs9372734 | circRNAs |  | N | 1.27E-07 |
| rs4642070 | circRNAs,TADs | TRANK1,GOLGA4 | N | 1.27E-07 |
| rs6891181 | circRNAs |  | N | 1.27E-07 |
| rs12904605 | CIRs,TFBRs | EGLN1P1,NMB | Y | 1.27E-07 |
| rs6880200 | circRNAs |  | N | 1.27E-07 |
| rs4626317 | circRNAs |  | N | 1.3E-07 |
| rs579518 | TADs | DENND2A | Y | 1.3E-07 |
| rs56159542 | circRNAs |  | Y | 1.3E-07 |
| rs34281605 | TADs,lncRNAs | PATL2,GPR176,WDR76 | N | 1.32E-07 |
| rs6874029 | circRNAs |  | N | 1.32E-07 |
| rs174584 | circRNAs,CIRs | BEST1 | Y | 1.32E-07 |
| rs7171233 | TADs,TFBRs,lncRNAs | PATL2,GPR176,C15orf53,WDR76 | Y | 1.32E-07 |
| rs34356257 | TADs,TFBRs,lncRNAs | PATL2,GPR176,C15orf53,WDR76 | N | 1.33E-07 |
| rs34141940 | TADs,lncRNAs | PATL2,GPR176,WDR76 | N | 1.33E-07 |
| rs7165988 | TADs,TFBRs,lncRNAs | PATL2,GPR176,C15orf53,WDR76 | Y | 1.35E-07 |
| rs58416181 | CIRs | SEC11A | Y | 1.35E-07 |
| rs12899953 | TFBRs | UBE2Q2P1,LINC00933 | Y | 1.35E-07 |
| rs6958468 | TADs | DENND2A | Y | 1.35E-07 |
| rs10774037 | circRNAs |  | Y | 1.37E-07 |
| rs17827795 | circRNAs |  | Y | 1.37E-07 |
| rs968050 | circRNAs |  | N | 1.4E-07 |
| rs60957376 | circRNAs |  | Y | 1.4E-07 |
| rs1961288 | TADs | SSBP1,DENND2A | Y | 1.4E-07 |
| rs9892737 | TADs | PCTP | N | 1.4E-07 |
| rs71403553 | TADs,TFBRs,lncRNAs | PATL2,GPR176,C15orf53,WDR76 | N | 1.41E-07 |
| rs2940390 | TADs | IMMP2L | N | 1.43E-07 |
| rs3829331 | circRNAs |  | N | 1.44E-07 |
| rs2136009 | TADs | IMMP2L | N | 1.44E-07 |
| rs57880522 | TADs | IMMP2L | N | 1.45E-07 |
| rs2966427 | TADs | IMMP2L | N | 1.45E-07 |
| rs72941253 | circRNAs,CIRs | LMAN2L | N | 1.48E-07 |
| rs35738019 | circRNAs |  | Y | 1.48E-07 |
| rs4726225 | TADs | SSBP1,DENND2A | Y | 1.49E-07 |
| rs11630760 | CIRs,TFBRs | LINC00933 | Y | 1.49E-07 |
| rs17183814 | circRNAs,TADs | COBLL1,SCN2A | Y | 1.49E-07 |
| rs10272579 | TADs,TFBRs | IMMP2L,ENSG00000226965 | N | 1.5E-07 |
| rs6792369 | TADs | VPRBP | Y | 1.51E-07 |
| rs4513668 | circRNAs |  | N | 1.54E-07 |
| rs12333992 | TADs | IMMP2L | N | 1.55E-07 |
| rs77353560 | circRNAs |  | N | 1.56E-07 |
| rs2126180 | TFBRs | ENSG00000226476 | N | 1.57E-07 |
| rs3843751 | circRNAs,CIRs | SLC44A2 | Y | 1.6E-07 |
| rs102275 | circRNAs,TADs | RTN3 | Y | 1.62E-07 |
| rs2163832 | circRNAs,CIRs | ILF3,ILF3-AS1 | Y | 1.63E-07 |
| rs220333 | circRNAs |  | Y | 1.66E-07 |
| rs10942269 | circRNAs |  | N | 1.66E-07 |
| rs174576 | circRNAs |  | Y | 1.68E-07 |
| rs56233902 | circRNAs |  | Y | 1.7E-07 |
| rs3843750 | circRNAs,CIRs | SLC44A2 | Y | 1.71E-07 |
| rs11633450 | circRNAs |  | Y | 1.72E-07 |
| rs7702047 | circRNAs |  | N | 1.73E-07 |
| rs9882924 | circRNAs,TADs | DLEC1,CCDC66,TRANK1 | N | 1.74E-07 |
| rs12912388 | circRNAs |  | Y | 1.75E-07 |
| rs34570071 | circRNAs |  | Y | 1.78E-07 |
| rs12914760 | circRNAs,CIRs | ZNF592 | Y | 1.79E-07 |
| rs998909 | TADs,CIRs,TFBRs | CYCS,ITIH4,PBRM1,VPRBP,NEK4 | Y | 1.79E-07 |
| rs6890850 | circRNAs |  | N | 1.84E-07 |
| rs35960805 | circRNAs,CIRs | ZNF592 | Y | 1.85E-07 |
| rs6867426 | circRNAs |  | N | 1.85E-07 |
| rs12289486 | circRNAs |  | N | 1.88E-07 |
| rs174593 | circRNAs |  | Y | 1.89E-07 |
| rs174583 | circRNAs,CIRs | BEST1 | Y | 1.91E-07 |
| rs11630507 | circRNAs |  | Y | 1.95E-07 |
| rs11638290 | circRNAs,TFBRs | ENSG00000259654 | Y | 1.97E-07 |
| rs13071584 | circRNAs,TADs,CIRs,TFBRs | ITIH4,VPRBP,NEK4 | Y | 1.97E-07 |
| rs11633413 | circRNAs |  | Y | 2.04E-07 |
| rs4328757 | circRNAs,TADs | DLEC1,CCDC66,TRANK1 | Y | 2.05E-07 |
| rs12629687 | circRNAs |  | Y | 2.06E-07 |
| rs925135 | TADs | MOBP,CTDSPL,ENTPD3-AS1 | N | 2.07E-07 |
| rs10896090 | circRNAs |  | Y | 2.08E-07 |
| rs34028043 | circRNAs |  | Y | 2.09E-07 |
| rs1487445 | circRNAs |  | N | 2.09E-07 |
| rs6767302 | CIRs | ATP1B3 | Y | 2.12E-07 |
| rs1114603 | circRNAs |  | N | 2.14E-07 |
| rs696366 | lncRNAs |  | N | 2.18E-07 |
| rs7720736 | circRNAs |  | N | 2.2E-07 |
| rs12577328 | circRNAs |  | N | 2.2E-07 |
| rs968470 | CIRs | CNNM4 | Y | 2.22E-07 |
| rs7122539 | circRNAs |  | Y | 2.23E-07 |
| rs12908699 | circRNAs |  | Y | 2.23E-07 |
| rs12911736 | circRNAs |  | Y | 2.23E-07 |
| rs12917221 | TADs | PATL2,GPR176,WDR76 | N | 2.24E-07 |
| rs79822078 | circRNAs |  | N | 2.25E-07 |
| rs111460293 | circRNAs |  | N | 2.25E-07 |
| rs11638554 | circRNAs |  | Y | 2.25E-07 |
| rs11237816 | circRNAs |  | N | 2.25E-07 |
| rs138324 | TADs,TFBRs | MIR4766,TNRC6B | N | 2.25E-07 |
| rs11636499 | circRNAs |  | Y | 2.27E-07 |
| rs34784022 | circRNAs,CIRs | ZNF592 | Y | 2.29E-07 |
| rs11062148 | circRNAs |  | N | 2.3E-07 |
| rs35316992 | circRNAs |  | Y | 2.33E-07 |
| rs4643294 | circRNAs |  | Y | 2.33E-07 |
| rs7733938 | circRNAs |  | N | 2.34E-07 |
| rs748455 | circRNAs,CIRs | EGLN1P1 | Y | 2.37E-07 |
| rs148001625 | circRNAs,CIRs | LPAR2 | N | 2.38E-07 |
| rs11237815 | circRNAs |  | N | 2.38E-07 |
| rs11130331 | circRNAs |  | Y | 2.39E-07 |
| rs35524990 | circRNAs |  | Y | 2.4E-07 |
| rs12497998 | circRNAs,TADs | NEK4 | Y | 2.41E-07 |
| rs62021219 | circRNAs |  | Y | 2.41E-07 |
| rs6910053 | circRNAs |  | N | 2.42E-07 |
| rs7477704 | circRNAs |  | N | 2.45E-07 |
| rs35024396 | TADs,lncRNAs | PATL2,GPR176,WDR76 | N | 2.45E-07 |
| rs6897189 | circRNAs |  | N | 2.47E-07 |
| rs4296548 | circRNAs,TADs | TRANK1,GOLGA4 | Y | 2.5E-07 |
| rs12273727 | circRNAs,TADs | TENM4,JRKL,JRKL-AS1,DLG2 | N | 2.5E-07 |
| rs34900908 | circRNAs |  | Y | 2.5E-07 |
| rs34452033 | circRNAs |  | Y | 2.53E-07 |
| rs10218679 | circRNAs,lncRNAs |  | N | 2.54E-07 |
| rs35958438 | TADs | PATL2,GPR176,WDR76 | N | 2.54E-07 |
| rs6617 | CIRs,TFBRs,lncRNAs | GLT8D1,,RPL41,C7orf73,SMIM4 | Y | 2.55E-07 |
| rs696364 | circRNAs |  | N | 2.56E-07 |
| rs4426897 | circRNAs |  | N | 2.57E-07 |
| rs7555117 | circRNAs |  | N | 2.58E-07 |
| rs11194997 | circRNAs |  | N | 2.59E-07 |
| rs35481323 | TADs | MOBP,CTDSPL,ENTPD3-AS1 | N | 2.59E-07 |
| rs2230534 | circRNAs,TADs | VPRBP | Y | 2.6E-07 |
| rs7223718 | circRNAs |  | Y | 2.61E-07 |
| rs2857482 | TADs | HCRTR2,PKHD1,PRIM2 | N | 2.64E-07 |
| rs11635505 | circRNAs |  | Y | 2.65E-07 |
| rs7252453 | TADs | ZNF208,ZNF431,ENSG00000267383,ENSG00000141979 | Y | 2.66E-07 |
| rs7809025 | TADs | SSBP1,DENND2A | Y | 2.69E-07 |
| rs3748376 | circRNAs |  | Y | 2.72E-07 |
| rs6452419 | circRNAs |  | N | 2.72E-07 |
| rs2172835 | TADs | PATL2,GPR176,WDR76 | Y | 2.72E-07 |
| rs12268910 | circRNAs |  | Y | 2.73E-07 |
| rs13023194 | lncRNAs |  | N | 2.73E-07 |
| rs13072940 | TADs | MOBP,CTDSPL,ENTPD3-AS1 | Y | 2.73E-07 |
| rs10791855 | circRNAs |  | N | 2.73E-07 |
| rs10948 | circRNAs,CIRs | ILF3,ILF3-AS1 | Y | 2.75E-07 |
| rs174574 | circRNAs |  | Y | 2.75E-07 |
| rs7707981 | circRNAs |  | N | 2.76E-07 |
| rs3015338 | circRNAs,lncRNAs |  | N | 2.76E-07 |
| rs4765904 | circRNAs |  | N | 2.77E-07 |
| rs3900556 | circRNAs |  | N | 2.77E-07 |
| rs2293121 | circRNAs |  | N | 2.77E-07 |
| rs3872223 | circRNAs |  | N | 2.78E-07 |
| rs566862 | circRNAs,lncRNAs |  | N | 2.79E-07 |
| rs484743 | circRNAs |  | N | 2.82E-07 |
| rs4687689 | circRNAs |  | Y | 2.82E-07 |
| rs1535 | circRNAs,TFBRs | FADS1 | Y | 2.85E-07 |
| rs12906348 | circRNAs,CIRs | ZNF592 | Y | 2.85E-07 |
| rs34342559 | circRNAs |  | Y | 2.85E-07 |
| rs62019472 | circRNAs |  | Y | 2.87E-07 |
| rs10041030 | circRNAs |  | N | 2.88E-07 |
| rs35557864 | circRNAs |  | Y | 2.88E-07 |
| rs3900555 | circRNAs |  | N | 2.89E-07 |
| rs2177054 | circRNAs |  | N | 2.9E-07 |
| rs10791850 | circRNAs |  | N | 2.9E-07 |
| rs2079978 | TADs | SSBP1,DENND2A | Y | 2.91E-07 |
| rs10791854 | circRNAs |  | N | 2.91E-07 |
| rs12907808 | circRNAs |  | Y | 2.92E-07 |
| rs6786043 | circRNAs,TADs | PBRM1 | Y | 2.93E-07 |
| rs528598 | circRNAs |  | N | 2.93E-07 |
| rs8103992 | TADs | ZNF208,ZNF431,ENSG00000267383,ENSG00000141979 | Y | 2.96E-07 |
| rs9758945 | circRNAs,TADs | PBRM1 | Y | 2.96E-07 |
| rs9880619 | circRNAs |  | Y | 2.96E-07 |
| rs2230535 | circRNAs,TADs | VPRBP | Y | 2.97E-07 |
| rs6727384 | circRNAs |  | Y | 2.99E-07 |
| rs11062145 | circRNAs |  | N | 3E-07 |
| rs2889313 | circRNAs |  | N | 3.02E-07 |
| rs2251219 | circRNAs,TADs,CIRs | CADPS,FHIT,RNU6-856P,CACNA2D3,CACNA2D2 | Y | 3.02E-07 |
| rs3953856 | circRNAs |  | N | 3.02E-07 |
| rs68026712 | circRNAs |  | N | 3.02E-07 |
| rs9871175 | circRNAs |  | Y | 3.02E-07 |
| rs1108222 | circRNAs |  | N | 3.03E-07 |
| rs12940636 | TADs | PCTP | Y | 3.08E-07 |
| rs6788993 | circRNAs,TADs | PBRM1 | Y | 3.09E-07 |
| rs552294 | circRNAs |  | N | 3.09E-07 |
| rs610060 | CIRs,TFBRs,lncRNAs | USP53,TWF2,ENSG00000178636,ACP1,ENSG00000239557,ENSG00000243224,TLR9 | Y | 3.1E-07 |
| rs77898373 | circRNAs |  | N | 3.1E-07 |
| rs10896093 | circRNAs,CIRs | PACS1,,ENSG00000255038 | N | 3.11E-07 |
| rs1108221 | circRNAs |  | N | 3.12E-07 |
| rs4239192 | circRNAs |  | Y | 3.13E-07 |
| rs6787154 | circRNAs,TADs | NEK4 | Y | 3.13E-07 |
| rs10405617 | circRNAs,CIRs | ILF3,ILF3-AS1 | Y | 3.14E-07 |
| rs2150211 | circRNAs |  | N | 3.14E-07 |
| rs928213 | circRNAs |  | N | 3.18E-07 |
| rs10896098 | circRNAs |  | N | 3.21E-07 |
| rs6534354 | TADs | SPATA5 | N | 3.21E-07 |
| rs7969091 | TFBRs | RHEBL1 | Y | 3.25E-07 |
| rs10896095 | circRNAs |  | N | 3.28E-07 |
| rs13074853 | circRNAs,TADs | NEK4 | Y | 3.29E-07 |
| rs10791856 | circRNAs |  | N | 3.3E-07 |
| rs33964154 | circRNAs,TADs | CADPS,FHIT,CACNA2D3,CACNA2D2 | Y | 3.3E-07 |
| rs13063160 | circRNAs,TADs | PBRM1 | Y | 3.31E-07 |
| rs2072390 | circRNAs,TADs | NEK4 | Y | 3.32E-07 |
| rs174528 | circRNAs,TADs | RTN3 | Y | 3.32E-07 |
| rs11062147 | circRNAs |  | N | 3.33E-07 |
| rs925549 | TADs | SPATA5 | N | 3.33E-07 |
| rs78381888 | circRNAs |  | Y | 3.36E-07 |
| rs4687687 | circRNAs |  | Y | 3.37E-07 |
| rs6445535 | circRNAs,TADs | NEK4 | Y | 3.37E-07 |
| rs2221903 | TADs | SPATA5 | N | 3.37E-07 |
| rs11715554 | CIRs | MLH1 | Y | 3.37E-07 |
| rs11062166 | circRNAs |  | N | 3.38E-07 |
| rs1866268 | TADs,CIRs,TFBRs | KMT2E,MALAT1,ENSG00000270117,EIF4A1,PBRM1,SNORA67,TM9SF3,LINC01004 | Y | 3.38E-07 |
| rs878919 | lncRNAs |  | N | 3.38E-07 |
| rs56080538 | circRNAs |  | Y | 3.39E-07 |
| rs10745843 | circRNAs |  | N | 3.41E-07 |
| rs11637728 | TFBRs | NMB | Y | 3.41E-07 |
| rs6788887 | circRNAs,TADs | PBRM1 | Y | 3.42E-07 |
| rs2738039 | circRNAs,CIRs | ILF3 | Y | 3.43E-07 |
| rs10407923 | circRNAs |  | Y | 3.44E-07 |
| rs9865248 | circRNAs |  | Y | 3.44E-07 |
| rs10804056 | TADs | STAT4 | N | 3.44E-07 |
| rs103197 | CIRs | ST13 | Y | 3.45E-07 |
| rs10848642 | circRNAs,lncRNAs |  | Y | 3.46E-07 |
| rs2940394 | TADs | IMMP2L | N | 3.51E-07 |
| rs6464217 | CIRs | BRAF | Y | 3.52E-07 |
| rs9985365 | circRNAs |  | Y | 3.52E-07 |
| rs3755798 | CIRs,TFBRs | GLT8D1,SMIM4 | Y | 3.52E-07 |
| rs4283765 | circRNAs |  | N | 3.54E-07 |
| rs11714030 | circRNAs,TADs | NEK4 | Y | 3.54E-07 |
| rs4392600 | circRNAs |  | N | 3.54E-07 |
| rs10501439 | circRNAs |  | Y | 3.55E-07 |
| rs2360942 | CIRs | ILF3-AS1 | Y | 3.56E-07 |
| rs2569512 | circRNAs |  | Y | 3.57E-07 |
| rs2190771 | circRNAs |  | N | 3.57E-07 |
| rs6730773 | CIRs | CNNM4 | N | 3.58E-07 |
| rs34739010 | circRNAs,TADs | NEK4 | Y | 3.59E-07 |
| rs7638162 | circRNAs,TADs | NEK4 | Y | 3.59E-07 |
| rs10896094 | circRNAs |  | N | 3.61E-07 |
| rs6464260 | TADs | SSBP1,DENND2A | N | 3.61E-07 |
| rs12635140 | circRNAs |  | Y | 3.62E-07 |
| rs7725320 | circRNAs |  | N | 3.62E-07 |
| rs7124165 | circRNAs,CIRs | GAL3ST3 | N | 3.64E-07 |
| rs17052259 | circRNAs,TADs,CIRs | CADPS,FHIT,SMIM4,CACNA2D3,CACNA2D2 | Y | 3.66E-07 |
| rs17052256 | circRNAs,TADs,CIRs | CADPS,FHIT,SMIM4,CACNA2D3,CACNA2D2 | Y | 3.66E-07 |
| rs13059862 | circRNAs,TADs | CADPS,FHIT,CACNA2D3,CACNA2D2 | Y | 3.66E-07 |
| rs767418 | circRNAs,TADs,CIRs,TFBRs | ENSG00000270941,NEK4 | Y | 3.68E-07 |
| rs3796353 | circRNAs,TADs,CIRs | CADPS,FHIT,SMIM4,CACNA2D3,CACNA2D2 | Y | 3.71E-07 |
| rs6065777 | circRNAs,CIRs | STK4-AS1 | Y | 3.73E-07 |
| rs7941431 | circRNAs |  | N | 3.73E-07 |
| rs11085260 | circRNAs |  | Y | 3.73E-07 |
| rs7929909 | circRNAs |  | N | 3.74E-07 |
| rs13234490 | TADs | SSBP1,DENND2A | Y | 3.74E-07 |
| rs1570 | circRNAs,TADs,TFBRs | CADPS,FHIT,RNU6-856P,CACNA2D3,CACNA2D2 | Y | 3.74E-07 |
| rs7931477 | circRNAs |  | N | 3.75E-07 |
| rs2164885 | circRNAs |  | Y | 3.75E-07 |
| rs3922316 | circRNAs |  | N | 3.75E-07 |
| rs1560707 | circRNAs,TFBRs | ENSG00000226104 | Y | 3.75E-07 |
| rs13079063 | CIRs | ENSG00000270941 | Y | 3.76E-07 |
| rs12148859 | TADs | PATL2,GPR176,WDR76 | N | 3.77E-07 |
| rs10750778 | circRNAs |  | N | 3.78E-07 |
| rs1860720 | TADs | SSBP1,DENND2A | Y | 3.78E-07 |
| rs2289247 | circRNAs,CIRs,TFBRs | SNORD69 | Y | 3.79E-07 |
| rs1108075 | circRNAs,TFBRs | CACNA1C-AS4 | N | 3.8E-07 |
| rs7946917 | circRNAs |  | N | 3.81E-07 |
| rs4833837 | TADs | SPATA5 | N | 3.81E-07 |
| rs12261598 | circRNAs |  | N | 3.82E-07 |
| rs12800670 | circRNAs |  | N | 3.82E-07 |
| rs1560708 | circRNAs |  | Y | 3.83E-07 |
| rs28565152 | circRNAs |  | N | 3.83E-07 |
| rs9992580 | TADs | SPATA5 | N | 3.83E-07 |
| rs10224312 | TADs | SSBP1,DENND2A | Y | 3.83E-07 |
| rs10791851 | circRNAs |  | N | 3.84E-07 |
| rs9736673 | circRNAs |  | N | 3.84E-07 |
| rs55678971 | TADs | PBRM1 | Y | 3.85E-07 |
| rs10068144 | circRNAs |  | N | 3.85E-07 |
| rs10791852 | circRNAs |  | N | 3.85E-07 |
| rs7943911 | circRNAs |  | N | 3.85E-07 |
| rs6786919 | circRNAs,TADs | CADPS,FHIT,CACNA2D3,CACNA2D2 | Y | 3.86E-07 |
| rs2079929 | circRNAs,TADs | NEK4 | Y | 3.87E-07 |
| rs7947802 | circRNAs |  | N | 3.89E-07 |
| rs10896086 | circRNAs |  | N | 3.89E-07 |
| rs6591208 | circRNAs |  | N | 3.89E-07 |
| rs10042303 | circRNAs |  | N | 3.91E-07 |
| rs11227426 | circRNAs |  | N | 3.91E-07 |
| rs17534709 | circRNAs |  | Y | 3.91E-07 |
| rs1398553 | TADs | SPATA5 | N | 3.92E-07 |
| rs1982075 | circRNAs |  | Y | 3.93E-07 |
| rs11227420 | circRNAs,CIRs | PACS1, | N | 3.94E-07 |
| rs10777957 | circRNAs |  | N | 3.96E-07 |
| rs3733041 | circRNAs |  | Y | 3.96E-07 |
| rs3774366 | circRNAs,TADs | PBRM1,VPRBP | Y | 3.97E-07 |
| rs9917834 | circRNAs,TADs | PBRM1 | Y | 3.99E-07 |
| rs34754793 | circRNAs,TADs | PBRM1 | Y | 3.99E-07 |
| rs4762559 | circRNAs,lncRNAs |  | Y | 3.99E-07 |
| rs2159644 | circRNAs,TADs | NEK4 | Y | 4E-07 |
| rs11129743 | CIRs | MLH1 | Y | 4E-07 |
| rs5758123 | circRNAs |  | Y | 4.01E-07 |
| rs6814458 | TADs | SPATA5 | N | 4.03E-07 |
| rs11130308 | circRNAs,TADs | PBRM1 | Y | 4.04E-07 |
| rs10239770 | TADs | SSBP1,DENND2A | Y | 4.05E-07 |
| rs2878628 | circRNAs,TADs,CIRs | CADPS,FHIT,RNU6-856P,CACNA2D3,CACNA2D2 | Y | 4.05E-07 |
| rs99780 | circRNAs,TFBRs | FADS1 | Y | 4.06E-07 |
| rs2857504 | TADs | HCRTR2,PKHD1,PRIM2 | N | 4.06E-07 |
| rs10896089 | circRNAs,CIRs | PACS1, | N | 4.07E-07 |
| rs9401593 | circRNAs,TADs | FAXC,ENSG00000271860 | N | 4.08E-07 |
| rs2857501 | TADs | HCRTR2,PKHD1,PRIM2 | N | 4.08E-07 |
| rs62253700 | circRNAs,TADs | PBRM1 | Y | 4.09E-07 |
| rs12424245 | circRNAs |  | N | 4.09E-07 |
| rs7717663 | circRNAs |  | N | 4.1E-07 |
| rs1398554 | TADs | SPATA5 | N | 4.12E-07 |
| rs11235 | circRNAs,CIRs | ENSG00000270941 | Y | 4.13E-07 |
| rs7801518 | CIRs | MRPS33 | Y | 4.14E-07 |
| rs2177053 | circRNAs |  | N | 4.15E-07 |
| rs4274955 | circRNAs |  | N | 4.15E-07 |
| rs6511708 | circRNAs |  | Y | 4.17E-07 |
| rs10896092 | circRNAs,CIRs | PACS1,,ENSG00000255038 | N | 4.18E-07 |
| rs102274 | circRNAs,TADs | RTN3 | Y | 4.18E-07 |
| rs7926402 | circRNAs |  | N | 4.19E-07 |
| rs11062159 | circRNAs |  | N | 4.2E-07 |
| rs10459221 | CIRs | ENSG00000258101 | Y | 4.2E-07 |
| rs2744504 | TADs | HCRTR2,PKHD1,PRIM2 | N | 4.22E-07 |
| rs2738042 | circRNAs |  | Y | 4.23E-07 |
| rs112209391 | TADs,CIRs,TFBRs,lncRNAs | TENM4,,MIR5579,JRKL,JRKL-AS1,DLG2 | N | 4.23E-07 |
| rs9375188 | circRNAs,TADs | FAXC,ENSG00000271860 | N | 4.24E-07 |
| rs61236902 | circRNAs |  | N | 4.26E-07 |
| rs11908600 | circRNAs,TADs,TFBRs | ENSG00000234948,ENSG00000252021,STK4,BCAS4 | Y | 4.26E-07 |
| rs6804145 | circRNAs,TADs,CIRs | PBRM1 | Y | 4.27E-07 |
| rs2336149 | circRNAs,TADs | PBRM1 | Y | 4.28E-07 |
| rs4013917 | circRNAs,CIRs | PACS1,,ENSG00000255038 | N | 4.28E-07 |
| rs12797586 | circRNAs,CIRs | PACS1,,GAL3ST3,ENSG00000255038 | N | 4.3E-07 |
| rs1476816 | TFBRs | MRPS33 | Y | 4.31E-07 |
| rs9630101 | circRNAs,TADs | SORCS1,SHOC2 | Y | 4.31E-07 |
| rs7937281 | circRNAs |  | N | 4.33E-07 |
| rs2360943 | CIRs | ILF3-AS1 | Y | 4.34E-07 |
| rs1533933 | CIRs,TFBRs | EIF4G2,MRPS33,ENSG00000246308,SUV420H1 | Y | 4.34E-07 |
| rs10896087 | circRNAs |  | N | 4.35E-07 |
| rs13195969 | miRNA target site | TFAP2B | N | 4.35E-07 |
| rs6584972 | CIRs | MXI1 | N | 4.37E-07 |
| rs7217151 | circRNAs,TADs | CA10,PLEKHM1,LRRC37A3 | Y | 4.37E-07 |
| rs4762557 | circRNAs |  | N | 4.38E-07 |
| rs7676523 | TADs | SPATA5 | N | 4.38E-07 |
| rs7611731 | circRNAs,TADs,CIRs | PBRM1 | Y | 4.39E-07 |
| rs6090435 | TADs,CIRs | TAF4,OPRL1,DIDO1 | N | 4.39E-07 |
| rs4726196 | TADs | SSBP1,DENND2A | Y | 4.4E-07 |
| rs13061423 | circRNAs,TADs | NEK4 | Y | 4.4E-07 |
| rs1044927 | lncRNAs |  | Y | 4.4E-07 |
| rs6770463 | circRNAs,TADs | PBRM1 | Y | 4.41E-07 |
| rs10745841 | circRNAs |  | N | 4.42E-07 |
| rs1487441 | circRNAs,TADs | FAXC,ENSG00000271860 | N | 4.43E-07 |
| rs8102380 | circRNAs |  | Y | 4.43E-07 |
| rs35315313 | circRNAs,CIRs,TFBRs | SNORD69,ENSG00000222345 | Y | 4.46E-07 |
| rs13060048 | circRNAs,TADs | CADPS,FHIT,CACNA2D3,CACNA2D2 | Y | 4.46E-07 |
| rs55653665 | circRNAs |  | N | 4.46E-07 |
| rs12797158 | circRNAs |  | N | 4.46E-07 |
| rs7676741 | TADs | SPATA5 | N | 4.47E-07 |
| rs2965186 | circRNAs,CIRs,TFBRs | MEF2B,ATP13A1,TSR1,SGSM2,RPL15,GATAD2A,MEF2BNB,ATF1,HNRNPAB | Y | 4.48E-07 |
| rs8067817 | TADs | ENSG00000174093,CDK12 | Y | 4.5E-07 |
| rs10745842 | circRNAs |  | Y | 4.51E-07 |
| rs964102 | circRNAs |  | N | 4.51E-07 |
| rs580891 | circRNAs |  | N | 4.53E-07 |
| rs471484 | circRNAs |  | N | 4.54E-07 |
| rs10875917 | CIRs | ENSG00000258101 | Y | 4.55E-07 |
| rs138354 | circRNAs |  | Y | 4.56E-07 |
| rs1009532 | circRNAs |  | N | 4.57E-07 |
| rs11718509 | circRNAs,TADs | PBRM1 | Y | 4.57E-07 |
| rs485857 | circRNAs |  | N | 4.59E-07 |
| rs73406518 | circRNAs,TADs,CIRs | FSIP1,LRRC57,TP53BP1,RTF1,PLA2G4E | Y | 4.59E-07 |
| rs255353 | circRNAs |  | Y | 4.61E-07 |
| rs11670882 | TADs,CIRs,TFBRs | ZNF208,ATP13A1,CILP2,YJEFN3,NDUFA13,ZNF431,ENSG00000267383,ENSG00000258674,ENSG00000141979 | Y | 4.62E-07 |
| rs5758131 | circRNAs |  | Y | 4.63E-07 |
| rs10791849 | circRNAs,CIRs,lncRNAs | ENSG00000238752 | N | 4.64E-07 |
| rs7931544 | circRNAs |  | N | 4.65E-07 |
| rs4687637 | circRNAs,TADs | PBRM1 | Y | 4.65E-07 |
| rs10865974 | TADs | PBRM1 | Y | 4.69E-07 |
| rs10744559 | circRNAs |  | N | 4.69E-07 |
| rs6591207 | circRNAs |  | N | 4.71E-07 |
| rs527897 | circRNAs,CIRs | GAL3ST3 | Y | 4.72E-07 |
| rs12515751 | circRNAs |  | N | 4.73E-07 |
| rs174537 | circRNAs,TADs | RTN3 | Y | 4.74E-07 |
| rs10896085 | circRNAs |  | N | 4.77E-07 |
| rs2817378 | TADs | HCRTR2,PKHD1,PRIM2 | N | 4.82E-07 |
| rs9645684 | circRNAs |  | N | 4.83E-07 |
| rs2710314 | circRNAs |  | Y | 4.83E-07 |
| rs7544145 | CIRs | HIST2H2AA4,VPS45 | N | 4.84E-07 |
| rs10896091 | circRNAs |  | N | 4.84E-07 |
| rs11231677 | circRNAs,CIRs | FKBP2 | N | 4.85E-07 |
| rs6971577 | lncRNAs |  | Y | 4.86E-07 |
| rs2283287 | circRNAs |  | Y | 4.87E-07 |
| rs4687547 | circRNAs,TADs | PBRM1 | Y | 4.87E-07 |
| rs13082960 | circRNAs,TADs | NEK4 | Y | 4.87E-07 |
| rs10791853 | circRNAs |  | N | 4.88E-07 |
| rs4687548 | circRNAs,TADs | PBRM1,VPRBP | Y | 4.9E-07 |
| rs2238045 | circRNAs |  | N | 4.93E-07 |
| rs2535629 | circRNAs,TADs | VPRBP | Y | 4.93E-07 |
| rs7120326 | circRNAs |  | N | 4.94E-07 |
| rs57957974 | CIRs | ENSG00000255224 | Y | 4.98E-07 |
| rs174536 | circRNAs,TADs | RTN3 | Y | 5.01E-07 |
| rs11130325 | circRNAs,TADs | NEK4 | Y | 5.02E-07 |
| rs12063329 | CIRs,lncRNAs | VPS45 | Y | 5.03E-07 |
| rs10896099 | circRNAs |  | N | 5.04E-07 |
| rs2635253 | TADs | ENSG00000236922 | N | 5.06E-07 |
| rs6790330 | circRNAs,TADs | PBRM1 | Y | 5.07E-07 |
| rs12637627 | circRNAs,TADs | PBRM1 | Y | 5.07E-07 |
| rs918299 | circRNAs |  | N | 5.08E-07 |
| rs7115081 | circRNAs,CIRs | PACS1, | N | 5.09E-07 |
| rs7628578 | circRNAs,TADs | PBRM1 | Y | 5.09E-07 |
| rs12487591 | circRNAs,TADs,CIRs | PBRM1,VPRBP | Y | 5.09E-07 |
| rs6762457 | circRNAs,TADs | PBRM1 | Y | 5.14E-07 |
| rs12901215 | TADs | PATL2,GPR176,WDR76 | N | 5.15E-07 |
| rs7938376 | circRNAs |  | N | 5.17E-07 |
| rs11130317 | circRNAs |  | Y | 5.17E-07 |
| rs34476536 | circRNAs |  | N | 5.18E-07 |
| rs2710346 | circRNAs |  | Y | 5.18E-07 |
| rs6445531 | TADs | PBRM1 | Y | 5.19E-07 |
| rs3733046 | circRNAs,TADs | PBRM1 | Y | 5.2E-07 |
| rs13081031 | circRNAs,TADs | PBRM1,VPRBP | Y | 5.2E-07 |
| rs2283288 | circRNAs |  | Y | 5.21E-07 |
| rs34157897 | TADs | PBRM1 | Y | 5.21E-07 |
| rs61909095 | circRNAs |  | N | 5.21E-07 |
| rs11130323 | circRNAs,TADs | NEK4 | Y | 5.21E-07 |
| rs5756323 | circRNAs,TADs | CABIN1 | Y | 5.22E-07 |
| rs2268026 | circRNAs,TADs | NEK4 | Y | 5.23E-07 |
| rs3859514 | circRNAs |  | Y | 5.24E-07 |
| rs33967311 | circRNAs,TADs | PBRM1 | Y | 5.24E-07 |
| rs10896097 | circRNAs |  | N | 5.26E-07 |
| rs3794296 | circRNAs |  | N | 5.26E-07 |
| rs174529 | circRNAs,TADs,CIRs | MYRF,RTN3 | Y | 5.26E-07 |
| rs4435633 | circRNAs,TADs | PBRM1 | Y | 5.29E-07 |
| rs12563424 | CIRs | TMEM56-RWDD3 | N | 5.29E-07 |
| rs11633075 | CIRs,TFBRs | UBE2Q2P1,LINC00933 | Y | 5.3E-07 |
| rs7792447 | TADs | SSBP1,DENND2A | Y | 5.33E-07 |
| rs3755806 | circRNAs,TADs,CIRs | PBRM1,VPRBP | Y | 5.35E-07 |
| rs174591 | circRNAs |  | Y | 5.36E-07 |
| rs489337 | circRNAs,CIRs | PACS1, | N | 5.37E-07 |
| rs12061856 | CIRs,lncRNAs | VPS45,OTUD7B | Y | 5.37E-07 |
| rs13085775 | circRNAs,TADs | PBRM1,VPRBP | Y | 5.37E-07 |
| rs7625743 | circRNAs,TADs | PBRM1 | Y | 5.37E-07 |
| rs2164884 | circRNAs,TADs | PBRM1 | Y | 5.38E-07 |
| rs1987678 | circRNAs |  | N | 5.4E-07 |
| rs62253740 | circRNAs,TADs | PBRM1 | Y | 5.4E-07 |
| rs17138230 | circRNAs |  | Y | 5.42E-07 |
| rs112968809 | TADs,TFBRs | EPB42,CDAN1,ENSG00000246283 | N | 5.43E-07 |
| rs8068260 | TADs | ENSG00000174093,CDK12 | Y | 5.46E-07 |
| rs5749986 | circRNAs |  | Y | 5.48E-07 |
| rs10791858 | circRNAs |  | N | 5.48E-07 |
| rs4687648 | circRNAs,TADs | NEK4 | Y | 5.51E-07 |
| rs3852066 | circRNAs,TADs | PBRM1 | Y | 5.52E-07 |
| rs1055615 | circRNAs |  | N | 5.52E-07 |
| rs34954168 | circRNAs,TADs | PBRM1 | Y | 5.52E-07 |
| rs10791859 | circRNAs |  | N | 5.54E-07 |
| rs7942894 | circRNAs |  | N | 5.55E-07 |
| rs2336545 | circRNAs,TADs,TFBRs,lncRNAs | ENSG00000271137,NEK4 | Y | 5.56E-07 |
| rs1278516 | TADs | EPS15,AGBL4,USP24 | Y | 5.56E-07 |
| rs12226649 | circRNAs,CIRs | ENSG00000238752 | N | 5.56E-07 |
| rs7213683 | circRNAs |  | Y | 5.56E-07 |
| rs7941469 | circRNAs,CIRs | GAL3ST3 | N | 5.57E-07 |
| rs987335 | circRNAs |  | N | 5.58E-07 |
| rs2581827 | circRNAs |  | Y | 5.62E-07 |
| rs6445529 | circRNAs,TADs | PBRM1,VPRBP | Y | 5.62E-07 |
| rs10036712 | circRNAs |  | N | 5.62E-07 |
| rs12498066 | circRNAs,TADs | PBRM1 | Y | 5.64E-07 |
| rs13069481 | circRNAs,TADs | PBRM1,VPRBP | Y | 5.64E-07 |
| rs68021750 | circRNAs,TADs | PBRM1 | Y | 5.64E-07 |
| rs7622694 | circRNAs,TADs | PBRM1,VPRBP | Y | 5.64E-07 |
| rs61074241 | circRNAs |  | Y | 5.66E-07 |
| rs13085331 | circRNAs,TADs | PBRM1 | Y | 5.66E-07 |
| rs4687633 | circRNAs,TADs | PBRM1 | Y | 5.67E-07 |
| rs67539070 | circRNAs,TADs | PBRM1 | Y | 5.67E-07 |
| rs11130314 | circRNAs,TADs | PBRM1 | Y | 5.68E-07 |
| rs12279943 | circRNAs,TADs | TENM4,JRKL,JRKL-AS1,DLG2 | N | 5.68E-07 |
| rs6967214 | CIRs | MRPS33 | Y | 5.68E-07 |
| rs174535 | circRNAs,TADs | RTN3 | Y | 5.7E-07 |
| rs1108074 | circRNAs |  | N | 5.7E-07 |
| rs565198 | circRNAs |  | Y | 5.7E-07 |
| rs7916271 | TADs,TFBRs | KCNMA1,LIPA,NRG3,CYP2C18,CDHR1,HTR7,TSPAN14,DLG5,ENSG00000228566,TTC40,GRID1 | Y | 5.72E-07 |
| rs6869288 | circRNAs |  | N | 5.73E-07 |
| rs3774365 | circRNAs,TADs,CIRs | PBRM1,VPRBP | Y | 5.73E-07 |
| rs35755081 | TADs | DENND2A | N | 5.75E-07 |
| rs62253703 | circRNAs,TADs | PBRM1 | Y | 5.76E-07 |
| rs2336542 | circRNAs,TADs | PBRM1 | Y | 5.77E-07 |
| rs12124898 | CIRs | HIST2H2AB | Y | 5.78E-07 |
| rs547087 | circRNAs |  | N | 5.78E-07 |
| rs11227403 | circRNAs |  | N | 5.78E-07 |
| rs61614979 | circRNAs,TADs | SORCS1,SHOC2 | N | 5.81E-07 |
| rs4794221 | circRNAs,TADs | JUP,PCTP,FOXK2,CDC27,ENSG00000263317 | Y | 5.81E-07 |
| rs11130312 | circRNAs,TADs | PBRM1,VPRBP | Y | 5.81E-07 |
| rs1042779 | TADs | VPRBP | Y | 5.83E-07 |
| rs2175567 | CIRs,TFBRs | EGLN1P1,NMB | Y | 5.83E-07 |
| rs1508186 | TADs | IMMP2L | N | 5.84E-07 |
| rs12496634 | circRNAs,TADs | PBRM1 | Y | 5.84E-07 |
| rs11719685 | circRNAs,TADs | PBRM1,VPRBP | Y | 5.85E-07 |
| rs11130313 | circRNAs,TADs | PBRM1,VPRBP | Y | 5.85E-07 |
| rs35249778 | circRNAs,TADs | PBRM1,VPRBP | Y | 5.86E-07 |
| rs1819204 | circRNAs |  | Y | 5.89E-07 |
| rs11227415 | circRNAs |  | N | 5.89E-07 |
| rs13086297 | circRNAs,TADs | PBRM1,VPRBP | Y | 5.9E-07 |
| rs6805156 | circRNAs,TADs | PBRM1 | Y | 5.9E-07 |
| rs11714565 | circRNAs,TADs | PBRM1 | Y | 5.9E-07 |
| rs34215106 | circRNAs,TADs | PBRM1,VPRBP | Y | 5.91E-07 |
| rs56125600 | CIRs | HIST2H2BE,OTUD7B,BOLA1 | N | 5.92E-07 |
| rs1862005 | circRNAs |  | N | 5.92E-07 |
| rs11717043 | circRNAs,TADs | PBRM1,VPRBP | Y | 5.92E-07 |
| rs55918911 | CIRs | HIST2H2BE,BOLA1 | N | 5.92E-07 |
| rs13064064 | circRNAs,TADs | PBRM1,VPRBP | Y | 5.93E-07 |
| rs62255362 | circRNAs,TADs | PBRM1 | Y | 5.94E-07 |
| rs4687629 | circRNAs,TADs | PBRM1 | Y | 5.95E-07 |
| rs1860002 | circRNAs |  | N | 5.95E-07 |
| rs7642198 | circRNAs,TADs | PBRM1,VPRBP | Y | 5.95E-07 |
| rs12519539 | circRNAs |  | N | 5.96E-07 |
| rs13065019 | circRNAs,TADs | PBRM1 | Y | 5.96E-07 |
| rs34494381 | circRNAs |  | Y | 5.97E-07 |
| rs10750777 | circRNAs |  | N | 5.98E-07 |
| rs11614764 | circRNAs |  | N | 5.98E-07 |
| rs72694905 | circRNAs |  | N | 5.98E-07 |
| rs62129987 | circRNAs,CIRs | ILF3,ILF3-AS1 | Y | 5.99E-07 |
| rs6584962 | TADs,CIRs | ENSG00000223381,ENSG00000229981,SORCS1,CCDC147,XPNPEP1 | N | 5.99E-07 |
| rs6928 | circRNAs |  | Y | 6E-07 |
| rs2501575 | circRNAs |  | Y | 6E-07 |
| rs11227410 | circRNAs |  | N | 6.02E-07 |
| rs11130315 | circRNAs,TADs | PBRM1 | Y | 6.05E-07 |
| rs62255364 | circRNAs,TADs | PBRM1 | Y | 6.06E-07 |
| rs7083619 | circRNAs,TADs,TFBRs | SORCS1,SHOC2,ADD3 | N | 6.06E-07 |
| rs113704493 | TADs | EPS15,AGBL4,USP24 | N | 6.09E-07 |
| rs2238048 | circRNAs |  | N | 6.1E-07 |
| rs11194940 | circRNAs,TADs | SORCS1,SHOC2 | N | 6.1E-07 |
| rs11720243 | circRNAs,TADs | PBRM1 | Y | 6.11E-07 |
| rs2590846 | circRNAs,TADs | PBRM1 | Y | 6.11E-07 |
| rs7652191 | circRNAs,TADs | PBRM1 | Y | 6.14E-07 |
| rs535395 | circRNAs |  | N | 6.15E-07 |
| rs9630102 | circRNAs,TADs | SORCS1,SHOC2 | N | 6.18E-07 |
| rs2289250 | circRNAs,TADs | PBRM1 | Y | 6.19E-07 |
| rs524281 | circRNAs |  | Y | 6.2E-07 |
| rs13068293 | circRNAs,TADs | PBRM1,VPRBP | Y | 6.2E-07 |
| rs11585955 | circRNAs,CIRs | HIST2H2BE,HIST2H2AC,BOLA1 | N | 6.21E-07 |
| rs9611433 | circRNAs |  | Y | 6.22E-07 |
| rs11720159 | circRNAs,TADs | PBRM1 | Y | 6.22E-07 |
| rs72692897 | circRNAs |  | N | 6.24E-07 |
| rs6887473 | circRNAs |  | N | 6.24E-07 |
| rs11227414 | circRNAs |  | N | 6.25E-07 |
| rs7906904 | circRNAs,TADs,CIRs | ENSG00000203876,ENSG00000223381,ENSG00000229981,SORCS1,CCDC147 | Y | 6.26E-07 |
| rs7614498 | circRNAs,TADs | PBRM1 | Y | 6.26E-07 |
| rs7126075 | circRNAs |  | N | 6.26E-07 |
| rs2238057 | circRNAs |  | N | 6.27E-07 |
| rs11227413 | circRNAs |  | N | 6.32E-07 |
| rs11130310 | circRNAs,TADs | PBRM1,VPRBP | Y | 6.32E-07 |
| rs2501577 | circRNAs |  | N | 6.33E-07 |
| rs34757451 | circRNAs,TADs | PBRM1 | Y | 6.39E-07 |
| rs893363 | TADs,CIRs | RNU2-59P,CACNA2D3 | Y | 6.4E-07 |
| rs72692895 | circRNAs |  | N | 6.4E-07 |
| rs10075788 | TADs,lncRNAs | CAST,,ENSG00000251513,MCTP1 | N | 6.4E-07 |
| rs11583720 | circRNAs,CIRs | HIST2H2BE,HIST2H2AC,BOLA1 | N | 6.42E-07 |
| rs7935336 | circRNAs |  | N | 6.42E-07 |
| rs13086898 | circRNAs,TADs | PBRM1,VPRBP | Y | 6.42E-07 |
| rs6073595 | circRNAs,TADs | ENSG00000234948,STK4,BCAS4 | Y | 6.43E-07 |
| rs62002181 | TADs | LRFN5 | N | 6.43E-07 |
| rs11227411 | circRNAs,CIRs | ENSG00000255038 | N | 6.46E-07 |
| rs11130311 | circRNAs,TADs | PBRM1,VPRBP | Y | 6.46E-07 |
| rs10459232 | CIRs | ENSG00000258101 | Y | 6.47E-07 |
| rs77146033 | circRNAs,TADs | PBRM1 | Y | 6.47E-07 |
| rs34131103 | circRNAs,TADs | ENSG00000234948,STK4,BCAS4 | Y | 6.48E-07 |
| rs10896080 | circRNAs |  | N | 6.48E-07 |
| rs72896994 | TADs | EPS15,AGBL4,USP24 | N | 6.49E-07 |
| rs6504703 | circRNAs,TADs | JUP,PCTP,FOXK2,CDC27,ENSG00000263317 | Y | 6.56E-07 |
| rs10875918 | CIRs | RHEBL1 | Y | 6.57E-07 |
| rs10777956 | circRNAs |  | N | 6.58E-07 |
| rs11714248 | CIRs | MLH1 | Y | 6.58E-07 |
| rs12063281 | CIRs,lncRNAs | VPS45,OTUD7B | N | 6.59E-07 |
| rs11237804 | circRNAs |  | N | 6.59E-07 |
| rs7266289 | circRNAs |  | Y | 6.6E-07 |
| rs549187 | circRNAs |  | Y | 6.61E-07 |
| rs11227409 | circRNAs |  | Y | 6.61E-07 |
| rs1561337 | circRNAs,TADs | PBRM1,VPRBP | Y | 6.62E-07 |
| rs11607393 | circRNAs |  | N | 6.62E-07 |
| rs1906252 | circRNAs,TADs | FAXC,ENSG00000271860 | Y | 6.62E-07 |
| rs6065773 | circRNAs |  | Y | 6.63E-07 |
| rs72692901 | circRNAs |  | N | 6.63E-07 |
| rs2175420 | circRNAs,TADs | TENM4,JRKL,JRKL-AS1,DLG2 | N | 6.66E-07 |
| rs10888569 | CIRs | HIST2H2AB,HIST2H2BE,BOLA1,ENSG00000223945 | N | 6.66E-07 |
| rs174533 | circRNAs,TADs | RTN3 | Y | 6.67E-07 |
| rs72692896 | circRNAs |  | N | 6.67E-07 |
| rs3825067 | circRNAs,CIRs | ENSG00000255038 | N | 6.67E-07 |
| rs4687546 | circRNAs,TADs | PBRM1 | Y | 6.69E-07 |
| rs13043430 | circRNAs,TADs | ENSG00000234948,STK4,BCAS4 | Y | 6.7E-07 |
| rs3733045 | circRNAs,TADs,CIRs | PBRM1,VPRBP | Y | 6.7E-07 |
| rs55840906 | CIRs,lncRNAs | VPS45 | N | 6.71E-07 |
| rs72828245 | circRNAs,TADs | SORCS1,SHOC2 | N | 6.74E-07 |
| rs68119487 | circRNAs |  | N | 6.74E-07 |
| rs8080145 | circRNAs,TADs | CA10,PLEKHM1,LRRC37A3 | Y | 6.75E-07 |
| rs12061167 | CIRs | VPS45,OTUD7B | N | 6.75E-07 |
| rs10896081 | circRNAs |  | N | 6.76E-07 |
| rs6504704 | circRNAs,TADs | JUP,PCTP,FOXK2,CDC27,ENSG00000263317 | Y | 6.76E-07 |
| rs17420378 | circRNAs,TADs | ENSG00000234948,STK4,BCAS4 | Y | 6.78E-07 |
| rs28404639 | circRNAs |  | N | 6.79E-07 |
| rs17106175 | TADs | EPS15,AGBL4,USP24 | N | 6.8E-07 |
| rs2257216 | circRNAs |  | Y | 6.8E-07 |
| rs7268941 | circRNAs |  | Y | 6.83E-07 |
| rs35140738 | circRNAs |  | Y | 6.85E-07 |
| rs7915021 | circRNAs,TADs | RSU1 | N | 6.86E-07 |
| rs78286375 | circRNAs |  | N | 6.87E-07 |
| rs10403579 | circRNAs,TADs | ZNF208,ENSG00000267383,ENSG00000141979 | N | 6.88E-07 |
| rs34168767 | circRNAs,TADs | PBRM1 | Y | 6.88E-07 |
| rs13041360 | circRNAs |  | Y | 6.89E-07 |
| rs12263484 | TADs | ENSG00000223381,ENSG00000229981,SORCS1,CCDC147 | Y | 6.9E-07 |
| rs6073615 | circRNAs |  | Y | 6.9E-07 |
| rs240068 | circRNAs |  | Y | 6.91E-07 |
| rs2143610 | circRNAs |  | Y | 6.95E-07 |
| rs4850478 | TADs | STAT4 | N | 6.96E-07 |
| rs12275848 | circRNAs,TADs | TENM4,JRKL,JRKL-AS1,DLG2 | N | 6.98E-07 |
| rs185308 | circRNAs,TFBRs | ENSG00000249175 | Y | 7E-07 |
| rs4950077 | CIRs | TMEM56-RWDD3 | N | 7.01E-07 |
| rs12576775 | circRNAs |  | Y | 7.02E-07 |
| rs174530 | circRNAs,TADs | RTN3 | Y | 7.04E-07 |
| rs2027349 | CIRs,TFBRs | HIST2H2AA3,OSER1,SNORD101,SSBP1,VPS45,FARS2,LYRM4,CDC42,OSER1-AS1,OTUD7B,RPS12,CNIH1,WEE2-AS1 | Y | 7.06E-07 |
| rs12697705 | circRNAs |  | N | 7.06E-07 |
| rs571434 | circRNAs |  | N | 7.09E-07 |
| rs13060675 | circRNAs,TADs | PBRM1,VPRBP | Y | 7.1E-07 |
| rs11718420 | circRNAs,TADs | PBRM1 | Y | 7.12E-07 |
| rs1964542 | CIRs,TFBRs | HIST2H2AB,HIST2H2BE,BOLA1,ENSG00000223945 | Y | 7.13E-07 |
| rs113583672 | CIRs | LRRC57 | N | 7.13E-07 |
| rs7520930 | CIRs,TFBRs,lncRNAs | VPS45 | Y | 7.14E-07 |
| rs72692857 | CIRs,lncRNAs | VPS45,OTUD7B | N | 7.17E-07 |
| rs10042201 | circRNAs |  | N | 7.19E-07 |
| rs9911355 | circRNAs,TADs | CA10,PLEKHM1,LRRC37A3 | Y | 7.21E-07 |
| rs12061565 | CIRs | HIST2H2BE,BOLA1 | N | 7.23E-07 |
| rs4926412 | CIRs | VPS45 | N | 7.27E-07 |
| rs72694906 | circRNAs |  | N | 7.3E-07 |
| rs1339495 | CIRs | HIST2H2BE,VPS45,OTUD7B,BOLA1 | N | 7.3E-07 |
| rs28360639 | TADs | HCRTR2,PKHD1,PRIM2 | N | 7.3E-07 |
| rs2169895 | CIRs | HIST2H2BE,VPS45,BOLA1 | N | 7.35E-07 |
| rs6803012 | circRNAs,TADs | PBRM1 | Y | 7.36E-07 |
| rs192793035 | CIRs | FEN1 | Y | 7.36E-07 |
| rs72694908 | circRNAs,CIRs | HIST2H2AC | N | 7.37E-07 |
| rs13041216 | circRNAs |  | Y | 7.37E-07 |
| rs6591223 | circRNAs,CIRs,TFBRs | RBM4B,RCE1,PC | Y | 7.42E-07 |
| rs2060675 | circRNAs |  | Y | 7.42E-07 |
| rs56188926 | circRNAs |  | N | 7.43E-07 |
| rs17138318 | circRNAs,TADs | TENM4,JRKL,JRKL-AS1,DLG2 | N | 7.44E-07 |
| rs2878632 | circRNAs,TADs | PBRM1 | Y | 7.45E-07 |
| rs7068482 | circRNAs,TADs | SORCS1,SHOC2 | N | 7.46E-07 |
| rs62255396 | circRNAs,TADs | NEK4 | Y | 7.46E-07 |
| rs9915099 | circRNAs |  | Y | 7.47E-07 |
| rs7921609 | TADs | ENSG00000223381,ENSG00000229981,SORCS1,CCDC147 | N | 7.47E-07 |
| rs12049852 | circRNAs |  | N | 7.47E-07 |
| rs78308718 | circRNAs |  | N | 7.47E-07 |
| rs4665972 | circRNAs |  | N | 7.5E-07 |
| rs9879090 | circRNAs,TADs | PBRM1,VPRBP | Y | 7.5E-07 |
| rs1339494 | CIRs,lncRNAs | HIST2H2BE,VPS45,OTUD7B,BOLA1 | N | 7.52E-07 |
| rs7720123 | circRNAs |  | N | 7.53E-07 |
| rs2276824 | circRNAs,TADs | PBRM1 | Y | 7.54E-07 |
| rs5755099 | circRNAs |  | Y | 7.56E-07 |
| rs11130319 | CIRs | GLT8D1 | Y | 7.58E-07 |
| rs2318788 | circRNAs,TADs | CA10,PLEKHM1,LRRC37A3 | Y | 7.58E-07 |
| rs34870295 | circRNAs |  | N | 7.63E-07 |
| rs72828247 | circRNAs,TADs | SORCS1,SHOC2 | Y | 7.66E-07 |
| rs4950083 | CIRs,TFBRs | TMEM56,ENSG00000231992,TMEM56-RWDD3,RWDD3 | N | 7.68E-07 |
| rs11237805 | circRNAs |  | N | 7.69E-07 |
| rs4448992 | circRNAs,TADs | CA10,PLEKHM1,LRRC37A3 | Y | 7.7E-07 |
| rs3862386 | circRNAs |  | N | 7.71E-07 |
| rs4926411 | CIRs | VPS45 | N | 7.72E-07 |
| rs1571617 | circRNAs,TADs | SORCS1,SHOC2 | N | 7.74E-07 |
| rs2238046 | circRNAs |  | N | 7.74E-07 |
| rs187084 | circRNAs |  | Y | 7.74E-07 |
| rs3786007 | circRNAs,TADs | CA10,PLEKHM1,LRRC37A3 | Y | 7.77E-07 |
| rs16966448 | circRNAs |  | N | 7.78E-07 |
| rs11589801 | CIRs | HIST2H2AA4 | N | 7.78E-07 |
| rs12060211 | CIRs | HIST2H2BE,BOLA1 | N | 7.79E-07 |
| rs7550350 | CIRs,lncRNAs | VPS45 | N | 7.82E-07 |
| rs34864931 | circRNAs |  | Y | 7.82E-07 |
| rs1053007 | circRNAs,CIRs | ILF3,ILF3-AS1 | Y | 7.83E-07 |
| rs8136590 | circRNAs,CIRs | RNA5SP493 | Y | 7.84E-07 |
| rs11194926 | TADs | ENSG00000223381,ENSG00000229981,SORCS1,CCDC147 | Y | 7.84E-07 |
| rs2564930 | circRNAs |  | Y | 7.84E-07 |
| rs12076604 | TADs | EPS15,AGBL4,USP24 | Y | 7.85E-07 |
| rs10735362 | circRNAs |  | Y | 7.86E-07 |
| rs73404791 | circRNAs |  | Y | 7.86E-07 |
| rs4794820 | TADs,CIRs | ENSG00000174093,FBXL20,CDK12,ORMDL3,ENSG00000264968 | Y | 7.87E-07 |
| rs488320 | circRNAs |  | N | 7.89E-07 |
| rs2006893 | circRNAs |  | Y | 7.89E-07 |
| rs55633125 | CIRs | HIST2H2BE,OTUD7B,BOLA1 | N | 7.9E-07 |
| rs8078958 | circRNAs,TADs | JUP,PCTP,FOXK2,CDC27,ENSG00000263317 | Y | 7.91E-07 |
| rs10896077 | circRNAs |  | N | 7.92E-07 |
| rs12093499 | CIRs | HIST2H2BE,BOLA1 | N | 7.94E-07 |
| rs1397721 | circRNAs,TADs,CIRs | TENM4,MIR5579,JRKL,JRKL-AS1,DLG2 | N | 7.94E-07 |
| rs2283794 | circRNAs |  | Y | 7.96E-07 |
| rs61545625 | CIRs | EXOSC4 | Y | 7.99E-07 |
| rs7086057 | circRNAs |  | N | 8.01E-07 |
| rs8076189 | circRNAs,TADs,CIRs | MBTD1,CA10,PLEKHM1,LRRC37A3 | Y | 8.03E-07 |
| rs4245670 | CIRs,TFBRs | TMEM56,TMEM56-RWDD3,ALG14 | N | 8.05E-07 |
| rs4058847 | circRNAs |  | Y | 8.08E-07 |
| rs13085895 | circRNAs,TADs | PBRM1,VPRBP | Y | 8.09E-07 |
| rs1892848 | circRNAs |  | Y | 8.11E-07 |
| rs4687639 | circRNAs,TADs | PBRM1,VPRBP | Y | 8.11E-07 |
| rs12074281 | CIRs | HIST2H2AB,HIST2H2BE,BOLA1,ENSG00000223945 | N | 8.13E-07 |
| rs6073616 | circRNAs,CIRs | STK4-AS1 | Y | 8.15E-07 |
| rs4344373 | CIRs,TFBRs | TMEM56,TMEM56-RWDD3,RWDD3,ALG14 | N | 8.16E-07 |
| rs7271519 | circRNAs,TADs | ENSG00000234948,STK4,BCAS4 | Y | 8.16E-07 |
| rs2245424 | circRNAs |  | Y | 8.17E-07 |
| rs10848632 | circRNAs |  | Y | 8.18E-07 |
| rs67712855 | circRNAs |  | N | 8.2E-07 |
| rs917654 | circRNAs,TADs | JUP,PCTP,FOXK2,CDC27,ENSG00000263317 | Y | 8.2E-07 |
| rs2237311 | circRNAs |  | Y | 8.21E-07 |
| rs12058502 | CIRs | HIST2H2BE,BOLA1 | N | 8.21E-07 |
| rs12058090 | CIRs | HIST2H2BE,BOLA1 | N | 8.23E-07 |
| rs45605540 | circRNAs |  | N | 8.24E-07 |
| rs35545554 | circRNAs |  | Y | 8.25E-07 |
| rs113607766 | CIRs,TFBRs | HIST2H2AB,HIST2H2BE,VPS45,BOLA1,ENSG00000223945 | N | 8.26E-07 |
| rs2237306 | circRNAs |  | Y | 8.27E-07 |
| rs1511240 | circRNAs |  | N | 8.28E-07 |
| rs9926049 | circRNAs |  | N | 8.28E-07 |
| rs4687630 | circRNAs,TADs | PBRM1 | Y | 8.28E-07 |
| rs118089279 | CIRs | KMT2E,RINT1,ENSG00000232104,RFX3,LINC01004 | N | 8.29E-07 |
| rs7540847 | CIRs | VPS45 | N | 8.3E-07 |
| rs12065681 | CIRs | VPS45,OTUD7B | N | 8.31E-07 |
| rs34359922 | circRNAs,miRNA target site | LRRC57 | Y | 8.32E-07 |
| rs12063059 | CIRs | OTUD7B | N | 8.32E-07 |
| rs9607287 | circRNAs |  | Y | 8.32E-07 |
| rs2635252 | TADs | ENSG00000236922 | N | 8.35E-07 |
| rs7183893 | TADs | PATL2,GPR176,WDR76 | N | 8.37E-07 |
| rs5749998 | circRNAs,CIRs | MAPK1 | Y | 8.4E-07 |
| rs4850477 | TADs | STAT4 | N | 8.41E-07 |
| rs2710313 | circRNAs |  | Y | 8.43E-07 |
| rs5999521 | circRNAs,CIRs,TFBRs | RNA5SP493 | Y | 8.43E-07 |
| rs2007044 | circRNAs |  | Y | 8.44E-07 |
| rs72828224 | TADs | ENSG00000223381,ENSG00000229981,SORCS1,CCDC147 | N | 8.45E-07 |
| rs12479532 | TADs,CIRs,TFBRs | TAF4,EEF1A2,ESRP1,ENSG00000261437,DIDO1 | N | 8.49E-07 |
| rs11205326 | circRNAs |  | N | 8.51E-07 |
| rs7222039 | TADs | ENSG00000174093,CDK12 | Y | 8.52E-07 |
| rs6017463 | circRNAs |  | Y | 8.53E-07 |
| rs10749674 | circRNAs |  | N | 8.54E-07 |
| rs4515041 | circRNAs |  | Y | 8.56E-07 |
| rs2710326 | circRNAs |  | Y | 8.57E-07 |
| rs72692900 | circRNAs |  | N | 8.58E-07 |
| rs72692893 | circRNAs |  | N | 8.58E-07 |
| rs6065778 | circRNAs,CIRs | STK4-AS1 | Y | 8.58E-07 |
| rs11584354 | CIRs,TFBRs,lncRNAs | FOS,HIST2H2AA3,FARS2,VPS45,LYRM4,OTUD7B,HNRNPAB | N | 8.58E-07 |
| rs2635254 | TADs | ENSG00000236922 | N | 8.59E-07 |
| rs5750113 | circRNAs |  | Y | 8.59E-07 |
| rs72692898 | circRNAs |  | N | 8.59E-07 |
| rs111465664 | circRNAs |  | N | 8.59E-07 |
| rs72692899 | circRNAs |  | N | 8.6E-07 |
| rs13943 | circRNAs |  | Y | 8.6E-07 |
| rs112287706 | circRNAs |  | N | 8.6E-07 |
| rs3810155 | CIRs,TFBRs | PLAG1,IER3,AP1M2,AGFG1,FLOT1,ENSG00000272273,ILF3,QTRT1,CHCHD7,XRCC6BP1,COQ5,RCCD1,MIR5703,RNU2-59P,NUTF2,DOLPP1,GADD45B,DCUN1D4,ILF3-AS1,ENSG00000258384,RERE,ENSG00000263105,MIR638,TUBB4B | Y | 8.61E-07 |
| rs56212907 | circRNAs |  | N | 8.63E-07 |
| rs12914433 | lncRNAs |  | Y | 8.63E-07 |
| rs2635242 | TADs | ENSG00000236922 | N | 8.63E-07 |
| rs7263949 | circRNAs |  | Y | 8.64E-07 |
| rs6073618 | circRNAs |  | Y | 8.67E-07 |
| rs12060027 | CIRs | VPS45 | N | 8.67E-07 |
| rs6866154 | circRNAs |  | N | 8.68E-07 |
| rs3810609 | circRNAs |  | Y | 8.7E-07 |
| rs2710339 | circRNAs |  | Y | 8.7E-07 |
| rs2336670 | circRNAs |  | Y | 8.7E-07 |
| rs1019195 | circRNAs,TADs | CA10,PLEKHM1,LRRC37A3 | Y | 8.71E-07 |
| rs62368675 | circRNAs |  | N | 8.73E-07 |
| rs472518 | circRNAs |  | Y | 8.77E-07 |
| rs34654732 | circRNAs,TADs | ENSG00000234948,STK4,BCAS4 | Y | 8.82E-07 |
| rs9901084 | circRNAs,TADs | CA10,PLEKHM1,LRRC37A3 | Y | 8.84E-07 |
| rs2239018 | circRNAs |  | N | 8.86E-07 |
| rs7133502 | circRNAs |  | N | 8.9E-07 |
| rs28660389 | CIRs | OTUD7B | N | 8.91E-07 |
| rs3733039 | TADs,CIRs,TFBRs | MALAT1,ENSG00000270117,PBRM1 | Y | 8.91E-07 |
| rs7250942 | circRNAs |  | Y | 8.95E-07 |
| rs4980532 | circRNAs |  | N | 9.02E-07 |
| rs9607347 | CIRs | PPM1F | Y | 9.03E-07 |
| rs6414570 | circRNAs,TADs | NEK4 | Y | 9.06E-07 |
| rs1278522 | TADs | EPS15,AGBL4,USP24 | N | 9.06E-07 |
| rs10120508 | TADs | CDK5RAP2 | Y | 9.08E-07 |
| rs2668592 | circRNAs |  | Y | 9.08E-07 |
| rs2191443 | circRNAs,TADs,lncRNAs | CA10,PLEKHM1,LRRC37A3 | Y | 9.1E-07 |
| rs4915698 | circRNAs |  | Y | 9.15E-07 |
| rs1824848 | CIRs | HIST2H2AA4,VPS45 | N | 9.15E-07 |
| rs12061196 | CIRs | VPS45,OTUD7B | N | 9.19E-07 |
| rs2336672 | circRNAs |  | Y | 9.2E-07 |
| rs2302776 | circRNAs,TADs,CIRs | ENSG00000174093,CDK12,PSMD3 | Y | 9.22E-07 |
| rs112541899 | CIRs | OTUD7B | N | 9.23E-07 |
| rs9904536 | circRNAs |  | Y | 9.23E-07 |
| rs4516540 | circRNAs |  | Y | 9.24E-07 |
| rs11589585 | CIRs | HIST2H2BE,BOLA1 | N | 9.24E-07 |
| rs6694729 | circRNAs,CIRs | HIST2H2AB | N | 9.25E-07 |
| rs61156785 | CIRs | KIAA1875 | Y | 9.27E-07 |
| rs7553622 | circRNAs |  | N | 9.29E-07 |
| rs11205321 | CIRs,TFBRs | HIST2H2AB,HIST2H2BE,VPS45,BOLA1,ENSG00000223945 | Y | 9.29E-07 |
| rs61624875 | circRNAs,TADs | SORCS1,SHOC2 | N | 9.31E-07 |
| rs3850838 | circRNAs |  | N | 9.31E-07 |
| rs71339819 | circRNAs |  | N | 9.33E-07 |
| rs6518986 | circRNAs |  | Y | 9.34E-07 |
| rs7970642 | circRNAs |  | N | 9.34E-07 |
| rs10415278 | circRNAs,TADs,CIRs | ZNF208,GMIP,ENSG00000267383,ENSG00000141979 | Y | 9.35E-07 |
| rs2229383 | circRNAs,CIRs | ILF3 | Y | 9.35E-07 |
| rs7582249 | circRNAs |  | Y | 9.37E-07 |
| rs61844069 | circRNAs,TADs | RSU1 | N | 9.38E-07 |
| rs2372720 | circRNAs |  | N | 9.39E-07 |
| rs72694919 | circRNAs |  | N | 9.39E-07 |
| rs71604175 | circRNAs |  | Y | 9.39E-07 |
| rs72694917 | circRNAs |  | N | 9.4E-07 |
| rs72694943 | CIRs | PLEKHO1 | N | 9.4E-07 |
| rs4604890 | circRNAs,CIRs | ENSG00000238752 | N | 9.41E-07 |
| rs80157846 | circRNAs |  | Y | 9.41E-07 |
| rs72694918 | circRNAs |  | N | 9.41E-07 |
| rs3810610 | circRNAs |  | Y | 9.42E-07 |
| rs4804514 | CIRs,TFBRs,lncRNAs | ENSG00000268403,ENSG00000264666,RPS4XP16,ILF3,KEAP1,QTRT1,ZNF143,COQ5,INTS6-AS1,METTL23,TMED2,JMJD6,ENSG00000247373,FBXO28,C19orf38,PEMT,GADD45B,ILF3-AS1,MIR638 | Y | 9.43E-07 |
| rs11704205 | circRNAs,CIRs,TFBRs | RNA5SP493 | Y | 9.43E-07 |
| rs72748702 | TADs | ENSG00000259986,ENSG00000166503,ADAMTS7P4 | Y | 9.43E-07 |
| rs57440982 | circRNAs,TADs | SORCS1,SHOC2 | N | 9.43E-07 |
| rs2283792 | circRNAs,CIRs | ENSG00000224086 | Y | 9.46E-07 |
| rs7488242 | circRNAs |  | N | 9.47E-07 |
| rs12240333 | circRNAs,TADs | SORCS1,SHOC2 | N | 9.53E-07 |
| rs2276834 | CIRs | WDR82,PHF7 | Y | 9.56E-07 |
| rs5999550 | circRNAs,CIRs | RNA5SP493 | Y | 9.56E-07 |
| rs12263915 | circRNAs,TADs | SORCS1,SHOC2 | N | 9.56E-07 |
| rs35626751 | circRNAs |  | Y | 9.56E-07 |
| rs6060023 | circRNAs |  | Y | 9.57E-07 |
| rs6073623 | circRNAs |  | Y | 9.57E-07 |
| rs11194941 | circRNAs,TADs | SORCS1,SHOC2 | N | 9.58E-07 |
| rs2266967 | circRNAs |  | Y | 9.59E-07 |
| rs61884064 | circRNAs,TADs | TENM4,JRKL,JRKL-AS1,DLG2 | N | 9.62E-07 |
| rs6073621 | circRNAs |  | Y | 9.62E-07 |
| rs7622851 | CIRs,TFBRs | GLYCTK-AS1,MIR135A1,WDR82 | Y | 9.62E-07 |
| rs55897175 | TADs | ENSG00000223381,ENSG00000229981,SORCS1,CCDC147 | N | 9.64E-07 |
| rs72694927 | circRNAs |  | N | 9.66E-07 |
| rs975442 | TADs,lncRNAs | ENSG00000223381,ENSG00000229981,SORCS1,CCDC147 | Y | 9.67E-07 |
| rs7653028 | circRNAs,TADs | NEK4 | Y | 9.7E-07 |
| rs113664928 | circRNAs |  | N | 9.73E-07 |
| rs1318597 | circRNAs |  | N | 9.74E-07 |
| rs2238047 | circRNAs |  | N | 9.75E-07 |
| rs7550323 | CIRs | VPS45 | N | 9.78E-07 |
| rs7519918 | CIRs | HIST2H2BE,OTUD7B,BOLA1 | N | 9.8E-07 |
| rs6796726 | circRNAs,TADs | PBRM1,VPRBP | Y | 9.8E-07 |
| rs10933 | TADs,CIRs,TFBRs | KMT2E,EIF4A1,MGEA5,PBRM1,SNORA67,SPCS1,KCNIP2-AS1,TM9SF3,LINC01004 | N | 9.8E-07 |
| rs12621381 | TADs | DPP10 | N | 9.8E-07 |
| rs4821402 | circRNAs,CIRs | YPEL1 | Y | 9.81E-07 |
| rs7199910 | circRNAs |  | N | 9.83E-07 |
| rs175382 | TADs | JUP,PCTP,FOXK2,CDC27,ENSG00000263317 | Y | 9.88E-07 |
| rs58404036 | CIRs,lncRNAs | ENSG00000255224 | Y | 9.89E-07 |
| rs7979741 | circRNAs |  | N | 9.91E-07 |
| rs4924683 | CIRs | LRRC57,SNAP23 | Y | 9.91E-07 |
| rs111298861 | circRNAs |  | N | 9.94E-07 |
| rs8076318 | circRNAs,TADs | CA10,PLEKHM1,LRRC37A3 | Y | 9.95E-07 |
| rs35586490 | circRNAs |  | N | 9.98E-07 |
| rs114534140 | circRNAs |  | N | 0.000001 |
| rs7555080 | circRNAs |  | N | 1E-06 |
| rs1406579 | TADs | STAT4 | N | 1E-06 |
| rs2238049 | circRNAs |  | N | 1.01E-06 |
| rs11205317 | CIRs | VPS45,OTUD7B | N | 1.01E-06 |
| rs112521467 | circRNAs |  | N | 1.01E-06 |
| rs111397987 | circRNAs |  | N | 1.01E-06 |
| rs112245786 | circRNAs |  | N | 1.01E-06 |
| rs113316220 | circRNAs |  | N | 1.01E-06 |
| rs9311474 | circRNAs |  | Y | 1.01E-06 |
| rs17095350 | TADs | ENSG00000223381,ENSG00000229981,SORCS1,CCDC147 | N | 1.01E-06 |
| rs12254785 | TADs | ENSG00000223381,ENSG00000229981,SORCS1,CCDC147 | N | 1.01E-06 |
| rs2270448 | circRNAs,TFBRs | PACS1 | Y | 1.01E-06 |
| rs112814713 | circRNAs |  | N | 1.01E-06 |
| rs72694915 | circRNAs |  | N | 1.01E-06 |
| rs72694914 | circRNAs |  | N | 1.01E-06 |
| rs12098106 | CIRs | VPS45,OTUD7B | N | 1.01E-06 |
| rs11717836 | TADs | VPRBP | Y | 1.02E-06 |
| rs72694923 | circRNAs |  | N | 1.02E-06 |
| rs1919635 | TADs | STAT1 | N | 1.02E-06 |
| rs59042914 | circRNAs,TADs | SORCS1,SHOC2 | N | 1.02E-06 |
| rs76347963 | TADs,TFBRs | ZNF208,ENSG00000267383,ENSG00000141979,ZNF101 | N | 1.02E-06 |
| rs174575 | circRNAs,CIRs | NAP1L4 | Y | 1.02E-06 |
| rs1821879 | circRNAs,TADs | PBRM1 | Y | 1.02E-06 |
| rs72692902 | circRNAs |  | N | 1.03E-06 |
| rs12244557 | circRNAs,TADs | SORCS1,SHOC2 | N | 1.03E-06 |
| rs10848635 | circRNAs |  | Y | 1.03E-06 |
| rs8081088 | CIRs | MBTD1 | Y | 1.03E-06 |
| rs1023330 | CIRs | ENSG00000235501 | Y | 1.03E-06 |
| rs6073622 | circRNAs |  | Y | 1.03E-06 |
| rs2118540 | circRNAs,TADs | PBRM1 | Y | 1.03E-06 |
| rs8065491 | TADs | PCTP | N | 1.03E-06 |
| rs1892846 | circRNAs |  | Y | 1.03E-06 |
| rs1961958 | circRNAs,TADs | CADPS,FHIT,CACNA2D3,CACNA2D2 | Y | 1.03E-06 |
| rs35828350 | CIRs | ALPK3 | Y | 1.03E-06 |
| rs7127808 | circRNAs |  | N | 1.04E-06 |
| rs17126890 | TADs | ENSG00000223381,ENSG00000229981,SORCS1,CCDC147 | N | 1.04E-06 |
| rs11085743 | CIRs | AP1M2 | Y | 1.04E-06 |
| rs72694944 | CIRs | PLEKHO1 | N | 1.04E-06 |
| rs7079713 | circRNAs,TADs,TFBRs | SORCS1,SHOC2,ADD3 | Y | 1.05E-06 |
| rs12620822 | TADs | STAT4 | N | 1.05E-06 |
| rs60095211 | TADs,CIRs | FER1L4,NCOA6,ASIP | Y | 1.05E-06 |
| rs7250071 | CIRs,TFBRs | AP1M2,USE1,YIPF2,ILF3,QTRT1,GADD45B,COQ5,ILF3-AS1,MIR638 | N | 1.05E-06 |
| rs6682989 | TADs | RAB3B,AGBL4,LRP8,FAF1 | N | 1.05E-06 |
| rs62026530 | CIRs | ENSG00000254414 | Y | 1.05E-06 |
| rs28560140 | CIRs,lncRNAs | OTUD7B | N | 1.06E-06 |
| rs10848631 | circRNAs |  | N | 1.06E-06 |
| rs34748157 | circRNAs,TADs,CIRs | ENSG00000234948,STK4-AS1,STK4,BCAS4 | Y | 1.06E-06 |
| rs55909733 | CIRs | PLEKHO1 | N | 1.06E-06 |
| rs6725371 | TADs | STAT1 | N | 1.06E-06 |
| rs6870325 | circRNAs |  | N | 1.06E-06 |
| rs12066132 | CIRs | VPS45 | N | 1.06E-06 |
| rs72692865 | CIRs | VPS45 | N | 1.06E-06 |
| rs255373 | circRNAs |  | Y | 1.06E-06 |
| rs12067422 | CIRs | VPS45 | N | 1.07E-06 |
| rs5999515 | circRNAs,CIRs,TFBRs | RNA5SP493 | Y | 1.07E-06 |
| rs2419313 | circRNAs,TADs | SORCS1,SHOC2 | N | 1.07E-06 |
| rs12066293 | CIRs | VPS45 | N | 1.07E-06 |
| rs174546 | circRNAs,CIRs | MIR1908 | Y | 1.07E-06 |
| rs13158214 | circRNAs |  | N | 1.07E-06 |
| rs1278518 | TADs | EPS15,AGBL4,USP24 | N | 1.07E-06 |
| rs4327046 | circRNAs |  | N | 1.08E-06 |
| rs7978433 | circRNAs |  | N | 1.08E-06 |
| rs72898937 | circRNAs |  | N | 1.08E-06 |
| rs6513959 | circRNAs,TADs | ENSG00000234948,STK4,BCAS4 | Y | 1.08E-06 |
| rs7918659 | CIRs | NIFKP9 | N | 1.08E-06 |
| rs7575482 | TADs | STAT1 | N | 1.08E-06 |
| rs17766532 | circRNAs |  | N | 1.08E-06 |
| rs4924687 | circRNAs,TADs | FSIP1,TP53BP1,RTF1,PLA2G4E | Y | 1.08E-06 |
| rs11636189 | circRNAs |  | Y | 1.08E-06 |
| rs3925724 | circRNAs,CIRs | HIST2H2AB | Y | 1.08E-06 |
| rs11205319 | lncRNAs |  | N | 1.08E-06 |
| rs6495988 | TADs | PATL2,GPR176,WDR76 | N | 1.08E-06 |
| rs2033284 | TADs | SH3GL3,ENSG00000259986,ENSG00000166503,ADAMTS7P4 | Y | 1.08E-06 |
| rs4926391 | CIRs | HIST2H2AA4 | N | 1.09E-06 |
| rs28753817 | CIRs,lncRNAs | OTUD7B | N | 1.09E-06 |
| rs13377169 | circRNAs,TADs | SORCS1,SHOC2 | N | 1.09E-06 |
| rs133082 | lncRNAs |  | Y | 1.09E-06 |
| rs35775745 | circRNAs |  | N | 1.09E-06 |
| rs7931485 | circRNAs |  | Y | 1.09E-06 |
| rs9662055 | CIRs | OTUD7B | N | 1.09E-06 |
| rs10860392 | circRNAs |  | Y | 1.09E-06 |
| rs6073591 | circRNAs,TADs | ENSG00000234948,STK4,BCAS4 | Y | 1.1E-06 |
| rs13037444 | circRNAs,TADs | ENSG00000234948,STK4,BCAS4 | Y | 1.1E-06 |
| rs34550586 | circRNAs,CIRs,TFBRs | RNA5SP493 | Y | 1.1E-06 |
| rs734163 | TADs | ENSG00000223381,ENSG00000229981,SORCS1,CCDC147 | Y | 1.11E-06 |
| rs62021208 | circRNAs,CIRs | ZNF592 | Y | 1.11E-06 |
| rs8072548 | TADs | ENSG00000174093,CDK12 | Y | 1.11E-06 |
| rs35516100 | circRNAs,CIRs | ZNF592 | Y | 1.11E-06 |
| rs5995983 | CIRs | RBX1 | Y | 1.11E-06 |
| rs2319280 | CIRs | VPS45,OTUD7B | N | 1.11E-06 |
| rs6073582 | circRNAs,TADs | ENSG00000234948,STK4,BCAS4 | Y | 1.11E-06 |
| rs12899449 | TADs | PATL2,GPR176,WDR76 | Y | 1.12E-06 |
| rs72692869 | CIRs | OTUD7B | N | 1.12E-06 |
| rs72694926 | circRNAs,CIRs | HIST2H2AB | N | 1.12E-06 |
| rs72694928 | circRNAs |  | N | 1.12E-06 |
| rs6538899 | circRNAs |  | N | 1.12E-06 |
| rs58042601 | TADs,CIRs,TFBRs | FSIP1,LRRC57,STARD9,TP53BP1,RTF1,PLA2G4E | Y | 1.12E-06 |
| rs11668721 | circRNAs,TADs | ZNF208,ENSG00000267383,ENSG00000141979 | Y | 1.12E-06 |
| rs58836765 | TADs | EPS15,AGBL4,USP24 | N | 1.12E-06 |
| rs2336146 | circRNAs,TADs | PBRM1 | Y | 1.12E-06 |
| rs758771 | circRNAs |  | Y | 1.12E-06 |
| rs56273398 | circRNAs,TADs,CIRs | ENSG00000203876,ENSG00000223381,ENSG00000229981,SORCS1,CCDC147 | N | 1.12E-06 |
| rs1061737 | circRNAs,lncRNAs |  | Y | 1.12E-06 |
| rs73068046 | circRNAs,TADs | DLEC1,CCDC66,TRANK1 | N | 1.12E-06 |
| rs55841622 | circRNAs |  | Y | 1.13E-06 |
| rs12625875 | circRNAs |  | N | 1.13E-06 |
| rs12677998 | circRNAs |  | Y | 1.13E-06 |
| rs6073581 | circRNAs,TADs | ENSG00000234948,STK4,BCAS4 | Y | 1.13E-06 |
| rs6073593 | circRNAs,TADs | ENSG00000234948,STK4,BCAS4 | Y | 1.13E-06 |
| rs2238052 | circRNAs |  | N | 1.13E-06 |
| rs4425211 | TADs,CIRs | DLEC1,CCDC66,MLH1,TRANK1,EPM2AIP1 | Y | 1.13E-06 |
| rs2742313 | circRNAs |  | Y | 1.13E-06 |
| rs7613933 | circRNAs |  | Y | 1.13E-06 |
| rs9823697 | circRNAs |  | Y | 1.14E-06 |
| rs1906859 | TADs | ENSG00000236922 | N | 1.14E-06 |
| rs174545 | circRNAs,CIRs | MIR1908 | Y | 1.14E-06 |
| rs9633985 | circRNAs |  | Y | 1.14E-06 |
| rs8141851 | circRNAs |  | Y | 1.15E-06 |
| rs2098013 | circRNAs |  | N | 1.15E-06 |
| rs35203246 | TADs | SH3GL3,ENSG00000259986,ENSG00000166503,ADAMTS7P4 | Y | 1.15E-06 |
| rs767329 | TADs | STK31 | Y | 1.15E-06 |
| rs7858026 | circRNAs |  | N | 1.15E-06 |
| rs78136662 | TADs | MOBP,CTDSPL,ENTPD3-AS1 | N | 1.16E-06 |
| rs2268024 | circRNAs,TADs | VPRBP | Y | 1.16E-06 |
| rs4565840 | circRNAs,TADs | ENSG00000223381,ENSG00000229981,SORCS1,CCDC147 | N | 1.16E-06 |
| rs4478016 | circRNAs |  | Y | 1.16E-06 |
| rs11930030 | CIRs | BBS12 | N | 1.16E-06 |
| rs56207135 | CIRs | ENSG00000255224 | Y | 1.16E-06 |
| rs6866155 | circRNAs |  | N | 1.16E-06 |
| rs2465101 | circRNAs |  | Y | 1.16E-06 |
| rs2239020 | circRNAs |  | N | 1.17E-06 |
| rs174553 | circRNAs |  | Y | 1.17E-06 |
| rs73406545 | TADs,CIRs | ENSG00000260490,FSIP1,TP53BP1,RTF1,ENSG00000261822,PLA2G4E | Y | 1.17E-06 |
| rs2289249 | circRNAs,TADs | CADPS,FHIT,CACNA2D3,CACNA2D2 | Y | 1.17E-06 |
| rs10774033 | circRNAs |  | N | 1.17E-06 |
| rs9998941 | circRNAs |  | N | 1.17E-06 |
| rs12473806 | TADs | STAT1 | N | 1.17E-06 |
| rs61884062 | circRNAs,TADs | TENM4,JRKL,JRKL-AS1,DLG2 | N | 1.17E-06 |
| rs10774032 | circRNAs |  | N | 1.17E-06 |
| rs10459125 | circRNAs |  | Y | 1.18E-06 |
| rs7629072 | circRNAs,CIRs | WDR82 | Y | 1.18E-06 |
| rs7174485 | TADs | PATL2,GPR176,WDR76 | N | 1.18E-06 |
| rs7904096 | circRNAs,TADs | SORCS1,SHOC2 | N | 1.18E-06 |
| rs6445358 | circRNAs,CIRs | TWF2,TLR9 | Y | 1.18E-06 |
| rs12629701 | circRNAs,TADs | PBRM1 | Y | 1.18E-06 |
| rs12571577 | TADs | ENSG00000223381,ENSG00000229981,SORCS1,CCDC147 | N | 1.19E-06 |
| rs2239022 | circRNAs |  | N | 1.19E-06 |
| rs76130670 | circRNAs |  | N | 1.19E-06 |
| rs17300048 | TFBRs | EFTUD1P1,ENSG00000259609 | Y | 1.19E-06 |
| rs2581819 | circRNAs |  | Y | 1.19E-06 |
| rs3810154 | CIRs,TFBRs | IER3,AP1M2,CMC1,AGFG1,QTRT1,CHCHD7,WDR75,RCCD1,ENSG00000258384,ENSG00000263105,MIR638,TUBB4B,PLAG1,FLOT1,ENSG00000272273,ILF3,XRCC6BP1,COQ5,YIPF2,NUTF2,RNU2-59P,MIR5703,DOLPP1,GADD45B,DCUN1D4,RERE,ILF3-AS1 | Y | 1.19E-06 |
| rs17095355 | circRNAs,TADs | SORCS1,SHOC2 | Y | 1.2E-06 |
| rs62021197 | circRNAs |  | Y | 1.2E-06 |
| rs9922678 | circRNAs |  | N | 1.2E-06 |
| rs1060330 | circRNAs |  | Y | 1.2E-06 |
| rs4687650 | circRNAs,TADs | VPRBP | Y | 1.2E-06 |
| rs758174 | circRNAs |  | N | 1.21E-06 |
| rs150966 | circRNAs |  | Y | 1.21E-06 |
| rs111861488 | TADs,CIRs | ENSG00000260490,FSIP1,LRRC57,STARD9,TP53BP1,RTF1,ZNF106,PLA2G4E | Y | 1.21E-06 |
| rs2239023 | circRNAs |  | N | 1.21E-06 |
| rs58096078 | circRNAs,TADs | SORCS1,SHOC2 | N | 1.21E-06 |
| rs10848636 | circRNAs |  | Y | 1.21E-06 |
| rs2255107 | circRNAs |  | Y | 1.21E-06 |
| rs758173 | circRNAs |  | N | 1.22E-06 |
| rs2439434 | circRNAs |  | Y | 1.22E-06 |
| rs2239021 | circRNAs |  | N | 1.22E-06 |
| rs11062156 | circRNAs |  | Y | 1.22E-06 |
| rs174550 | circRNAs,CIRs | FADS2 | Y | 1.22E-06 |
| rs174554 | circRNAs |  | Y | 1.22E-06 |
| rs1545762 | circRNAs |  | Y | 1.22E-06 |
| rs2239024 | circRNAs |  | N | 1.22E-06 |
| rs4765670 | circRNAs |  | N | 1.22E-06 |
| rs12072155 | circRNAs,TADs | EPS15,AGBL4,USP24 | N | 1.22E-06 |
| rs34818287 | circRNAs |  | N | 1.23E-06 |
| rs1567540 | circRNAs,TADs | TENM4,JRKL,JRKL-AS1,DLG2 | N | 1.23E-06 |
| rs7905306 | circRNAs,TADs | ENSG00000223381,ENSG00000229981,SORCS1,CCDC147 | N | 1.23E-06 |
| rs17106184 | circRNAs,TADs | EPS15,AGBL4,USP24 | N | 1.23E-06 |
| rs72694954 | circRNAs |  | N | 1.23E-06 |
| rs3803094 | circRNAs |  | N | 1.23E-06 |
| rs12570375 | TADs | ENSG00000223381,ENSG00000229981,SORCS1,CCDC147 | N | 1.23E-06 |
| rs4765669 | circRNAs |  | N | 1.23E-06 |
| rs11557713 | circRNAs |  | Y | 1.23E-06 |
| rs2239026 | circRNAs |  | N | 1.23E-06 |
| rs10848637 | circRNAs |  | N | 1.24E-06 |
| rs55853893 | CIRs,lncRNAs | ENSG00000255224 | Y | 1.24E-06 |
| rs2989476 | circRNAs |  | N | 1.24E-06 |
| rs758172 | circRNAs |  | N | 1.24E-06 |
| rs2239027 | circRNAs |  | N | 1.24E-06 |
| rs2239025 | circRNAs |  | N | 1.24E-06 |
| rs8079998 | circRNAs |  | Y | 1.24E-06 |
| rs2122517 | circRNAs,TADs,TFBRs | SORCS1,SHOC2,ADD3 | Y | 1.25E-06 |
| rs72898923 | circRNAs |  | N | 1.25E-06 |
| rs1872772 | circRNAs,CIRs | RCOR2,MARK2 | Y | 1.25E-06 |
| rs111625883 | TADs,CIRs | FSIP1,LRRC57,TP53BP1,RTF1,ZNF106,PLA2G4E | Y | 1.25E-06 |
| rs2098167 | TADs | VPRBP | Y | 1.25E-06 |
| rs7614727 | circRNAs,CIRs | WDR82 | Y | 1.25E-06 |
| rs2239019 | circRNAs |  | N | 1.26E-06 |
| rs13098776 | circRNAs,TADs | PBRM1,VPRBP | Y | 1.26E-06 |
| rs113870552 | circRNAs |  | N | 1.26E-06 |
| rs116894709 | circRNAs,TFBRs | TNS4 | N | 1.26E-06 |
| rs2238050 | circRNAs |  | N | 1.26E-06 |
| rs12569906 | TADs | ENSG00000223381,ENSG00000229981,SORCS1,CCDC147 | N | 1.27E-06 |
| rs10501444 | circRNAs,TADs | TENM4,JRKL,JRKL-AS1,DLG2 | N | 1.27E-06 |
| rs2302774 | circRNAs,TADs,TFBRs | ENSG00000174093,SNORD124,CDK12 | Y | 1.27E-06 |
| rs4336110 | circRNAs,TADs | PBRM1 | Y | 1.27E-06 |
| rs6538898 | circRNAs |  | N | 1.27E-06 |
| rs9903808 | circRNAs,CIRs | ENSG00000264895 | Y | 1.27E-06 |
| rs10848627 | circRNAs |  | N | 1.27E-06 |
| rs8064608 | circRNAs,TADs | CA10,PLEKHM1,LRRC37A3 | Y | 1.28E-06 |
| rs6504700 | circRNAs |  | Y | 1.28E-06 |
| rs61489160 | circRNAs,TADs | FSIP1,TP53BP1,RTF1,PLA2G4E | Y | 1.28E-06 |
| rs2899059 | circRNAs,TADs,CIRs | ENSG00000260490,,FSIP1,LRRC57,TP53BP1,RTF1,ENSG00000261822,PLA2G4E | N | 1.28E-06 |
| rs2238054 | circRNAs |  | Y | 1.28E-06 |
| rs36126054 | CIRs,TFBRs | EGLN1P1,ENSG00000254414 | Y | 1.28E-06 |
| rs17586181 | TADs | STAT1 | N | 1.28E-06 |
| rs60812675 | circRNAs,TADs | FSIP1,TP53BP1,RTF1,PLA2G4E | Y | 1.28E-06 |
| rs1014147 | circRNAs,TADs | CA10,PLEKHM1,LRRC37A3 | Y | 1.28E-06 |
| rs13041792 | CIRs,TFBRs | GSS | Y | 1.28E-06 |
| rs731793 | circRNAs,TADs | ENSG00000223381,ENSG00000229981,SORCS1,CCDC147 | Y | 1.28E-06 |
| rs12727057 | circRNAs |  | Y | 1.29E-06 |
| rs6778844 | circRNAs,TADs | CADPS,FHIT,CACNA2D3,CACNA2D2 | Y | 1.29E-06 |
| rs72828218 | TADs,CIRs | ENSG00000223381,ENSG00000229981,SORCS1,CCDC147,XPNPEP1 | N | 1.29E-06 |
| rs55882033 | TADs | MOBP,CTDSPL,ENTPD3-AS1 | N | 1.29E-06 |
| rs7977423 | circRNAs |  | N | 1.29E-06 |
| rs9916077 | circRNAs,TADs,lncRNAs | CA10,PLEKHM1,LRRC37A3 | Y | 1.29E-06 |
| rs2372634 | circRNAs |  | Y | 1.29E-06 |
| rs9905643 | circRNAs,TADs | CA10,PLEKHM1,LRRC37A3 | Y | 1.29E-06 |
| rs7957545 | circRNAs |  | N | 1.29E-06 |
| rs7307199 | circRNAs |  | N | 1.29E-06 |
| rs10795 | CIRs,TFBRs,lncRNAs | EGLN1P1,ENSG00000254414 | Y | 1.29E-06 |
| rs6753285 | TADs | STAT4 | N | 1.29E-06 |
| rs7308351 | circRNAs |  | N | 1.3E-06 |
| rs9934477 | circRNAs |  | N | 1.3E-06 |
| rs9611440 | circRNAs,CIRs | RBX1 | Y | 1.3E-06 |
| rs62021198 | circRNAs |  | Y | 1.3E-06 |
| rs1489555 | TADs | ENSG00000236922 | N | 1.3E-06 |
| rs6884862 | circRNAs |  | N | 1.3E-06 |
| rs4762556 | circRNAs |  | Y | 1.3E-06 |
| rs6073587 | circRNAs,TADs,CIRs | ENSG00000234948,STK4,BCAS4 | Y | 1.3E-06 |
| rs11654208 | circRNAs,TADs | ENSG00000174093,CDK12 | Y | 1.3E-06 |
| rs55728195 | circRNAs,TADs | ENSG00000223381,ENSG00000229981,SORCS1,CCDC147 | N | 1.3E-06 |
| rs7177621 | TADs | PATL2,GPR176,WDR76 | N | 1.3E-06 |
| rs9893910 | circRNAs,TADs | CA10,PLEKHM1,LRRC37A3 | Y | 1.3E-06 |
| rs7299393 | circRNAs |  | Y | 1.3E-06 |
| rs7216962 | circRNAs |  | Y | 1.31E-06 |
| rs59607464 | circRNAs,CIRs | ENSG00000261716 | N | 1.31E-06 |
| rs56349882 | circRNAs,CIRs | ENSG00000261716 | N | 1.31E-06 |
| rs4794214 | circRNAs,TADs | CA10,PLEKHM1,LRRC37A3 | Y | 1.31E-06 |
| rs731591 | TADs | JUP,PCTP,FOXK2,CDC27,ENSG00000263317 | Y | 1.31E-06 |
| rs10848628 | circRNAs |  | Y | 1.31E-06 |
| rs9926303 | circRNAs |  | N | 1.31E-06 |
| rs13182474 | circRNAs |  | N | 1.31E-06 |
| rs13159500 | circRNAs |  | N | 1.32E-06 |
| rs12939682 | circRNAs,TADs,CIRs | ENSG00000174093,THRA,CDK12 | Y | 1.32E-06 |
| rs1376111 | circRNAs,TADs,CIRs | ENSG00000203876,ENSG00000223381,ENSG00000229981,SORCS1,CCDC147 | N | 1.32E-06 |
| rs72898910 | circRNAs,TADs | EPS15,AGBL4,USP24 | N | 1.32E-06 |
| rs11650690 | circRNAs |  | Y | 1.32E-06 |
| rs11917269 | circRNAs,TADs | CADPS,ZDHHC3,CACNA2D2 | Y | 1.32E-06 |
| rs11231600 | CIRs,TFBRs | ENSG00000256280 | N | 1.33E-06 |
| rs11650030 | circRNAs |  | Y | 1.33E-06 |
| rs11167248 | circRNAs,CIRs | GSS, | Y | 1.33E-06 |
| rs2239549 | TADs | VPRBP | Y | 1.33E-06 |
| rs58426216 | circRNAs,CIRs | ENSG00000272993 | N | 1.33E-06 |
| rs10745839 | circRNAs |  | N | 1.33E-06 |
| rs10848629 | circRNAs |  | N | 1.34E-06 |
| rs11954508 | circRNAs |  | N | 1.34E-06 |
| rs4246215 | CIRs | BEST1 | Y | 1.34E-06 |
| rs10106152 | circRNAs,CIRs | MATN2, | N | 1.34E-06 |
| rs62202941 | circRNAs |  | N | 1.34E-06 |
| rs113194201 | circRNAs,TADs | FSIP1,TP53BP1,RTF1,PLA2G4E | Y | 1.34E-06 |
| rs6538895 | circRNAs |  | N | 1.35E-06 |
| rs7297582 | circRNAs |  | Y | 1.35E-06 |
| rs4603535 | TFBRs | SCAND2P | Y | 1.35E-06 |
| rs6798246 | circRNAs,TADs | CADPS,FHIT,CACNA2D3,CACNA2D2 | Y | 1.35E-06 |
| rs2635337 | TADs,lncRNAs | ENSG00000236922 | N | 1.35E-06 |
| rs2372636 | circRNAs |  | N | 1.35E-06 |
| rs28410310 | circRNAs |  | Y | 1.36E-06 |
| rs10848633 | circRNAs |  | Y | 1.36E-06 |
| rs4762554 | circRNAs |  | N | 1.36E-06 |
| rs4762555 | circRNAs |  | N | 1.36E-06 |
| rs12453388 | circRNAs |  | Y | 1.36E-06 |
| rs9901536 | TADs | JUP,PCTP,FOXK2,CDC27,ENSG00000263317 | Y | 1.36E-06 |
| rs7953690 | circRNAs |  | N | 1.36E-06 |
| rs2481979 | circRNAs,lncRNAs |  | N | 1.38E-06 |
| rs10860390 | circRNAs |  | N | 1.38E-06 |
| rs10507107 | circRNAs |  | N | 1.38E-06 |
| rs77594613 | TADs | STK31 | N | 1.38E-06 |
| rs13377046 | circRNAs,TADs | SORCS1,SHOC2 | N | 1.38E-06 |
| rs2163725 | lncRNAs |  | N | 1.39E-06 |
| rs12277481 | circRNAs |  | N | 1.39E-06 |
| rs9988702 | TADs | ENSG00000223381,ENSG00000229981,SORCS1,CCDC147 | N | 1.39E-06 |
| rs6591825 | circRNAs |  | Y | 1.39E-06 |
| rs8054791 | circRNAs |  | N | 1.39E-06 |
| rs2872530 | circRNAs |  | N | 1.4E-06 |
| rs12449426 | circRNAs |  | Y | 1.4E-06 |
| rs56272614 | circRNAs |  | N | 1.4E-06 |
| rs35289073 | circRNAs,CIRs | MBTD1 | Y | 1.4E-06 |
| rs4765902 | circRNAs |  | Y | 1.4E-06 |
| rs10423449 | CIRs,TFBRs | GMIP,YJEFN3,ENSG00000258674 | Y | 1.4E-06 |
| rs1029871 | circRNAs,TADs | NEK4 | Y | 1.4E-06 |
| rs921351 | TADs | ENSG00000223381,ENSG00000229981,SORCS1,CCDC147 | Y | 1.41E-06 |
| rs2372637 | circRNAs |  | N | 1.41E-06 |
| rs10792422 | circRNAs |  | N | 1.41E-06 |
| rs1984616 | TADs | ENSG00000236922 | N | 1.41E-06 |
| rs7069789 | circRNAs,TADs | SORCS1,SHOC2 | N | 1.41E-06 |
| rs7961981 | circRNAs |  | N | 1.41E-06 |
| rs12265204 | circRNAs,TADs | SORCS1,SHOC2 | N | 1.41E-06 |
| rs958086 | circRNAs,TADs | SORCS1,SHOC2 | N | 1.41E-06 |
| rs73406534 | circRNAs,TADs | FSIP1,TP53BP1,RTF1,PLA2G4E | Y | 1.42E-06 |
| rs381523 | CIRs | ENSG00000224086,ENSG00000272954 | Y | 1.42E-06 |
| rs923247 | circRNAs |  | N | 1.42E-06 |
| rs174573 | circRNAs |  | Y | 1.42E-06 |
| rs113358355 | circRNAs,TADs | FSIP1,TP53BP1,RTF1,PLA2G4E | Y | 1.42E-06 |
| rs10792423 | circRNAs |  | N | 1.42E-06 |
| rs884480 | TADs,CIRs,TFBRs | ZNF208,ATP13A1,GMIP,ENSG00000267419,YJEFN3,NDUFA13,LPAR2,ENSG00000267383,ENSG00000258674,RNU11,ENSG00000141979,PBX4 | Y | 1.42E-06 |
| rs9729667 | CIRs | NEXN | Y | 1.42E-06 |
| rs1961959 | circRNAs,TADs | CADPS,FHIT,CACNA2D3,CACNA2D2 | Y | 1.43E-06 |
| rs11605500 | circRNAs,CIRs | C11orf95,,ENSG00000255651,MARK2 | N | 1.43E-06 |
| rs7961789 | circRNAs |  | N | 1.43E-06 |
| rs494678 | circRNAs |  | Y | 1.44E-06 |
| rs2130237 | TADs | ENSG00000236922 | N | 1.44E-06 |
| rs2372635 | circRNAs |  | N | 1.44E-06 |
| rs2247228 | TADs | ENSG00000236922 | N | 1.44E-06 |
| rs7970121 | circRNAs |  | Y | 1.45E-06 |
| rs13078422 | circRNAs,TADs | PBRM1 | Y | 1.45E-06 |
| rs2239551 | TADs | VPRBP | Y | 1.45E-06 |
| rs36094159 | TADs | PATL2,GPR176,WDR76 | N | 1.45E-06 |
| rs3862006 | circRNAs,TADs | SORCS1,SHOC2 | Y | 1.45E-06 |
| rs2304275 | circRNAs,CIRs | PRKAG1 | Y | 1.45E-06 |
| rs2329883 | TFBRs | ENSG00000225544 | Y | 1.45E-06 |
| rs10848634 | circRNAs |  | Y | 1.46E-06 |
| rs12916712 | circRNAs,CIRs | ZSCAN2 | Y | 1.46E-06 |
| rs9828491 | circRNAs |  | Y | 1.46E-06 |
| rs67651814 | circRNAs,TADs,CIRs | JPH2,SULF2,ENSG00000234948,STK4-AS1,STAU1 | N | 1.46E-06 |
| rs2635342 | TADs | ENSG00000236922 | N | 1.46E-06 |
| rs2581803 | circRNAs |  | Y | 1.46E-06 |
| rs7974917 | circRNAs |  | Y | 1.47E-06 |
| rs12267714 | circRNAs,TADs | SORCS1,SHOC2 | Y | 1.47E-06 |
| rs72694959 | CIRs | VPS45 | N | 1.47E-06 |
| rs55802315 | CIRs | HIST2H2AA4,VPS45 | N | 1.47E-06 |
| rs7110843 | circRNAs |  | Y | 1.47E-06 |
| rs113028838 | circRNAs,CIRs,lncRNAs | ZNF106 | N | 1.47E-06 |
| rs4980528 | circRNAs,CIRs | RCOR2 | N | 1.47E-06 |
| rs11109725 | circRNAs |  | N | 1.47E-06 |
| rs1486936 | circRNAs |  | Y | 1.48E-06 |
| rs6478404 | TADs | CDK5RAP2,ASTN2 | Y | 1.48E-06 |
| rs7108375 | circRNAs |  | N | 1.48E-06 |
| rs2372642 | circRNAs |  | N | 1.48E-06 |
| rs72694942 | CIRs | PLEKHO1 | N | 1.49E-06 |
| rs2215417 | circRNAs,CIRs | MBTD1 | Y | 1.49E-06 |
| rs13166360 | circRNAs |  | N | 1.49E-06 |
| rs7000180 | CIRs | HSF1 | Y | 1.49E-06 |
| rs8079104 | TADs | JUP,PCTP,FOXK2,CDC27,ENSG00000263317 | Y | 1.49E-06 |
| rs734164 | TADs | ENSG00000223381,ENSG00000229981,SORCS1,CCDC147 | N | 1.49E-06 |
| rs150970 | TFBRs | ENSG00000259244 | Y | 1.49E-06 |
| rs7314469 | circRNAs |  | Y | 1.49E-06 |
| rs2932188 | TADs | ENSG00000236922 | N | 1.5E-06 |
| rs12570612 | circRNAs,TADs | ENSG00000223381,ENSG00000229981,SORCS1,CCDC147 | N | 1.5E-06 |
| rs6801235 | circRNAs |  | Y | 1.5E-06 |
| rs11655655 | circRNAs,TADs | ENSG00000174093,CDK12 | Y | 1.5E-06 |
| rs11714419 | circRNAs,TADs | PBRM1 | Y | 1.5E-06 |
| rs3789577 | circRNAs |  | N | 1.5E-06 |
| rs12267704 | circRNAs,TADs | SORCS1,SHOC2 | N | 1.5E-06 |
| rs2888379 | circRNAs |  | N | 1.51E-06 |
| rs12247564 | circRNAs,TADs | SORCS1,SHOC2 | N | 1.51E-06 |
| rs3208509 | CIRs | ENSG00000261716 | Y | 1.51E-06 |
| rs12259261 | circRNAs,TADs | SORCS1,SHOC2 | N | 1.51E-06 |
| rs55864908 | circRNAs |  | N | 1.52E-06 |
| rs35862056 | circRNAs |  | N | 1.52E-06 |
| rs921348 | circRNAs,TADs,CIRs | ENSG00000203876,ENSG00000223381,ENSG00000229981,SORCS1,CCDC147 | Y | 1.52E-06 |
| rs174547 | circRNAs,CIRs | GRPEL1 | Y | 1.52E-06 |
| rs998817 | TADs | ENSG00000236922 | Y | 1.53E-06 |
| rs10942267 | circRNAs |  | N | 1.53E-06 |
| rs4980529 | circRNAs |  | N | 1.53E-06 |
| rs4263076 | lncRNAs |  | N | 1.53E-06 |
| rs1412071 | TADs | CDK5RAP2,ASTN2 | N | 1.54E-06 |
| rs73404717 | circRNAs,CIRs | ZNF106 | N | 1.54E-06 |
| rs7105288 | circRNAs |  | Y | 1.54E-06 |
| rs734165 | TADs | ENSG00000223381,ENSG00000229981,SORCS1,CCDC147 | Y | 1.54E-06 |
| rs752584 | TADs,CIRs,TFBRs | ENSG00000223381,WDR5B,ENSG00000229981,ENSG00000228397,ENSG00000272758,SORCS1,CCDC147,LINC00339,XPNPEP1 | N | 1.55E-06 |
| rs2238053 | circRNAs |  | N | 1.55E-06 |
| rs35795311 | TADs | PATL2,GPR176,WDR76 | N | 1.55E-06 |
| rs6120798 | circRNAs |  | Y | 1.55E-06 |
| rs867616 | circRNAs |  | N | 1.55E-06 |
| rs2372719 | circRNAs |  | Y | 1.56E-06 |
| rs4530734 | circRNAs |  | N | 1.56E-06 |
| rs7951079 | circRNAs |  | Y | 1.56E-06 |
| rs7118913 | circRNAs |  | Y | 1.56E-06 |
| rs10860389 | circRNAs |  | N | 1.56E-06 |
| rs3789576 | circRNAs |  | N | 1.57E-06 |
| rs8076387 | circRNAs,TADs | CA10,PLEKHM1,LRRC37A3 | Y | 1.57E-06 |
| rs12246851 | circRNAs,TADs | SORCS1,SHOC2 | N | 1.57E-06 |
| rs10860388 | circRNAs |  | N | 1.57E-06 |
| rs12250404 | circRNAs,TADs | SORCS1,SHOC2 | N | 1.57E-06 |
| rs7939351 | circRNAs |  | N | 1.57E-06 |
| rs12571674 | circRNAs,TADs | ENSG00000223381,ENSG00000229981,SORCS1,CCDC147 | Y | 1.58E-06 |
| rs1461359 | circRNAs |  | N | 1.58E-06 |
| rs2581816 | circRNAs |  | Y | 1.58E-06 |
| rs1817342 | circRNAs |  | N | 1.59E-06 |
| rs12517386 | circRNAs |  | N | 1.59E-06 |
| rs11194933 | circRNAs,TADs | SORCS1,SHOC2 | N | 1.6E-06 |
| rs113841607 | TADs | RAB3B,AGBL4,LRP8,FAF1 | N | 1.6E-06 |
| rs9933832 | circRNAs |  | N | 1.61E-06 |
| rs77927505 | circRNAs |  | N | 1.61E-06 |
| rs10514230 | circRNAs |  | N | 1.61E-06 |
| rs10735361 | circRNAs |  | N | 1.61E-06 |
| rs68093531 | TADs,TFBRs | SLAMF9,AIM2 | N | 1.61E-06 |
| rs2268025 | circRNAs,TADs | NEK4 | Y | 1.62E-06 |
| rs11657291 | TADs | PCTP | N | 1.62E-06 |
| rs7314011 | circRNAs |  | N | 1.62E-06 |
| rs2239550 | TADs | VPRBP | Y | 1.62E-06 |
| rs1381171 | circRNAs |  | N | 1.63E-06 |
| rs17126886 | TADs | ENSG00000223381,ENSG00000229981,SORCS1,CCDC147 | N | 1.63E-06 |
| rs1108073 | circRNAs,TFBRs | CACNA1C-AS4 | Y | 1.63E-06 |
| rs2372640 | circRNAs |  | N | 1.63E-06 |
| rs3818253 | circRNAs |  | Y | 1.64E-06 |
| rs73006705 | TADs | ZNF208,ENSG00000267383,ENSG00000141979 | N | 1.64E-06 |
| rs11740689 | circRNAs |  | N | 1.64E-06 |
| rs11194923 | TADs | ENSG00000223381,ENSG00000229981,SORCS1,CCDC147 | N | 1.64E-06 |
| rs7708829 | circRNAs |  | N | 1.64E-06 |
| rs7944519 | circRNAs |  | Y | 1.65E-06 |
| rs35004449 | circRNAs |  | Y | 1.65E-06 |
| rs55717725 | circRNAs,TADs | RBM20,SHOC2,SORCS1,CCDC147,MXI1,XPNPEP1,ENSG00000223381,NRAP,ENSG00000229981,GUCY2GP,HELLS,INPP5A | N | 1.65E-06 |
| rs7937314 | circRNAs,CIRs | MARK2 | N | 1.65E-06 |
| rs10897452 | circRNAs |  | N | 1.65E-06 |
| rs12064532 | CIRs | VPS45,OTUD7B | N | 1.65E-06 |
| rs2019611 | lncRNAs |  | Y | 1.65E-06 |
| rs2372730 | circRNAs |  | N | 1.66E-06 |
| rs10745840 | circRNAs |  | N | 1.66E-06 |
| rs2888378 | circRNAs |  | Y | 1.66E-06 |
| rs11231637 | circRNAs,CIRs | MARK2 | Y | 1.66E-06 |
| rs11194924 | TADs | ENSG00000223381,ENSG00000229981,SORCS1,CCDC147 | N | 1.67E-06 |
| rs11062162 | circRNAs |  | N | 1.67E-06 |
| rs12488527 | circRNAs,TADs | PBRM1 | Y | 1.67E-06 |
| rs34003767 | circRNAs,TADs,CIRs | ENSG00000174093,THRA,CDK12 | Y | 1.67E-06 |
| rs736408 | circRNAs,TADs,CIRs | VPRBP,ITIH3 | Y | 1.67E-06 |
| rs7073324 | circRNAs,TADs | SORCS1,SHOC2 | N | 1.68E-06 |
| rs11130327 | circRNAs,TADs | NEK4 | Y | 1.68E-06 |
| rs97384 | circRNAs |  | Y | 1.68E-06 |
| rs12805133 | circRNAs |  | Y | 1.68E-06 |
| rs2155201 | circRNAs |  | Y | 1.68E-06 |
| rs112114764 | CIRs,TFBRs | HDAC5,RYBP,C17orf53,ATXN7L3 | Y | 1.68E-06 |
| rs8076679 | circRNAs |  | Y | 1.69E-06 |
| rs150965 | circRNAs |  | Y | 1.69E-06 |
| rs10897454 | circRNAs |  | Y | 1.7E-06 |
| rs12257668 | circRNAs,TADs | ENSG00000223381,ENSG00000229981,SORCS1,CCDC147 | N | 1.71E-06 |
| rs4687657 | lncRNAs |  | Y | 1.72E-06 |
| rs11709448 | circRNAs,TADs | PBRM1,VPRBP | Y | 1.72E-06 |
| rs6120793 | circRNAs |  | Y | 1.72E-06 |
| rs6119560 | circRNAs |  | Y | 1.72E-06 |
| rs352139 | circRNAs,CIRs | TWF2 | Y | 1.72E-06 |
| rs17126902 | circRNAs,TADs | ENSG00000223381,ENSG00000229981,SORCS1,CCDC147 | Y | 1.73E-06 |
| rs11194922 | TADs | ENSG00000223381,ENSG00000229981,SORCS1,CCDC147 | N | 1.73E-06 |
| rs8906 | CIRs,TFBRs,lncRNAs | GLT8D1,,SPCS1,SMIM4 | Y | 1.73E-06 |
| rs174541 | CIRs | FADS3 | Y | 1.73E-06 |
| rs7948656 | circRNAs |  | N | 1.74E-06 |
| rs2300149 | TADs | VPRBP | Y | 1.74E-06 |
| rs2710331 | circRNAs,TADs | VPRBP | Y | 1.74E-06 |
| rs9607340 | CIRs,TFBRs | YPEL1,MAPK1,ZBTB21,RNA5SP493,RCOR1,MRPL33 | Y | 1.74E-06 |
| rs7623199 | circRNAs,TADs | PBRM1 | Y | 1.74E-06 |
| rs9295354 | circRNAs |  | N | 1.74E-06 |
| rs35211965 | circRNAs,TADs | PBRM1 | Y | 1.75E-06 |
| rs6584974 | CIRs | NIFKP9 | N | 1.75E-06 |
| rs174572 | circRNAs,TFBRs | FADS1 | Y | 1.75E-06 |
| rs111791123 | circRNAs |  | N | 1.76E-06 |
| rs72673100 | CIRs,lncRNAs | TMSB10 | N | 1.76E-06 |
| rs11097326 | TADs | GRID2,CCSER1 | N | 1.76E-06 |
| rs56982416 | circRNAs |  | N | 1.76E-06 |
| rs2401448 | TADs | ENSG00000259986,ENSG00000166503,ADAMTS7P4 | Y | 1.76E-06 |
| rs2372721 | circRNAs |  | Y | 1.77E-06 |
| rs17127148 | CIRs | ENSG00000228417 | N | 1.77E-06 |
| rs1885116 | circRNAs |  | Y | 1.77E-06 |
| rs149987188 | TADs | SH3GL3,ENSG00000259986,ENSG00000166503,ADAMTS7P4 | Y | 1.77E-06 |
| rs1885117 | circRNAs |  | Y | 1.77E-06 |
| rs12632265 | circRNAs,TADs | PBRM1 | Y | 1.77E-06 |
| rs2276815 | circRNAs |  | Y | 1.78E-06 |
| rs7085888 | circRNAs,TADs,CIRs,TFBRs | ENSG00000203876,ENSG00000223381,ENSG00000229981,SORCS1,CCDC147 | N | 1.78E-06 |
| rs73068042 | circRNAs,TADs | TRANK1,GOLGA4 | N | 1.78E-06 |
| rs2239547 | CIRs | NEK4 | Y | 1.78E-06 |
| rs10774030 | circRNAs |  | Y | 1.78E-06 |
| rs67346571 | circRNAs |  | N | 1.78E-06 |
| rs13065851 | circRNAs,TADs | PBRM1,VPRBP | Y | 1.78E-06 |
| rs72828232 | circRNAs,TADs | SORCS1,SHOC2 | N | 1.79E-06 |
| rs2239038 | circRNAs |  | Y | 1.79E-06 |
| rs2286798 | TADs | VPRBP | Y | 1.79E-06 |
| rs7721321 | circRNAs |  | N | 1.8E-06 |
| rs9371601 | circRNAs,TADs | OPRM1,SYNE1,IPCEF1,CCDC170 | Y | 1.8E-06 |
| rs4980503 | circRNAs |  | N | 1.8E-06 |
| rs6768697 | circRNAs,TADs | PBRM1 | Y | 1.81E-06 |
| rs13061253 | circRNAs,TADs | PBRM1 | Y | 1.81E-06 |
| rs2336145 | circRNAs,TADs | PBRM1 | Y | 1.81E-06 |
| rs11062146 | circRNAs |  | N | 1.81E-06 |
| rs2635218 | TADs | ENSG00000236922 | N | 1.81E-06 |
| rs10777955 | circRNAs |  | N | 1.81E-06 |
| rs12619873 | TADs | STAT4 | N | 1.82E-06 |
| rs11162556 | TADs | LPHN2,GIPC2 | N | 1.83E-06 |
| rs4687552 | circRNAs,TADs,lncRNAs | VPRBP | Y | 1.83E-06 |
| rs9611441 | circRNAs |  | Y | 1.83E-06 |
| rs35409678 | circRNAs,TADs | PBRM1 | Y | 1.84E-06 |
| rs9610458 | circRNAs |  | Y | 1.85E-06 |
| rs17126877 | circRNAs,TADs | RBM20,SHOC2,SORCS1,CCDC147,MXI1,XPNPEP1,ENSG00000223381,NRAP,ENSG00000229981,GUCY2GP,HELLS,INPP5A | N | 1.85E-06 |
| rs6504701 | circRNAs |  | Y | 1.85E-06 |
| rs10897453 | circRNAs |  | N | 1.86E-06 |
| rs16994801 | circRNAs |  | N | 1.86E-06 |
| rs189049 | TADs,CIRs | HDAC5,ASB16-AS1 | Y | 1.86E-06 |
| rs55781439 | circRNAs,TADs | RBM20,SHOC2,SORCS1,CCDC147,MXI1,XPNPEP1,ENSG00000223381,NRAP,ENSG00000229981,GUCY2GP,HELLS,INPP5A | N | 1.87E-06 |
| rs12156491 | TADs | CDK5RAP2,ASTN2 | N | 1.87E-06 |
| rs4808979 | TADs | ENSG00000141979 | N | 1.87E-06 |
| rs13157978 | circRNAs |  | N | 1.87E-06 |
| rs2900160 | TADs | CDK5RAP2,ASTN2 | N | 1.87E-06 |
| rs148853476 | TADs | ENSG00000236922 | N | 1.87E-06 |
| rs2271431 | circRNAs |  | Y | 1.88E-06 |
| rs12486847 | circRNAs,TADs | PBRM1 | Y | 1.88E-06 |
| rs876333 | circRNAs |  | N | 1.88E-06 |
| rs113244990 | circRNAs |  | Y | 1.88E-06 |
| rs7102006 | circRNAs |  | N | 1.89E-06 |
| rs477223 | TADs,TFBRs | DENND2A,TMEM178B | Y | 1.89E-06 |
| rs11078935 | circRNAs,TADs | ENSG00000174093,CDK12 | Y | 1.89E-06 |
| rs17817923 | circRNAs |  | N | 1.89E-06 |
| rs34174818 | TADs,CIRs | ZNF208,ENSG00000267383,ENSG00000141979,ZNF101 | N | 1.89E-06 |
| rs4837644 | TADs | CDK5RAP2,ASTN2 | N | 1.9E-06 |
| rs1478124 | TADs | STAT4 | N | 1.9E-06 |
| rs72934134 | circRNAs |  | N | 1.91E-06 |
| rs11130307 | circRNAs,TADs | PBRM1 | Y | 1.92E-06 |
| rs12632381 | circRNAs,TADs | PBRM1 | Y | 1.92E-06 |
| rs80011513 | circRNAs |  | N | 1.92E-06 |
| rs2061733 | TADs | STAT4 | N | 1.93E-06 |
| rs42421 | circRNAs |  | N | 1.93E-06 |
| rs5755694 | circRNAs |  | Y | 1.93E-06 |
| rs2378258 | circRNAs |  | Y | 1.93E-06 |
| rs28820635 | circRNAs |  | Y | 1.93E-06 |
| rs55683820 | circRNAs,TADs | RBM20,SHOC2,SORCS1,CCDC147,MXI1,XPNPEP1,ENSG00000223381,NRAP,ENSG00000229981,GUCY2GP,HELLS,INPP5A | N | 1.94E-06 |
| rs11194920 | TADs | ENSG00000223381,ENSG00000229981,SORCS1,CCDC147 | N | 1.94E-06 |
| rs34115864 | circRNAs,TADs | PBRM1 | Y | 1.95E-06 |
| rs11708075 | circRNAs,TADs | PBRM1 | Y | 1.95E-06 |
| rs6805539 | circRNAs,TADs | PBRM1 | Y | 1.96E-06 |
| rs4130905 | circRNAs,TADs | PBRM1 | Y | 1.96E-06 |
| rs12833856 | TADs | SVOP,CKAP4,SH2B3,TCHP | N | 1.97E-06 |
| rs7946447 | circRNAs |  | N | 1.97E-06 |
| rs16843284 | CIRs | BCAS4 | N | 1.97E-06 |
| rs880090 | circRNAs,CIRs,TFBRs | LPAR2 | Y | 1.97E-06 |
| rs12571872 | TADs | ENSG00000223381,NRAP,ENSG00000229981,RBM20,SORCS1,SHOC2,CCDC147,GUCY2GP,MXI1,XPNPEP1,INPP5A,HELLS | N | 1.97E-06 |
| rs12570423 | TADs | ENSG00000223381,ENSG00000229981,SORCS1,CCDC147 | N | 1.98E-06 |
| rs11194917 | TADs | ENSG00000223381,ENSG00000229981,SORCS1,CCDC147 | N | 1.98E-06 |
| rs13082208 | circRNAs,TADs | NEK4 | Y | 1.98E-06 |
| rs8142455 | circRNAs |  | Y | 1.99E-06 |
| rs2254388 | CIRs,TFBRs | PACS1,PARD6B,ENSG00000255038 | N | 1.99E-06 |
| rs2266979 | circRNAs,TADs | CABIN1 | Y | 2E-06 |
| rs6088678 | circRNAs |  | Y | 2E-06 |
| rs8078174 | CIRs | C17orf53 | Y | 2.01E-06 |
| rs7223165 | circRNAs |  | Y | 2.02E-06 |
| rs3802941 | circRNAs |  | N | 2.02E-06 |
| rs13095332 | circRNAs,TADs,TFBRs | ENSG00000271137,NEK4 | Y | 2.02E-06 |
| rs4687636 | circRNAs,TADs | PBRM1 | Y | 2.03E-06 |
| rs12570230 | TADs | ENSG00000223381,ENSG00000229981,SORCS1,CCDC147 | N | 2.03E-06 |
| rs11168827 | circRNAs |  | Y | 2.03E-06 |
| rs12570145 | circRNAs,TADs | ENSG00000223381,ENSG00000229981,SORCS1,CCDC147 | N | 2.05E-06 |
| rs2455636 | TADs | EPS15,STIL,AGBL4-IT1,AGBL4,SMIM12,LRP8,USP24,TTC39A | N | 2.05E-06 |
| rs6120797 | circRNAs |  | Y | 2.05E-06 |
| rs670104 | circRNAs |  | Y | 2.06E-06 |
| rs4926409 | CIRs | PLEKHO1 | N | 2.06E-06 |
| rs17126883 | TADs | ENSG00000223381,ENSG00000229981,SORCS1,CCDC147 | Y | 2.06E-06 |
| rs11585823 | TADs | EPS15,STIL,AGBL4-IT1,AGBL4,SMIM12,LRP8,USP24,TTC39A | N | 2.06E-06 |
| rs17264436 | circRNAs,TADs | PBRM1 | Y | 2.06E-06 |
| rs11087101 | circRNAs |  | Y | 2.06E-06 |
| rs34610142 | circRNAs,TADs | PBRM1 | Y | 2.06E-06 |
| rs1717671 | circRNAs |  | N | 2.06E-06 |
| rs2286800 | circRNAs,TADs | NEK4 | Y | 2.06E-06 |
| rs35166191 | TADs | EPS15,STIL,AGBL4-IT1,AGBL4,SMIM12,LRP8,USP24,TTC39A | N | 2.06E-06 |
| rs6087659 | circRNAs |  | Y | 2.07E-06 |
| rs11152349 | circRNAs |  | Y | 2.07E-06 |
| rs76910682 | TADs | EPS15,STIL,AGBL4-IT1,AGBL4,SMIM12,LRP8,USP24,TTC39A | N | 2.07E-06 |
| rs6976 | circRNAs |  | Y | 2.07E-06 |
| rs62253733 | circRNAs,TADs | PBRM1,VPRBP | Y | 2.07E-06 |
| rs72896969 | TADs | EPS15,STIL,AGBL4-IT1,AGBL4,SMIM12,LRP8,USP24,TTC39A | N | 2.08E-06 |
| rs34537256 | circRNAs,TADs | PBRM1,VPRBP | Y | 2.08E-06 |
| rs2391000 | TADs | STK31 | Y | 2.08E-06 |
| rs7589572 | TADs | STAT1 | N | 2.08E-06 |
| rs4687638 | circRNAs,TADs | PBRM1,VPRBP | Y | 2.08E-06 |
| rs7080576 | circRNAs,TADs | ENSG00000223381,ENSG00000229981,SORCS1,CCDC147 | Y | 2.08E-06 |
| rs4977836 | lncRNAs |  | N | 2.08E-06 |
| rs11577346 | circRNAs,CIRs | HIST2H2AC | N | 2.09E-06 |
| rs60213435 | circRNAs |  | N | 2.09E-06 |
| rs2372639 | circRNAs |  | N | 2.1E-06 |
| rs4842849 | TADs | SH3GL3,ENSG00000259986,ENSG00000166503,ADAMTS7P4 | Y | 2.1E-06 |
| rs67002381 | circRNAs |  | N | 2.1E-06 |
| rs2372638 | circRNAs |  | N | 2.1E-06 |
| rs4926859 | TADs | EPS15,STIL,AGBL4-IT1,AGBL4,SMIM12,LRP8,USP24,TTC39A | N | 2.1E-06 |
| rs4926406 | circRNAs |  | N | 2.1E-06 |
| rs72898921 | circRNAs |  | N | 2.11E-06 |
| rs35272691 | TADs | ENSG00000174093 | Y | 2.11E-06 |
| rs2013125 | circRNAs |  | Y | 2.11E-06 |
| rs6120790 | circRNAs |  | Y | 2.12E-06 |
| rs12487445 | circRNAs,TADs | PBRM1 | Y | 2.12E-06 |
| rs12570820 | circRNAs,TADs | SORCS1,SHOC2 | N | 2.12E-06 |
| rs7139247 | circRNAs |  | N | 2.12E-06 |
| rs62028142 | TADs | SH3GL3,ENSG00000259986,ENSG00000166503,ADAMTS7P4 | Y | 2.12E-06 |
| rs10190023 | lncRNAs |  | N | 2.12E-06 |
| rs6967482 | TADs | DENND2A | Y | 2.12E-06 |
| rs6088685 | circRNAs |  | Y | 2.13E-06 |
| rs13433216 | circRNAs |  | Y | 2.13E-06 |
| rs6984358 | circRNAs |  | N | 2.13E-06 |
| rs9295358 | circRNAs |  | N | 2.13E-06 |
| rs4842854 | TADs | SH3GL3,ENSG00000259986,ENSG00000166503,ADAMTS7P4 | Y | 2.13E-06 |
| rs991360 | circRNAs |  | Y | 2.14E-06 |
| rs11917680 | circRNAs |  | Y | 2.14E-06 |
| rs4762548 | circRNAs |  | Y | 2.14E-06 |
| rs261625 | circRNAs |  | Y | 2.14E-06 |
| rs11635597 | lncRNAs |  | Y | 2.15E-06 |
| rs10774034 | circRNAs |  | N | 2.15E-06 |
| rs138328 | TADs | TNRC6B | Y | 2.15E-06 |
| rs1474784 | circRNAs |  | N | 2.16E-06 |
| rs752075 | circRNAs |  | Y | 2.16E-06 |
| rs2746612 | TADs | EPS15,STIL,AGBL4-IT1,AGBL4,SMIM12,LRP8,USP24,TTC39A | N | 2.16E-06 |
| rs1060293 | circRNAs |  | Y | 2.16E-06 |
| rs10792421 | CIRs,TFBRs | C11orf95,RCOR2,NAA40,ENSG00000256280,ENSG00000255651,MARK2 | Y | 2.16E-06 |
| rs892087 | circRNAs |  | Y | 2.16E-06 |
| rs11739075 | circRNAs |  | N | 2.16E-06 |
| rs6464278 | TADs | SSBP1,DENND2A | Y | 2.17E-06 |
| rs2372724 | circRNAs |  | N | 2.18E-06 |
| rs11720432 | circRNAs,TADs | PBRM1,VPRBP | Y | 2.18E-06 |
| rs6419378 | circRNAs |  | N | 2.18E-06 |
| rs6120804 | circRNAs |  | Y | 2.18E-06 |
| rs2635347 | TADs | ENSG00000236922 | N | 2.19E-06 |
| rs3825267 | circRNAs |  | N | 2.19E-06 |
| rs1278523 | TADs | EPS15,AGBL4,USP24 | N | 2.19E-06 |
| rs34173654 | circRNAs,TADs | PBRM1 | Y | 2.19E-06 |
| rs6421686 | circRNAs |  | N | 2.19E-06 |
| rs3860265 | TADs | SH3GL3,ENSG00000259986,ENSG00000166503,ADAMTS7P4 | Y | 2.2E-06 |
| rs6762813 | circRNAs,CIRs,TFBRs | SNORD69 | Y | 2.2E-06 |
| rs35107891 | circRNAs,TADs | PBRM1 | Y | 2.2E-06 |
| rs8501 | circRNAs |  | Y | 2.2E-06 |
| rs59804002 | TADs | ENSG00000223381,ENSG00000229981,SORCS1,CCDC147 | N | 2.2E-06 |
| rs1134020 | TADs | SH3GL3,ENSG00000259986,ENSG00000166503,ADAMTS7P4 | Y | 2.2E-06 |
| rs2401432 | TADs | SH3GL3,ENSG00000259986,ENSG00000166503,ADAMTS7P4 | Y | 2.21E-06 |
| rs78808294 | circRNAs |  | N | 2.21E-06 |
| rs72906818 | TADs | FAF1 | N | 2.21E-06 |
| rs4980507 | circRNAs |  | N | 2.22E-06 |
| rs34774166 | circRNAs |  | N | 2.22E-06 |
| rs4794219 | circRNAs,TADs | CA10,PLEKHM1,LRRC37A3 | Y | 2.22E-06 |
| rs10865973 | TADs | PBRM1 | Y | 2.22E-06 |
| rs6414569 | circRNAs,TADs | PBRM1 | Y | 2.22E-06 |
| rs8063193 | circRNAs |  | N | 2.23E-06 |
| rs13076193 | circRNAs,CIRs,TFBRs | SNORD69,ENSG00000222345 | Y | 2.23E-06 |
| rs1529115 | TADs | STAT4 | N | 2.24E-06 |
| rs3788332 | circRNAs |  | Y | 2.24E-06 |
| rs6031991 | TFBRs | KCNS1 | N | 2.24E-06 |
| rs6088686 | circRNAs |  | Y | 2.24E-06 |
| rs11177 | circRNAs,CIRs,TFBRs | ENSG00000252787,PBRM1,SNORD19 | Y | 2.24E-06 |
| rs113041465 | TADs | STK31 | N | 2.25E-06 |
| rs55698168 | CIRs | TTC37,ARSK,ENSG00000251314 | N | 2.25E-06 |
| rs2635329 | TADs | ENSG00000236922 | N | 2.25E-06 |
| rs57367626 | circRNAs |  | N | 2.25E-06 |
| rs4765911 | circRNAs |  | N | 2.25E-06 |
| rs325380 | lncRNAs |  | Y | 2.26E-06 |
| rs10780035 | circRNAs,TADs | NEK4 | Y | 2.26E-06 |
| rs3746444 | Mature miRNA region,TFBRs | MIR499A | Y | 2.26E-06 |
| rs6120828 | circRNAs |  | Y | 2.26E-06 |
| rs12123055 | TADs | EPS15,STIL,AGBL4-IT1,AGBL4,SMIM12,LRP8,USP24,TTC39A | N | 2.26E-06 |
| rs6088682 | circRNAs |  | Y | 2.27E-06 |
| rs2083180 | circRNAs,TADs | PBRM1,VPRBP | Y | 2.27E-06 |
| rs6120799 | circRNAs |  | Y | 2.27E-06 |
| rs12139515 | TADs,CIRs | RAB3B,AGBL4,ENSG00000221492,LRP8,FAF1 | N | 2.27E-06 |
| rs10860387 | circRNAs |  | N | 2.27E-06 |
| rs3746435 | CIRs | GGT7,MIR499A | Y | 2.27E-06 |
| rs2268027 | circRNAs,TADs,CIRs | NEK4 | Y | 2.27E-06 |
| rs1331623 | TADs | CDK5RAP2,ASTN2 | Y | 2.27E-06 |
| rs11078936 | circRNAs,TADs | ENSG00000174093,CDK12 | Y | 2.28E-06 |
| rs565298 | circRNAs,TADs | STARD9,RTF1 | N | 2.28E-06 |
| rs7800233 | circRNAs,lncRNAs |  | N | 2.28E-06 |
| rs11078931 | circRNAs,TADs | ENSG00000174093 | Y | 2.29E-06 |
| rs148910335 | TADs | STK31 | N | 2.29E-06 |
| rs62211622 | TADs,CIRs | CEP250,CNBD2,NCOA6 | Y | 2.29E-06 |
| rs4765668 | circRNAs |  | N | 2.29E-06 |
| rs12154475 | TADs | STK31 | N | 2.29E-06 |
| rs12154468 | TADs | STK31 | N | 2.29E-06 |
| rs743409 | circRNAs,CIRs | ENSG00000224086 | Y | 2.3E-06 |
| rs2102188 | TADs | EPS15,STIL,AGBL4-IT1,AGBL4,SMIM12,LRP8,USP24,TTC39A | N | 2.3E-06 |
| rs2372643 | circRNAs |  | N | 2.3E-06 |
| rs72896949 | TADs | RAB3B,AGBL4,LRP8,FAF1 | N | 2.31E-06 |
| rs7137868 | circRNAs |  | N | 2.31E-06 |
| rs10416125 | CIRs | AP1M2 | Y | 2.33E-06 |
| rs12488461 | circRNAs,TADs | PBRM1 | Y | 2.33E-06 |
| rs11231640 | CIRs | RCOR2 | N | 2.33E-06 |
| rs10401513 | CIRs | ILF3-AS1 | Y | 2.33E-06 |
| rs17826816 | circRNAs |  | N | 2.33E-06 |
| rs570098 | circRNAs,CIRs | STIP1 | Y | 2.34E-06 |
| rs11167253 | circRNAs |  | Y | 2.35E-06 |
| rs6538897 | circRNAs |  | N | 2.35E-06 |
| rs7693745 | circRNAs |  | N | 2.35E-06 |
| rs6859225 | circRNAs |  | N | 2.36E-06 |
| rs2635231 | TADs | ENSG00000236922 | N | 2.36E-06 |
| rs7979379 | circRNAs |  | N | 2.37E-06 |
| rs6120803 | circRNAs |  | Y | 2.37E-06 |
| rs10777953 | circRNAs |  | N | 2.37E-06 |
| rs1331622 | TADs | CDK5RAP2,ASTN2 | N | 2.37E-06 |
| rs10777954 | circRNAs |  | N | 2.37E-06 |
| rs10950456 | circRNAs |  | N | 2.38E-06 |
| rs758171 | circRNAs |  | N | 2.38E-06 |
| rs175386 | TADs | JUP,PCTP,FOXK2,CDC27,ENSG00000263317 | Y | 2.39E-06 |
| rs12754946 | circRNAs |  | Y | 2.39E-06 |
| rs475805 | circRNAs |  | N | 2.39E-06 |
| rs17106219 | circRNAs |  | N | 2.39E-06 |
| rs7965982 | circRNAs |  | Y | 2.4E-06 |
| rs4143327 | circRNAs |  | N | 2.4E-06 |
| rs12242642 | circRNAs,TADs | SORCS1,SHOC2 | N | 2.4E-06 |
| rs12065210 | circRNAs |  | N | 2.4E-06 |
| rs7250602 | circRNAs |  | Y | 2.41E-06 |
| rs6119548 | CIRs | MYH7B | Y | 2.42E-06 |
| rs7975384 | circRNAs |  | N | 2.42E-06 |
| rs3746436 | CIRs | MIR499A | Y | 2.43E-06 |
| rs4687549 | circRNAs,TADs | NEK4 | Y | 2.43E-06 |
| rs12742653 | circRNAs |  | N | 2.43E-06 |
| rs148262742 | TADs | FAF1 | N | 2.44E-06 |
| rs4980505 | circRNAs,CIRs | RNU6-1306P,MARK2 | N | 2.45E-06 |
| rs11097328 | TADs | GRID2,CCSER1 | N | 2.45E-06 |
| rs56005129 | circRNAs |  | N | 2.46E-06 |
| rs112879098 | circRNAs |  | N | 2.46E-06 |
| rs12496476 | circRNAs,TADs | PBRM1 | Y | 2.46E-06 |
| rs77732910 | circRNAs |  | N | 2.47E-06 |
| rs11440648 | CIRs | MAPK1 | Y | 2.47E-06 |
| rs239917 | circRNAs,CIRs | ENSG00000224086 | Y | 2.48E-06 |
| rs2905983 | CIRs | C17orf53 | Y | 2.48E-06 |
| rs2028216 | circRNAs,TADs | PBRM1 | Y | 2.48E-06 |
| rs10058613 | circRNAs |  | Y | 2.48E-06 |
| rs4842852 | TADs | SH3GL3,ENSG00000259986,ENSG00000166503,ADAMTS7P4 | Y | 2.49E-06 |
| rs4730065 | circRNAs |  | N | 2.5E-06 |
| rs12910381 | TADs | SH3GL3,ENSG00000259986,ENSG00000166503,ADAMTS7P4 | Y | 2.5E-06 |
| rs11231606 | circRNAs |  | N | 2.51E-06 |
| rs7221814 | TADs,CIRs | ENSG00000174093,FBXL20,CDK12,ORMDL3,ENSG00000264968 | Y | 2.51E-06 |
| rs2251165 | circRNAs |  | Y | 2.52E-06 |
| rs11719514 | circRNAs,TADs | PBRM1,VPRBP | Y | 2.52E-06 |
| rs11205718 | TADs | EPS15,STIL,AGBL4-IT1,AGBL4,SMIM12,LRP8,USP24,TTC39A | N | 2.52E-06 |
| rs6799185 | lncRNAs |  | Y | 2.52E-06 |
| rs261004 | circRNAs |  | N | 2.52E-06 |
| rs9916158 | circRNAs,TADs | ENSG00000174093,CDK12 | Y | 2.53E-06 |
| rs10804057 | TADs | STAT4 | N | 2.54E-06 |
| rs5003769 | circRNAs |  | N | 2.54E-06 |
| rs13072473 | lncRNAs |  | N | 2.54E-06 |
| rs7297221 | circRNAs |  | Y | 2.54E-06 |
| rs9972916 | TADs | JUP,PCTP,FOXK2,CDC27,ENSG00000263317 | Y | 2.55E-06 |
| rs227580 | circRNAs |  | Y | 2.55E-06 |
| rs10791860 | circRNAs |  | Y | 2.55E-06 |
| rs3862007 | circRNAs,TFBRs | RN7SL450P | N | 2.56E-06 |
| rs12145113 | TADs | EPS15,STIL,AGBL4-IT1,AGBL4,SMIM12,LRP8,USP24,TTC39A | N | 2.56E-06 |
| rs9549695 | circRNAs |  | Y | 2.56E-06 |
| rs17716499 | CIRs | DIO3,DIO3OS | N | 2.57E-06 |
| rs11639302 | TADs | SH3GL3,ENSG00000259986,ENSG00000166503,ADAMTS7P4 | Y | 2.58E-06 |
| rs8077456 | TADs | ENSG00000174093 | Y | 2.59E-06 |
| rs10818315 | TADs | CDK5RAP2 | N | 2.6E-06 |
| rs11078930 | circRNAs,TADs | ENSG00000174093 | Y | 2.6E-06 |
| rs6456099 | circRNAs |  | N | 2.61E-06 |
| rs34930574 | circRNAs |  | N | 2.61E-06 |
| rs7485825 | circRNAs |  | N | 2.61E-06 |
| rs9889715 | circRNAs,TADs | CA10,PLEKHM1,LRRC37A3 | Y | 2.62E-06 |
| rs752448 | circRNAs |  | Y | 2.62E-06 |
| rs175379 | TADs | JUP,PCTP,FOXK2,CDC27,ENSG00000263317 | Y | 2.62E-06 |
| rs956274 | TADs,lncRNAs | CAST,,ENSG00000251513,MCTP1 | N | 2.63E-06 |
| rs11062161 | circRNAs,lncRNAs |  | Y | 2.63E-06 |
| rs10415961 | CIRs | AP1M2 | Y | 2.63E-06 |
| rs884964 | circRNAs |  | N | 2.63E-06 |
| rs4842946 | TADs | SH3GL3,ENSG00000259986,ENSG00000166503,ADAMTS7P4 | Y | 2.63E-06 |
| rs7756763 | circRNAs |  | N | 2.63E-06 |
| rs484201 | circRNAs |  | N | 2.64E-06 |
| rs6088700 | circRNAs |  | Y | 2.64E-06 |
| rs6120821 | circRNAs |  | Y | 2.64E-06 |
| rs12422554 | circRNAs |  | N | 2.65E-06 |
| rs2372641 | circRNAs |  | N | 2.65E-06 |
| rs7964358 | circRNAs |  | N | 2.65E-06 |
| rs678 | TADs | VPRBP | Y | 2.65E-06 |
| rs227584 | circRNAs |  | Y | 2.67E-06 |
| rs112860674 | circRNAs,TADs | NEK4 | Y | 2.67E-06 |
| rs12096508 | circRNAs |  | N | 2.68E-06 |
| rs174551 | circRNAs,CIRs | FADS2 | Y | 2.68E-06 |
| rs9858939 | lncRNAs |  | Y | 2.68E-06 |
| rs4502182 | TADs,TFBRs | UBE2Q2P8,SH3GL3,ENSG00000259986,ENSG00000166503,ADAMTS7P4 | Y | 2.69E-06 |
| rs6087667 | circRNAs |  | Y | 2.69E-06 |
| rs12154473 | circRNAs |  | N | 2.69E-06 |
| rs6088694 | circRNAs |  | Y | 2.7E-06 |
| rs2653897 | TADs | EPS15,STIL,AGBL4-IT1,AGBL4,SMIM12,LRP8,USP24,TTC39A | N | 2.71E-06 |
| rs6120813 | circRNAs |  | Y | 2.71E-06 |
| rs6088691 | circRNAs |  | Y | 2.71E-06 |
| rs13245298 | circRNAs |  | N | 2.71E-06 |
| rs4842846 | TADs,TFBRs | UBE2Q2P8,SH3GL3,ENSG00000259986,ENSG00000166503,ADAMTS7P4 | Y | 2.71E-06 |
| rs6060030 | circRNAs |  | Y | 2.71E-06 |
| rs1560413 | circRNAs |  | Y | 2.71E-06 |
| rs74779340 | TADs | STK31 | N | 2.72E-06 |
| rs227583 | circRNAs,CIRs | LSM12 | Y | 2.72E-06 |
| rs261043 | circRNAs |  | N | 2.72E-06 |
| rs17231202 | circRNAs |  | N | 2.72E-06 |
| rs2966419 | TADs | IMMP2L | N | 2.73E-06 |
| rs11134596 | circRNAs |  | N | 2.74E-06 |
| rs6120772 | CIRs | NCOA6 | Y | 2.75E-06 |
| rs7759578 | circRNAs |  | N | 2.75E-06 |
| rs6088690 | circRNAs |  | Y | 2.76E-06 |
| rs2246556 | circRNAs |  | Y | 2.77E-06 |
| rs10399964 | circRNAs,TADs | RBM20,SHOC2,SORCS1,CCDC147,MXI1,XPNPEP1,ENSG00000223381,NRAP,ENSG00000229981,GUCY2GP,HELLS,INPP5A | N | 2.77E-06 |
| rs4794218 | circRNAs,TADs | CA10,PLEKHM1,LRRC37A3 | Y | 2.77E-06 |
| rs34342173 | TADs,lncRNAs | ENSG00000174093,CDK12 | Y | 2.77E-06 |
| rs34933056 | TADs | ENSG00000174093,CDK12 | Y | 2.77E-06 |
| rs17319032 | circRNAs |  | Y | 2.78E-06 |
| rs6087666 | circRNAs |  | Y | 2.78E-06 |
| rs185054241 | TADs,CIRs,TFBRs | DBNL,SEC24B,ANK2,SEC24B-AS1,RPL34,PLA2G12A,CENPE,ENSG00000249815,EGF | N | 2.78E-06 |
| rs6120812 | circRNAs |  | Y | 2.78E-06 |
| rs7067604 | circRNAs,TADs | SORCS1,SHOC2 | Y | 2.79E-06 |
| rs10875913 | circRNAs |  | Y | 2.8E-06 |
| rs2246287 | circRNAs,CIRs | ENSG00000267638 | Y | 2.8E-06 |
| rs6928829 | circRNAs |  | N | 2.8E-06 |
| rs10747559 | circRNAs |  | Y | 2.81E-06 |
| rs8136867 | circRNAs,CIRs | ENSG00000224086 | Y | 2.81E-06 |
| rs7210107 | circRNAs,TADs | JUP,PCTP,FOXK2,CDC27,ENSG00000263317 | Y | 2.82E-06 |
| rs10230128 | circRNAs,CIRs | KMT2E,,LINC01004 | N | 2.82E-06 |
| rs4499200 | TADs | SH3GL3,ENSG00000259986,ENSG00000166503,ADAMTS7P4 | Y | 2.83E-06 |
| rs11237799 | circRNAs |  | Y | 2.84E-06 |
| rs11589380 | TADs | EPS15,STIL,AGBL4-IT1,AGBL4,SMIM12,LRP8,USP24,TTC39A | N | 2.84E-06 |
| rs8079310 | circRNAs,lncRNAs |  | Y | 2.84E-06 |
| rs11652139 | circRNAs,TADs | ENSG00000174093 | Y | 2.84E-06 |
| rs1004936 | circRNAs |  | N | 2.84E-06 |
| rs6493054 | circRNAs,TADs | STARD9,RTF1 | N | 2.85E-06 |
| rs6088703 | circRNAs |  | Y | 2.86E-06 |
| rs4337266 | TADs | ENSG00000259986,ENSG00000166503,ADAMTS7P4 | Y | 2.86E-06 |
| rs7932890 | circRNAs |  | Y | 2.87E-06 |
| rs6767095 | CIRs | ATP1B3 | Y | 2.87E-06 |
| rs4683645 | circRNAs |  | Y | 2.87E-06 |
| rs62028133 | TADs | ENSG00000259986,ENSG00000166503,ADAMTS7P4 | Y | 2.88E-06 |
| rs174597 | circRNAs |  | Y | 2.89E-06 |
| rs1560412 | circRNAs |  | Y | 2.89E-06 |
| rs11168839 | CIRs,lncRNAs | ENSG00000257346 | Y | 2.89E-06 |
| rs1705360 | circRNAs,TADs | STARD9,RTF1 | N | 2.91E-06 |
| rs6960603 | TADs,CIRs,TFBRs | MRPS33,DENND2A,TMEM178B | Y | 2.91E-06 |
| rs2843728 | circRNAs |  | Y | 2.92E-06 |
| rs60527016 | circRNAs |  | Y | 2.92E-06 |
| rs41356645 | circRNAs |  | N | 2.93E-06 |
| rs13087772 | circRNAs,TADs | NEK4 | Y | 2.93E-06 |
| rs11231624 | circRNAs |  | N | 2.94E-06 |
| rs2965187 | circRNAs |  | Y | 2.94E-06 |
| rs2425004 | TADs | CEP250,CNBD2 | Y | 2.95E-06 |
| rs1352574 | TADs | ENSG00000236922 | N | 2.95E-06 |
| rs9926912 | circRNAs |  | N | 2.96E-06 |
| rs2896002 | TADs | SH3GL3,ENSG00000259986,ENSG00000166503,ADAMTS7P4 | Y | 2.96E-06 |
| rs4765667 | circRNAs |  | N | 2.97E-06 |
| rs6575858 | CIRs | DIO3,DIO3OS | N | 2.97E-06 |
| rs11130321 | circRNAs,TADs | NEK4 | Y | 2.98E-06 |
| rs34568744 | circRNAs,CIRs | PCID2 | Y | 2.98E-06 |
| rs3774354 | TADs | VPRBP | Y | 2.98E-06 |
| rs9859932 | circRNAs |  | Y | 2.98E-06 |
| rs10432705 | TADs | PLB1 | N | 2.99E-06 |
| rs11671684 | TADs,CIRs | ZNF208,ATP13A1,YJEFN3,ENSG00000267383,ENSG00000258674,ENSG00000141979 | N | 2.99E-06 |
| rs12916348 | circRNAs |  | Y | 2.99E-06 |
| rs9886312 | CIRs | MRPS33,BRAF | Y | 3E-06 |
| rs722219 | TADs,CIRs | DENND2A,TMEM178B | Y | 0.000003 |
| rs1127894 | CIRs,TFBRs | RBM4,RNU4-39P,RBM14,RBM14-RBM4,CTSF | Y | 3E-06 |
| rs34453912 | circRNAs,TADs | ENSG00000174093,CDK12 | Y | 3E-06 |
| rs3787223 | circRNAs |  | Y | 3E-06 |
| rs2370414 | circRNAs |  | Y | 3E-06 |
| rs55943631 | circRNAs |  | N | 3.01E-06 |
| rs1349348 | circRNAs |  | Y | 3.03E-06 |
| rs1848093 | CIRs | ENSG00000259609 | Y | 3.04E-06 |
| rs2653889 | TADs | RAB3B,AGBL4,LRP8,FAF1 | N | 3.04E-06 |
| rs2268023 | TADs | VPRBP | Y | 3.04E-06 |
| rs2227321 | TADs,TFBRs | CSF3,ENSG00000174093,ENSG00000265799,CDK12 | Y | 3.06E-06 |
| rs8070746 | circRNAs,TADs | CA10,PLEKHM1,LRRC37A3 | Y | 3.06E-06 |
| rs4599515 | circRNAs |  | N | 3.07E-06 |
| rs2306899 | circRNAs,CIRs | WHSC1L1 | Y | 3.07E-06 |
| rs2048793 | circRNAs |  | N | 3.08E-06 |
| rs9788038 | circRNAs |  | N | 3.08E-06 |
| rs2302777 | circRNAs,TADs | ENSG00000174093,CDK12 | Y | 3.08E-06 |
| rs58574309 | circRNAs |  | N | 3.09E-06 |
| rs4842853 | TADs | SH3GL3,ENSG00000259986,ENSG00000166503,ADAMTS7P4 | Y | 3.09E-06 |
| rs150961 | circRNAs |  | Y | 3.1E-06 |
| rs12423277 | circRNAs |  | Y | 3.11E-06 |
| rs12453341 | circRNAs,TADs | ENSG00000174093,CDK12 | Y | 3.11E-06 |
| rs3746446 | CIRs | TRPC4AP | Y | 3.11E-06 |
| rs11878202 | circRNAs |  | N | 3.12E-06 |
| rs2115937 | circRNAs |  | Y | 3.12E-06 |
| rs3782357 | circRNAs |  | Y | 3.12E-06 |
| rs10178015 | lncRNAs |  | N | 3.13E-06 |
| rs1489545 | TADs | ENSG00000236922 | Y | 3.13E-06 |
| rs571171 | circRNAs |  | N | 3.15E-06 |
| rs7727697 | circRNAs |  | N | 3.16E-06 |
| rs8113682 | circRNAs |  | Y | 3.16E-06 |
| rs12522444 | circRNAs |  | N | 3.16E-06 |
| rs4571799 | TADs | CDK5RAP2 | Y | 3.17E-06 |
| rs11696652 | circRNAs |  | Y | 3.17E-06 |
| rs1260326 | circRNAs,lncRNAs |  | Y | 3.18E-06 |
| rs7191614 | TADs | HYDIN,SMPD3 | Y | 3.18E-06 |
| rs75849460 | circRNAs |  | N | 3.18E-06 |
| rs6790923 | circRNAs |  | Y | 3.19E-06 |
| rs11767331 | TADs | DENND2A | Y | 3.19E-06 |
| rs143455831 | circRNAs,TADs | CAST,ENSG00000251513,ENSG00000251314,ENSG00000249776,GPR98,FAM172A,MCTP1 | N | 3.19E-06 |
| rs6060043 | circRNAs,TADs | FER1L4,NCOA6,ASIP | Y | 3.2E-06 |
| rs12866090 | circRNAs |  | Y | 3.2E-06 |
| rs9549696 | circRNAs |  | Y | 3.2E-06 |
| rs11630887 | circRNAs |  | Y | 3.2E-06 |
| rs3760570 | circRNAs |  | Y | 3.2E-06 |
| rs72906810 | TADs | FAF1 | N | 3.2E-06 |
| rs62202937 | circRNAs |  | N | 3.21E-06 |
| rs4726263 | TADs | DENND2A | Y | 3.21E-06 |
| rs34648856 | circRNAs,TADs | ENSG00000174093 | Y | 3.21E-06 |
| rs185310 | circRNAs |  | Y | 3.22E-06 |
| rs2721793 | circRNAs,TADs | DFNA5,CCDC126 | Y | 3.22E-06 |
| rs12451897 | circRNAs,TADs | ENSG00000174093,CDK12 | Y | 3.23E-06 |
| rs2635341 | TADs | ENSG00000236922 | N | 3.23E-06 |
| rs2372727 | circRNAs |  | N | 3.24E-06 |
| rs2635186 | TADs | ENSG00000236922 | N | 3.24E-06 |
| rs6504702 | circRNAs,TADs | CA10,PLEKHM1,LRRC37A3 | Y | 3.25E-06 |
| rs1948254 | TADs | ENSG00000236922 | N | 3.25E-06 |
| rs10043984 | circRNAs |  | N | 3.25E-06 |
| rs1072982 | circRNAs |  | Y | 3.25E-06 |
| rs6444424 | circRNAs |  | N | 3.26E-06 |
| rs6944492 | TADs | DENND2A | Y | 3.26E-06 |
| rs77034359 | circRNAs |  | Y | 3.26E-06 |
| rs1054442 | CIRs,TFBRs | WNT1,WNT10B,ENSG00000258283,ENSG00000272822,RNU6-940P,DDX23,ARF3 | Y | 3.27E-06 |
| rs3744245 | circRNAs,TADs | ENSG00000174093 | Y | 3.27E-06 |
| rs2840079 | CIRs | C11orf95,OTUB1,ENSG00000255651 | Y | 3.28E-06 |
| rs9940694 | circRNAs |  | N | 3.28E-06 |
| rs55882341 | circRNAs |  | N | 3.28E-06 |
| rs2273738 | circRNAs,TADs | RBM20,SHOC2,SORCS1,CCDC147,MXI1,XPNPEP1,ENSG00000223381,NRAP,ENSG00000229981,GUCY2GP,HELLS,INPP5A | N | 3.28E-06 |
| rs3774355 | TADs | VPRBP | Y | 3.29E-06 |
| rs1381168 | circRNAs |  | N | 3.3E-06 |
| rs192312486 | circRNAs |  | N | 3.31E-06 |
| rs28786717 | circRNAs,CIRs | CDC25C | N | 3.31E-06 |
| rs6503490 | CIRs | ASB16 | Y | 3.31E-06 |
| rs76451960 | circRNAs |  | Y | 3.32E-06 |
| rs898727 | circRNAs |  | N | 3.32E-06 |
| rs12094280 | circRNAs |  | N | 3.32E-06 |
| rs11207639 | circRNAs |  | N | 3.32E-06 |
| rs17589320 | TADs | ENSG00000259986,ENSG00000166503,ADAMTS7P4 | Y | 3.32E-06 |
| rs2363079 | circRNAs,lncRNAs |  | N | 3.32E-06 |
| rs10783301 | circRNAs,CIRs | KMT2D | Y | 3.32E-06 |
| rs2425003 | TADs | CEP250,CNBD2 | Y | 3.32E-06 |
| rs56012312 | lncRNAs |  | N | 3.33E-06 |
| rs2227319 | TADs,TFBRs,lncRNAs | CSF3,,ENSG00000174093,ENSG00000265799,CDK12 | Y | 3.33E-06 |
| rs2372632 | circRNAs |  | N | 3.33E-06 |
| rs411348 | circRNAs |  | N | 3.33E-06 |
| rs10848626 | circRNAs |  | N | 3.35E-06 |
| rs4911160 | circRNAs |  | Y | 3.35E-06 |
| rs10848625 | circRNAs |  | N | 3.35E-06 |
| rs62202938 | circRNAs |  | N | 3.36E-06 |
| rs12911596 | TADs | SH3GL3,ENSG00000259986,ENSG00000166503,ADAMTS7P4 | Y | 3.37E-06 |
| rs2969622 | TADs | ENSG00000236922 | N | 3.37E-06 |
| rs1948246 | TADs | ENSG00000236922 | Y | 3.37E-06 |
| rs12668848 | circRNAs |  | N | 3.38E-06 |
| rs35440248 | circRNAs |  | N | 3.38E-06 |
| rs2635326 | TADs | ENSG00000236922 | N | 3.38E-06 |
| rs76246075 | circRNAs |  | N | 3.39E-06 |
| rs35306827 | circRNAs |  | Y | 3.39E-06 |
| rs10736731 | circRNAs,miRNA target site | MARK2 | Y | 3.39E-06 |
| rs7253821 | TADs | ZNF208,ENSG00000267383,ENSG00000141979 | Y | 3.39E-06 |
| rs12637632 | circRNAs,TADs | PBRM1 | Y | 3.4E-06 |
| rs910872 | circRNAs |  | Y | 3.41E-06 |
| rs175381 | TADs | JUP,PCTP,FOXK2,CDC27,ENSG00000263317 | Y | 3.41E-06 |
| rs7554367 | CIRs | OTUD7B | Y | 3.41E-06 |
| rs5999750 | circRNAs |  | Y | 3.42E-06 |
| rs35131895 | circRNAs |  | N | 3.43E-06 |
| rs228768 | circRNAs,TADs,CIRs | HDAC5,ENSG00000267638 | Y | 3.43E-06 |
| rs71570522 | TADs | HCRTR2,PKHD1,PRIM2 | N | 3.43E-06 |
| rs12944572 | CIRs | C17orf53 | Y | 3.44E-06 |
| rs10994384 | circRNAs |  | N | 3.44E-06 |
| rs727605 | circRNAs |  | N | 3.44E-06 |
| rs7932432 | circRNAs |  | N | 3.45E-06 |
| rs9934669 | circRNAs |  | N | 3.45E-06 |
| rs882193 | circRNAs |  | Y | 3.46E-06 |
| rs113028610 | circRNAs |  | N | 3.46E-06 |
| rs1007893 | CIRs,TFBRs,lncRNAs | ENSG00000267416,,MCM3AP,C21orf58,DIP2A,RAN | Y | 3.46E-06 |
| rs1986224 | TADs | ENSG00000236922 | N | 3.48E-06 |
| rs73616608 | TADs,CIRs | CEP250,CNBD2,NCOA6 | N | 3.48E-06 |
| rs1948252 | TADs | ENSG00000236922 | N | 3.49E-06 |
| rs7199375 | circRNAs |  | N | 3.49E-06 |
| rs2159908 | circRNAs |  | N | 3.5E-06 |
| rs2159909 | circRNAs |  | N | 3.5E-06 |
| rs4606311 | TADs,CIRs | SLAMF9,ENSG00000228560,AIM2 | N | 3.5E-06 |
| rs2969623 | TADs | ENSG00000236922 | N | 3.51E-06 |
| rs9549359 | circRNAs |  | Y | 3.51E-06 |
| rs28821001 | circRNAs |  | Y | 3.51E-06 |
| rs6060034 | circRNAs |  | Y | 3.51E-06 |
| rs10896100 | circRNAs |  | Y | 3.51E-06 |
| rs7271289 | circRNAs,TADs,CIRs | FER1L4,NCOA6,ASIP | Y | 3.51E-06 |
| rs910871 | circRNAs |  | Y | 3.52E-06 |
| rs703987 | circRNAs |  | N | 3.52E-06 |
| rs12916198 | TADs | SH3GL3,ENSG00000259986,ENSG00000166503,ADAMTS7P4 | Y | 3.53E-06 |
| rs12669532 | circRNAs |  | N | 3.54E-06 |
| rs2270401 | circRNAs,TADs,lncRNAs | ENSG00000174093,CDK12 | Y | 3.54E-06 |
| rs10875915 | circRNAs,CIRs | PRKAG1 | Y | 3.54E-06 |
| rs3803398 | TADs | SH3GL3,ENSG00000259986,ENSG00000166503,ADAMTS7P4 | Y | 3.55E-06 |
| rs6088603 | circRNAs,TADs | FER1L4,NCOA6,ASIP | Y | 3.56E-06 |
| rs10888713 | TADs | FAF1 | N | 3.56E-06 |
| rs34341238 | circRNAs,TADs | PBRM1,VPRBP | Y | 3.57E-06 |
| rs4072639 | circRNAs,TADs | ENSG00000174093,CDK12 | Y | 3.57E-06 |
| rs11631921 | CIRs,TFBRs | ZNF592,ZSCAN2 | Y | 3.57E-06 |
| rs12576275 | circRNAs |  | N | 3.58E-06 |
| rs6058117 | circRNAs,TADs | FER1L4,NCOA6,ASIP | Y | 3.58E-06 |
| rs1865065 | circRNAs |  | Y | 3.6E-06 |
| rs11867302 | circRNAs,TADs | ENSG00000174093 | Y | 3.6E-06 |
| rs7220138 | circRNAs |  | Y | 3.6E-06 |
| rs6440047 | circRNAs |  | Y | 3.6E-06 |
| rs1533126 | circRNAs |  | N | 3.6E-06 |
| rs2236989 | circRNAs,TADs | CADPS,ZDHHC3,CACNA2D2 | Y | 3.61E-06 |
| rs6542199 | TADs | DPP10 | N | 3.61E-06 |
| rs1860056 | circRNAs |  | N | 3.62E-06 |
| rs16856991 | circRNAs,CIRs | CCDC173 | Y | 3.62E-06 |
| rs4338765 | TADs | ENSG00000259986,ENSG00000166503,ADAMTS7P4 | Y | 3.63E-06 |
| rs7639052 | circRNAs |  | Y | 3.63E-06 |
| rs6503488 | CIRs | MPP2,C17orf53,ASB16 | Y | 3.63E-06 |
| rs2012704 | circRNAs |  | N | 3.63E-06 |
| rs11227439 | CIRs,TFBRs | ENSG00000254458,RAB1B,KLC2,ENSG00000254762 | Y | 3.64E-06 |
| rs11624408 | circRNAs |  | N | 3.64E-06 |
| rs12621972 | circRNAs |  | Y | 3.64E-06 |
| rs6773700 | circRNAs |  | Y | 3.64E-06 |
| rs537001 | circRNAs,CIRs | STIP1 | N | 3.65E-06 |
| rs3935281 | circRNAs,TADs | ENSG00000174093,CDK12 | Y | 3.65E-06 |
| rs111498086 | TADs | STK31 | N | 3.67E-06 |
| rs9549367 | circRNAs |  | Y | 3.68E-06 |
| rs17300292 | circRNAs |  | Y | 3.68E-06 |
| rs35474552 | TADs | SH3GL3,ENSG00000259986,ENSG00000166503,ADAMTS7P4 | Y | 3.7E-06 |
| rs2370413 | circRNAs |  | Y | 3.7E-06 |
| rs6142237 | circRNAs |  | Y | 3.71E-06 |
| rs57331401 | TADs | FAF1 | N | 3.72E-06 |
| rs3743669 | circRNAs,TADs | HYDIN,SMPD3 | Y | 3.73E-06 |
| rs2969620 | TADs | ENSG00000236922 | N | 3.73E-06 |
| rs5756052 | CIRs | MAPK1 | Y | 3.74E-06 |
| rs66826786 | circRNAs,TADs | JUP,ACACA | Y | 3.75E-06 |
| rs62029586 | TADs | SH3GL3,ENSG00000259986,ENSG00000166503,ADAMTS7P4 | Y | 3.75E-06 |
| rs261006 | circRNAs |  | Y | 3.75E-06 |
| rs491554 | TADs | DENND2A | Y | 3.76E-06 |
| rs11662248 | circRNAs |  | N | 3.76E-06 |
| rs7210007 | lncRNAs |  | N | 3.76E-06 |
| rs57131731 | circRNAs,TADs | FER1L4,NCOA6,ASIP | Y | 3.77E-06 |
| rs3994541 | circRNAs |  | Y | 3.78E-06 |
| rs9939870 | circRNAs,TADs | HYDIN,SMPD3 | Y | 3.78E-06 |
| rs61881586 | circRNAs,TADs | SORCS1,SHOC2 | N | 3.78E-06 |
| rs3787220 | circRNAs |  | Y | 3.78E-06 |
| rs12122800 | CIRs | ASH1L | Y | 3.78E-06 |
| rs62211612 | circRNAs,TADs | FER1L4,NCOA6,ASIP | Y | 3.79E-06 |
| rs6058115 | circRNAs |  | Y | 3.79E-06 |
| rs1874252 | TADs,CIRs,TFBRs | PATL2,ENSG00000259747,GPR176,WDR76 | N | 3.79E-06 |
| rs35677834 | TADs | SH3GL3,ENSG00000259986,ENSG00000166503,ADAMTS7P4 | Y | 3.8E-06 |
| rs3861723 | circRNAs |  | Y | 3.8E-06 |
| rs56298082 | circRNAs |  | N | 3.81E-06 |
| rs255375 | circRNAs |  | Y | 3.81E-06 |
| rs2653907 | TADs | EPS15,STIL,AGBL4-IT1,AGBL4,SMIM12,LRP8,USP24,TTC39A | Y | 3.82E-06 |
| rs4417778 | circRNAs |  | Y | 3.82E-06 |
| rs2424998 | circRNAs,TADs,CIRs | FER1L4,NCOA6,ASIP | Y | 3.82E-06 |
| rs4302281 | circRNAs |  | Y | 3.82E-06 |
| rs11784833 | CIRs,TFBRs | GRINA,FBXL6,BOP1 | Y | 3.82E-06 |
| rs55739615 | TADs | ENSG00000174093,FBXL20,CDK12 | Y | 3.83E-06 |
| rs41499647 | circRNAs |  | Y | 3.83E-06 |
| rs7338235 | circRNAs |  | Y | 3.84E-06 |
| rs7207464 | CIRs | C17orf53 | Y | 3.84E-06 |
| rs12570607 | TADs | ENSG00000223381,ENSG00000229981,SORCS1,CCDC147 | N | 3.84E-06 |
| rs150967 | circRNAs |  | Y | 3.85E-06 |
| rs12906983 | TADs | SH3GL3,ENSG00000259986,ENSG00000166503,ADAMTS7P4 | Y | 3.85E-06 |
| rs2635190 | TADs | ENSG00000236922 | N | 3.86E-06 |
| rs353547 | circRNAs,CIRs | TWF2,TLR9 | Y | 3.86E-06 |
| rs10942275 | circRNAs |  | N | 3.86E-06 |
| rs7089755 | circRNAs,TADs | RSU1,ST8SIA6 | N | 3.86E-06 |
| rs7613107 | circRNAs |  | Y | 3.86E-06 |
| rs62433108 | circRNAs |  | Y | 3.86E-06 |
| rs4473241 | CIRs | GRN | Y | 3.87E-06 |
| rs13076111 | CIRs,TFBRs | ATP1B3 | Y | 3.87E-06 |
| rs2635230 | TADs | ENSG00000236922 | N | 3.87E-06 |
| rs13384219 | CIRs | VRK2 | N | 3.88E-06 |
| rs13014440 | TADs | DPP10 | N | 3.88E-06 |
| rs509556 | CIRs | ACTN3 | Y | 3.88E-06 |
| rs3785073 | circRNAs,CIRs | MIR1538,COG8 | Y | 3.88E-06 |
| rs33369 | circRNAs |  | N | 3.88E-06 |
| rs6120826 | circRNAs |  | Y | 3.88E-06 |
| rs630021 | CIRs,TFBRs | CTSF | Y | 3.9E-06 |
| rs2635335 | TADs | ENSG00000236922 | N | 3.9E-06 |
| rs498933 | TADs | DENND2A | Y | 3.91E-06 |
| rs11587399 | TADs | EPS15,STIL,AGBL4-IT1,AGBL4,SMIM12,LRP8,USP24,TTC39A | N | 3.91E-06 |
| rs12279388 | circRNAs |  | N | 3.92E-06 |
| rs6584970 | circRNAs |  | Y | 3.92E-06 |
| rs4656248 | TADs | ENSG00000228560,AIM2 | N | 3.92E-06 |
| rs3829643 | circRNAs,CIRs | CNNM4,MIR4754 | N | 3.92E-06 |
| rs7306078 | circRNAs |  | Y | 3.93E-06 |
| rs11762357 | circRNAs,TADs | TMEM178B | Y | 3.93E-06 |
| rs11631947 | circRNAs |  | Y | 3.95E-06 |
| rs2246535 | TADs | ENSG00000236922 | N | 3.95E-06 |
| rs75263403 | circRNAs |  | N | 3.96E-06 |
| rs4762547 | circRNAs |  | N | 3.96E-06 |
| rs35124791 | circRNAs,CIRs | MIR1538 | Y | 3.96E-06 |
| rs3760569 | circRNAs |  | N | 3.96E-06 |
| rs78849264 | TADs | DFNA5,CCDC126 | N | 3.96E-06 |
| rs12056698 | circRNAs |  | N | 3.97E-06 |
| rs1860752 | circRNAs,TADs | TMEM178B | Y | 3.97E-06 |
| rs10512928 | circRNAs |  | N | 3.98E-06 |
| rs3902025 | TADs,CIRs,TFBRs | ENSG00000174093,GSDMA,FBXL20,CDK12 | Y | 3.98E-06 |
| rs10848624 | circRNAs |  | N | 3.99E-06 |
| rs16887277 | circRNAs |  | Y | 3.99E-06 |
| rs17126936 | circRNAs,TADs | SORCS1,SHOC2 | N | 4E-06 |
| rs2237607 | circRNAs |  | N | 4E-06 |
| rs703968 | circRNAs |  | N | 0.000004 |
| rs1572358 | circRNAs |  | N | 4E-06 |
| rs6120825 | circRNAs |  | Y | 4E-06 |
| rs2372726 | circRNAs |  | N | 4.01E-06 |
| rs75063896 | circRNAs,TADs | SORCS1,SHOC2 | N | 4.02E-06 |
| rs35501888 | circRNAs |  | N | 4.02E-06 |
| rs2635333 | TADs | ENSG00000236922 | N | 4.03E-06 |
| rs7089 | circRNAs |  | Y | 4.04E-06 |
| rs1572359 | circRNAs |  | N | 4.06E-06 |
| rs7187984 | circRNAs |  | N | 4.07E-06 |
| rs73404723 | circRNAs,CIRs | ZNF106 | N | 4.07E-06 |
| rs7816998 | circRNAs |  | Y | 4.07E-06 |
| rs4146115 | CIRs | ENSG00000204929 | N | 4.09E-06 |
| rs9549705 | circRNAs |  | Y | 4.09E-06 |
| rs1032292 | lncRNAs |  | Y | 4.09E-06 |
| rs9939815 | circRNAs |  | N | 4.09E-06 |
| rs2339519 | TADs | KLHL29,ENSG00000231200,ENSG00000234207,ATAD2B | N | 4.1E-06 |
| rs6452410 | circRNAs |  | N | 4.1E-06 |
| rs7749359 | circRNAs |  | N | 4.1E-06 |
| rs481902 | circRNAs |  | N | 4.1E-06 |
| rs16887273 | circRNAs |  | Y | 4.1E-06 |
| rs35156560 | TADs | SH3GL3,ENSG00000259986,ENSG00000166503,ADAMTS7P4 | Y | 4.11E-06 |
| rs6954854 | circRNAs,TADs | SP4 | N | 4.11E-06 |
| rs25645 | TADs | ENSG00000174093,CDK12 | Y | 4.11E-06 |
| rs748179 | circRNAs |  | N | 4.11E-06 |
| rs9939817 | circRNAs |  | N | 4.11E-06 |
| rs55666830 | circRNAs |  | N | 4.13E-06 |
| rs9938300 | circRNAs |  | N | 4.13E-06 |
| rs10445310 | circRNAs,TADs | JUP,ACACA | Y | 4.14E-06 |
| rs2784159 | TADs | EPS15,STIL,AGBL4-IT1,AGBL4,SMIM12,LRP8,USP24,TTC39A | N | 4.15E-06 |
| rs703973 | circRNAs |  | N | 4.15E-06 |
| rs2784143 | TADs | EPS15,STIL,AGBL4-IT1,AGBL4,SMIM12,LRP8,USP24,TTC39A | N | 4.16E-06 |
| rs6088594 | circRNAs |  | Y | 4.16E-06 |
| rs12453343 | circRNAs,TADs | ENSG00000174093,CDK12 | Y | 4.16E-06 |
| rs11777067 | circRNAs |  | Y | 4.16E-06 |
| rs1539738 | circRNAs |  | N | 4.17E-06 |
| rs34382810 | circRNAs |  | N | 4.17E-06 |
| rs228769 | circRNAs,TADs,CIRs,TFBRs | HDAC5,ENSG00000267638 | Y | 4.17E-06 |
| rs7794979 | TADs | SSBP1,DENND2A | Y | 4.17E-06 |
| rs71494212 | circRNAs |  | N | 4.17E-06 |
| rs7217858 | circRNAs |  | Y | 4.17E-06 |
| rs11557347 | circRNAs |  | N | 4.18E-06 |
| rs553046 | TADs | DENND2A | Y | 4.18E-06 |
| rs12451547 | circRNAs,TADs | ENSG00000174093,CDK12 | Y | 4.19E-06 |
| rs16842765 | TADs | ENSG00000234281 | N | 4.19E-06 |
| rs4730071 | circRNAs |  | N | 4.21E-06 |
| rs10750781 | circRNAs,CIRs,TFBRs | ENSG00000255468,CCDC85B,ENSG00000254762 | Y | 4.21E-06 |
| rs6461049 | circRNAs |  | Y | 4.23E-06 |
| rs7196023 | circRNAs |  | N | 4.24E-06 |
| rs4842847 | TADs | SH3GL3,ENSG00000259986,ENSG00000166503,ADAMTS7P4 | Y | 4.24E-06 |
| rs13330072 | circRNAs,CIRs | COG8 | Y | 4.25E-06 |
| rs16966529 | circRNAs |  | N | 4.26E-06 |
| rs3756766 | CIRs,TFBRs | EGR1,KDM3B,CDC25C | N | 4.27E-06 |
| rs6584964 | circRNAs,TADs | SORCS1,SHOC2 | N | 4.27E-06 |
| rs17106235 | circRNAs |  | N | 4.27E-06 |
| rs6000070 | CIRs | MAPK1 | Y | 4.28E-06 |
| rs4845399 | CIRs | UBE2Q1 | Y | 4.28E-06 |
| rs12946632 | circRNAs |  | Y | 4.28E-06 |
| rs721769 | circRNAs |  | Y | 4.29E-06 |
| rs56058270 | circRNAs |  | Y | 4.3E-06 |
| rs8053736 | circRNAs,CIRs | PDF | Y | 4.3E-06 |
| rs34842775 | TADs | ENSG00000234281 | N | 4.3E-06 |
| rs2635241 | TADs | ENSG00000236922 | N | 4.3E-06 |
| rs2227333 | TADs | ENSG00000174093,CDK12 | Y | 4.31E-06 |
| rs62029599 | TADs | SH3GL3,ENSG00000259986,ENSG00000166503,ADAMTS7P4 | Y | 4.31E-06 |
| rs7189901 | circRNAs |  | N | 4.31E-06 |
| rs7811349 | lncRNAs |  | N | 4.31E-06 |
| rs9549698 | circRNAs |  | Y | 4.32E-06 |
| rs61554907 | TADs | JUP,ACACA | N | 4.32E-06 |
| rs12903946 | TADs | SH3GL3,ENSG00000259986,ENSG00000166503,ADAMTS7P4 | Y | 4.32E-06 |
| rs56236407 | TADs | ZNF208,ENSG00000267383,ENSG00000141979 | N | 4.34E-06 |
| rs740297 | circRNAs |  | N | 4.34E-06 |
| rs12493107 | circRNAs,TADs | PBRM1 | Y | 4.35E-06 |
| rs9914973 | TADs | ENSG00000174093 | Y | 4.35E-06 |
| rs1211642 | circRNAs,TADs | STARD9,RTF1 | N | 4.35E-06 |
| rs35082360 | circRNAs |  | N | 4.36E-06 |
| rs13411201 | TADs | WDPCP,PNPT1,PUS10,ENSG00000233891,EHBP1,ENSG00000204929,COMMD1,LINC01122,ENSG00000271955,CTNNA2,C1D | N | 4.36E-06 |
| rs4691753 | circRNAs |  | N | 4.36E-06 |
| rs11194950 | circRNAs |  | N | 4.38E-06 |
| rs2721794 | circRNAs,TADs | DFNA5,CCDC126 | Y | 4.38E-06 |
| rs12325060 | circRNAs,CIRs | COG8 | Y | 4.38E-06 |
| rs9459708 | circRNAs |  | N | 4.38E-06 |
| rs352162 | CIRs,lncRNAs | TWF2,ENSG00000173366,TLR9 | Y | 4.39E-06 |
| rs6538894 | circRNAs |  | N | 4.39E-06 |
| rs2000939 | CIRs | ACTN3 | Y | 4.39E-06 |
| rs7090570 | circRNAs |  | N | 4.39E-06 |
| rs6060017 | circRNAs |  | Y | 4.4E-06 |
| rs10818318 | TADs | CDK5RAP2 | Y | 4.41E-06 |
| rs62029593 | TADs | SH3GL3,ENSG00000259986,ENSG00000166503,ADAMTS7P4 | Y | 4.41E-06 |
| rs62029594 | TADs | SH3GL3,ENSG00000259986,ENSG00000166503,ADAMTS7P4 | Y | 4.41E-06 |
| rs10113411 | circRNAs |  | Y | 4.43E-06 |
| rs7340228 | TADs | DPP10 | N | 4.43E-06 |
| rs59705032 | circRNAs,CIRs | STARD9 | N | 4.45E-06 |
| rs10492859 | circRNAs |  | Y | 4.45E-06 |
| rs703971 | circRNAs |  | N | 4.46E-06 |
| rs4835680 | circRNAs |  | N | 4.46E-06 |
| rs6120819 | circRNAs |  | Y | 4.47E-06 |
| rs2237604 | circRNAs,CIRs | ENSG00000272918 | N | 4.48E-06 |
| rs2287248 | circRNAs |  | Y | 4.48E-06 |
| rs3794422 | circRNAs |  | Y | 4.48E-06 |
| rs7180483 | circRNAs |  | N | 4.48E-06 |
| rs677488 | CIRs | ACTN3 | Y | 4.49E-06 |
| rs468120 | TADs | SH3GL3,ENSG00000259986,ENSG00000166503,ADAMTS7P4 | Y | 4.49E-06 |
| rs12172847 | circRNAs |  | Y | 4.5E-06 |
| rs10268866 | circRNAs |  | Y | 4.51E-06 |
| rs12409958 | circRNAs |  | Y | 4.51E-06 |
| rs2048794 | circRNAs |  | Y | 4.51E-06 |
| rs112874039 | circRNAs |  | N | 4.52E-06 |
| rs3780953 | circRNAs,TADs | RBM20,SHOC2,SORCS1,CCDC147,MXI1,XPNPEP1,ENSG00000223381,NRAP,ENSG00000229981,GUCY2GP,HELLS,INPP5A | N | 4.52E-06 |
| rs11740057 | circRNAs |  | Y | 4.53E-06 |
| rs11562897 | TADs | ENSG00000236922 | N | 4.53E-06 |
| rs72898957 | circRNAs |  | N | 4.54E-06 |
| rs113115457 | circRNAs,TADs | TRANK1,GOLGA4 | N | 4.54E-06 |
| rs1884432 | circRNAs |  | Y | 4.54E-06 |
| rs4924287 | TADs | PATL2,GPR176,WDR76 | Y | 4.55E-06 |
| rs6694036 | TADs | FAF1 | N | 4.55E-06 |
| rs10518766 | circRNAs |  | Y | 4.55E-06 |
| rs2293446 | CIRs,TFBRs | ZNFX1,ENSG00000258101,PRKAG1,RCC1,SNHG3,UBE2N,SERINC5,ENSG00000258232,TFRC,SNORD12C,GOLPH3,ENSG00000272086,TBXA2R,RNU6-450P,CACTIN-AS1,ZFAS1 | Y | 4.55E-06 |
| rs62246862 | CIRs | MLH1 | N | 4.55E-06 |
| rs35372971 | TADs | SH3GL3,ENSG00000259986,ENSG00000166503,ADAMTS7P4 | Y | 4.56E-06 |
| rs34692369 | TADs | SH3GL3,ENSG00000259986,ENSG00000166503,ADAMTS7P4 | Y | 4.56E-06 |
| rs4008953 | circRNAs |  | N | 4.57E-06 |
| rs34448227 | circRNAs |  | Y | 4.57E-06 |
| rs78271077 | circRNAs |  | N | 4.58E-06 |
| rs3785074 | circRNAs |  | Y | 4.58E-06 |
| rs3826412 | circRNAs,lncRNAs |  | Y | 4.59E-06 |
| rs11638835 | circRNAs,TADs | STARD9,RTF1 | N | 4.59E-06 |
| rs240064 | circRNAs,CIRs | MAPK1,YPEL1,ENSG00000224086 | Y | 4.59E-06 |
| rs72807808 | circRNAs |  | N | 4.59E-06 |
| rs7741396 | circRNAs |  | Y | 4.59E-06 |
| rs3829814 | lncRNAs |  | N | 4.59E-06 |
| rs71446680 | circRNAs,CIRs | PCID2 | N | 4.6E-06 |
| rs62438671 | circRNAs |  | N | 4.6E-06 |
| rs4835791 | circRNAs |  | N | 4.61E-06 |
| rs72828203 | circRNAs,TADs | RBM20,SHOC2,SORCS1,CCDC147,MXI1,XPNPEP1,ENSG00000223381,NRAP,ENSG00000229981,GUCY2GP,HELLS,INPP5A | N | 4.61E-06 |
| rs4835790 | circRNAs |  | N | 4.61E-06 |
| rs12602514 | TADs,lncRNAs | ENSG00000174093 | Y | 4.61E-06 |
| rs6059928 | circRNAs |  | Y | 4.63E-06 |
| rs6059915 | circRNAs |  | Y | 4.65E-06 |
| rs9356502 | circRNAs |  | N | 4.67E-06 |
| rs10479762 | circRNAs |  | N | 4.67E-06 |
| rs4282054 | circRNAs,TADs,TFBRs | CADPS,FHIT,SMIM4,CACNA2D3,CACNA2D2 | Y | 4.67E-06 |
| rs12916379 | TADs | PATL2,GPR176,WDR76 | Y | 4.68E-06 |
| rs60584955 | circRNAs |  | Y | 4.69E-06 |
| rs11194981 | circRNAs |  | Y | 4.69E-06 |
| rs73404763 | circRNAs,TFBRs | ENSG00000261684 | Y | 4.69E-06 |
| rs111440053 | circRNAs,TADs | TRANK1,GOLGA4 | N | 4.7E-06 |
| rs79879286 | TADs | STK31 | N | 4.7E-06 |
| rs12500921 | circRNAs |  | N | 4.7E-06 |
| rs12333059 | circRNAs |  | N | 4.72E-06 |
| rs545009 | CIRs | RBM4,RNU4-39P,RBM14,RBM14-RBM4,ENSG00000250105 | Y | 4.72E-06 |
| rs76633357 | circRNAs,CIRs | ZNF101 | N | 4.72E-06 |
| rs12156553 | TADs | CDK5RAP2,ASTN2 | N | 4.73E-06 |
| rs1884431 | circRNAs |  | Y | 4.74E-06 |
| rs6059908 | lncRNAs |  | Y | 4.74E-06 |
| rs2302757 | circRNAs |  | Y | 4.74E-06 |
| rs7069128 | circRNAs |  | Y | 4.74E-06 |
| rs6059919 | circRNAs |  | Y | 4.75E-06 |
| rs2635334 | TADs | ENSG00000236922 | N | 4.75E-06 |
| rs1005579 | CIRs | YPEL1 | Y | 4.75E-06 |
| rs9925619 | TADs,TFBRs | HYDIN,ENSG00000260371,SMPD3 | Y | 4.75E-06 |
| rs10791863 | circRNAs |  | Y | 4.76E-06 |
| rs678397 | CIRs | ACTN3 | Y | 4.77E-06 |
| rs9549718 | circRNAs |  | Y | 4.77E-06 |
| rs2635353 | TADs | ENSG00000236922 | N | 4.78E-06 |
| rs11642764 | circRNAs |  | N | 4.79E-06 |
| rs2287251 | circRNAs |  | Y | 4.79E-06 |
| rs1271354 | circRNAs,TADs | SORCS1,SHOC2 | N | 4.79E-06 |
| rs12467914 | TADs | ENSG00000234281 | N | 4.8E-06 |
| rs9549701 | circRNAs |  | Y | 4.8E-06 |
| rs2079883 | circRNAs |  | Y | 4.81E-06 |
| rs12584008 | circRNAs |  | Y | 4.81E-06 |
| rs1572156 | circRNAs |  | Y | 4.81E-06 |
| rs12868163 | circRNAs |  | Y | 4.82E-06 |
| rs34555317 | TADs | JUP,PCTP,FOXK2,CDC27,ENSG00000263317 | Y | 4.83E-06 |
| rs13077007 | CIRs,lncRNAs | CD47 | N | 4.83E-06 |
| rs33368 | circRNAs |  | Y | 4.83E-06 |
| rs72807810 | circRNAs |  | N | 4.84E-06 |
| rs2240920 | circRNAs,TADs,CIRs | VPRBP,NEK4 | Y | 4.84E-06 |
| rs4726268 | TADs | DENND2A | Y | 4.84E-06 |
| rs4065275 | circRNAs,TADs,CIRs | ENSG00000174093,FBXL20,LRRC3C,CDK12,PSMD3 | Y | 4.84E-06 |
| rs3764124 | circRNAs,CIRs | PCID2 | Y | 4.85E-06 |
| rs73404714 | circRNAs |  | N | 4.85E-06 |
| rs9347141 | circRNAs |  | N | 4.85E-06 |
| rs2635361 | TADs | ENSG00000236922 | Y | 4.85E-06 |
| rs11637533 | circRNAs |  | Y | 4.85E-06 |
| rs572846 | CIRs | BBS1,CCS,ENSG00000250105 | Y | 4.85E-06 |
| rs12517410 | circRNAs,CIRs | CDC25C | N | 4.85E-06 |
| rs1506536 | circRNAs |  | N | 4.86E-06 |
| rs13003000 | TADs | ENSG00000234281 | N | 4.87E-06 |
| rs6497524 | circRNAs |  | N | 4.88E-06 |
| rs16973320 | circRNAs |  | N | 4.88E-06 |
| rs36071767 | circRNAs,TADs | ENSG00000174093,CDK12 | Y | 4.88E-06 |
| rs191124455 | CIRs,TFBRs | WHSC1L1,ENSG00000272092 | N | 4.88E-06 |
| rs4911431 | circRNAs |  | Y | 4.88E-06 |
| rs12639551 | circRNAs,TADs,lncRNAs | STT3B,,GADL1,ZCWPW2,RBMS3 | N | 4.89E-06 |
| rs12752254 | TADs | LPHN2,PIGK,MSH4,GIPC2,TTLL7 | Y | 4.89E-06 |
| rs12667625 | circRNAs,CIRs | KMT2E,,LINC01004 | N | 4.9E-06 |
| rs72906805 | TADs | FAF1 | N | 4.9E-06 |
| rs174598 | circRNAs |  | Y | 4.92E-06 |
| rs10269027 | circRNAs |  | Y | 4.92E-06 |
| rs6060009 | circRNAs |  | Y | 4.92E-06 |
| rs10896105 | circRNAs |  | Y | 4.92E-06 |
| rs155022 | circRNAs |  | Y | 4.93E-06 |
| rs11870683 | TADs | ENSG00000174093 | Y | 4.94E-06 |
| rs7088861 | lncRNAs |  | N | 4.94E-06 |
| rs149007343 | circRNAs,TADs | FAF1 | N | 4.94E-06 |
| rs6992943 | circRNAs,CIRs | WHSC1L1 | Y | 4.94E-06 |
| rs12453334 | circRNAs,TADs | ENSG00000174093 | Y | 4.95E-06 |
| rs2025247 | circRNAs |  | Y | 4.95E-06 |
| rs1552246 | TADs | ENSG00000253877 | N | 4.96E-06 |
| rs72906804 | TADs | FAF1 | N | 4.97E-06 |
| rs4911157 | circRNAs |  | Y | 4.97E-06 |
| rs72828241 | circRNAs,TADs | SORCS1,SHOC2 | N | 4.98E-06 |
| rs10821795 | CIRs | CDK1 | N | 4.98E-06 |
| rs6534040 | TADs | ENSG00000236922 | N | 4.98E-06 |
| rs3118119 | CIRs | HIST2H2AB,BOLA1 | N | 4.99E-06 |
| rs9641341 | circRNAs,CIRs | KMT2E,,LINC01004 | N | 4.99E-06 |
| rs1180221 | circRNAs |  | N | 5E-06 |
| rs12913179 | circRNAs,TADs | SH3GL3,ENSG00000259986,ENSG00000166503,ADAMTS7P4 | Y | 5E-06 |
| rs4610671 | circRNAs |  | N | 5.01E-06 |
| rs1948240 | TADs | ENSG00000236922 | N | 5.02E-06 |
| rs3803393 | circRNAs,TADs | SH3GL3,ENSG00000259986,ENSG00000166503,ADAMTS7P4 | Y | 5.03E-06 |
| rs12913942 | circRNAs,TADs | SH3GL3,ENSG00000259986,ENSG00000166503,ADAMTS7P4 | Y | 5.03E-06 |
| rs3803394 | TADs | SH3GL3,ENSG00000259986,ENSG00000166503,ADAMTS7P4 | Y | 5.03E-06 |
| rs61881587 | circRNAs,TADs | SORCS1,SHOC2 | N | 5.03E-06 |
| rs261007 | circRNAs |  | Y | 5.04E-06 |
| rs56054520 | TADs | SH3GL3,ENSG00000259986,ENSG00000166503,ADAMTS7P4 | Y | 5.04E-06 |
| rs17138171 | circRNAs |  | Y | 5.04E-06 |
| rs9348164 | circRNAs |  | Y | 5.04E-06 |
| rs2316463 | circRNAs |  | Y | 5.05E-06 |
| rs10884919 | circRNAs,TADs | SORCS1,SHOC2 | N | 5.06E-06 |
| rs8050843 | circRNAs |  | N | 5.06E-06 |
| rs6577035 | circRNAs |  | Y | 5.07E-06 |
| rs7763880 | circRNAs |  | Y | 5.07E-06 |
| rs58120505 | circRNAs |  | N | 5.07E-06 |
| rs11632988 | TADs,TFBRs | SH3GL3,ENSG00000259986,ENSG00000221247,ENSG00000166503,ADAMTS7P4 | Y | 5.08E-06 |
| rs11633181 | circRNAs,TADs | SH3GL3,ENSG00000259986,ENSG00000166503,ADAMTS7P4 | Y | 5.09E-06 |
| rs9783184 | circRNAs |  | N | 5.09E-06 |
| rs4867897 | circRNAs |  | Y | 5.1E-06 |
| rs58999833 | TADs,CIRs | SLAMF9,ENSG00000228560,IGSF9,AIM2 | N | 5.1E-06 |
| rs9549709 | circRNAs,CIRs | PCID2 | Y | 5.1E-06 |
| rs12569425 | circRNAs,TADs | SORCS1,SHOC2 | Y | 5.1E-06 |
| rs3764125 | circRNAs,CIRs | PCID2 | Y | 5.11E-06 |
| rs11194947 | circRNAs,TFBRs | RN7SL450P | N | 5.11E-06 |
| rs77204993 | circRNAs |  | N | 5.12E-06 |
| rs215001 | circRNAs,TADs | OPRM1,SYNE1,IPCEF1,CCDC170 | Y | 5.12E-06 |
| rs16887275 | circRNAs |  | Y | 5.15E-06 |
| rs2898674 | circRNAs,CIRs | ENSG00000272092 | Y | 5.15E-06 |
| rs2284239 | circRNAs |  | N | 5.16E-06 |
| rs2969625 | TADs | ENSG00000236922 | Y | 5.16E-06 |
| rs34867153 | TADs,CIRs | JUP,ACACA,CASC3 | N | 5.18E-06 |
| rs35986397 | TADs | SH3GL3,ENSG00000259986,ENSG00000166503,ADAMTS7P4 | Y | 5.2E-06 |
| rs12584981 | circRNAs |  | Y | 5.2E-06 |
| rs12463845 | TADs | ENSG00000234281 | N | 5.2E-06 |
| rs10105555 | circRNAs |  | Y | 5.2E-06 |
| rs4647903 | circRNAs,CIRs | ENSG00000255201 | Y | 5.2E-06 |
| rs10433550 | circRNAs,TADs | PBRM1 | Y | 5.21E-06 |
| rs3779921 | circRNAs |  | Y | 5.22E-06 |
| rs2287247 | circRNAs |  | Y | 5.23E-06 |
| rs7006757 | circRNAs |  | Y | 5.23E-06 |
| rs4081123 | TADs | SH3GL3,ENSG00000259986,ENSG00000166503,ADAMTS7P4 | Y | 5.23E-06 |
| rs3850451 | circRNAs |  | Y | 5.23E-06 |
| rs62029597 | TADs | SH3GL3,ENSG00000259986,ENSG00000166503,ADAMTS7P4 | Y | 5.23E-06 |
| rs62535709 | circRNAs |  | Y | 5.23E-06 |
| rs68184996 | circRNAs,TADs | SH3GL3,ENSG00000259986,ENSG00000166503,ADAMTS7P4 | Y | 5.24E-06 |
| rs9549715 | circRNAs |  | Y | 5.25E-06 |
| rs28865701 | circRNAs |  | N | 5.25E-06 |
| rs7999579 | circRNAs |  | Y | 5.26E-06 |
| rs4793079 | CIRs,TFBRs | HDAC5,C17orf53,ATXN7L3 | Y | 5.28E-06 |
| rs2293971 | circRNAs |  | Y | 5.28E-06 |
| rs2302756 | circRNAs |  | Y | 5.28E-06 |
| rs7706425 | circRNAs |  | Y | 5.28E-06 |
| rs2302754 | circRNAs |  | Y | 5.29E-06 |
| rs10783300 | circRNAs |  | Y | 5.29E-06 |
| rs138339060 | circRNAs |  | N | 5.3E-06 |
| rs4516437 | TADs | DPP10 | N | 5.32E-06 |
| rs6542200 | TADs | DPP10 | N | 5.32E-06 |
| rs2155030 | circRNAs,CIRs,TFBRs | ENSG00000254461 | Y | 5.33E-06 |
| rs28472726 | TADs | ZNF208,ENSG00000267383,ENSG00000141979 | N | 5.33E-06 |
| rs62029596 | TADs | SH3GL3,ENSG00000259986,ENSG00000166503,ADAMTS7P4 | Y | 5.33E-06 |
| rs56046724 | circRNAs |  | N | 5.34E-06 |
| rs9938316 | circRNAs,CIRs | PDF,COG8 | Y | 5.34E-06 |
| rs2635209 | TADs | ENSG00000236922 | N | 5.36E-06 |
| rs12309 | TADs | ENSG00000174093,CDK12 | Y | 5.36E-06 |
| rs10103315 | circRNAs,CIRs | WHSC1L1 | Y | 5.38E-06 |
| rs7001340 | circRNAs |  | N | 5.38E-06 |
| rs2240455 | circRNAs,CIRs | KMT2E,,LINC01004 | Y | 5.38E-06 |
| rs912012 | circRNAs |  | Y | 5.39E-06 |
| rs72898961 | circRNAs |  | N | 5.39E-06 |
| rs62211617 | TADs | CEP250,CNBD2 | Y | 5.4E-06 |
| rs11699062 | TADs | CEP250,CNBD2 | Y | 5.4E-06 |
| rs113213591 | TADs | STK31 | N | 5.41E-06 |
| rs35946436 | circRNAs |  | N | 5.41E-06 |
| rs9549708 | circRNAs |  | Y | 5.41E-06 |
| rs12452367 | circRNAs |  | N | 5.41E-06 |
| rs11763870 | circRNAs |  | N | 5.41E-06 |
| rs56365338 | circRNAs |  | Y | 5.42E-06 |
| rs62535711 | circRNAs |  | Y | 5.42E-06 |
| rs2635239 | TADs | ENSG00000236922 | N | 5.43E-06 |
| rs59176647 | circRNAs |  | N | 5.43E-06 |
| rs10401926 | TADs | ZNF208,ENSG00000267383,ENSG00000141979 | Y | 5.43E-06 |
| rs10958567 | circRNAs |  | Y | 5.43E-06 |
| rs2316443 | circRNAs,TFBRs | PCID2 | Y | 5.43E-06 |
| rs28735056 | circRNAs |  | N | 5.44E-06 |
| rs72805345 | TADs | WDPCP,PNPT1,PUS10,ENSG00000233891,EHBP1,ENSG00000204929,COMMD1,LINC01122,ENSG00000271955,CTNNA2,C1D | N | 5.44E-06 |
| rs12942155 | CIRs | MBTD1 | Y | 5.45E-06 |
| rs12868316 | circRNAs |  | Y | 5.46E-06 |
| rs6466046 | circRNAs |  | N | 5.46E-06 |
| rs4656859 | TADs,CIRs,TFBRs | ENSG00000228560,IGSF9,AIM2 | N | 5.47E-06 |
| rs10896076 | circRNAs |  | N | 5.48E-06 |
| rs6893826 | circRNAs |  | N | 5.5E-06 |
| rs7214540 | circRNAs |  | Y | 5.5E-06 |
| rs739461 | circRNAs |  | Y | 5.51E-06 |
| rs11879838 | circRNAs |  | N | 5.51E-06 |
| rs35569628 | circRNAs |  | Y | 5.51E-06 |
| rs7502971 | circRNAs,TADs,CIRs | ENSG00000174093,CDK12,CASC3 | Y | 5.52E-06 |
| rs12869947 | circRNAs,CIRs | PCID2 | Y | 5.53E-06 |
| rs4794566 | TADs | PCTP | N | 5.53E-06 |
| rs10889181 | lncRNAs |  | N | 5.54E-06 |
| rs9549699 | circRNAs |  | Y | 5.54E-06 |
| rs73404730 | circRNAs |  | N | 5.54E-06 |
| rs6059916 | circRNAs |  | Y | 5.56E-06 |
| rs703986 | circRNAs |  | N | 5.57E-06 |
| rs6060042 | circRNAs,TADs | FER1L4,NCOA6,ASIP | Y | 5.57E-06 |
| rs4564863 | circRNAs |  | Y | 5.57E-06 |
| rs10849910 | TADs | SVOP,CKAP4,SH2B3,TCHP | N | 5.58E-06 |
| rs71446682 | circRNAs,CIRs | PCID2 | N | 5.58E-06 |
| rs10958700 | circRNAs |  | Y | 5.58E-06 |
| rs183337163 | circRNAs,TADs | FAF1 | N | 5.59E-06 |
| rs4082793 | circRNAs |  | N | 5.59E-06 |
| rs10129264 | circRNAs |  | N | 5.59E-06 |
| rs6497540 | circRNAs |  | Y | 5.59E-06 |
| rs9347142 | circRNAs |  | N | 5.59E-06 |
| rs2287246 | circRNAs |  | Y | 5.6E-06 |
| rs591776 | circRNAs |  | N | 5.6E-06 |
| rs6142199 | circRNAs |  | Y | 5.61E-06 |
| rs615205 | CIRs,TFBRs | CTSF | Y | 5.61E-06 |
| rs1688643 | circRNAs |  | N | 5.62E-06 |
| rs12603332 | TADs,CIRs,TFBRs | ENSG00000174093,FBXL20,CDK12,ENSG00000264968 | Y | 5.62E-06 |
| rs2241726 | circRNAs |  | Y | 5.63E-06 |
| rs12764899 | circRNAs,CIRs | AS3MT | Y | 5.63E-06 |
| rs9549710 | circRNAs |  | Y | 5.63E-06 |
| rs6060040 | circRNAs |  | Y | 5.63E-06 |
| rs7212573 | circRNAs |  | Y | 5.64E-06 |
| rs13317 | circRNAs,CIRs | ENSG00000255201 | Y | 5.65E-06 |
| rs10791861 | circRNAs,CIRs | YIF1A | Y | 5.66E-06 |
| rs3024739 | TFBRs | ENSG00000269125 | Y | 5.66E-06 |
| rs12584813 | circRNAs,CIRs | PCID2 | Y | 5.67E-06 |
| rs6944847 | circRNAs |  | N | 5.68E-06 |
| rs4656250 | TADs | AIM2 | N | 5.68E-06 |
| rs10129812 | circRNAs |  | N | 5.68E-06 |
| rs12154649 | TADs | STK31 | N | 5.68E-06 |
| rs7222752 | CIRs | NPTX1 | N | 5.69E-06 |
| rs8080943 | CIRs | MBTD1 | Y | 5.69E-06 |
| rs12913791 | circRNAs,TADs | SH3GL3,ENSG00000259986,ENSG00000166503,ADAMTS7P4 | Y | 5.69E-06 |
| rs7220360 | CIRs | MBTD1 | Y | 5.7E-06 |
| rs16957091 | circRNAs,TADs | EPB42 | Y | 5.7E-06 |
| rs932542 | circRNAs |  | Y | 5.7E-06 |
| rs4099847 | TADs | SH3GL3,ENSG00000259986,ENSG00000166503,ADAMTS7P4 | Y | 5.72E-06 |
| rs10467004 | circRNAs |  | N | 5.72E-06 |
| rs11227433 | circRNAs |  | Y | 5.74E-06 |
| rs7298691 | circRNAs |  | N | 5.75E-06 |
| rs111481776 | circRNAs,TADs | SORCS1,SHOC2 | N | 5.75E-06 |
| rs34989271 | circRNAs,CIRs | PCID2 | Y | 5.75E-06 |
| rs72805988 | circRNAs |  | N | 5.76E-06 |
| rs75139387 | circRNAs |  | Y | 5.78E-06 |
| rs6059936 | circRNAs |  | Y | 5.78E-06 |
| rs9549711 | circRNAs |  | Y | 5.79E-06 |
| rs3861721 | circRNAs |  | Y | 5.79E-06 |
| rs13336666 | circRNAs |  | N | 5.79E-06 |
| rs9549364 | circRNAs |  | Y | 5.8E-06 |
| rs11619691 | circRNAs |  | Y | 5.8E-06 |
| rs9549713 | circRNAs |  | Y | 5.8E-06 |
| rs3861720 | circRNAs |  | Y | 5.8E-06 |
| rs4099846 | TADs | SH3GL3,ENSG00000259986,ENSG00000166503,ADAMTS7P4 | Y | 5.8E-06 |
| rs2711129 | circRNAs,TADs | MPP6 | N | 5.81E-06 |
| rs2011258 | circRNAs |  | N | 5.81E-06 |
| rs2236652 | circRNAs |  | Y | 5.81E-06 |
| rs6059931 | circRNAs |  | Y | 5.82E-06 |
| rs10900851 | CIRs | ENSG00000249971 | N | 5.82E-06 |
| rs2155031 | circRNAs,CIRs,TFBRs | ENSG00000254461 | Y | 5.82E-06 |
| rs560556 | CIRs,TFBRs | CCDC87,CTSF | Y | 5.82E-06 |
| rs58641431 | circRNAs |  | Y | 5.83E-06 |
| rs35269451 | circRNAs,CIRs | MBTD1 | Y | 5.83E-06 |
| rs7908878 | CIRs | ENSG00000228417 | Y | 5.84E-06 |
| rs2315271 | circRNAs |  | N | 5.84E-06 |
| rs2236651 | circRNAs,CIRs | ENSG00000245156 | Y | 5.84E-06 |
| rs86708 | CIRs | MCHR1 | Y | 5.85E-06 |
| rs4393319 | circRNAs,CIRs | ENSG00000245156 | Y | 5.87E-06 |
| rs10889184 | lncRNAs |  | N | 5.88E-06 |
| rs9905051 | circRNAs |  | Y | 5.89E-06 |
| rs9549712 | circRNAs |  | Y | 5.9E-06 |
| rs57747440 | circRNAs,CIRs | PCID2 | Y | 5.91E-06 |
| rs10958682 | circRNAs,CIRs | ENSG00000255201 | Y | 5.91E-06 |
| rs7201930 | circRNAs |  | N | 5.91E-06 |
| rs6120684 | circRNAs |  | Y | 5.91E-06 |
| rs4410355 | circRNAs |  | Y | 5.92E-06 |
| rs71446679 | circRNAs,CIRs | PCID2 | N | 5.92E-06 |
| rs35181082 | circRNAs |  | Y | 5.92E-06 |
| rs10240574 | circRNAs |  | N | 5.92E-06 |
| rs72898946 | circRNAs |  | N | 5.92E-06 |
| rs6087602 | circRNAs |  | Y | 5.93E-06 |
| rs10735363 | circRNAs |  | Y | 5.93E-06 |
| rs1331621 | TADs | CDK5RAP2,ASTN2 | N | 5.94E-06 |
| rs10129582 | circRNAs |  | N | 5.95E-06 |
| rs5844475 | circRNAs |  | Y | 5.95E-06 |
| rs11971732 | lncRNAs |  | N | 5.95E-06 |
| rs6087662 | circRNAs |  | Y | 5.95E-06 |
| rs2282755 | circRNAs |  | Y | 5.96E-06 |
| rs7833995 | TADs | ENSG00000253877 | N | 5.97E-06 |
| rs6059937 | circRNAs |  | Y | 5.98E-06 |
| rs35522895 | TADs | SH3GL3,ENSG00000259986,ENSG00000166503,ADAMTS7P4 | Y | 6E-06 |
| rs10249965 | circRNAs |  | N | 6E-06 |
| rs9915554 | CIRs | MBTD1 | Y | 6.01E-06 |
| rs6452412 | circRNAs |  | N | 6.01E-06 |
| rs115640879 | circRNAs |  | N | 6.01E-06 |
| rs2521760 | circRNAs,TADs | DFNA5,CCDC126 | Y | 6.01E-06 |
| rs740300 | circRNAs |  | Y | 6.01E-06 |
| rs1814518 | TADs,CIRs,TFBRs,lncRNAs | EIF4EBP2P2,FSIP1,STARD9,TP53BP1,RTF1,PLA2G4E | N | 6.02E-06 |
| rs12911585 | circRNAs,TADs | STARD9,ENSG00000259536 | Y | 6.02E-06 |
| rs11130326 | circRNAs,TADs | NEK4 | Y | 6.02E-06 |
| rs7994324 | circRNAs |  | Y | 6.03E-06 |
| rs11168822 | circRNAs |  | Y | 6.03E-06 |
| rs10896101 | circRNAs |  | Y | 6.03E-06 |
| rs12708640 | circRNAs |  | N | 6.04E-06 |
| rs2378199 | circRNAs |  | Y | 6.04E-06 |
| rs215005 | circRNAs,TADs | SYNE1,IPCEF1,RGS17,TFB1M | N | 6.04E-06 |
| rs2238056 | circRNAs |  | Y | 6.04E-06 |
| rs2295443 | circRNAs |  | Y | 6.05E-06 |
| rs6060047 | circRNAs,TADs | FER1L4,NCOA6,ASIP | Y | 6.05E-06 |
| rs7780887 | circRNAs |  | Y | 6.06E-06 |
| rs2843738 | CIRs | ENSG00000253437 | Y | 6.06E-06 |
| rs34725331 | circRNAs,CIRs | PBX4 | N | 6.06E-06 |
| rs62533709 | circRNAs |  | Y | 6.08E-06 |
| rs75595651 | circRNAs |  | N | 6.08E-06 |
| rs7169009 | TADs,TFBRs | EPB42,CDAN1,ENSG00000246283 | Y | 6.08E-06 |
| rs77087420 | circRNAs |  | N | 6.09E-06 |
| rs9348162 | circRNAs |  | Y | 6.1E-06 |
| rs12906497 | TADs | EPB42 | N | 6.11E-06 |
| rs476551 | circRNAs |  | Y | 6.11E-06 |
| rs2132157 | TADs | PATL2,GPR176,WDR76 | Y | 6.11E-06 |
| rs7015738 | TADs | ENSG00000253877 | N | 6.12E-06 |
| rs2287253 | circRNAs |  | Y | 6.13E-06 |
| rs10739538 | TADs | CDK5RAP2,ASTN2 | N | 6.13E-06 |
| rs697240 | circRNAs |  | N | 6.14E-06 |
| rs9549706 | circRNAs |  | Y | 6.15E-06 |
| rs917834 | circRNAs |  | N | 6.17E-06 |
| rs10280470 | circRNAs |  | Y | 6.17E-06 |
| rs2239192 | TADs | SVOP,CKAP4,SH2B3,TCHP | N | 6.19E-06 |
| rs2276036 | circRNAs,CIRs,TFBRs | ENSG00000254855,ENSG00000255468,B3GNT1 | Y | 6.19E-06 |
| rs6088515 | circRNAs,CIRs | DYNLRB1,ENSG00000206582 | Y | 6.2E-06 |
| rs5755654 | circRNAs |  | Y | 6.2E-06 |
| rs9938117 | circRNAs |  | N | 6.2E-06 |
| rs11974928 | lncRNAs |  | N | 6.21E-06 |
| rs2068474 | circRNAs |  | Y | 6.21E-06 |
| rs12028665 | CIRs | BCAS4 | N | 6.23E-06 |
| rs61847579 | CIRs | MTMR3 | N | 6.24E-06 |
| rs2017969 | circRNAs |  | Y | 6.24E-06 |
| rs10808140 | circRNAs |  | Y | 6.24E-06 |
| rs7406066 | circRNAs |  | Y | 6.25E-06 |
| rs2396170 | circRNAs |  | N | 6.25E-06 |
| rs2378249 | circRNAs |  | Y | 6.26E-06 |
| rs9806806 | circRNAs |  | N | 6.26E-06 |
| rs12672764 | lncRNAs |  | Y | 6.27E-06 |
| rs960437 | circRNAs |  | N | 6.29E-06 |
| rs2240453 | circRNAs,lncRNAs |  | Y | 6.29E-06 |
| rs6059961 | circRNAs |  | Y | 6.3E-06 |
| rs34591918 | TADs | SH3GL3,ENSG00000259986,ENSG00000166503,ADAMTS7P4 | Y | 6.3E-06 |
| rs6087622 | circRNAs |  | Y | 6.31E-06 |
| rs12945077 | CIRs | MBTD1 | Y | 6.31E-06 |
| rs13227554 | circRNAs |  | N | 6.31E-06 |
| rs60173821 | TADs | ENSG00000234281 | N | 6.32E-06 |
| rs10279486 | circRNAs |  | N | 6.32E-06 |
| rs6058089 | circRNAs |  | Y | 6.32E-06 |
| rs531490 | CIRs | ACTN3 | Y | 6.33E-06 |
| rs4291157 | circRNAs |  | Y | 6.34E-06 |
| rs2889849 | circRNAs |  | Y | 6.34E-06 |
| rs871925 | circRNAs |  | N | 6.34E-06 |
| rs11655732 | circRNAs |  | Y | 6.34E-06 |
| rs139639752 | TADs | STAT4 | N | 6.34E-06 |
| rs8047364 | circRNAs |  | N | 6.35E-06 |
| rs2117029 | circRNAs |  | Y | 6.35E-06 |
| rs6931604 | circRNAs |  | N | 6.36E-06 |
| rs214967 | circRNAs,TADs | OPRM1,SYNE1,IPCEF1,CCDC170 | N | 6.36E-06 |
| rs60446665 | circRNAs |  | N | 6.37E-06 |
| rs2284350 | TADs | VPRBP | Y | 6.38E-06 |
| rs1572155 | circRNAs |  | Y | 6.38E-06 |
| rs2635331 | TADs | ENSG00000236922 | N | 6.38E-06 |
| rs2336148 | circRNAs,TADs | PBRM1 | Y | 6.4E-06 |
| rs2581810 | circRNAs |  | Y | 6.4E-06 |
| rs9549702 | circRNAs |  | Y | 6.41E-06 |
| rs17817995 | TADs | PCTP | Y | 6.42E-06 |
| rs9907636 | circRNAs |  | Y | 6.43E-06 |
| rs11976329 | circRNAs |  | N | 6.44E-06 |
| rs10249170 | circRNAs |  | N | 6.44E-06 |
| rs7925123 | circRNAs |  | Y | 6.48E-06 |
| rs2635344 | TADs | ENSG00000236922 | N | 6.48E-06 |
| rs12910334 | TADs | SH3GL3,ENSG00000259986,ENSG00000166503,ADAMTS7P4 | Y | 6.49E-06 |
| rs34926599 | TFBRs | FTH1P5 | N | 6.5E-06 |
| rs2293445 | circRNAs,CIRs | ENSG00000257913 | Y | 6.51E-06 |
| rs2211577 | TADs | LPHN2,GIPC2 | N | 6.52E-06 |
| rs6059969 | circRNAs |  | Y | 6.52E-06 |
| rs9463652 | TFBRs | RPS17P5 | N | 6.53E-06 |
| rs1854895 | TADs | CDK5RAP2 | N | 6.53E-06 |
| rs12540007 | circRNAs |  | Y | 6.53E-06 |
| rs71559038 | CIRs | HIST1H2BK | N | 6.53E-06 |
| rs10818312 | TADs | CDK5RAP2,ASTN2 | N | 6.54E-06 |
| rs28434158 | circRNAs |  | N | 6.55E-06 |
| rs59308327 | TADs | CEP250,CNBD2 | Y | 6.55E-06 |
| rs3110496 | CIRs,TFBRs | ANKRD13B,ENSG00000264647 | Y | 6.56E-06 |
| rs7755114 | circRNAs |  | Y | 6.56E-06 |
| rs1138908 | circRNAs,CIRs | ENSG00000258101 | Y | 6.58E-06 |
| rs2635369 | TADs | ENSG00000236922 | N | 6.58E-06 |
| rs6718852 | TADs | DPP10 | N | 6.58E-06 |
| rs548985 | circRNAs,TADs | SYNE1,HDAC2,ENSG00000235652,RGS17,CCDC170 | N | 6.58E-06 |
| rs9549694 | circRNAs |  | Y | 6.58E-06 |
| rs9936542 | circRNAs |  | N | 6.59E-06 |
| rs7810119 | circRNAs,CIRs | ENSG00000272918 | N | 6.59E-06 |
| rs11231629 | circRNAs |  | N | 6.59E-06 |
| rs2529090 | circRNAs,TADs | MPP6 | N | 6.6E-06 |
| rs2316442 | circRNAs |  | Y | 6.6E-06 |
| rs28867132 | circRNAs |  | Y | 6.6E-06 |
| rs7073969 | circRNAs,TADs | SORCS1,SHOC2 | N | 6.63E-06 |
| rs174538 | circRNAs,CIRs,TFBRs | MIR611,RPL35,ZNF639,CBX3,TMEM258,ENSG00000272888,ZBED5-AS1,DCAF11,BEST1,INCENP,CHD2,CSDE1,ABCB10,GEMIN6,CFL1P1,FEN1,ENSG00000273419,FADS3,FADS2,SRSF7,NRL,ARPC5L,FNBP4,CCDC171,DNTTIP2 | Y | 6.63E-06 |
| rs6868422 | circRNAs |  | N | 6.64E-06 |
| rs9549361 | circRNAs |  | Y | 6.65E-06 |
| rs78068749 | TADs | RAB3B,AGBL4,LRP8,FAF1 | N | 6.66E-06 |
| rs12534140 | circRNAs,CIRs | KMT2E,,LINC01004 | N | 6.67E-06 |
| rs2155198 | TFBRs,lncRNAs | ENSG00000255320 | Y | 6.68E-06 |
| rs12508789 | circRNAs |  | N | 6.68E-06 |
| rs17126931 | circRNAs,TADs,TFBRs | SORCS1,SHOC2,ADD3 | N | 6.68E-06 |
| rs3794799 | circRNAs |  | Y | 6.68E-06 |
| rs94194 | circRNAs |  | Y | 6.69E-06 |
| rs7785197 | circRNAs |  | N | 6.69E-06 |
| rs72898944 | circRNAs |  | N | 6.7E-06 |
| rs1604607 | TADs | EPS15,STIL,AGBL4-IT1,AGBL4,SMIM12,LRP8,USP24,TTC39A | N | 6.71E-06 |
| rs2287223 | circRNAs |  | Y | 6.71E-06 |
| rs17680262 | TADs,CIRs | TCHP,MYO1H | N | 6.71E-06 |
| rs11079946 | circRNAs |  | Y | 6.72E-06 |
| rs1948217 | TADs | ENSG00000236922 | N | 6.72E-06 |
| rs7298574 | circRNAs |  | N | 6.72E-06 |
| rs9881468 | TADs,CIRs,TFBRs | VPRBP,ITIH3 | Y | 6.73E-06 |
| rs3992625 | TADs | RAB3B,AGBL4,LRP8,FAF1 | N | 6.73E-06 |
| rs4794565 | TADs | PCTP | Y | 6.74E-06 |
| rs10958683 | circRNAs,CIRs | ENSG00000255201 | N | 6.75E-06 |
| rs12453513 | circRNAs |  | Y | 6.76E-06 |
| rs9915264 | circRNAs |  | Y | 6.76E-06 |
| rs4848364 | TADs | DPP10 | N | 6.76E-06 |
| rs4483592 | circRNAs,CIRs | ENSG00000254510,RIN1,KLC2,YIF1A | Y | 6.77E-06 |
| rs2240457 | circRNAs,CIRs | ENSG00000272918 | Y | 6.77E-06 |
| rs45510091 | circRNAs |  | N | 6.77E-06 |
| rs77735715 | TADs | DPP10 | N | 6.78E-06 |
| rs3735377 | CIRs,lncRNAs | KMT2E,,ENSG00000249492,C5orf28,ENSG00000259884,LINC01004 | N | 6.78E-06 |
| rs45510500 | circRNAs |  | N | 6.79E-06 |
| rs17537498 | TADs | WDPCP,PNPT1,PUS10,ENSG00000233891,EHBP1,ENSG00000204929,COMMD1,LINC01122,ENSG00000271955,CTNNA2,C1D | N | 6.8E-06 |
| rs4331993 | circRNAs,TADs | OPRM1,SYNE1,IPCEF1,CCDC170 | N | 6.81E-06 |
| rs6788153 | TADs | OPA1,FGF12,LEPREL1,TPRG1,OSTN | N | 6.82E-06 |
| rs12411130 | TADs | LPHN2,PIGK,MSH4,GIPC2,TTLL7 | N | 6.83E-06 |
| rs11205728 | circRNAs |  | Y | 6.84E-06 |
| rs10818310 | TADs | CDK5RAP2,ASTN2 | N | 6.84E-06 |
| rs2401443 | TADs | SH3GL3,ENSG00000259986,ENSG00000166503,ADAMTS7P4 | Y | 6.85E-06 |
| rs2378260 | circRNAs,TADs | FER1L4,NCOA6,ASIP | Y | 6.86E-06 |
| rs6577034 | circRNAs |  | Y | 6.86E-06 |
| rs72904801 | TADs,CIRs | MUC1,MIR92B,CDKN2C,FAF1 | N | 6.87E-06 |
| rs9910220 | circRNAs |  | Y | 6.87E-06 |
| rs1567126 | circRNAs |  | N | 6.88E-06 |
| rs4647905 | circRNAs,CIRs | ENSG00000255201 | Y | 6.89E-06 |
| rs3118125 | CIRs | HIST2H2BE,BOLA1 | Y | 6.91E-06 |
| rs73990674 | TADs | PCTP | N | 6.91E-06 |
| rs57495888 | circRNAs |  | Y | 6.91E-06 |
| rs3789586 | circRNAs |  | Y | 6.93E-06 |
| rs28460372 | circRNAs |  | N | 6.93E-06 |
| rs62068171 | TADs | ENSG00000174093,FBXL20,CDK12 | Y | 6.93E-06 |
| rs6087608 | circRNAs |  | Y | 6.95E-06 |
| rs2721816 | circRNAs,TADs | MPP6 | Y | 6.95E-06 |
| rs10231782 | circRNAs |  | N | 6.95E-06 |
| rs6915668 | circRNAs |  | N | 6.96E-06 |
| rs6915670 | circRNAs |  | N | 6.96E-06 |
| rs2240465 | circRNAs |  | Y | 6.96E-06 |
| rs4425086 | TADs | DPP10 | N | 6.97E-06 |
| rs56301252 | TADs,CIRs | ENSG00000174093,FBXL20,CDK12,PSMD3,ENSG00000264968 | Y | 6.97E-06 |
| rs62535674 | circRNAs |  | Y | 6.97E-06 |
| rs4656861 | TADs | AIM2 | N | 6.98E-06 |
| rs2237605 | circRNAs,CIRs | ENSG00000272918 | N | 6.98E-06 |
| rs9889777 | circRNAs,TADs,CIRs,TFBRs | SPAG9,NME2,MBTD1,NME1-NME2,CA10,NME1,UTP18,PLEKHM1,LRRC37A3,SKA2 | Y | 6.99E-06 |
| rs72847313 | lncRNAs |  | N | 7E-06 |
| rs8001506 | circRNAs |  | Y | 0.000007 |
| rs2721785 | circRNAs,TADs | MPP6 | N | 7E-06 |
| rs4730068 | circRNAs |  | N | 7E-06 |
| rs11223791 | TFBRs | B3GAT1 | N | 7.01E-06 |
| rs80108923 | circRNAs |  | N | 7.01E-06 |
| rs10075648 | circRNAs |  | N | 7.01E-06 |
| rs2372733 | circRNAs |  | N | 7.02E-06 |
| rs2237602 | circRNAs,CIRs | ENSG00000272918 | N | 7.03E-06 |
| rs12123061 | TADs | LPHN2,GIPC2 | N | 7.03E-06 |
| rs7209638 | circRNAs,TADs | CA10,PLEKHM1,LRRC37A3 | Y | 7.04E-06 |
| rs17750747 | lncRNAs |  | Y | 7.05E-06 |
| rs2372732 | circRNAs |  | N | 7.06E-06 |
| rs11656911 | circRNAs |  | Y | 7.07E-06 |
| rs6060025 | circRNAs |  | Y | 7.07E-06 |
| rs3794297 | circRNAs |  | N | 7.09E-06 |
| rs79280580 | TADs | CDK5RAP2 | N | 7.09E-06 |
| rs8080734 | TADs | ENSG00000174093,FBXL20,CDK12 | Y | 7.11E-06 |
| rs11788723 | TADs | CDK5RAP2 | N | 7.11E-06 |
| rs55963510 | CIRs | HIST2H2AA4,VPS45 | N | 7.14E-06 |
| rs6887937 | circRNAs |  | N | 7.15E-06 |
| rs12943083 | circRNAs,CIRs | MBTD1,UTP18 | Y | 7.16E-06 |
| rs8061352 | circRNAs,CIRs | MIR1538,ENSG00000143674,CYB5B | Y | 7.16E-06 |
| rs10983555 | circRNAs |  | N | 7.17E-06 |
| rs72898951 | circRNAs |  | N | 7.18E-06 |
| rs660879 | circRNAs |  | N | 7.22E-06 |
| rs3799585 | circRNAs |  | N | 7.22E-06 |
| rs9295357 | circRNAs |  | Y | 7.23E-06 |
| rs1984413 | circRNAs |  | Y | 7.23E-06 |
| rs6868748 | circRNAs |  | N | 7.23E-06 |
| rs2298468 | circRNAs |  | Y | 7.24E-06 |
| rs2299299 | circRNAs,CIRs,lncRNAs | KMT2E, | Y | 7.25E-06 |
| rs536809 | TADs,CIRs,TFBRs | MRPS33,DENND2A,TMEM178B | Y | 7.25E-06 |
| rs7166632 | circRNAs |  | N | 7.26E-06 |
| rs10261782 | circRNAs |  | Y | 7.26E-06 |
| rs10431449 | circRNAs |  | N | 7.26E-06 |
| rs174595 | circRNAs |  | Y | 7.28E-06 |
| rs56999964 | circRNAs,TADs | SORCS1,SHOC2 | N | 7.28E-06 |
| rs8077529 | circRNAs |  | Y | 7.28E-06 |
| rs10431448 | circRNAs |  | Y | 7.28E-06 |
| rs28823350 | circRNAs |  | N | 7.28E-06 |
| rs3804644 | circRNAs |  | N | 7.31E-06 |
| rs113779084 | circRNAs,TFBRs | THSD7A | N | 7.32E-06 |
| rs214971 | circRNAs,TADs | OPRM1,SYNE1,IPCEF1,CCDC170 | N | 7.32E-06 |
| rs184773571 | circRNAs |  | N | 7.33E-06 |
| rs67652222 | CIRs | HIST1H2BL | N | 7.34E-06 |
| rs10888697 | circRNAs |  | Y | 7.34E-06 |
| rs7223928 | circRNAs,TADs | CA10,PLEKHM1,LRRC37A3 | Y | 7.35E-06 |
| rs71474496 | circRNAs,TADs | STARD9,RTF1 | N | 7.36E-06 |
| rs7830233 | circRNAs |  | N | 7.36E-06 |
| rs8068115 | circRNAs,TADs | CA10,PLEKHM1,LRRC37A3 | Y | 7.37E-06 |
| rs325400 | circRNAs,CIRs | HNRNPA1P62 | Y | 7.38E-06 |
| rs844499 | circRNAs |  | N | 7.38E-06 |
| rs2288865 | circRNAs,TADs | ZNF208,ENSG00000267383,ENSG00000141979 | Y | 7.38E-06 |
| rs113922010 | TADs | STK31 | N | 7.39E-06 |
| rs9350984 | circRNAs |  | N | 7.4E-06 |
| rs6088538 | circRNAs |  | Y | 7.4E-06 |
| rs6727756 | TADs | STAT1 | N | 7.42E-06 |
| rs2743327 | CIRs | RBPJL,TOMM34 | N | 7.42E-06 |
| rs10474031 | circRNAs |  | N | 7.42E-06 |
| rs59978327 | circRNAs,CIRs | WHSC1L1 | N | 7.42E-06 |
| rs10245589 | circRNAs |  | Y | 7.43E-06 |
| rs9844736 | TFBRs | TMEM110-MUSTN1,TMEM110 | Y | 7.43E-06 |
| rs12056642 | circRNAs |  | Y | 7.45E-06 |
| rs10984543 | TADs | CDK5RAP2 | N | 7.45E-06 |
| rs28461911 | circRNAs |  | N | 7.45E-06 |
| rs11227447 | CIRs | ENSG00000254458 | N | 7.45E-06 |
| rs169974 | circRNAs,TADs | OPRM1,SYNE1,IPCEF1,CCDC170 | Y | 7.46E-06 |
| rs12075888 | circRNAs |  | N | 7.47E-06 |
| rs214973 | circRNAs,TADs | OPRM1,SYNE1,IPCEF1,CCDC170 | N | 7.47E-06 |
| rs9825679 | circRNAs |  | Y | 7.48E-06 |
| rs3893044 | TADs | ENSG00000174093,FBXL20,CDK12 | Y | 7.51E-06 |
| rs2743326 | CIRs | RBPJL,TOMM34 | N | 7.53E-06 |
| rs214974 | circRNAs,TADs | OPRM1,SYNE1,IPCEF1,CCDC170 | N | 7.55E-06 |
| rs11986274 | circRNAs,CIRs,TFBRs,lncRNAs | ENSG00000254981 | Y | 7.55E-06 |
| rs60730 | circRNAs |  | Y | 7.55E-06 |
| rs174600 | circRNAs |  | Y | 7.56E-06 |
| rs70015 | circRNAs,TADs | OPRM1,SYNE1,IPCEF1,CCDC170 | N | 7.56E-06 |
| rs70016 | circRNAs,TADs | OPRM1,SYNE1,IPCEF1,CCDC170 | N | 7.56E-06 |
| rs13306109 | circRNAs |  | N | 7.58E-06 |
| rs108499 | circRNAs,TADs | RTN3 | Y | 7.59E-06 |
| rs70017 | circRNAs,TADs | OPRM1,SYNE1,IPCEF1,CCDC170 | N | 7.59E-06 |
| rs2270197 | TADs | VPRBP | Y | 7.6E-06 |
| rs999895 | circRNAs,CIRs | KMT2E,,LINC01004 | N | 7.61E-06 |
| rs56300324 | circRNAs |  | Y | 7.62E-06 |
| rs10888695 | circRNAs |  | Y | 7.62E-06 |
| rs1984412 | circRNAs |  | N | 7.62E-06 |
| rs35715914 | CIRs | HIST1H2BL | Y | 7.63E-06 |
| rs6416962 | circRNAs,CIRs | MBTD1 | Y | 7.63E-06 |
| rs7007097 | circRNAs,CIRs,lncRNAs | PPAPDC1B | Y | 7.65E-06 |
| rs2235226 | CIRs,TFBRs | MATN4,SDC4 | N | 7.65E-06 |
| rs7581349 | TFBRs | ENSG00000226791 | N | 7.65E-06 |
| rs4065985 | TADs,lncRNAs | ENSG00000174093,FBXL20,CDK12 | Y | 7.69E-06 |
| rs12122269 | TADs | LPHN2,GIPC2 | N | 7.69E-06 |
| rs2372734 | circRNAs |  | N | 7.69E-06 |
| rs60558877 | circRNAs,CIRs,TFBRs,lncRNAs | L2HGDH,ATP5S,WHSC1L1,SNORD43,COQ5,ENSG00000272092 | Y | 7.7E-06 |
| rs12401738 | lncRNAs |  | Y | 7.71E-06 |
| rs886131 | TADs,lncRNAs | SVOP,CKAP4,SH2B3,TCHP | N | 7.72E-06 |
| rs10857336 | circRNAs |  | N | 7.73E-06 |
| rs2741501 | CIRs | RBPJL,TOMM34 | N | 7.77E-06 |
| rs9831116 | TADs | LINC00882 | N | 7.78E-06 |
| rs6504698 | circRNAs,CIRs | MBTD1 | Y | 7.79E-06 |
| rs62021209 | TFBRs | NMB | Y | 7.82E-06 |
| rs62068170 | TADs | ENSG00000174093,FBXL20,CDK12 | Y | 7.82E-06 |
| rs73188305 | circRNAs |  | N | 7.83E-06 |
| rs7711020 | circRNAs |  | N | 7.83E-06 |
| rs3778377 | circRNAs |  | N | 7.85E-06 |
| rs71559051 | CIRs,TFBRs | TAF11,HIST1H2AC,ZSCAN16-AS1,ANKS1A,HIST1H2BM,HIST1H4K,TXLNG,HIST1H2AJ | Y | 7.87E-06 |
| rs757958 | circRNAs |  | N | 7.87E-06 |
| rs62533710 | circRNAs |  | Y | 7.88E-06 |
| rs3859274 | circRNAs |  | Y | 7.88E-06 |
| rs3213676 | circRNAs |  | Y | 7.89E-06 |
| rs899130 | CIRs | PDIA4 | N | 7.9E-06 |
| rs8107058 | circRNAs |  | N | 7.91E-06 |
| rs174548 | circRNAs,CIRs | FADS2 | Y | 7.92E-06 |
| rs2380760 | circRNAs,CIRs | PC | N | 7.93E-06 |
| rs62533713 | circRNAs |  | Y | 7.93E-06 |
| rs214976 | circRNAs,TADs | OPRM1,SYNE1,IPCEF1,CCDC170 | Y | 7.93E-06 |
| rs41273471 | circRNAs |  | N | 7.94E-06 |
| rs11786625 | circRNAs |  | N | 7.94E-06 |
| rs10777959 | circRNAs |  | N | 7.96E-06 |
| rs7543272 | circRNAs |  | Y | 7.98E-06 |
| rs3742987 | circRNAs,TADs,miRNA target site | EPB42,CDAN1 | N | 7.98E-06 |
| rs2287250 | circRNAs |  | Y | 7.99E-06 |
| rs2344714 | circRNAs |  | N | 7.99E-06 |
| rs10791845 | circRNAs,CIRs | PACS1,,ENSG00000255038 | Y | 8E-06 |
| rs17655544 | lncRNAs |  | Y | 8.01E-06 |
| rs2287249 | circRNAs |  | Y | 8.02E-06 |
| rs326340 | circRNAs |  | N | 8.02E-06 |
| rs2010691 | circRNAs |  | N | 8.03E-06 |
| rs11984651 | circRNAs |  | N | 8.03E-06 |
| rs57172187 | circRNAs |  | Y | 8.03E-06 |
| rs3115319 | CIRs | SEPHS1P7 | N | 8.05E-06 |
| rs11987153 | circRNAs |  | N | 8.05E-06 |
| rs12677006 | circRNAs |  | N | 8.05E-06 |
| rs2635210 | TADs | ENSG00000236922 | Y | 8.06E-06 |
| rs12681483 | circRNAs |  | N | 8.06E-06 |
| rs12681501 | circRNAs |  | N | 8.06E-06 |
| rs13277090 | circRNAs |  | N | 8.07E-06 |
| rs326327 | circRNAs |  | N | 8.08E-06 |
| rs9549717 | circRNAs |  | Y | 8.08E-06 |
| rs74762181 | TADs | CDK5RAP2 | N | 8.09E-06 |
| rs2843744 | circRNAs,TFBRs | ENSG00000253739 | Y | 8.09E-06 |
| rs11701561 | circRNAs |  | N | 8.1E-06 |
| rs13391813 | TADs | DPP10 | N | 8.1E-06 |
| rs2635340 | TADs | ENSG00000236922 | N | 8.11E-06 |
| rs78132606 | circRNAs |  | N | 8.12E-06 |
| rs34540281 | TADs | JUP,ACACA | Y | 8.12E-06 |
| rs10206960 | TADs | DPP10 | N | 8.12E-06 |
| rs10224564 | circRNAs |  | N | 8.13E-06 |
| rs327183 | circRNAs |  | N | 8.13E-06 |
| rs2287244 | circRNAs |  | Y | 8.16E-06 |
| rs12464093 | circRNAs |  | Y | 8.17E-06 |
| rs13021208 | circRNAs,TADs | ASXL2,PLB1,ENSG00000231200,DPYSL5,BIRC6,ITSN2,ALK,KLHL29,ENSG00000233862,WDR35,DNAJC27-AS1,MAPRE3,ATAD2B | N | 8.17E-06 |
| rs4273084 | circRNAs |  | Y | 8.17E-06 |
| rs10410775 | circRNAs,CIRs | DNM2,MIR638 | N | 8.2E-06 |
| rs10739537 | TADs | CDK5RAP2,ASTN2 | N | 8.2E-06 |
| rs12121630 | circRNAs |  | N | 8.2E-06 |
| rs255371 | circRNAs |  | Y | 8.21E-06 |
| rs34706883 | TADs,CIRs,TFBRs | ENSG00000251665,HIST1H2BG,HIST1H2AE,CCNL1,DNAH8,ENSG00000247011,ENSG00000258651,HIST1H2BM,HIST1H2BN,HIST1H4A,NUBP2,MIR1908,HIST1H3B,HIST1H4C,HIST1H2AK,HIST1H2AJ,TPR,C1orf27 | Y | 8.21E-06 |
| rs7719373 | circRNAs |  | N | 8.22E-06 |
| rs6740446 | TADs | DPP10 | N | 8.23E-06 |
| rs1038094 | lncRNAs |  | Y | 8.24E-06 |
| rs10279602 | circRNAs |  | N | 8.25E-06 |
| rs2305481 | circRNAs,TADs,CIRs | ENSG00000174093,GSDMA | Y | 8.28E-06 |
| rs12705299 | circRNAs |  | N | 8.28E-06 |
| rs1016388 | circRNAs |  | N | 8.28E-06 |
| rs72845070 | circRNAs |  | N | 8.29E-06 |
| rs875567 | circRNAs |  | N | 8.29E-06 |
| rs56280801 | circRNAs |  | Y | 8.3E-06 |
| rs6087607 | circRNAs |  | Y | 8.3E-06 |
| rs8027427 | circRNAs,TADs,CIRs | EPB42,CDAN1 | N | 8.31E-06 |
| rs9603814 | circRNAs |  | Y | 8.31E-06 |
| rs217318 | circRNAs,TADs | IBTK,SNAP91,DOPEY1 | N | 8.31E-06 |
| rs12671368 | circRNAs |  | Y | 8.32E-06 |
| rs11802661 | lncRNAs |  | N | 8.32E-06 |
| rs2956395 | CIRs,TFBRs | ENSG00000255553,MIR611,MYRF,FADS2,SHANK2,FEN1 | N | 8.33E-06 |
| rs62078931 | TADs,CIRs | HDAC5,ASB16-AS1 | Y | 8.34E-06 |
| rs28549876 | circRNAs,TADs,CIRs | EPB42,CDAN1 | N | 8.34E-06 |
| rs2071507 | TADs | VPRBP | Y | 8.34E-06 |
| rs1494497 | TADs | LINC00882 | N | 8.35E-06 |
| rs36013793 | circRNAs |  | Y | 8.35E-06 |
| rs2159102 | circRNAs |  | N | 8.35E-06 |
| rs8026233 | circRNAs,TADs,CIRs | EPB42,CDAN1 | N | 8.36E-06 |
| rs62029585 | TADs | SH3GL3,ENSG00000259986,ENSG00000166503,ADAMTS7P4 | Y | 8.37E-06 |
| rs1470840 | circRNAs |  | N | 8.37E-06 |
| rs1563953 | circRNAs |  | N | 8.38E-06 |
| rs8041103 | circRNAs |  | Y | 8.39E-06 |
| rs13212651 | TADs,CIRs,TFBRs | HIST1H2AC,HIST1H3J,HIST1H2BC,HIST1H2BG,HIST1H2AE,WARS2,DARS2,DNAH8,ZNF165,HIST1H2BO,HIST1H2BN,HIST1H4A,ENSG00000231365,HIST1H4C,HIST1H2AK,HIST1H2AM | Y | 8.4E-06 |
| rs4265781 | circRNAs,TADs,TFBRs | EPB42,CDAN1 | N | 8.41E-06 |
| rs9198 | circRNAs,CIRs | PPAPDC1B | Y | 8.41E-06 |
| rs12530888 | circRNAs,TADs | MPP6 | Y | 8.41E-06 |
| rs13192965 | circRNAs |  | Y | 8.41E-06 |
| rs10423311 | circRNAs |  | N | 8.42E-06 |
| rs11227448 | CIRs | ENSG00000254458 | Y | 8.42E-06 |
| rs2216107 | CIRs,TFBRs | KMT2E-AS1,ENSG00000212195,ENSG00000261135 | N | 8.42E-06 |
| rs6984464 | circRNAs |  | Y | 8.43E-06 |
| rs12078434 | circRNAs,TADs | FAF1 | N | 8.43E-06 |
| rs2721817 | circRNAs,TADs | MPP6 | Y | 8.46E-06 |
| rs1331591 | TADs | CDK5RAP2 | Y | 8.46E-06 |
| rs492233 | circRNAs,TADs | SYNE1,IPCEF1,RGS17,TFB1M | N | 8.46E-06 |
| rs12083078 | circRNAs |  | Y | 8.47E-06 |
| rs9749627 | TADs | DPP10 | N | 8.47E-06 |
| rs12130132 | lncRNAs |  | N | 8.48E-06 |
| rs7844851 | circRNAs |  | N | 8.48E-06 |
| rs67101035 | circRNAs,CIRs | HIST1H2AC,ZSCAN16-AS1,ANKS1A,RBM23,HIST1H4K,BACH2,KLF12,VIM,TXLNG,RNU1-122P,TAF11,HIST1H2BM,UBR7,PRMT5-AS1,C14orf142,HIST1H2AJ | Y | 8.49E-06 |
| rs2385556 | circRNAs,TFBRs | RNU6-1322P | N | 8.49E-06 |
| rs34194357 | TADs | DNAH8 | Y | 8.5E-06 |
| rs36116761 | TADs | DNAH8 | Y | 8.5E-06 |
| rs13209332 | CIRs | RNU7-26P,HIST1H2AK | Y | 8.51E-06 |
| rs9894098 | circRNAs |  | Y | 8.51E-06 |
| rs2288867 | circRNAs,TADs | ZNF208,ENSG00000267383,ENSG00000141979 | Y | 8.52E-06 |
| rs6538902 | circRNAs |  | Y | 8.53E-06 |
| rs2282568 | circRNAs |  | Y | 8.53E-06 |
| rs71559050 | CIRs | ENSG00000235358 | Y | 8.54E-06 |
| rs35848276 | CIRs | HIST1H2AK | Y | 8.56E-06 |
| rs5995269 | circRNAs |  | Y | 8.57E-06 |
| rs4869118 | circRNAs |  | Y | 8.58E-06 |
| rs55990877 | circRNAs,TADs | RSU1,ST8SIA6 | N | 8.58E-06 |
| rs2063064 | circRNAs |  | N | 8.59E-06 |
| rs59498392 | circRNAs,TFBRs | ENSG00000254981 | Y | 8.6E-06 |
| rs10783299 | CIRs,TFBRs | WNT1,ENSG00000257913,ENSG00000272822,RNU6-940P,ENSG00000258101,PRKAG1,ARF3 | Y | 8.62E-06 |
| rs72819373 | TADs | ITK,ENSG00000250061 | N | 8.62E-06 |
| rs115277297 | TADs | LINC00882,MORC1,LINC00636 | N | 8.64E-06 |
| rs10260346 | circRNAs |  | N | 8.66E-06 |
| rs1738438 | circRNAs,TADs | SYNE1,IPCEF1,RGS17,TFB1M | N | 8.66E-06 |
| rs12998770 | circRNAs,TADs | ASXL2,PLB1,ENSG00000231200,DPYSL5,BIRC6,ITSN2,ALK,KLHL29,ENSG00000233862,WDR35,DNAJC27-AS1,MAPRE3,ATAD2B | N | 8.67E-06 |
| rs7744910 | circRNAs |  | N | 8.68E-06 |
| rs4730069 | circRNAs |  | N | 8.69E-06 |
| rs186717631 | TADs | ENSG00000229618,DGKB,ZPBP,ENSG00000230333,ABCB5 | N | 8.7E-06 |
| rs11998592 | circRNAs |  | N | 8.71E-06 |
| rs4322911 | circRNAs |  | Y | 8.72E-06 |
| rs72898950 | circRNAs |  | N | 8.73E-06 |
| rs60960031 | TADs | ENSG00000231031,ENSG00000233005,C2orf43 | N | 8.75E-06 |
| rs70018 | circRNAs,TADs | OPRM1,SYNE1,IPCEF1,CCDC170 | Y | 8.75E-06 |
| rs7502233 | TADs,CIRs,TFBRs | JUP,ACACA,MED24,CASC3 | Y | 8.76E-06 |
| rs4558594 | TADs | DPP10 | N | 8.76E-06 |
| rs10818317 | TADs | CDK5RAP2 | Y | 8.77E-06 |
| rs958628 | circRNAs |  | N | 8.79E-06 |
| rs453091 | circRNAs |  | N | 8.8E-06 |
| rs2162973 | circRNAs |  | N | 8.81E-06 |
| rs10101301 | circRNAs |  | Y | 8.82E-06 |
| rs214969 | circRNAs,TADs | OPRM1,SYNE1,IPCEF1,CCDC170 | Y | 8.82E-06 |
| rs10818314 | TADs | CDK5RAP2 | N | 8.84E-06 |
| rs9675302 | circRNAs,CIRs | UTP18,ENSG00000264895 | Y | 8.84E-06 |
| rs7222463 | circRNAs |  | Y | 8.85E-06 |
| rs16887324 | circRNAs |  | Y | 8.85E-06 |
| rs6088688 | circRNAs |  | N | 8.86E-06 |
| rs61847646 | circRNAs |  | N | 8.87E-06 |
| rs12055997 | circRNAs |  | N | 8.87E-06 |
| rs34718920 | CIRs,TFBRs | MIR3143,HIST1H2BK,HIST1H2BJ,HIST1H2AH,HIST1H2AJ,HIST1H4I | Y | 8.89E-06 |
| rs35982103 | CIRs | HIST1H2AG | Y | 8.9E-06 |
| rs12898924 | circRNAs |  | Y | 8.9E-06 |
| rs111949078 | TADs | PKHD1L1 | N | 8.91E-06 |
| rs4808976 | circRNAs |  | N | 8.91E-06 |
| rs3933088 | circRNAs |  | N | 8.92E-06 |
| rs326333 | circRNAs |  | N | 8.94E-06 |
| rs720022 | circRNAs |  | N | 8.94E-06 |
| rs9643870 | circRNAs |  | Y | 8.94E-06 |
| rs1567121 | circRNAs |  | N | 8.96E-06 |
| rs17573792 | TADs | CDK5RAP2,ASTN2 | Y | 8.96E-06 |
| rs17175814 | circRNAs,TFBRs,lncRNAs | RPS20P22 | N | 8.96E-06 |
| rs2721800 | circRNAs,TADs | MPP6 | Y | 8.96E-06 |
| rs10244764 | circRNAs |  | N | 8.96E-06 |
| rs1122174 | circRNAs,CIRs | AHCY,DYNLRB1,ENSG00000206582 | Y | 8.97E-06 |
| rs12265850 | circRNAs,TADs | RSU1,ST8SIA6 | N | 8.97E-06 |
| rs7424436 | TADs | STAT4 | N | 8.98E-06 |
| rs12089568 | circRNAs |  | N | 8.98E-06 |
| rs6718522 | TADs | DPP10 | N | 8.98E-06 |
| rs6493063 | circRNAs |  | N | 8.99E-06 |
| rs6908747 | circRNAs,TADs | OPRM1,SYNE1,IPCEF1,CCDC170 | N | 9E-06 |
| rs231038 | circRNAs |  | N | 9E-06 |
| rs965130 | circRNAs |  | N | 9.01E-06 |
| rs12945884 | circRNAs,CIRs | RN7SL735P | Y | 9.01E-06 |
| rs12674515 | circRNAs |  | Y | 9.03E-06 |
| rs10888693 | circRNAs |  | Y | 9.04E-06 |
| rs77506998 | CIRs | RHOD | N | 9.04E-06 |
| rs11205788 | circRNAs,TADs | FAF1 | N | 9.04E-06 |
| rs9371599 | circRNAs,TADs | OPRM1,SYNE1,IPCEF1,CCDC170 | N | 9.04E-06 |
| rs506181 | circRNAs,TADs | OPRM1,SYNE1,IPCEF1,CCDC170 | N | 9.06E-06 |
| rs579464 | circRNAs,TADs | OPRM1,SYNE1,IPCEF1,CCDC170 | Y | 9.06E-06 |
| rs41273465 | circRNAs |  | N | 9.06E-06 |
| rs6904183 | circRNAs,TADs | OPRM1,SYNE1,IPCEF1,CCDC170 | N | 9.08E-06 |
| rs13382645 | TADs | DPP10 | N | 9.09E-06 |
| rs9324 | TADs | VPRBP | Y | 9.09E-06 |
| rs8076651 | circRNAs |  | Y | 9.1E-06 |
| rs9294278 | circRNAs,TADs | ENSG00000271793,IBTK,SNAP91,DOPEY1 | Y | 9.1E-06 |
| rs62366246 | circRNAs |  | N | 9.12E-06 |
| rs11205727 | circRNAs |  | Y | 9.13E-06 |
| rs2237609 | circRNAs |  | N | 9.13E-06 |
| rs174534 | circRNAs,TADs | RTN3 | Y | 9.13E-06 |
| rs7841617 | circRNAs |  | Y | 9.14E-06 |
| rs929482 | circRNAs |  | Y | 9.16E-06 |
| rs4855773 | TADs,lncRNAs | LINC00882 | N | 9.17E-06 |
| rs62266392 | circRNAs,TADs | CD96,LINC00882,LSAMP,QTRTD1,BBX,ENSG00000228980,MORC1,LINC00636 | N | 9.17E-06 |
| rs3026433 | circRNAs,CIRs,TFBRs | ENSG00000272173,ENSG00000224474,PGAM1,POLR2A,BAG5,APOPT1,RNU7-1,ATP2A2,ENSG00000256500,HMGA1P3,ZBTB4,IFT81,ANAPC7 | Y | 9.18E-06 |
| rs231043 | circRNAs |  | N | 9.18E-06 |
| rs79770412 | CIRs,TFBRs | CNIH2,ENSG00000245156,ENSG00000254452 | N | 9.19E-06 |
| rs7763343 | circRNAs,TADs | OPRM1,SYNE1,IPCEF1,CCDC170 | N | 9.2E-06 |
| rs703969 | circRNAs |  | Y | 9.2E-06 |
| rs6557228 | circRNAs,TADs | OPRM1,SYNE1,IPCEF1,CCDC170 | N | 9.22E-06 |
| rs4872313 | circRNAs,TFBRs,lncRNAs | CDCA2 | Y | 9.23E-06 |
| rs11998709 | circRNAs |  | Y | 9.24E-06 |
| rs12602854 | TADs | JUP,ACACA | Y | 9.25E-06 |
| rs112968754 | circRNAs |  | N | 9.25E-06 |
| rs4644136 | circRNAs,CIRs | PSMG3-AS1 | N | 9.26E-06 |
| rs12678205 | circRNAs,CIRs,TFBRs,lncRNAs | HIST4H4,IER5L,WHSC1L1,SNORD43,COQ5,ZCCHC8,ENSG00000272092 | Y | 9.27E-06 |
| rs6119569 | circRNAs |  | Y | 9.27E-06 |
| rs214961 | circRNAs,TADs | OPRM1,SYNE1,IPCEF1,CCDC170 | N | 9.28E-06 |
| rs9577220 | circRNAs |  | Y | 9.28E-06 |
| rs6058112 | circRNAs |  | Y | 9.28E-06 |
| rs13212318 | circRNAs |  | Y | 9.28E-06 |
| rs9549366 | circRNAs |  | Y | 9.29E-06 |
| rs13023094 | circRNAs,TADs | ASXL2,PLB1,ENSG00000231200,DPYSL5,BIRC6,ITSN2,ALK,KLHL29,ENSG00000233862,WDR35,DNAJC27-AS1,MAPRE3,ATAD2B | Y | 9.29E-06 |
| rs214963 | circRNAs,TADs | OPRM1,SYNE1,IPCEF1,CCDC170 | N | 9.29E-06 |
| rs9549716 | circRNAs |  | Y | 9.29E-06 |
| rs7920657 | circRNAs |  | Y | 9.3E-06 |
| rs492921 | circRNAs,TADs | OPRM1,SYNE1,IPCEF1,CCDC170 | N | 9.3E-06 |
| rs17408134 | circRNAs,TFBRs | ENSG00000233242 | Y | 9.3E-06 |
| rs57095912 | circRNAs |  | N | 9.3E-06 |
| rs214964 | circRNAs,TADs | OPRM1,SYNE1,IPCEF1,CCDC170 | N | 9.3E-06 |
| rs508931 | circRNAs,TADs | OPRM1,SYNE1,IPCEF1,CCDC170 | N | 9.3E-06 |
| rs214962 | circRNAs,TADs | OPRM1,SYNE1,IPCEF1,CCDC170 | N | 9.31E-06 |
| rs231039 | circRNAs |  | N | 9.33E-06 |
| rs214960 | circRNAs,TADs | OPRM1,SYNE1,IPCEF1,CCDC170 | N | 9.33E-06 |
| rs9549365 | circRNAs |  | Y | 9.34E-06 |
| rs2891761 | lncRNAs |  | N | 9.35E-06 |
| rs56158970 | lncRNAs |  | N | 9.36E-06 |
| rs60290409 | circRNAs |  | N | 9.37E-06 |
| rs2283347 | TADs | SVOP,CKAP4,SH2B3,TCHP | N | 9.38E-06 |
| rs2146750 | CIRs | PCID2 | Y | 9.38E-06 |
| rs7920697 | circRNAs |  | Y | 9.39E-06 |
| rs13194781 | TADs | DNAH8 | Y | 9.39E-06 |
| rs17751184 | CIRs,TFBRs | ING5,HIST1H1C,HIST1H2BL,HIST1H2BG,HIST1H2AI,HIST1H4PS1,ENSG00000250220 | Y | 9.39E-06 |
| rs2721802 | circRNAs,TADs | MPP6 | N | 9.4E-06 |
| rs4304177 | circRNAs,TADs | OPRM1,SYNE1,IPCEF1,CCDC170 | N | 9.4E-06 |
| rs16957104 | circRNAs,TADs | EPB42 | N | 9.41E-06 |
| rs112594088 | TADs,CIRs | FER1L4,NCOA6,ASIP | N | 9.41E-06 |
| rs10953465 | circRNAs,CIRs | KMT2E,,LINC01004 | Y | 9.42E-06 |
| rs16887325 | circRNAs |  | Y | 9.42E-06 |
| rs4730078 | circRNAs |  | Y | 9.42E-06 |
| rs2023569 | circRNAs,TADs | ENSG00000271793,IBTK,SNAP91,DOPEY1 | N | 9.43E-06 |
| rs6452415 | circRNAs |  | N | 9.44E-06 |
| rs112780433 | circRNAs |  | N | 9.44E-06 |
| rs35819751 | TADs,CIRs | MIR3143,HIST1H2BK,HIST1H2AH,DNAH8 | Y | 9.44E-06 |
| rs6952534 | circRNAs |  | N | 9.44E-06 |
| rs10953466 | circRNAs,CIRs | KMT2E,,LINC01004 | N | 9.45E-06 |
| rs7639267 | circRNAs,TADs,CIRs,TFBRs | GLT8D1,,CADPS,FHIT,NT5DC2,SMIM4,CACNA2D3,CACNA2D2 | Y | 9.45E-06 |
| rs3739252 | CIRs,TFBRs,lncRNAs | PPAPDC1B | Y | 9.45E-06 |
| rs77626951 | TADs | FAM134B,ANKH | N | 9.46E-06 |
| rs77545289 | circRNAs |  | N | 9.46E-06 |
| rs1488935 | TFBRs | ENSG00000255487 | Y | 9.47E-06 |
| rs10888687 | TADs | EPS15,STIL,AGBL4-IT1,AGBL4,SMIM12,LRP8,USP24,TTC39A | N | 9.47E-06 |
| rs13032016 | TADs | DPP10 | N | 9.47E-06 |
| rs2302883 | circRNAs,CIRs | GAL3ST3,CCDC85B,HENMT1 | N | 9.48E-06 |
| rs7009732 | circRNAs |  | N | 9.48E-06 |
| rs12510659 | circRNAs |  | N | 9.49E-06 |
| rs1075653 | TADs | VPRBP | Y | 9.5E-06 |
| rs6557227 | circRNAs,TADs | OPRM1,SYNE1,IPCEF1,CCDC170 | N | 9.5E-06 |
| rs2135219 | CIRs | MIR4258,PMVK | Y | 9.5E-06 |
| rs6557226 | circRNAs,TADs | OPRM1,SYNE1,IPCEF1,CCDC170 | N | 9.5E-06 |
| rs10136429 | circRNAs |  | N | 9.5E-06 |
| rs12114353 | circRNAs |  | N | 9.51E-06 |
| rs780093 | circRNAs |  | Y | 9.53E-06 |
| rs6538901 | circRNAs |  | Y | 9.54E-06 |
| rs3132057 | CIRs | SEPHS1P7 | N | 9.54E-06 |
| rs28681082 | circRNAs,TFBRs | ENSG00000254981 | Y | 9.55E-06 |
| rs623271 | circRNAs,TADs | STARD9,ENSG00000259536 | Y | 9.55E-06 |
| rs231041 | circRNAs |  | N | 9.56E-06 |
| rs72898953 | circRNAs |  | N | 9.58E-06 |
| rs13199772 | TADs,CIRs,TFBRs | HIST1H2AB,HIST1H2BC,HIST1H1E,HIST1H2BPS2,RNU5E-1,HIST1H2BJ,RNU5B-1,HIST1H3C,DNAH8 | Y | 9.59E-06 |
| rs10760038 | TADs | CDK5RAP2,ASTN2 | N | 9.59E-06 |
| rs6577031 | circRNAs |  | Y | 9.6E-06 |
| rs7502539 | TADs,CIRs,TFBRs | JUP,ACACA,MED24 | N | 9.6E-06 |
| rs34965299 | CIRs | HIST1H2AK | Y | 9.62E-06 |
| rs7948839 | circRNAs,CIRs | RBM4B,PC | Y | 9.62E-06 |
| rs6877102 | circRNAs |  | N | 9.62E-06 |
| rs2635240 | TADs | ENSG00000236922 | N | 9.63E-06 |
| rs6925067 | circRNAs,TADs,miRNA target site | OPRM1,SYNE1,IPCEF1,CCDC170 | N | 9.63E-06 |
| rs9549685 | circRNAs |  | Y | 9.64E-06 |
| rs2016875 | circRNAs,CIRs,TFBRs | ENSG00000254981 | Y | 9.65E-06 |
| rs2234549 | circRNAs |  | Y | 9.65E-06 |
| rs12917018 | circRNAs,TADs,CIRs | EPB42,CDAN1 | Y | 9.67E-06 |
| rs2314133 | circRNAs |  | N | 9.67E-06 |
| rs6557842 | circRNAs |  | N | 9.68E-06 |
| rs7247142 | TADs | ZNF208,ENSG00000267383,ENSG00000141979 | N | 9.69E-06 |
| rs6538900 | circRNAs |  | N | 9.71E-06 |
| rs61881638 | circRNAs |  | N | 9.74E-06 |
| rs17709180 | circRNAs |  | Y | 9.74E-06 |
| rs12573187 | circRNAs |  | N | 9.75E-06 |
| rs11985168 | circRNAs |  | Y | 9.75E-06 |
| rs2581805 | circRNAs |  | Y | 9.76E-06 |
| rs2581797 | circRNAs |  | Y | 9.76E-06 |
| rs61993167 | circRNAs |  | N | 9.77E-06 |
| rs16887377 | circRNAs |  | Y | 9.78E-06 |
| rs7771568 | circRNAs,TADs | OPRM1,SYNE1,IPCEF1,CCDC170 | N | 9.8E-06 |
| rs4318888 | circRNAs,TADs | OPRM1,SYNE1,IPCEF1,CCDC170 | Y | 9.8E-06 |
| rs12074982 | circRNAs,TADs | FAF1 | N | 9.8E-06 |
| rs9549691 | circRNAs,CIRs,TFBRs | CUL4A,PROZ,ENSG00000259438 | Y | 9.81E-06 |
| rs28674538 | circRNAs |  | Y | 9.81E-06 |
| rs141752034 | TADs | RSF1,MAP6 | N | 9.82E-06 |
| rs17763089 | TADs,CIRs,TFBRs | HIST1H2AB,HIST1H2BC,HIST1H1E,HIST1H1B,RNU5E-1,HIST1H3C,ENSG00000261135,C9orf41,DNAH8,RNU1-1 | Y | 9.83E-06 |
| rs2324447 | circRNAs,TADs | ENSG00000271793,IBTK,SNAP91,DOPEY1 | N | 9.84E-06 |
| rs12666949 | circRNAs |  | N | 9.85E-06 |
| rs12601749 | TADs,CIRs | ENSG00000174093,FBXL20,LRRC3C,CDK12 | Y | 9.86E-06 |
| rs1033656 | circRNAs,TADs | ENSG00000271793,IBTK,SNAP91,DOPEY1 | N | 9.86E-06 |
| rs174560 | circRNAs,CIRs,TFBRs,lncRNAs | MIR1908,ANAPC4,KCTD20,PXT1 | Y | 9.87E-06 |
| rs2280847 | circRNAs |  | Y | 9.87E-06 |
| rs9549352 | circRNAs,CIRs | PROZ | Y | 9.88E-06 |
| rs12246530 | circRNAs,TADs | RSU1,ST8SIA6 | Y | 9.89E-06 |
| rs13199906 | TADs,CIRs,TFBRs | HIST1H2AB,HIST1H2BC,HIST1H1E,HIST1H2BPS2,RNU5E-1,HIST1H2BJ,RNU5B-1,HIST1H3C,DNAH8 | Y | 9.89E-06 |
| rs1599918 | circRNAs |  | Y | 9.9E-06 |
| rs7261964 | circRNAs |  | Y | 9.93E-06 |
| rs4710074 | circRNAs |  | Y | 9.95E-06 |
| rs12600745 | circRNAs,TADs,CIRs | SPAG9,CA10,PLEKHM1,LRRC37A3 | Y | 9.97E-06 |
| rs60647227 | TADs,CIRs | IGSF9,AIM2 | N | 9.98E-06 |
| rs6791926 | TADs | OPA1,FGF12,LEPREL1,TPRG1,OSTN | N | 9.98E-06 |
| rs17695758 | TADs,CIRs | HIST1H2BC,DNAH8 | Y | 9.99E-06 |

**Abbreviation:** transcription factor binding regions (**TFBRs**); chromatin interactive regions (**CIRs**); long non-coding RNAs regions (**lncRNAs**); topologically associated domains (**TADs**); circular RNAs regions (**circRNAs**); yes(**Y**); no (**N**)
