# Supplementary material for: An integrative analysis of genome-wide association study and regulatory SNP annotation datasets identified candidate genes for bipolar disorder
Source: Int J Bipolar Disord. 2020 Feb 3;8:6. doi: 10.1186/s40345-019-0170-z (PMC6995798; doi:10.1186/s40345-019-0170-z)
Supplement: Supplementary file 2 — Additional file 2: Table S2. rSNP functional annotation analysis results of BD GWAS dataset 2. [file 40345_2019_170_MOESM2_ESM.docx]

Table S2. rSNP functional annotation analysis results of BD GWAS dataset 2

| **SNP** | **SNP-related regulatory elements** | **element-gene (E-G) pairs** | **eQTL^*^** | **P** |
| --- | --- | --- | --- | --- |
| rs9371601 | circRNAs, TADs | OPRM1,SYNE1,IPCEF1,CCDC170 | N | 4.33E-09 |
| rs6557230 | circRNAs, TADs | OPRM1,SYNE1,IPCEF1,CCDC170 | N | 5.18E-09 |
| rs6557229 | circRNAs, TADs | OPRM1,SYNE1,IPCEF1,CCDC170 | N | 5.28E-09 |
| rs4318888 | circRNAs, TADs | OPRM1,SYNE1,IPCEF1,CCDC170 | N | 1.08E-08 |
| rs9383995 | circRNAs, TADs | OPRM1,SYNE1,IPCEF1,CCDC170 | N | 1.54E-08 |
| rs214976 | circRNAs, TADs | OPRM1,SYNE1,IPCEF1,CCDC170 | Y | 2.47E-08 |
| rs12576775 | circRNAs |  | N | 2.66E-08 |
| rs214952 | circRNAs, TADs | SYNE1,IPCEF1,RGS17,TFB1M | Y | 3.19E-08 |
| rs551900 | circRNAs, TADs | SYNE1,HDAC2,ENSG00000235652,RGS17,CCDC170 | Y | 3.35E-08 |
| rs4523096 | circRNAs, TADs | OPRM1,SYNE1,IPCEF1,CCDC170 | N | 3.40E-08 |
| rs1203233 | circRNAs, TADs | SYNE1,HDAC2,ENSG00000235652,RGS17,CCDC170 | Y | 3.51E-08 |
| rs20585 | circRNAs, TADs | SYNE1,HDAC2,ENSG00000235652,RGS17,CCDC170 | Y | 3.67E-08 |
| rs214944 | circRNAs, TADs | SYNE1,HDAC2,ENSG00000235652,RGS17,CCDC170 | Y | 3.71E-08 |
| rs214945 | circRNAs, TADs | SYNE1,HDAC2,ENSG00000235652,RGS17,CCDC170 | Y | 3.74E-08 |
| rs70018 | circRNAs, TADs | OPRM1,SYNE1,IPCEF1,CCDC170 | Y | 3.76E-08 |
| rs70015 | circRNAs, TADs | OPRM1,SYNE1,IPCEF1,CCDC170 | Y | 3.88E-08 |
| rs70017 | circRNAs, TADs | OPRM1,SYNE1,IPCEF1,CCDC170 | Y | 3.91E-08 |
| rs17138230 | circRNAs |  | N | 4.60E-08 |
| rs2623968 | circRNAs |  | Y | 4.73E-08 |
| rs551681 | circRNAs, TADs | SYNE1,HDAC2,ENSG00000235652,RGS17,CCDC170 | Y | 4.88E-08 |
| rs169974 | circRNAs, TADs | OPRM1,SYNE1,IPCEF1,CCDC170 | Y | 5.06E-08 |
| rs214941 | circRNAs, TADs | SYNE1,HDAC2,ENSG00000235652,RGS17,CCDC170 | Y | 6.41E-08 |
| rs214942 | circRNAs, TADs | SYNE1,HDAC2,ENSG00000235652,RGS17,CCDC170 | Y | 6.69E-08 |
| rs521514 | circRNAs, TADs | SYNE1,IPCEF1,RGS17,TFB1M | Y | 6.77E-08 |
| rs492233 | circRNAs, TADs | SYNE1,IPCEF1,RGS17,TFB1M | Y | 6.79E-08 |
| rs2623970 | circRNAs |  | Y | 6.91E-08 |
| rs525210 | circRNAs, TADs | SYNE1,IPCEF1,RGS17,TFB1M | Y | 7.09E-08 |
| rs214969 | circRNAs, TADs | OPRM1,SYNE1,IPCEF1,CCDC170 | Y | 7.12E-08 |
| rs7932890 | circRNAs |  | N | 7.26E-08 |
| rs549981 | circRNAs, TADs | SYNE1,HDAC2,ENSG00000235652,RGS17,CCDC170 | Y | 7.69E-08 |
| rs214962 | circRNAs, TADs | OPRM1,SYNE1,IPCEF1,CCDC170 | Y | 7.95E-08 |
| rs214961 | circRNAs, TADs | OPRM1,SYNE1,IPCEF1,CCDC170 | Y | 7.97E-08 |
| rs214963 | circRNAs, TADs | OPRM1,SYNE1,IPCEF1,CCDC170 | Y | 8.14E-08 |
| rs10875914 | circRNAs, CIRs | KMT2D | N | 8.27E-08 |
| rs7759578 | circRNAs |  | N | 8.28E-08 |
| rs506181 | circRNAs, TADs | OPRM1,SYNE1,IPCEF1,CCDC170 | Y | 8.39E-08 |
| rs553642 | circRNAs, TADs | SYNE1,IPCEF1,RGS17,TFB1M | Y | 8.49E-08 |
| rs10459232 | CIRs | ENSG00000258101 | N | 8.81E-08 |
| rs7969091 | TFBRs | RHEBL1 | N | 8.91E-08 |
| rs7763880 | circRNAs |  | N | 8.96E-08 |
| rs127196 | circRNAs, TADs | SYNE1,IPCEF1,RGS17,TFB1M | Y | 8.97E-08 |
| rs12290811 | circRNAs |  | N | 9.25E-08 |
| rs7756410 | circRNAs, TADs | OPRM1,SYNE1,IPCEF1,CCDC170 | N | 9.32E-08 |
| rs579464 | circRNAs, TADs | OPRM1,SYNE1,IPCEF1,CCDC170 | Y | 9.48E-08 |
| rs488673 | circRNAs, TADs | SYNE1,HDAC2,ENSG00000235652,RGS17,CCDC170 | Y | 9.62E-08 |
| rs177330 | circRNAs, TADs | SYNE1,IPCEF1,RGS17,TFB1M | Y | 9.82E-08 |
| rs2449116 | circRNAs |  | Y | 9.92E-08 |
| rs490448 | circRNAs, TADs | SYNE1,IPCEF1,RGS17,TFB1M | Y | 1.01E-07 |
| rs215001 | circRNAs, TADs | OPRM1,SYNE1,IPCEF1,CCDC170 | Y | 1.04E-07 |
| rs17138171 | circRNAs |  | N | 1.06E-07 |
| rs1949962 | circRNAs |  | Y | 1.15E-07 |
| rs214943 | circRNAs, TADs | SYNE1,HDAC2,ENSG00000235652,RGS17,CCDC170 | Y | 1.23E-07 |
| rs522437 | circRNAs, TADs | SYNE1,HDAC2,ENSG00000235652,RGS17,CCDC170 | Y | 1.23E-07 |
| rs214954 | circRNAs, TADs | SYNE1,IPCEF1,RGS17,TFB1M | Y | 1.24E-07 |
| rs11168839 | CIRs, lncRNAs | ENSG00000257346 | N | 1.24E-07 |
| rs9397512 | circRNAs |  | N | 1.27E-07 |
| rs548400 | circRNAs, TADs | SYNE1,IPCEF1,RGS17,TFB1M | Y | 1.29E-07 |
| rs12279388 | circRNAs |  | N | 1.30E-07 |
| rs1944449 | circRNAs |  | Y | 1.43E-07 |
| rs544098 | circRNAs, TADs | SYNE1,IPCEF1,RGS17,TFB1M | Y | 1.44E-07 |
| rs2746418 | circRNAs, TADs | SYNE1,IPCEF1,RGS17,TFB1M | Y | 1.54E-07 |
| rs7774755 | circRNAs |  | N | 1.57E-07 |
| rs6557226 | circRNAs, TADs | OPRM1,SYNE1,IPCEF1,CCDC170 | N | 1.62E-07 |
| rs544125 | circRNAs, TADs | SYNE1,IPCEF1,RGS17,TFB1M | Y | 1.66E-07 |
| rs2348798 | circRNAs |  | Y | 1.67E-07 |
| rs550685 | circRNAs, TADs | SYNE1,HDAC2,ENSG00000235652,RGS17,CCDC170 | Y | 1.70E-07 |
| rs9371603 | circRNAs |  | N | 1.72E-07 |
| rs3782357 | circRNAs |  | Y | 1.73E-07 |
| rs1830219 | circRNAs, lncRNAs |  | Y | 1.76E-07 |
| rs2117029 | circRNAs |  | Y | 1.79E-07 |
| rs10875915 | circRNAs, CIRs | PRKAG1 | N | 1.82E-07 |
| rs2293445 | circRNAs, CIRs | ENSG00000257913 | Y | 1.96E-07 |
| rs10875913 | circRNAs |  | N | 1.98E-07 |
| rs736408 | circRNAs, TADs, CIRs | VPRBP,ITIH3 | Y | 2.00E-07 |
| rs1527369 | circRNAs |  | Y | 2.05E-07 |
| rs2623958 | circRNAs |  | Y | 2.08E-07 |
| rs11168854 | CIRs | TUBA1B | N | 2.14E-07 |
| rs11237799 | circRNAs |  | N | 2.20E-07 |
| rs214997 | circRNAs, TADs | OPRM1,SYNE1,IPCEF1,CCDC170 | Y | 2.30E-07 |
| rs11237805 | circRNAs |  | N | 2.41E-07 |
| rs10783299 | CIRs, TFBRs | WNT1,ENSG00000257913,ENSG00000272822,RNU6-940P,  ENSG00000258101,PRKAG1,ARF3 | N | 2.53E-07 |
| rs12821008 | CIRs | RHEBL1 | N | 2.68E-07 |
| rs214993 | circRNAs, TADs | OPRM1,SYNE1,IPCEF1,CCDC170 | Y | 3.03E-07 |
| rs1738438 | circRNAs, TADs | SYNE1,IPCEF1,RGS17,TFB1M | Y | 3.06E-07 |
| rs527021 | circRNAs, TADs | OPRM1,SYNE1,IPCEF1,CCDC170 | Y | 3.51E-07 |
| rs502268 | circRNAs, TADs | OPRM1,SYNE1,IPCEF1,CCDC170 | Y | 3.60E-07 |
| rs214994 | circRNAs, TADs | OPRM1,SYNE1,IPCEF1,CCDC170 | Y | 3.67E-07 |
| rs954129 | circRNAs, lncRNAs |  | Y | 3.83E-07 |
| rs214987 | circRNAs, TADs | SYNE1,IPCEF1,RGS17,TFB1M | Y | 4.04E-07 |
| rs4509131 | circRNAs |  | N | 4.28E-07 |
| rs2623932 | circRNAs |  | Y | 4.30E-07 |
| rs12577093 | circRNAs |  | N | 4.33E-07 |
| rs2271893 | circRNAs, CIRs, TFBRs | ANKRD36,,CNNM4,LMAN2L,APPBP2 | Y | 4.59E-07 |
| rs214981 | circRNAs, TADs | SYNE1,IPCEF1,RGS17,TFB1M | Y | 4.61E-07 |
| rs12580349 | circRNAs |  | N | 5.05E-07 |
| rs214980 | circRNAs, TADs | SYNE1,IPCEF1,RGS17,TFB1M | Y | 5.19E-07 |
| rs2251219 | circRNAs, TADs, CIRs | CADPS,FHIT,RNU6-856P,CACNA2D3,CACNA2D2 | Y | 5.45E-07 |
| rs2240919 | circRNAs, TADs | VPRBP | Y | 5.50E-07 |
| rs3755799 | TADs | VPRBP | Y | 6.14E-07 |
| rs2300149 | TADs | VPRBP | Y | 6.48E-07 |
| rs10875910 | circRNAs, CIRs | ENSG00000258101 | N | 6.56E-07 |
| rs998909 | TADs, CIRs, TFBRs | CYCS,ITIH4,PBRM1,VPRBP,NEK4 | Y | 6.64E-07 |
| rs2239549 | TADs | VPRBP | Y | 6.68E-07 |
| rs2878628 | circRNAs, TADs, CIRs | CADPS,FHIT,RNU6-856P,CACNA2D3,CACNA2D2 | Y | 6.83E-07 |
| rs214978 | circRNAs, TADs, TFBRs | SYNE1,IPCEF1,RNA5SP223,RGS17,TFB1M | Y | 6.85E-07 |
| rs11130315 | circRNAs, TADs | PBRM1 | N | 7.16E-07 |
| rs10875912 | circRNAs, CIRs | TUBA1B | N | 7.25E-07 |
| rs11717836 | TADs | VPRBP | N | 7.28E-07 |
| rs11614738 | circRNAs |  | N | 7.53E-07 |
| rs6803012 | circRNAs, TADs | PBRM1 | N | 7.72E-07 |
| rs11085829 | circRNAs, CIRs | ENSG00000267610 | N | 7.85E-07 |
| rs2535629 | circRNAs, TADs | VPRBP | Y | 8.20E-07 |
| rs4926298 | circRNAs, TADs | ZNF675,NFIX,ZNF627 | N | 8.36E-07 |
| rs2289247 | circRNAs, CIRs ,TFBRs | SNORD69 | Y | 8.55E-07 |
| rs13085775 | circRNAs, TADs | PBRM1,VPRBP | N | 8.63E-07 |
| rs3755806 | circRNAs, TADs, CIRs | PBRM1,VPRBP | Y | 8.70E-07 |
| rs6762813 | circRNAs, CIRs, TFBRs | SNORD69 | N | 8.74E-07 |
| rs7611731 | circRNAs, TADs, CIRs | PBRM1 | N | 8.77E-07 |
| rs6804145 | circRNAs, TADs, CIRs | PBRM1 | N | 8.89E-07 |
| rs10783302 | circRNAs, CIRs | ENSG00000258101 | N | 8.89E-07 |
| rs1029871 | circRNAs, TADs | NEK4 | Y | 8.97E-07 |
| rs10865973 | TADs | PBRM1 | N | 9.02E-07 |
| rs2289250 | circRNAs, TADs | PBRM1 | Y | 9.29E-07 |
| rs11177 | circRNAs, CIRs, TFBRs | ENSG00000252787,PBRM1,SNORD19 | Y | 9.35E-07 |
| rs4481150 | circRNAs, TADs, CIRs | VPRBP,ITIH3 | N | 9.38E-07 |
| rs2019065 | TADs | VPRBP | Y | 9.57E-07 |
| rs2336149 | circRNAs, TADs | PBRM1 | Y | 9.73E-07 |
| rs4687548 | circRNAs, TADs | PBRM1,VPRBP | N | 9.74E-07 |
| rs1561337 | circRNAs, TADs | PBRM1,VPRBP | Y | 9.88E-07 |
| rs6617 | CIRs, TFBRs, lncRNAs | GLT8D1,,RPL41,C7orf73,SMIM4 | Y | 1.00E-06 |
| rs3733045 | circRNAs, TADs, CIRs | PBRM1,VPRBP | Y | 1.03E-06 |
| rs2268025 | circRNAs, TADs | NEK4 | Y | 1.03E-06 |
| rs2028216 | circRNAs, TADs | PBRM1 | Y | 1.04E-06 |
| rs13068293 | circRNAs, TADs | PBRM1,VPRBP | N | 1.05E-06 |
| rs3755798 | CIRs, TFBRs | GLT8D1,SMIM4 | Y | 1.05E-06 |
| rs11130312 | circRNAs, TADs | PBRM1,VPRBP | N | 1.05E-06 |
| rs13064064 | circRNAs, TADs | PBRM1,VPRBP | N | 1.06E-06 |
| rs13085895 | circRNAs, TADs | PBRM1,VPRBP | N | 1.06E-06 |
| rs1866268 | TADs, CIRs, TFBRs | KMT2E,MALAT1,ENSG00000270117,EIF4A1,PBRM1,  SNORA67,TM9SF3,LINC01004 | Y | 1.08E-06 |
| rs3774366 | circRNAs, TADs | PBRM1,VPRBP | Y | 1.08E-06 |
| rs13079063 | CIRs | ENSG00000270941 | N | 1.11E-06 |
| rs17052259 | circRNAs, TADs, CIRs | CADPS,FHIT,SMIM4,CACNA2D3,CACNA2D2 | N | 1.12E-06 |
| rs3733041 | circRNAs |  | Y | 1.12E-06 |
| rs11237796 | circRNAs, lncRNAs |  | N | 1.13E-06 |
| rs3774365 | circRNAs, TADs,CIRs | PBRM1,VPRBP | Y | 1.15E-06 |
| rs12635140 | circRNAs |  | N | 1.17E-06 |
| rs12497998 | circRNAs, TADs | NEK4 | N | 1.22E-06 |
| rs10865974 | TADs | PBRM1 | N | 1.22E-06 |
| rs2164885 | circRNAs |  | Y | 1.23E-06 |
| rs2239551 | TADs | VPRBP | Y | 1.25E-06 |
| rs2268027 | circRNAs, TADs, CIRs | NEK4 | Y | 1.26E-06 |
| rs2268023 | TADs | VPRBP | Y | 1.27E-06 |
| rs10501439 | circRNAs |  | N | 1.30E-06 |
| rs2336542 | circRNAs, TADs | PBRM1 | Y | 1.32E-06 |
| rs6805156 | circRNAs, TADs | PBRM1 | N | 1.34E-06 |
| rs4765913 | circRNAs |  | N | 1.35E-06 |
| rs3796353 | circRNAs, TADs, CIRs | CADPS,FHIT,SMIM4,CACNA2D3,CACNA2D2 | Y | 1.37E-06 |
| rs2072390 | circRNAs, TADs | NEK4 | Y | 1.37E-06 |
| rs7652191 | circRNAs, TADs | PBRM1 | N | 1.38E-06 |
| rs11720243 | circRNAs, TADs | PBRM1 | N | 1.44E-06 |
| rs931068 | circRNAs, TADs | ZNF675,NFIX,ZNF627 | Y | 1.50E-06 |
| rs1274726 | circRNAs |  | Y | 1.51E-06 |
| rs12487445 | circRNAs, TADs | PBRM1 | N | 1.52E-06 |
| rs12487591 | circRNAs, TADs, CIRs | PBRM1,VPRBP | N | 1.53E-06 |
| rs4687629 | circRNAs, TADs | PBRM1 | N | 1.55E-06 |
| rs13082208 | circRNAs, TADs | NEK4 | N | 1.58E-06 |
| rs2118540 | circRNAs, TADs | PBRM1 | Y | 1.58E-06 |
| rs544863 | circRNAs, TADs | SYNE1,IPCEF1,RGS17,TFB1M | Y | 1.60E-06 |
| rs11720159 | circRNAs, TADs | PBRM1 | N | 1.61E-06 |
| rs1961959 | circRNAs, TADs | CADPS,FHIT,CACNA2D3,CACNA2D2 | Y | 1.63E-06 |
| rs17264436 | circRNAs, TADs | PBRM1 | N | 1.64E-06 |
| rs11714419 | circRNAs, TADs | PBRM1 | N | 1.66E-06 |
| rs2336545 | circRNAs, TADs, TFBRs, lncRNAs | ENSG00000271137,NEK4 | Y | 1.70E-06 |
| rs13082960 | circRNAs, TADs | NEK4 | N | 1.80E-06 |
| rs1961958 | circRNAs, TADs | CADPS,FHIT,CACNA2D3,CACNA2D2 | Y | 1.80E-06 |
| rs10896135 | circRNAs |  | N | 1.83E-06 |
| rs2336146 | circRNAs, TADs | PBRM1 | Y | 1.85E-06 |
| rs1042779 | TADs | VPRBP | Y | 1.90E-06 |
| rs968470 | CIRs | CNNM4 | Y | 1.93E-06 |
| rs2535627 | lncRNAs |  | Y | 1.93E-06 |
| rs11714565 | circRNAs, TADs | PBRM1 | N | 1.94E-06 |
| rs10028075 | circRNAs, TADs | WDFY3,KLHL8,HERC3,MAPK10 | N | 1.95E-06 |
| rs6727384 | circRNAs |  | N | 1.96E-06 |
| rs6778844 | circRNAs, TADs | CADPS,FHIT,CACNA2D3,CACNA2D2 | N | 1.96E-06 |
| rs6798246 | circRNAs, TADs | CADPS,FHIT,CACNA2D3,CACNA2D2 | N | 1.98E-06 |
| rs10009993 | circRNAs, TADs | KLHL8,CCSER1,MAPK10,STPG2 | N | 2.02E-06 |
| rs12644947 | circRNAs, TADs | KLHL8,CCSER1,MAPK10,STPG2 | N | 2.04E-06 |
| rs2289249 | circRNAs, TADs | CADPS,FHIT,CACNA2D3,CACNA2D2 | Y | 2.04E-06 |
| rs6591222 | circRNAs |  | N | 2.06E-06 |
| rs6730773 | CIRs | CNNM4 | N | 2.06E-06 |
| rs4687550 | TADs | VPRBP | N | 2.06E-06 |
| rs9324 | TADs | VPRBP | Y | 2.06E-06 |
| rs1076425 | TADs | VPRBP | Y | 2.08E-06 |
| rs2083180 | circRNAs, TADs | PBRM1,VPRBP | Y | 2.08E-06 |
| rs12912251 | TADs, lncRNAs | PATL2,GPR176,WDR76 | N | 2.10E-06 |
| rs6810413 | circRNAs, TADs | WDFY3,KLHL8,HERC3,MAPK10 | N | 2.11E-06 |
| rs9881468 | TADs, CIRs, TFBRs | VPRBP,ITIH3 | N | 2.15E-06 |
| rs1075653 | TADs | VPRBP | Y | 2.17E-06 |
| rs2071508 | TADs | VPRBP | Y | 2.22E-06 |
| rs2270197 | TADs | VPRBP | Y | 2.25E-06 |
| rs11130323 | circRNAs, TADs | NEK4 | N | 2.28E-06 |
| rs2268026 | circRNAs, TADs | NEK4 | Y | 2.37E-06 |
| rs2623981 | circRNAs |  | Y | 2.39E-06 |
| rs2071507 | TADs | VPRBP | Y | 2.41E-06 |
| rs2071506 | TADs | VPRBP | Y | 2.55E-06 |
| rs7582249 | circRNAs |  | N | 2.60E-06 |
| rs7930203 | circRNAs, CIRs | RBM4B | N | 2.66E-06 |
| rs2304275 | circRNAs, CIRs | PRKAG1 | Y | 2.69E-06 |
| rs1318597 | circRNAs |  | Y | 2.74E-06 |
| rs2286798 | TADs | VPRBP | Y | 2.87E-06 |
| rs6976 | circRNAs |  | Y | 2.94E-06 |
| rs678 | TADs | VPRBP | Y | 3.00E-06 |
| rs4687551 | TADs | VPRBP | N | 3.07E-06 |
| rs4487330 | circRNAs, TADs | KLHL8,CCSER1,MAPK10,STPG2 | N | 3.09E-06 |
| rs4133139 | circRNAs, TADs | KLHL8,CCSER1,MAPK10,STPG2 | Y | 3.13E-06 |
| rs7165988 | TADs, TFBRs, lncRNAs | PATL2,GPR176,C15orf53,WDR76 | N | 3.21E-06 |
| rs9884770 | circRNAs, TADs | KLHL8,CCSER1,MAPK10,STPG2 | N | 3.44E-06 |
| rs7171233 | TADs, TFBRs, lncRNAs | PATL2,GPR176,C15orf53,WDR76 | N | 3.48E-06 |
| rs9995879 | circRNAs, TADs | KLHL8,CCSER1,MAPK10,STPG2 | N | 3.60E-06 |
| rs3774354 | TADs | VPRBP | Y | 3.62E-06 |
| rs4482754 | circRNAs, TADs | KLHL8,CCSER1,MAPK10,STPG2 | N | 3.62E-06 |
| rs13112959 | circRNAs, TADs | WDFY3,KLHL8,HERC3,MAPK10 | N | 3.63E-06 |
| rs1469869 | circRNAs, TADs | WDFY3,KLHL8,HERC3,MAPK10 | Y | 3.66E-06 |
| rs3774355 | TADs | VPRBP | Y | 3.69E-06 |
| rs2710323 | TADs | VPRBP | Y | 3.72E-06 |
| rs1436521 | circRNAs, TADs | KLHL8,CCSER1,MAPK10,STPG2 | Y | 3.78E-06 |
| rs11097091 | circRNAs, TADs | KLHL8,CCSER1,MAPK10,STPG2 | N | 3.81E-06 |
| rs4501213 | circRNAs, TADs | KLHL8,CCSER1,MAPK10,STPG2 | N | 3.81E-06 |
| rs7570 | circRNAs, CIRs, TFBRs | RLF,,RBM4B,ENSG00000259687,RNF6,PFDN1,ENSG00000119660,  SCARNA2,TNFAIP8,YRDC,RCE1,HNRNPAB | Y | 3.84E-06 |
| rs2287921 | circRNAs |  | Y | 4.00E-06 |
| rs12502934 | circRNAs, TADs | KLHL8,CCSER1,MAPK10,STPG2 | N | 4.16E-06 |
| rs4332037 | circRNAs |  | N | 4.28E-06 |
| rs11168751 | circRNAs |  | N | 4.34E-06 |
| rs4930390 | circRNAs |  | N | 4.36E-06 |
| rs7163869 | TADs, TFBRs, lncRNAs | PATL2,GPR176,C15orf53,WDR76 | N | 4.43E-06 |
| rs6461009 | circRNAs |  | N | 4.59E-06 |
| rs7127580 | circRNAs, TADs | TENM4,JRKL,JRKL-AS1,DLG2 | N | 4.61E-06 |
| rs2281587 | circRNAs, TADs | SH3PXD2A | Y | 4.78E-06 |
| rs10747562 | CIRs, TFBRs | LMBR1L | N | 4.78E-06 |
| rs1054442 | CIRs, TFBRs | WNT1,WNT10B,ENSG00000258283,ENSG00000272822,  RNU6-940P,DDX23,ARF3 | Y | 4.80E-06 |
| rs6550435 | TADs | MOBP,CTDSPL,ENTPD3-AS1 | N | 4.80E-06 |
| rs2279596 | circRNAs, CIRs | ENSG00000258101 | Y | 4.80E-06 |
| rs11191741 | circRNAs, TADs | SH3PXD2A | N | 4.82E-06 |
| rs3775187 | circRNAs, TADs | WDFY3,KLHL8,HERC3,MAPK10 | Y | 5.02E-06 |
| rs12898460 | TADs, lncRNAs | PATL2,GPR176,WDR76 | N | 5.10E-06 |
| rs3968 | circRNAs |  | Y | 5.15E-06 |
| rs3741621 | circRNAs |  | Y | 5.16E-06 |
| rs2047101 | circRNAs, CIRs | ENSG00000258101 | Y | 5.40E-06 |
| rs10747561 | circRNAs |  | N | 5.46E-06 |
| rs9882911 | circRNAs, TADs | TRANK1,GOLGA4 | N | 5.48E-06 |
| rs10510760 | circRNAs, TADs, CIRs | PBRM1,VPRBP,SMIM4 | N | 5.63E-06 |
| rs6947019 | circRNAs |  | N | 5.85E-06 |
| rs12730292 | TADs | LPHN2,GIPC2 | N | 5.93E-06 |
| rs12902447 | circRNAs |  | N | 5.95E-06 |
| rs12615689 | lncRNAs |  | N | 6.07E-06 |
| rs9834970 | TADs | MOBP,CTDSPL,ENTPD3-AS1 | N | 6.19E-06 |
| rs875326 | lncRNAs |  | Y | 6.32E-06 |
| rs11162405 | CIRs, TFBRs | FUBP1,SERBP1,ENSG00000227963,ZZZ3,SERPINB1,  ENSG00000273338,SNORD45C,GIPC2,TAGLN2,ENSG00000269968 | N | 6.41E-06 |
| rs9821223 | circRNAs, TADs | TRANK1,GOLGA4 | N | 6.53E-06 |
| rs4678910 | circRNAs, TADs | TRANK1,GOLGA4 | N | 6.62E-06 |
| rs4765914 | circRNAs |  | N | 6.78E-06 |
| rs3617 | circRNAs, TADs | VPRBP | Y | 6.85E-06 |
| rs4234258 | circRNAs, TADs | MOBP,CTDSPL,ENTPD3-AS1 | N | 7.03E-06 |
| rs12903120 | TADs, TFBRs | PATL2,GPR176,C15orf53,WDR76 | N | 7.04E-06 |
| rs2240920 | circRNAs, TADs, CIRs | VPRBP,NEK4 | Y | 7.25E-06 |
| rs7581349 | TFBRs | ENSG00000226791 | N | 7.29E-06 |
| rs4624519 | TADs | MOBP,CTDSPL,ENTPD3-AS1 | N | 7.43E-06 |
| rs2175420 | circRNAs, TADs | TENM4,JRKL,JRKL-AS1,DLG2 | Y | 7.45E-06 |
| rs4146115 | CIRs | ENSG00000204929 | Y | 7.52E-06 |
| rs9811916 | circRNAs, TADs | MOBP,CTDSPL,ENTPD3-AS1 | N | 7.62E-06 |
| rs2172835 | TADs | PATL2,GPR176,WDR76 | Y | 7.66E-06 |
| rs10267593 | circRNAs |  | N | 7.76E-06 |
| rs1532965 | TADs | MOBP,CTDSPL,ENTPD3-AS1 | Y | 7.79E-06 |
| rs12637912 | circRNAs, TADs | MOBP,CTDSPL,ENTPD3-AS1 | N | 7.86E-06 |
| rs10774037 | circRNAs |  | N | 7.91E-06 |
| rs1108842 | CIRs, TFBRs ,lncRNAs | EID1,ENSG00000272173,RNU7-1,CCDC88A,MGEA5,  PBRM1,SPCS1,KCNIP2-AS1,TM9SF3,ENSG00000235883 | Y | 7.92E-06 |
| rs9804190 | circRNAs, TADs | PHYHIPL,ANK3 | N | 7.95E-06 |
| rs9879090 | circRNAs, TADs | PBRM1,VPRBP | N | 7.99E-06 |
| rs9283389 | circRNAs |  | N | 8.01E-06 |
| rs3732386 | circRNAs, TADs | MOBP,CTDSPL,ENTPD3-AS1 | Y | 8.03E-06 |
| rs13003991 | TFBRs | RN7SL635P | N | 8.06E-06 |
| rs2239699 | TADs, CIRs | VPRBP,ITIH3 | Y | 8.35E-06 |
| rs12275848 | circRNAs, TADs | TENM4,JRKL,JRKL-AS1,DLG2 | N | 8.49E-06 |
| rs3741194 | circRNAs, CIRs | RHOD | Y | 8.57E-06 |
| rs10974874 | circRNAs, CIRs | JAK2 | N | 8.63E-06 |
| rs12120254 | TADs | LPHN2,GIPC2 | N | 8.65E-06 |
| rs2077432 | circRNAs, CIRs | RBM4,RBM14,RBM14-RBM4 | Y | 8.67E-06 |
| rs4678909 | circRNAs, TADs | MOBP,CTDSPL,ENTPD3-AS1 | N | 8.69E-06 |
| rs548978 | circRNAs, TADs | SYNE1,HDAC2,ENSG00000235652,RGS17,CCDC170 | Y | 8.91E-06 |
| rs7652637 | circRNAs, TADs | MOBP,CTDSPL,ENTPD3-AS1 | N | 8.93E-06 |
| rs1436529 | circRNAs, TADs | WDFY3,KLHL8,HERC3,MAPK10 | Y | 9.13E-06 |
| rs7827290 | CIRs | ENSG00000271959 | N | 9.36E-06 |
| rs7035298 | circRNAs |  | N | 9.83E-06 |

**Abbreviation:** transcription factor binding regions (**TFBRs**); chromatin interactive regions (**CIRs**); long non-coding RNAs regions (**lncRNAs**); topologically associated domains (**TADs**); circular RNAs regions (**circRNAs**); yes(**Y**); no (**N**)
