# Supplementary material for: An integrative analysis of genome-wide association study and regulatory SNP annotation datasets identified candidate genes for bipolar disorder
Source: Int J Bipolar Disord. 2020 Feb 3;8:6. doi: 10.1186/s40345-019-0170-z (PMC6995798; doi:10.1186/s40345-019-0170-z)
Supplement: Supplementary file 3 — Additional file 3: Table S3. List of the common target genes shared by BD GWAS dataset 1 and dataset 2. [file 40345_2019_170_MOESM3_ESM.docx]

**Table S3.** List of the common target genes shared by BD GWAS dataset 1 and dataset 2.

| Gene | SNP-associated regulatory elements | GWAS dataset 1 | | GWAS dataset 2 | |
| --- | --- | --- | --- | --- | --- |
|  |  | SNP | P_dataset1_ | SNP | P_dataset2_ |
| IPCEF1 | TADs | rs9371601 | 1.8E-06 | rs9371601 | 4.33E-09 |
| OPRM1 | TADs | rs9371601 | 1.8E-06 | rs9371601 | 4.33E-09 |
| SYNE1 | TADs | rs9371601 | 1.8E-06 | rs9371601 | 4.33E-09 |
| RGS17 | TADs | rs215005 | 6.04E-06 | rs214952 | 3.19E-08 |
| TFB1M | TADs | rs215005 | 6.04E-06 | rs214952 | 3.19E-08 |
| CCDC170 | TADs | rs9371601 | 1.8E-06 | rs551900 | 3.35E-08 |
| ENSG00000235652 | TADs | rs548985 | 6.58E-06 | rs551900 | 3.35E-08 |
| HDAC2 | TADs | rs548985 | 6.58E-06 | rs551900 | 3.35E-08 |
| KMT2D | CIRs | rs10783301 | 3.32E-06 | rs10875914 | 8.27E-08 |
| ENSG00000258101 | CIRs,TFBRs | rs10459221 | 4.2E-07 | rs10459232 | 8.81E-08 |
| RHEBL1 | CIRs,TFBRs | rs7969091 | 3.25E-07 | rs7969091 | 8.91E-08 |
| ENSG00000257346 | CIRs,lncRNAs | rs11168839 | 2.89E-06 | rs11168839 | 1.24E-07 |
| PRKAG1 | CIRs,TFBRs | rs2304275 | 1.45E-06 | rs10875915 | 1.82E-07 |
| ENSG00000257913 | CIRs,TFBRs | rs2293445 | 6.51E-06 | rs2293445 | 1.96E-07 |
| VPRBP | TADs,CIRs,TFBRs | rs2302417 | 4.93E-09 | rs736408 | 2.00E-07 |
| ITIH3 | TADs,CIRs,TFBRs | rs4481150 | 1.09E-08 | rs736408 | 2.00E-07 |
| ENSG00000272822 | CIRs,TFBRs | rs1054442 | 3.27E-06 | rs10783299 | 2.53E-07 |
| RNU6-940P | CIRs,TFBRs | rs1054442 | 3.27E-06 | rs10783299 | 2.53E-07 |
| WNT1 | CIRs,TFBRs | rs1054442 | 3.27E-06 | rs10783299 | 2.53E-07 |
| LMAN2L | TFBRs | rs72809838 | 6.47E-09 | rs2271893 | 4.59E-07 |
| ANKRD36 | CIRs,TFBRs | rs2271893 | 2.08E-08 | rs2271893 | 4.59E-07 |
| APPBP2 | TFBRs | rs2271893 | 2.08E-08 | rs2271893 | 4.59E-07 |
| CNNM4 | CIRs,TFBRs | rs2271893 | 2.08E-08 | rs2271893 | 4.59E-07 |
| CACNA2D2 | TADs,CIRs | rs2251219 | 3.02E-07 | rs2251219 | 5.45E-07 |
| CACNA2D3 | TADs,CIRs | rs2251219 | 3.02E-07 | rs2251219 | 5.45E-07 |
| CADPS | TADs,CIRs | rs2251219 | 3.02E-07 | rs2251219 | 5.45E-07 |
| FHIT | TADs,CIRs | rs2251219 | 3.02E-07 | rs2251219 | 5.45E-07 |
| RNU6-856P | TADs,CIRs | rs2251219 | 3.02E-07 | rs2251219 | 5.45E-07 |
| NEK4 | TADs,CIRs,TFBRs,lncRNAs | rs7646741 | 9.52E-09 | rs998909 | 6.64E-07 |
| PBRM1 | TADs,CIRs,TFBRs,lncRNAs | rs7612511 | 2.27E-08 | rs998909 | 6.64E-07 |
| ITIH4 | TADs,CIRs,TFBRs | rs7652667 | 6.48E-08 | rs998909 | 6.64E-07 |
| CYCS | TADs,CIRs,TFBRs | rs998909 | 1.79E-07 | rs998909 | 6.64E-07 |
| SNORD69 | CIRs,TFBRs | rs2289247 | 3.79E-07 | rs2289247 | 8.55E-07 |
| ENSG00000252787 | CIRs,TFBRs | rs3774349 | 3.91E-08 | rs11177 | 9.35E-07 |
| SNORD19 | CIRs,TFBRs | rs3774349 | 3.91E-08 | rs11177 | 9.35E-07 |
| SMIM4 | TADs,CIRs,TFBRs,lncRNAs | rs13083798 | 7.54E-08 | rs6617 | 1.00E-06 |
| C7orf73 | CIRs,TFBRs,lncRNAs | rs6617 | 2.55E-07 | rs6617 | 1E-06 |
| GLT8D1 | CIRs,TFBRs,lncRNAs | rs6617 | 2.55E-07 | rs6617 | 1E-06 |
| RPL41 | CIRs,TFBRs,lncRNAs | rs6617 | 2.55E-07 | rs6617 | 1E-06 |
| ENSG00000270117 | TADs,CIRs,TFBRs | rs1866268 | 3.38E-07 | rs1866268 | 1.08E-06 |
| EIF4A1 | TADs,CIRs, TFBRs | rs1866268 | 3.38E-07 | rs1866268 | 1.08E-06 |
| KMT2E | TADs,CIRs,TFBRs | rs1866268 | 3.38E-07 | rs1866268 | 1.08E-06 |
| LINC01004 | TADs,CIRs,TFBRs | rs1866268 | 3.38E-07 | rs1866268 | 1.08E-06 |
| MALAT1 | TADs,CIRs,TFBRs | rs1866268 | 3.38E-07 | rs1866268 | 1.08E-06 |
| SNORA67 | TADs,CIRs,TFBRs | rs1866268 | 3.38E-07 | rs1866268 | 1.08E-06 |
| ENSG00000270941 | CIRs | rs767418 | 3.68E-07 | rs13079063 | 1.11E-06 |
| ENSG00000271137 | TADs,TFBRs,lncRNAs | rs2336545 | 5.56E-07 | rs2336545 | 1.7E-06 |
| CCSER1 | TADs | rs11097326 | 1.76E-06 | rs10009993 | 2.02E-06 |
| GPR176 | TADs,TFBRs,lncRNAs | rs12898460 | 6.35E-08 | rs12912251 | 2.10E-06 |
| PATL2 | TADs,TFBRs,lncRNAs | rs12898460 | 6.35E-08 | rs12912251 | 2.10E-06 |
| WDR76 | TADs,TFBRs,lncRNAs | rs12898460 | 6.35E-08 | rs12912251 | 2.10E-06 |
| RBM4 | CIRs | rs6591223 | 7.42E-07 | rs7930203 | 2.66E-06 |
| RBM4B | CIRs,TFBRs | rs6591223 | 7.42E-07 | rs7930203 | 2.66E-06 |
| RCE1 | TFBRs | rs6591223 | 7.42E-07 | rs7570 | 3.84E-06 |
| HNRNPAB | TFBRs | rs2965186 | 4.48E-07 | rs7570 | 3.84E-06 |
| DLG2 | TADs | rs73496688 | 1.05E-08 | rs7127580 | 4.61E-06 |
| JRKL | TADs | rs73496688 | 1.05E-08 | rs7127580 | 4.61E-06 |
| JRKL-AS1 | TADs | rs73496688 | 1.05E-08 | rs7127580 | 4.61E-06 |
| TENM4 | TADs | rs73496688 | 1.05E-08 | rs7127580 | 4.61E-06 |
| ARF3 | CIRs,TFBRs | rs1054442 | 3.27E-06 | rs1054442 | 4.8E-06 |
| DDX23 | CIRs,TFBRs | rs1054442 | 3.27E-06 | rs1054442 | 4.8E-06 |
| ENSG00000258283 | CIRs,TFBRs | rs1054442 | 3.27E-06 | rs1054442 | 4.8E-06 |
| WNT10B | CIRs,TFBRs | rs1054442 | 3.27E-06 | rs1054442 | 4.8E-06 |
| CTDSPL | TADs | rs9834970 | 5.53E-14 | rs6550435 | 4.80E-06 |
| ENTPD3-AS1 | TADs | rs9834970 | 5.53E-14 | rs6550435 | 4.80E-06 |
| MOBP | TADs | rs9834970 | 5.53E-14 | rs6550435 | 4.80E-06 |
| GOLGA4 | TADs | rs9863798 | 1.14E-09 | rs9882911 | 5.48E-06 |
| TRANK1 | TADs | rs9863798 | 1.14E-09 | rs9882911 | 5.48E-06 |
| GIPC2 | TADs | rs11162556 | 1.83E-06 | rs12730292 | 5.93E-06 |
| LPHN2 | TADs | rs11162556 | 1.83E-06 | rs12730292 | 5.93E-06 |
| C15orf53 | TADs,TFBRs,lncRNAs | rs12903120 | 1.07E-07 | rs12903120 | 7.04E-06 |
| ENSG00000226791 | TFBRs | rs7581349 | 7.65E-06 | rs7581349 | 7.29E-06 |
| ENSG00000204929 | CIRs | rs4146115 | 4.09E-06 | rs4146115 | 7.52E-06 |
| ENSG00000272173 | CIRs,TFBRs,lncRNAs | rs1108842 | 4.01E-08 | rs1108842 | 7.92E-06 |
| CCDC88A | CIRs,TFBRs,lncRNAs | rs1108842 | 4.01E-08 | rs1108842 | 7.92E-06 |
| EID1 | CIRs,TFBRs,lncRNAs | rs1108842 | 4.01E-08 | rs1108842 | 7.92E-06 |
| ENSG00000235883 | CIRs,TFBRs,lncRNAs | rs1108842 | 4.01E-08 | rs1108842 | 7.92E-06 |
| KCNIP2-AS1 | CIRs,TFBRs,lncRNAs | rs1108842 | 4.01E-08 | rs1108842 | 7.92E-06 |
| MGEA5 | CIRs,TFBRs,lncRNAs | rs1108842 | 4.01E-08 | rs1108842 | 7.92E-06 |
| RNU7-1 | CIRs,TFBRs,lncRNAs | rs1108842 | 4.01E-08 | rs1108842 | 7.92E-06 |
| SPCS1 | CIRs,TFBRs,lncRNAs | rs1108842 | 4.01E-08 | rs1108842 | 7.92E-06 |
| TM9SF3 | TADs,CIRs,TFBRs,lncRNAs | rs1108842 | 4.01E-08 | rs1108842 | 7.92E-06 |
| RHOD | CIRs | rs77506998 | 9.04E-06 | rs3741194 | 8.57E-06 |
| RBM14 | CIRs | rs1127894 | 3E-06 | rs2077432 | 8.67E-06 |
| RBM14-RBM4 | CIRs | rs1127894 | 3E-06 | rs2077432 | 8.67E-06 |

**Abbreviation:** transcription factor binding regions (**TFBRs**); chromatin interactive regions (**CIRs**); long non-coding RNAs regions (**lncRNAs**);

topologically associated domains (**TADs**); circular RNAs regions (**circRNAs**); yes(**Y**); no (**N**)
