# Supplementary material for: An integrative analysis of genome-wide association study and regulatory SNP annotation datasets identified candidate genes for bipolar disorder
Source: Int J Bipolar Disord. 2020 Feb 3;8:6. doi: 10.1186/s40345-019-0170-z (PMC6995798; doi:10.1186/s40345-019-0170-z)
Supplement: Supplementary file 4 — Additional file 4: Table S4. FUMA gene set enrichment analysis results. [file 40345_2019_170_MOESM4_ESM.docx]

Table S4. FUMA gene set enrichment analysis results

| **Gene Set** | **P-value** | **adjusted P** |
| --- | --- | --- |
| ***Chemical and Genetic pertubation gene sets*** | | |
| NIKOLSKY_BREAST_CANCER_11Q12_Q14_AMPLICON | 1.65E-05 | 4.97E-02 |
| LASTOWSKA_NEUROBLASTOMA_COPY_NUMBER_DN | 2.90E-05 | 4.97E-02 |
| ***All computational gene sets*** | | |
| MORF_UNG | 1.66E-05 | 1.43E-02 |
| MORF_MTA1 | 5.29E-05 | 2.27E-02 |
| MORF_EIF3S2 | 1.30E-04 | 3.36E-02 |
| MORF_HDAC1 | 1.57E-04 | 3.36E-02 |
| MORF_CSNK2B | 2.75E-04 | 4.33E-02 |
| MORF_FEN1 | 3.03E-04 | 4.33E-02 |
| ***Immunologic signatures*** | | |
| GSE19401_PLN_VS_PEYERS_PATCH_FOLLICULAR_DC_UP | 2.64E-06 | 1.29E-02 |
| GSE29618_BCELL_VS_PDC_DN | 4.74E-05 | 4.96E-02 |
| GSE43955_TH0_VS_TGFB_IL6_TH17_ACT_CD4_TCELL_10H_UP | 4.97E-05 | 4.96E-02 |
| GSE22886_NAIVE_CD4_TCELL_VS_MEMORY_TCELL_DN | 5.09E-05 | 4.96E-02 |
| GSE3982_CTRL_VS_LPS_48H_DC_UP | 5.09E-05 | 4.96E-02 |
| ***Cancer gene neighborhoods*** | | |
| MORF_UNG | 1.66E-05 | 7.10E-03 |
| MORF_MTA1 | 5.29E-05 | 1.13E-02 |
| MORF_EIF3S2 | 1.30E-04 | 1.67E-02 |
| MORF_HDAC1 | 1.57E-04 | 1.67E-02 |
| MORF_CSNK2B | 2.75E-04 | 2.15E-02 |
| MORF_FEN1 | 3.03E-04 | 2.15E-02 |
| MORF_PRKCA | 4.79E-04 | 2.92E-02 |
| MORF_RAD23A | 6.36E-04 | 3.05E-02 |
| MORF_PRDX3 | 6.43E-04 | 3.05E-02 |
| MORF_RAC1 | 8.50E-04 | 3.63E-02 |
| MORF_FDXR | 1.08E-03 | 4.18E-02 |
| MORF_PTPN11 | 1.33E-03 | 4.73E-02 |
| MORF_PTPRB | 1.66E-03 | 4.80E-02 |
| MORF_MAGEA8 | 1.78E-03 | 4.80E-02 |
| MORF_CD8A | 2.02E-03 | 4.80E-02 |
| MORF_THPO | 2.11E-03 | 4.80E-02 |
| MORF_SOD1 | 2.30E-03 | 4.80E-02 |
| MORF_HDAC2 | 2.30E-03 | 4.80E-02 |
| MORF_MAP2K2 | 2.31E-03 | 4.80E-02 |
| MORF_FSHR | 2.33E-03 | 4.80E-02 |
| GNF2_G22P1 | 2.36E-03 | 4.80E-02 |
