# Supplementary figures and images for: An integrative analysis of genome-wide association study and regulatory SNP annotation datasets identified candidate genes for bipolar disorder
Source: Int J Bipolar Disord. 2020 Feb 3;8:6. doi: 10.1186/s40345-019-0170-z (PMC6995798; doi:10.1186/s40345-019-0170-z)

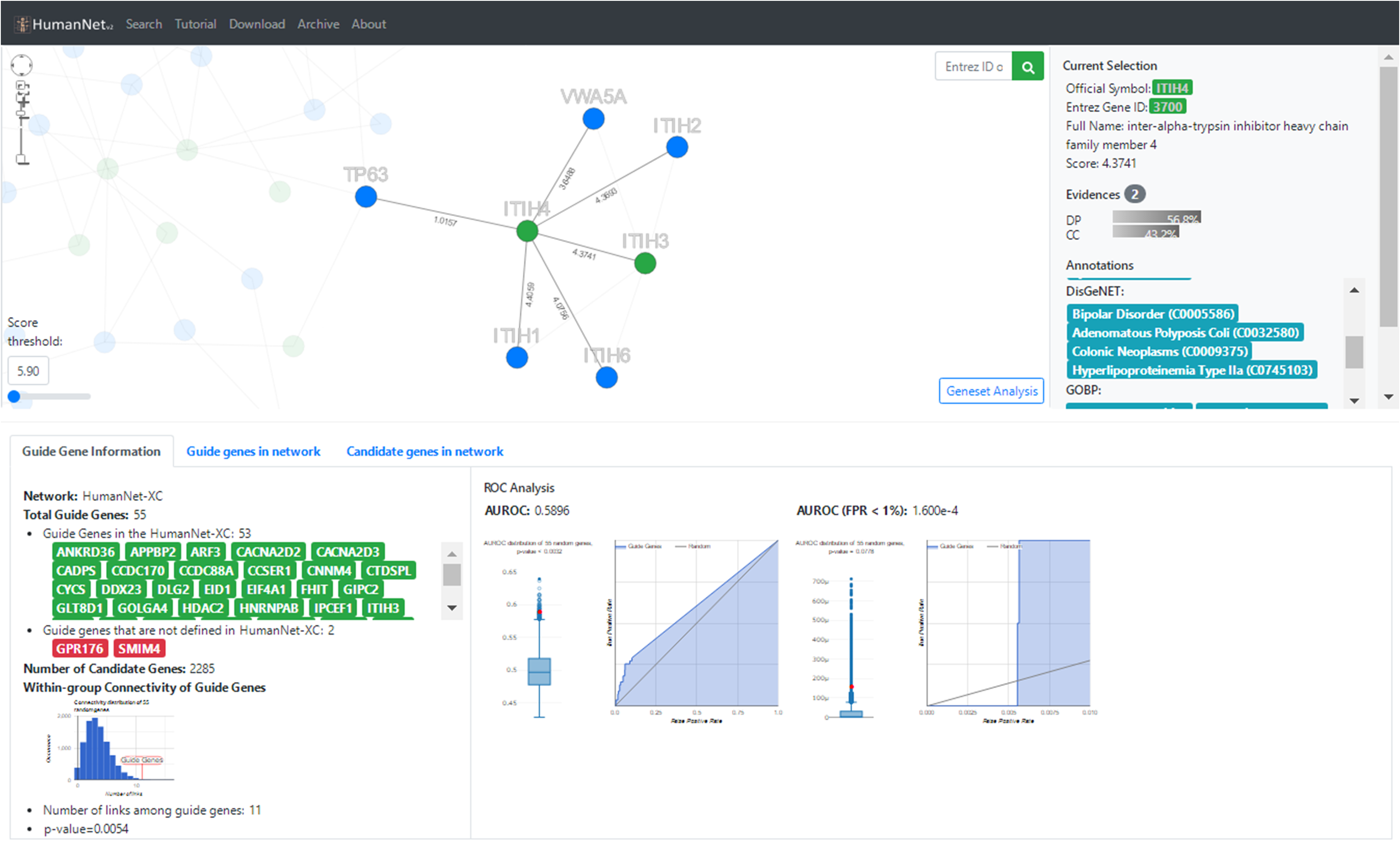

Supplement: Supplementary file 5 — Additional file 5: Figure S1. Based on the 85 common target genes as guide genes, we used HumanNet-XC to predict network-based disease gene. The upper panel shows the interactive network viewer, and a group of guide genes (green nodes), which can be annotated by their neighbors as putative candidate genes (blue nodes). The local subnetwork of the first ranked candidate, ITIH4, and its neighbors were highlighted. DISEASES and DisGeNET, serving to validate the specific prediction result, already annotate the retrieved gene ITIH4 for Bipolar Disorder. The lower panel shows the guide genes, including the statistical significance of within group connectivity of guide genes, and the observed network performance for guide gene recovery reported as receiver operating characteristic curve (ROC) curves. The area under the receiver operating characteristic curve (AUROC) indicated the predictive HumanNet-XC networks performance for a disease, which was based on the efficiency of guide gene recovery. [file 40345_2019_170_MOESM5_ESM.png]
