# Supplementary material for: An integrative analysis of genome-wide association study and regulatory SNP annotation datasets identified candidate genes for bipolar disorder
Source: Int J Bipolar Disord. 2020 Feb 3;8:6. doi: 10.1186/s40345-019-0170-z (PMC6995798; doi:10.1186/s40345-019-0170-z)
Supplement: Supplementary file 6 — Additional file 6: Table S5. List of the candidate genes identified by HumanNet-XC analysis. [file 40345_2019_170_MOESM6_ESM.docx]

Table S5. List of the candidate genes identified by HumanNet-XC analysis

| **Gene** | **Score** | **Evidences** | **Connected Queried Genes** |
| --- | --- | --- | --- |
| **EARS2** | 10.2651 | CE:0.580;GN:0.420 | CYCS;EIF4A1;DDX23;TFB1M |
| **PABPC1** | 9.741828 | LC:0.284;CC:0.265;CX:0.251;DB:0.200 | RPL41;EIF4A1;HNRNPAB;RBM4 |
| **SMYD2** | 9.024531 | HT:0.754;DB:0.246 | GOLGA4;KMT2E;PRKAG1;CCSER1 |
| **WLS** | 8.860415 | DB:0.672;LC:0.328 | OPRM1;WNT1;WNT10B |
| **ITIH1** | 8.814005 | DP:0.519;CC:0.481 | ITIH3;ITIH4 |
| **ITIH2** | 8.707739 | DP:0.586;CC:0.414 | ITIH3;ITIH4 |
| **RBM22** | 8.452246 | DP:0.573;CC:0.221;LC:0.206 | RBM4;RBM4B;RBM14-RBM4;DDX23 |
| **KMT2C** | 8.387658 | CC:0.342;DB:0.333;HT:0.187;LC:0.138 | KMT2E;KMT2D |
| **NDEL1** | 8.382499 | HT:0.586;LC:0.414 | CCDC88A;CCSER1;SYNE1 |
| **CACNA2D1** | 8.209675 | DB:0.401;DP:0.340;CC:0.259 | CACNA2D2;CACNA2D3 |
| **ITIH6** | 8.151256 | DP:1.000 | ITIH3;ITIH4 |
| **SUV39H2** | 8.081378 | DB:0.748;CC:0.252 | CTDSPL;KMT2E;KMT2D |
| **SETD1B** | 7.915443 | CC:0.423;DB:0.408;HT:0.169 | KMT2E;KMT2D |
| **WNT3A** | 7.78555 | DB:0.503;LC:0.497 | DLG2;WNT1;WNT10B |
| **EZH1** | 7.74246 | DB:0.795;LC:0.205 | KMT2E;KMT2D;HDAC2 |
| **CACNG2** | 7.737855 | DB:0.481;CC:0.285;LC:0.234 | DLG2;CACNA2D2;CACNA2D3 |
| **MATR3** | 7.695811 | HT:0.594;CC:0.406 | HNRNPAB;RBM4B;RBM14 |
| **EZH2** | 7.633927 | DB:0.632;HT:0.206;LC:0.163 | KMT2E;SYNE1;KMT2D;HDAC2 |
| **NRXN1** | 7.574001 | CC:0.723;DB:0.277 | MOBP;DLG2;CADPS |
| **TXNL4A** | 7.461791 | HT:0.368;CC:0.328;LC:0.304 | RHEBL1;DDX23 |
| **SRRM2** | 7.440904 | CC:0.792;LC:0.208 | EIF4A1;RBM14;DDX23 |
| **LEF1** | 7.32168 | LC:0.495;DB:0.282;CX:0.222 | CCDC88A;FHIT;WNT1 |
| **VWA5A** | 7.281882 | DP:0.712;GN:0.288 | ITIH3;ITIH4 |
| **SETDB1** | 7.220867 | DB:0.697;LC:0.303 | KMT2E;GIPC2;KMT2D |
| **SCN4A** | 7.135041 | DB:0.676;LC:0.324 | DLG2;CACNA2D2;CACNA2D3 |
| **CACNA2D4** | 7.120546 | DB:0.454;DP:0.368;CC:0.178 | CACNA2D2;CACNA2D3 |
| **PRKAG2** | 7.108537 | CC:0.315;LC:0.256;DP:0.233;DB:0.196 | CNNM4;PRKAG1 |
| **DARS2** | 7.098984 | CE:0.728;GN:0.272 | CYCS;EIF4A1;TFB1M |
| **SMARCC2** | 7.097095 | HT:0.367;CC:0.240;DB:0.217;LC:0.175 | PBRM1;RBM14 |
| **PDSS2** | 7.042709 | DB:0.501;CE:0.499 | CYCS;RCE1;TFB1M |
| **PDSS1** | 6.993792 | DB:0.504;CE:0.496 | CYCS;RCE1;TFB1M |
| **PRDM6** | 6.925275 | DB:0.729;LC:0.271 | KMT2E;KMT2D;HDAC2 |
| **BRD7** | 6.878038 | HT:0.368;LC:0.251;DB:0.214;CE:0.168 | PBRM1;HDAC2 |
| **FASTKD5** | 6.857987 | CE:0.659;LC:0.341 | TM9SF3;CYCS;TFB1M |
| **PRKAB2** | 6.747536 | HT:0.387;LC:0.308;DB:0.167;CC:0.137 | RHEBL1;PRKAG1 |
| **HDAC1** | 6.715928 | DB:0.343;HT:0.332;DP:0.325 | WNT1;HDAC2 |
| **SCN5A** | 6.709799 | DB:0.705;LC:0.295 | DLG2;CACNA2D2;CACNA2D3 |
| **SETD1A** | 6.692522 | DB:0.590;HT:0.207;LC:0.203 | KMT2E;KMT2D |
| **EIF4G1** | 6.684473 | LC:0.394;HT:0.345;DB:0.261 | EIF4A1;RBM4 |
| **CACNA1B** | 6.648218 | DB:0.725;CC:0.275 | CACNA2D2;CACNA2D3 |
| **KMT2B** | 6.613724 | DB:0.704;HT:0.296 | KMT2E;KMT2D |
| **SUZ12** | 6.605496 | LC:0.510;HT:0.257;DB:0.233 | RBM4B;SYNE1;DDX23;HDAC2 |
| **GFM1** | 6.574011 | CE:0.754;GN:0.246 | CYCS;EIF4A1;TFB1M |
| **SS18** | 6.571018 | LC:0.421;HT:0.351;CC:0.227 | PBRM1;RBM14;HDAC2 |
| **SSBP1** | 6.562258 | CE:0.411;CC:0.258;DB:0.203;HT:0.127 | CYCS;ARF3;TFB1M |
| **HNRNPD** | 6.544747 | CC:0.427;DP:0.303;HT:0.270 | HNRNPAB;RBM14 |
| **PAXIP1** | 6.535074 | LC:0.657;HT:0.343 | CCDC88A;DDX23;KMT2D |
| **GRIA1** | 6.500172 | CC:1.000 | MOBP;DLG2 |
| **WNT5B** | 6.49206 | DB:0.781;HT:0.219 | APPBP2;WNT1;WNT10B |
| **AKT1** | 6.472568 | DB:0.548;LC:0.452 | CCDC88A;RHEBL1;EIF4A1;WNT1 |
| **MEF2D** | 6.471973 | DB:0.683;LC:0.317 | KMT2D;TFB1M;HDAC2 |
| **EIF4EBP1** | 6.471797 | DB:0.444;LC:0.341;HT:0.215 | EID1;RHEBL1;EIF4A1 |
| **CACFD1** | 6.372493 | DB:1.000 | CACNA2D2;CACNA2D3 |
| **MTG2** | 6.37154 | CE:1.000 | CYCS;TFB1M |
| **TP63** | 6.357057 | LC:0.799;HT:0.201 | GOLGA4;ITIH4;HNRNPAB;DDX23 |
| **RBBP5** | 6.330147 | LC:0.458;HT:0.313;CC:0.229 | WDR76;KMT2D |
| **PPP2CA** | 6.290779 | DB:0.734;CC:0.266 | EIF4A1;PRKAG1;WNT1 |
| **COA6** | 6.285776 | CE:1.000 | CYCS;TFB1M |
| **SETD3** | 6.279774 | DB:1.000 | KMT2E;KMT2D |
| **PRDM9** | 6.279774 | DB:1.000 | KMT2E;KMT2D |
| **NSD3** | 6.279774 | DB:1.000 | KMT2E;KMT2D |
| **KMT5C** | 6.279774 | DB:1.000 | KMT2E;KMT2D |
| **KMT5B** | 6.279774 | DB:1.000 | KMT2E;KMT2D |
| **NSD1** | 6.279774 | DB:1.000 | KMT2E;KMT2D |
| **SETDB2** | 6.279774 | DB:1.000 | KMT2E;KMT2D |
| **SETD2** | 6.279774 | DB:1.000 | KMT2E;KMT2D |
| **NLGN3** | 6.273192 | CC:0.503;DB:0.267;LC:0.230 | DLG2;CADPS |
| **PRKAG3** | 6.263875 | CC:0.381;DP:0.336;DB:0.283 | CNNM4;PRKAG1 |
| **SUV39H1** | 6.258823 | DB:1.000 | KMT2E;KMT2D |
| **SETMAR** | 6.23659 | DB:0.775;HT:0.225 | APPBP2;KMT2E;KMT2D |
| **SMAD4** | 6.222615 | LC:0.719;DB:0.281 | PBRM1;RHEBL1;WNT1;RHOD |
| **UQCRC2** | 6.202283 | CE:1.000 | CYCS;TFB1M |
| **MEF2C** | 6.192235 | DB:1.000 | WNT1;TFB1M;HDAC2 |
| **TWNK** | 6.165762 | CE:0.692;DB:0.308 | CYCS;TFB1M |
| **NDUFAF1** | 6.165368 | CE:0.740;CC:0.260 | CYCS;TFB1M |
| **ACAT1** | 6.159123 | DB:1.000 | KMT2E;RCE1;KMT2D |
| **HNRNPM** | 6.148766 | HT:0.471;CC:0.276;LC:0.253 | RBM4;RBM4B;RBM14 |
| **RPL12** | 6.130563 | CC:0.687;CX:0.313 | RPL41;TFB1M |
| **PRPF31** | 6.097039 | CC:0.405;HT:0.377;LC:0.218 | RBM4;DDX23 |
| **SRSF7** | 6.096306 | DP:1.000 | RBM4;RBM4B;RBM14-RBM4 |
| **MRPS6** | 6.083677 | CE:1.000 | CYCS;TFB1M |
| **DPY30** | 6.080595 | HT:0.435;DB:0.304;LC:0.261 | KMT2E;KMT2D |
| **RPL23A** | 6.077568 | CC:0.643;CX:0.357 | RPL41;ARF3 |
| **MRPL15** | 6.044484 | CE:1.000 | CYCS;TFB1M |
| **LRPPRC** | 6.017521 | CE:0.586;CC:0.414 | CYCS;TFB1M |
| **ACAT2** | 6.010185 | DB:1.000 | KMT2E;RCE1;KMT2D |
| **CYTH3** | 6.010003 | LC:0.369;HT:0.359;CC:0.271 | IPCEF1;ARF3 |
| **WNT7A** | 5.960046 | DB:1.000 | WNT1;WNT10B |
| **WNT2** | 5.960046 | DB:1.000 | WNT1;WNT10B |
| **WNT2B** | 5.960046 | DB:1.000 | WNT1;WNT10B |
| **WNT9A** | 5.960046 | DB:1.000 | WNT1;WNT10B |
| **WNT7B** | 5.960046 | DB:1.000 | WNT1;WNT10B |
| **WNT9B** | 5.960046 | DB:1.000 | WNT1;WNT10B |
| **VPS26A** | 5.960046 | DB:1.000 | WNT1;WNT10B |
| **WNT6** | 5.960046 | DB:1.000 | WNT1;WNT10B |
| **WNT10A** | 5.960046 | DB:1.000 | WNT1;WNT10B |
| **WNT16** | 5.960046 | DB:1.000 | WNT1;WNT10B |
| **TMED5** | 5.960046 | DB:1.000 | WNT1;WNT10B |
| **VPS29** | 5.960046 | DB:1.000 | WNT1;WNT10B |
| **VPS35** | 5.960046 | DB:1.000 | WNT1;WNT10B |
| **ANKRD28** | 5.957785 | LC:0.593;DB:0.407 | |
| **CPSF6** | 5.954166 | CC:1.000 |  |
| **KMT2A** | 5.945059 | DB:0.540;HT:0.255;LC:0.206 | |
| **WDR5B** | 5.865001 | LC:1.000 |  |
| **MRPS16** | 5.822919 | CE:0.717;CC:0.283 | |
| **ACVR1** | 5.788624 | LC:0.663;DB:0.337 | |
| **FARS2** | 5.78545 | CE:1.000 |  |
| **HOMER1** | 5.778582 | CC:0.401;DB:0.344;HT:0.255 | |
| **COX6B1** | 5.772992 | CE:1.000 |  |
| **SF3B3** | 5.763126 | CC:0.839;CE:0.161 | |
| **SF3B2** | 5.749008 | CC:1.000 |  |
| **RPL10A** | 5.733412 | CC:0.381;CX:0.337;HT:0.282 | |
| **SMARCC1** | 5.691497 | HT:0.352;DB:0.243;CC:0.208;LC:0.196 | |
| **CTNNB1** | 5.677458 | LC:0.665;DB:0.335 | |
| **MRPS17** | 5.673867 | CE:1.000 |  |
| **HNRNPA3** | 5.671247 | CC:0.725;HT:0.275 | |
| **KIAA0391** | 5.644235 | CE:1.000 |  |
| **QRSL1** | 5.600655 | CE:1.000 |  |
| **SF3B1** | 5.566069 | CC:1.000 |  |
| **WNT3** | 5.562792 | DB:1.000 |  |
| **MRPL39** | 5.551814 | CE:1.000 |  |
| **FZD1** | 5.530606 | DB:0.547;LC:0.453 | |
| **ARFIP2** | 5.524426 | LC:0.342;DB:0.335;CC:0.324 | |
| **WNT4** | 5.506158 | DB:1.000 |  |
| **MECOM** | 5.502462 | HT:0.567;DB:0.433 | |
| **MRPL12** | 5.499044 | CE:0.417;CC:0.361;HT:0.222 | |
| **MRPS12** | 5.492221 | CE:1.000 |  |
| **HARS2** | 5.488879 | CE:1.000 |  |
| **BCS1L** | 5.479843 | CE:1.000 |  |
| **RAB6A** | 5.46459 | DP:0.542;LC:0.458 | |
| **ERG** | 5.442495 | LC:1.000 |  |
| **RAB1A** | 5.434217 | DP:0.539;CC:0.461 | |
| **ADRB2** | 5.42216 | LC:1.000 |  |
| **RAB5C** | 5.412894 | DP:0.553;CC:0.447 | |
| **ZCRB1** | 5.407644 | DP:1.000 |  |
| **MRPL55** | 5.398363 | CE:1.000 |  |
| **NCOA6** | 5.38588 | LC:0.500;HT:0.357;CE:0.143 | |
| **LARS2** | 5.381745 | CE:1.000 |  |
| **COA7** | 5.379366 | CE:1.000 |  |
| **UQCRB** | 5.368924 | CE:1.000 |  |
| **SNRPC** | 5.339135 | CC:0.512;LC:0.295;CE:0.194 | |
| **HNRNPUL1** | 5.317385 | CC:0.379;HT:0.327;LC:0.294 | |
| **KCNE3** | 5.314791 | DB:1.000 |  |
| **KCNE4** | 5.314791 | DB:1.000 |  |
| **KCNE1** | 5.314791 | DB:1.000 |  |
| **KCNE5** | 5.314791 | DB:1.000 |  |
| **CACNA1E** | 5.314791 | DB:1.000 |  |
| **CACNA1A** | 5.314791 | DB:1.000 |  |
| **KCNE2** | 5.314791 | DB:1.000 |  |
| **UQCR10** | 5.308672 | CE:1.000 |  |
| **FOXRED1** | 5.29333 | CE:1.000 |  |
| **RAB9A** | 5.293066 | DP:0.562;DB:0.438 | |
| **ARL2** | 5.265786 | DB:0.518;CC:0.482 | |
| **AASS** | 5.260336 | DB:1.000 |  |
| **CAMKMT** | 5.260336 | DB:1.000 |  |
| **PLOD2** | 5.260336 | DB:1.000 |  |
| **TMLHE** | 5.260336 | DB:1.000 |  |
| **BBOX1** | 5.260336 | DB:1.000 |  |
| **PRDM2** | 5.260336 | DB:1.000 |  |
| **KMT5A** | 5.260336 | DB:1.000 |  |
| **PRDM7** | 5.260336 | DB:1.000 |  |
| **PHYKPL** | 5.260336 | DB:1.000 |  |
| **PLOD1** | 5.260336 | DB:1.000 |  |
| **HYKK** | 5.260336 | DB:1.000 |  |
| **MRPS25** | 5.245732 | CE:0.735;CC:0.265 | |
| **PET117** | 5.243864 | CE:1.000 |  |
| **MRPL10** | 5.227877 | CE:1.000 |  |
| **WNT8B** | 5.215906 | DB:1.000 |  |
| **WNT8A** | 5.215906 | DB:1.000 |  |
| **MAPK14** | 5.210073 | LC:0.648;DB:0.352 | |
| **SPNS1** | 5.204604 | LC:1.000 |  |
| **GRID2** | 5.188293 | CC:0.682;LC:0.318 | |
| **GRK3** | 5.185367 | DP:0.512;LC:0.488 | |
| **DAP3** | 5.183866 | CE:0.645;CC:0.355 | |
| **PICK1** | 5.177155 | CC:0.577;LC:0.423 | |
| **EHMT1** | 5.163046 | DB:0.729;LC:0.271 | |
| **BMI1** | 5.162718 | LC:0.613;DB:0.387 | |
| **RGS7** | 5.159176 | CC:0.632;DP:0.368 | |
| **ZCCHC24** | 5.14514 | DP:1.000 |  |
| **ZCCHC3** | 5.14514 | DP:1.000 |  |
| **ZCCHC9** | 5.14514 | DP:1.000 |  |
| **ZCCHC10** | 5.14514 | DP:1.000 |  |
| **ZCCHC7** | 5.14514 | DP:1.000 |  |
| **ZCCHC13** | 5.14514 | DP:1.000 |  |
| **RTL3** | 5.14514 | DP:1.000 |  |
| **NDUFA8** | 5.144707 | CE:1.000 |  |
| **SETD7** | 5.130938 | DB:1.000 |  |
| **ASH1L** | 5.119794 | DB:1.000 |  |
| **HDAC11** | 5.112993 | DP:0.688;LC:0.312 | |
| **NSD2** | 5.105017 | DB:0.713;LC:0.287 | |
| **MRPL28** | 5.0798 | CE:1.000 |  |
| **SARS2** | 5.078131 | CE:0.805;GN:0.195 | |
| **DOT1L** | 5.072334 | DB:1.000 |  |
| **LEP** | 5.050525 | DB:1.000 |  |
| **MRPL43** | 5.037028 | CE:1.000 |  |
| **HNRNPA0** | 5.027728 | CC:1.000 |  |
| **APC** | 5.017027 | LC:0.639;DB:0.361 | |
| **TGFBR1** | 5.000308 | LC:0.640;DB:0.360 | |
| **SCFD1** | 4.988427 | LC:0.529;DB:0.471 | |
| **SF3A2** | 4.984541 | LC:0.403;CC:0.382;CE:0.215 | |
| **CREBBP** | 4.981544 | DB:0.719;LC:0.281 | |
| **COX4I1** | 4.963189 | CE:1.000 |  |
| **MRPL47** | 4.962701 | CE:1.000 |  |
| **MRPS18B** | 4.961178 | CE:0.574;CC:0.426 | |
| **TFB2M** | 4.94992 | DP:0.302;CC:0.294;CE:0.215;DB:0.189 | |
| **EXOC1** | 4.949179 | LC:0.646;HT:0.354 | |
| **NDUFS1** | 4.941287 | CE:0.672;CC:0.328 | |
| **MRPS14** | 4.933139 | CE:1.000 |  |
| **SF3B4** | 4.913278 | CC:0.633;LC:0.367 | |
| **GRIA2** | 4.903068 | CC:1.000 |  |
| **SMAD1** | 4.901696 | LC:0.633;DB:0.367 | |
| **CACNB2** | 4.896701 | DB:0.685;CC:0.315 | |
| **CACNA1D** | 4.868872 | DB:0.717;CC:0.283 | |
| **RAB6B** | 4.859378 | DP:0.587;CC:0.413 | |
| **SCN2B** | 4.856694 | DB:1.000 |  |
| **SCN4B** | 4.856694 | DB:1.000 |  |
| **SCN2A** | 4.856694 | DB:1.000 |  |
| **SCN3A** | 4.856694 | DB:1.000 |  |
| **SCN9A** | 4.856694 | DB:1.000 |  |
| **SCN3B** | 4.856694 | DB:1.000 |  |
| **SCN1A** | 4.856694 | DB:1.000 |  |
| **SCN7A** | 4.856694 | DB:1.000 |  |
| **SCN10A** | 4.856694 | DB:1.000 |  |
| **SCN8A** | 4.856694 | DB:1.000 |  |
| **SCN1B** | 4.856694 | DB:1.000 |  |
| **FGF12** | 4.856694 | DB:1.000 |  |
| **FGF13** | 4.856694 | DB:1.000 |  |
| **RANGRF** | 4.856694 | DB:1.000 |  |
| **FGF11** | 4.856694 | DB:1.000 |  |
| **SCN11A** | 4.856694 | DB:1.000 |  |
| **FGF14** | 4.856694 | DB:1.000 |  |
| **RPL35** | 4.85624 | CC:0.493;CX:0.295;CE:0.211 | |
| **RBMX** | 4.849074 | CC:1.000 |  |
| **MRPS9** | 4.834872 | LC:0.552;CE:0.448 | |
| **BAHD1** | 4.830497 | DP:0.616;LC:0.384 | |
| **MRPL16** | 4.821367 | CE:1.000 |  |
| **ARID2** | 4.819432 | HT:0.313;CE:0.253;LC:0.252;DB:0.182 | |
| **PTCD3** | 4.788687 | CE:1.000 |  |
| **CBX4** | 4.781889 | LC:0.527;DB:0.473 | |
| **SRPK2** | 4.781467 | CC:0.600;LC:0.400 | |
| **WNT11** | 4.780998 | DB:1.000 |  |
| **POLRMT** | 4.776656 | CC:0.310;CE:0.259;LC:0.232;DB:0.200 | |
| **RAB32** | 4.771988 | DP:0.609;CC:0.391 | |
| **CACNG7** | 4.758442 | DB:1.000 |  |
| **CACNA1F** | 4.758442 | DB:1.000 |  |
| **CACNA1S** | 4.758442 | DB:1.000 |  |
| **CACNG1** | 4.758442 | DB:1.000 |  |
| **CACNG6** | 4.758442 | DB:1.000 |  |
| **CACNG5** | 4.758442 | DB:1.000 |  |
| **EID2** | 4.758263 | CC:0.648;LC:0.352 | |
| **SRRM1** | 4.739131 | CC:0.628;LC:0.372 | |
| **GYG1** | 4.738121 | DP:0.652;LC:0.348 | |
| **PLOD3** | 4.736746 | DB:1.000 |  |
| **COLGALT1** | 4.736746 | DB:1.000 |  |
| **COLGALT2** | 4.736746 | DB:1.000 |  |
| **LIN9** | 4.723989 | HT:0.573;LC:0.427 | |
| **TRUB2** | 4.714754 | CE:1.000 |  |
| **CACNB4** | 4.713196 | DB:1.000 |  |
| **CACNB1** | 4.713196 | DB:1.000 |  |
| **SEC11C** | 4.71231 | DB:0.781;HT:0.219 | |
| **NDUFAF5** | 4.707913 | CE:1.000 |  |
| **NDUFV2** | 4.69495 | CE:1.000 |  |
| **SYT1** | 4.692958 | CC:0.598;LC:0.402 | |
| **NARS2** | 4.685147 | CE:1.000 |  |
| **CALM3** | 4.678073 | DB:0.373;CX:0.340;LC:0.288 | |
| **MRPL23** | 4.673502 | CE:1.000 |  |
| **YBEY** | 4.671967 | CE:0.798;HT:0.202 | |
| **MRPS11** | 4.653916 | CE:1.000 |  |
| **SCO2** | 4.653316 | CE:1.000 |  |
| **NDUFA10** | 4.653197 | CE:1.000 |  |
| **RARS2** | 4.650612 | CE:1.000 |  |
| **NDUFS2** | 4.648787 | CE:1.000 |  |
| **PRKAB1** | 4.645914 | HT:0.331;LC:0.248;CC:0.226;DB:0.195 | |
| **AADAT** | 4.639089 | DB:1.000 |  |
| **FZD7** | 4.637186 | LC:0.508;DB:0.492 | |
| **PRPF4** | 4.62494 | HT:0.518;CC:0.482 | |
| **EIF4G2** | 4.624051 | HT:0.305;DB:0.298;LC:0.221;CX:0.176 | |
| **CACNA1C** | 4.609877 | DB:1.000 |  |
| **MRPL21** | 4.609427 | CE:1.000 |  |
| **KCNQ1** | 4.602944 | DB:1.000 |  |
| **ICMT** | 4.602369 | CC:0.515;DB:0.485 | |
| **DLG3** | 4.600949 | CC:0.512;DB:0.488 | |
| **PRPF3** | 4.598311 | HT:0.526;CC:0.474 | |
| **SPCS2** | 4.597583 | DB:1.000 |  |
| **SPCS3** | 4.597583 | DB:1.000 |  |
| **SEC11A** | 4.597583 | DB:1.000 |  |
| **CACNG8** | 4.596218 | DB:1.000 |  |
| **CACNB3** | 4.596218 | DB:1.000 |  |
| **CACNG3** | 4.596218 | DB:1.000 |  |
| **DLST** | 4.586472 | DB:1.000 |  |
| **GFM2** | 4.584795 | CE:1.000 |  |
| **SS18L1** | 4.571242 | HT:0.659;LC:0.341 | |
| **CACNG4** | 4.571228 | DB:1.000 |  |
| **NDUFS5** | 4.570502 | CE:1.000 |  |
| **AR** | 4.559417 | LC:1.000 |  |
| **GIPC1** | 4.556231 | DP:0.558;CC:0.442 | |
| **CD2BP2** | 4.546882 | HT:0.613;CC:0.387 | |
| **HOMER3** | 4.515468 | DB:0.603;CC:0.397 | |
| **SMARCD1** | 4.51257 | HT:0.443;LC:0.291;DB:0.266 | |
| **IBA57** | 4.509428 | CE:0.670;HT:0.330 | |
| **SNRNP200** | 4.505791 | CC:0.507;HT:0.493 | |
| **MRPL2** | 4.500493 | CE:1.000 |  |
| **WDR5** | 4.499069 | HT:0.688;LC:0.312 | |
| **SMARCE1** | 4.486837 | HT:0.478;DB:0.286;LC:0.236 | |
| **EIF4G3** | 4.475496 | HT:0.434;DB:0.318;LC:0.248 | |
| **RBBP4** | 4.471446 | HT:0.633;DB:0.367 | |
| **HDAC3** | 4.461766 | DP:0.645;DB:0.355 | |
| **SART1** | 4.457578 | HT:0.465;CC:0.444;CE:0.090 | |
| **PENK** | 4.457146 | LC:1.000 |  |
| **COQ4** | 4.456337 | CE:1.000 |  |
| **CNNM2** | 4.455227 | DP:0.534;CC:0.466 | |
| **SIN3A** | 4.440359 | HT:0.637;DB:0.363 | |
| **SNRNP40** | 4.439375 | LC:0.535;CC:0.267;HT:0.198 | |
| **MRPS18C** | 4.431884 | CE:1.000 |  |
| **MRPS10** | 4.406034 | CE:1.000 |  |
| **EFTUD2** | 4.392267 | CC:0.522;HT:0.478 | |
| **MTERF1** | 4.381809 | CC:0.602;DB:0.398 | |
| **FZD8** | 4.379935 | LC:1.000 |  |
| **AXIN1** | 4.379824 | DP:0.541;DB:0.459 | |
| **DCAF6** | 4.377371 | LC:0.584;CX:0.416 | |
| **EIF4A2** | 4.375392 | HT:0.577;DB:0.423 | |
| **POLR2M** | 4.372582 | LC:1.000 |  |
| **RPL37** | 4.371608 | CC:0.443;CX:0.340;LC:0.217 | |
| **NDUFB3** | 4.360775 | CE:1.000 |  |
| **RAD21** | 4.344169 | LC:1.000 |  |
| **LMAN2** | 4.33971 | DP:0.465;DB:0.294;CC:0.241 | |
| **LIN37** | 4.336829 | HT:0.546;LC:0.454 | |
| **MARS2** | 4.333429 | CE:1.000 |  |
| **HNRNPA1** | 4.332641 | HT:0.349;CX:0.340;LC:0.311 | |
| **PRKAA1** | 4.327663 | HT:0.354;CC:0.224;DB:0.222;LC:0.199 | |
| **USP32** | 4.324303 | CC:0.580;HT:0.420 | |
| **MTA1** | 4.308878 | HT:1.000 |  |
| **RPL39** | 4.303548 | CC:0.521;CX:0.479 | |
| **MTA2** | 4.296596 | HT:1.000 |  |
| **E2F5** | 4.295364 | LC:1.000 |  |
| **KLF4** | 4.293127 | DB:0.642;LC:0.358 | |
| **TM9SF2** | 4.28601 | DP:0.692;LC:0.308 | |
| **PARD6B** | 4.285136 | LC:0.503;CC:0.497 | |
| **OGDHL** | 4.282561 | DB:1.000 |  |
| **OGDH** | 4.282561 | DB:1.000 |  |
| **EPAS1** | 4.272606 | LC:1.000 |  |
| **NSUN4** | 4.272259 | CC:0.337;CE:0.334;DB:0.329 | |
| **B2M** | 4.264608 | CX:0.609;LC:0.391 | |
| **DHX38** | 4.263735 | CC:0.802;CE:0.198 | |
| **GCDH** | 4.246653 | DB:1.000 |  |
| **PATL1** | 4.242338 | DP:1.000 |  |
| **C11orf80** | 4.242338 | DP:1.000 |  |
| **HDAC8** | 4.242313 | DP:1.000 |  |
| **MBD3** | 4.241317 | HT:1.000 |  |
| **LMAN1L** | 4.241044 | DP:0.613;DB:0.387 | |
| **WDR82** | 4.238794 | LC:1.000 |  |
| **CDK2AP1** | 4.238555 | HT:0.679;LC:0.321 | |
| **PRPF6** | 4.238533 | HT:0.746;CE:0.254 | |
| **DHX8** | 4.234656 | CC:0.810;CE:0.190 | |
| **HCFC1** | 4.230042 | HT:0.535;LC:0.465 | |
| **LMAN1** | 4.225506 | DP:0.612;DB:0.388 | |
| **MBD2** | 4.225323 | HT:0.703;LC:0.297 | |
| **ARL3** | 4.22522 | LC:0.589;CC:0.411 | |
| **RPL37A** | 4.224967 | CC:0.513;CX:0.487 | |
| **FDXR** | 4.22276 | LC:1.000 |  |
| **MTA3** | 4.21814 | HT:1.000 |  |
| **BCL7C** | 4.218104 | HT:1.000 |  |
| **NRXN3** | 4.215861 | DB:0.623;CC:0.377 | |
| **CTBP1** | 4.208589 | DB:1.000 |  |
| **CACHD1** | 4.203146 | DP:1.000 |  |
| **COX6C** | 4.196239 | CE:1.000 |  |
| **EIF3G** | 4.195864 | HT:1.000 |  |
| **ARF5** | 4.187886 | CC:1.000 |  |
| **RPL34** | 4.187719 | CC:0.525;CX:0.475 | |
| **ARF4** | 4.180087 | CC:1.000 |  |
| **IBTK** | 4.169766 | HT:1.000 |  |
| **RPL9** | 4.16818 | CC:0.522;CX:0.478 | |
| **NDUFC1** | 4.158337 | CE:1.000 |  |
| **SMAD2** | 4.152171 | LC:1.000 |  |
| **DHX16** | 4.144361 | CC:1.000 |  |
| **RPL38** | 4.141935 | CC:0.602;CX:0.398 | |
| **SYCP3** | 4.141186 | CC:1.000 |  |
| **PPARGC1A** | 4.131078 | DB:1.000 |  |
| **PRPF4B** | 4.124386 | CC:0.630;LC:0.370 | |
| **DDX46** | 4.121666 | CC:1.000 |  |
| **HINT3** | 4.119369 | DP:1.000 |  |
| **HINT1** | 4.119369 | DP:1.000 |  |
| **HINT2** | 4.119369 | DP:1.000 |  |
| **TM9SF4** | 4.108527 | DP:1.000 |  |
| **LDB1** | 4.099913 | DB:0.508;LC:0.492 | |
| **APTX** | 4.098247 | DP:1.000 |  |
| **PARS2** | 4.095862 | CE:0.614;LC:0.386 | |
| **HNRNPH3** | 4.093619 | CC:0.589;LC:0.411 | |
| **PRKAA2** | 4.090432 | HT:0.441;DB:0.289;LC:0.270 | |
| **RGS6** | 4.085053 | CC:0.571;DP:0.429 | |
| **TMEM171** | 4.082262 | CC:1.000 |  |
| **SMYD1** | 4.074635 | LC:1.000 |  |
| **KDM6A** | 4.071897 | HT:0.398;LC:0.380;CC:0.222 | |
| **CTDSP1** | 4.067496 | CC:0.623;HT:0.377 | |
| **TNIK** | 4.066469 | LC:1.000 |  |
| **DPF3** | 4.066101 | HT:1.000 |  |
| **DLGAP1** | 4.061387 | CC:0.410;DB:0.343;LC:0.247 | |
| **PAFAH1B3** | 4.043035 | CC:1.000 |  |
| **NPBWR2** | 4.028149 | DP:1.000 |  |
| **MRPL3** | 4.024022 | CE:0.676;CC:0.324 | |
| **RPL30** | 4.023979 | CC:0.512;CX:0.488 | |
| **EHMT2** | 4.023784 | DB:1.000 |  |
| **DHX15** | 4.022002 | CC:1.000 |  |
| **EIF4E** | 4.020526 | LC:0.454;HT:0.276;DB:0.270 | |
| **RPS15A** | 4.018564 | CC:0.501;CX:0.499 | |
| **UQCRC1** | 4.018187 | CE:0.643;LC:0.357 | |
| **ARID1A** | 4.013009 | HT:0.406;LC:0.314;DB:0.280 | |
| **CYTH1** | 4.012458 | HT:0.532;LC:0.468 | |
| **NDUFS7** | 4.003795 | CE:1.000 |  |
| **CADPS2** | 4.00378 | DP:1.000 |  |
| **PRPF8** | 4.003559 | HT:1.000 |  |
| **SMARCB1** | 4.000131 | HT:0.419;LC:0.293;DB:0.288 | |
| **CREB1** | 3.998743 | DB:0.537;LC:0.463 | |
| **CDC40** | 3.997467 | CC:1.000 |  |
| **RTN4IP1** | 3.989494 | CE:1.000 |  |
| **MRM3** | 3.98834 | DB:0.505;CE:0.495 | |
| **DHDDS** | 3.987067 | DB:1.000 |  |
| **NUS1** | 3.987067 | DB:1.000 |  |
| **IDI1** | 3.987067 | DB:1.000 |  |
| **PCYOX1** | 3.987067 | DB:1.000 |  |
| **FNTA** | 3.987067 | DB:1.000 |  |
| **FNTB** | 3.987067 | DB:1.000 |  |
| **GGPS1** | 3.987067 | DB:1.000 |  |
| **MVD** | 3.987067 | DB:1.000 |  |
| **ZMPSTE24** | 3.987067 | DB:1.000 |  |
| **IDI2** | 3.987067 | DB:1.000 |  |
| **SMARCD2** | 3.986438 | HT:0.427;DB:0.293;LC:0.280 | |
| **RYK** | 3.98543 | LC:1.000 |  |
| **MTERF4** | 3.978058 | DB:0.513;CE:0.487 | |
| **EPB41** | 3.977614 | DB:1.000 |  |
| **GRIN2A** | 3.975233 | CC:0.633;LC:0.367 | |
| **SRP19** | 3.973693 | DB:1.000 |  |
| **SRP68** | 3.973693 | DB:1.000 |  |
| **SRPRB** | 3.973693 | DB:1.000 |  |
| **OXA1L** | 3.973693 | DB:1.000 |  |
| **SRP14** | 3.973693 | DB:1.000 |  |
| **IMMP1L** | 3.973693 | DB:1.000 |  |
| **SRP54** | 3.973693 | DB:1.000 |  |
| **SRPRA** | 3.973693 | DB:1.000 |  |
| **IMMP2L** | 3.973693 | DB:1.000 |  |
| **SRP9** | 3.973693 | DB:1.000 |  |
| **SRP72** | 3.973693 | DB:1.000 |  |
| **TFAM** | 3.964814 | CE:0.380;LC:0.326;DB:0.294 | |
| **DAAM1** | 3.964364 | LC:0.423;HT:0.301;CX:0.276 | |
| **RPL35A** | 3.963414 | CC:0.417;CX:0.336;CE:0.247 | |
| **YARS2** | 3.963353 | CE:1.000 |  |
| **PDCD4** | 3.961676 | HT:0.637;LC:0.363 | |
| **STRA8** | 3.954704 | CC:1.000 |  |
| **RPS10** | 3.954575 | CX:0.562;CC:0.438 | |
| **RPS11** | 3.954316 | CC:0.519;CX:0.481 | |
| **PIPOX** | 3.944376 | DB:1.000 |  |
| **CENPE** | 3.944318 | LC:0.819;HT:0.181 | |
| **HADH** | 3.938753 | DB:1.000 |  |
| **CYTH2** | 3.937889 | HT:0.548;LC:0.452 | |
| **GAN** | 3.936155 | HT:1.000 |  |
| **ARID1B** | 3.932023 | HT:0.435;DB:0.302;LC:0.263 | |
| **MRM2** | 3.930651 | DB:0.549;CE:0.451 | |
| **PKN2** | 3.920979 | CC:0.581;LC:0.419 | |
| **ASH2L** | 3.919094 | HT:0.551;LC:0.449 | |
| **KIF23** | 3.905654 | LC:1.000 |  |
| **PTBP2** | 3.90477 | LC:1.000 |  |
| **SYNE4** | 3.901219 | DP:1.000 |  |
| **HNRNPU** | 3.896013 | CC:0.701;HT:0.299 | |
| **ACTB** | 3.895914 | CX:0.576;LC:0.424 | |
| **XAB2** | 3.891519 | CC:0.795;HT:0.205 | |
| **BCL7A** | 3.886697 | HT:1.000 |  |
| **CYTH4** | 3.882992 | LC:1.000 |  |
| **MORF4L1** | 3.882224 | HT:1.000 |  |
| **MRPL33** | 3.874878 | CE:1.000 |  |
| **BMPR1B** | 3.871058 | LC:1.000 |  |
| **ECHS1** | 3.869597 | DB:1.000 |  |
| **SMARCD3** | 3.865005 | HT:0.592;LC:0.408 | |
| **CHD4** | 3.862781 | HT:1.000 |  |
| **PDPK1** | 3.861087 | DB:1.000 |  |
| **SMARCA2** | 3.860697 | HT:0.593;DB:0.407 | |
| **HADHA** | 3.85927 | DB:1.000 |  |
| **CTDSP2** | 3.853356 | CC:1.000 |  |
| **CDC42** | 3.845679 | DP:0.586;LC:0.414 | |
| **SPINT2** | 3.843769 | LC:1.000 |  |
| **SHANK1** | 3.841256 | CC:0.537;DB:0.463 | |
| **PTGS2** | 3.840204 | HT:1.000 |  |
| **NOTCH2** | 3.83623 | DB:0.543;LC:0.457 | |
| **RPL27A** | 3.830997 | CC:0.571;CX:0.429 | |
| **MED1** | 3.828072 | DB:1.000 |  |
| **RPS8** | 3.822442 | CC:0.669;CX:0.331 | |
| **MRPS31** | 3.821825 | CE:1.000 |  |
| **CCND1** | 3.821227 | DB:1.000 |  |
| **MTOR** | 3.811124 | DB:0.510;LC:0.490 | |
| **PAGR1** | 3.808516 | HT:0.604;LC:0.396 | |
| **PRKCB** | 3.805466 | DB:1.000 |  |
| **PRKCA** | 3.805236 | DB:1.000 |  |
| **MRPS27** | 3.805099 | CC:0.504;CE:0.496 | |
| **EP300** | 3.804226 | DB:1.000 |  |
| **SNRPD3** | 3.803934 | CC:1.000 |  |
| **PIK3CA** | 3.801645 | DB:1.000 |  |
| **PIK3R1** | 3.801645 | DB:1.000 |  |
| **RPS2** | 3.800027 | CX:1.000 |  |
| **RPS18** | 3.788249 | CX:1.000 |  |
| **EEF1A1** | 3.777863 | CX:1.000 |  |
| **SENP3** | 3.776752 | LC:0.503;CC:0.497 | |
| **GOLGA1** | 3.775922 | CC:0.562;DB:0.438 | |
| **TATDN3** | 3.77578 | GN:1.000 |  |
| **SMARCA4** | 3.774903 | HT:0.666;LC:0.334 | |
| **MTG1** | 3.774155 | CE:1.000 |  |
| **TPT1** | 3.771464 | CX:1.000 |  |
| **E2F4** | 3.768226 | DB:0.539;LC:0.461 | |
| **PPRC1** | 3.768204 | DB:0.607;CX:0.393 | |
| **CASK** | 3.765584 | DB:1.000 |  |
| **SFPQ** | 3.756165 | CC:1.000 |  |
| **NPBWR1** | 3.755466 | DP:1.000 |  |
| **LSM4** | 3.748824 | CC:1.000 |  |
| **ACTL6A** | 3.746176 | HT:0.629;LC:0.371 | |
| **RPL18A** | 3.745391 | CC:0.594;CX:0.406 | |
| **GATAD2A** | 3.734297 | HT:0.664;LC:0.336 | |
| **SF3A3** | 3.732892 | CC:1.000 |  |
| **SYNCRIP** | 3.732678 | CC:1.000 |  |
| **MRPL20** | 3.729127 | CE:1.000 |  |
| **SLU7** | 3.723998 | CC:1.000 |  |
| **CRNKL1** | 3.723998 | CC:1.000 |  |
| **UBB** | 3.722685 | CX:0.536;HT:0.464 | |
| **HNRNPH1** | 3.715396 | CC:0.786;HT:0.214 | |
| **COX5B** | 3.714359 | CE:1.000 |  |
| **RPS27A** | 3.709853 | CC:0.524;CX:0.476 | |
| **GABRA1** | 3.706178 | CC:1.000 |  |
| **WARS2** | 3.700495 | CE:1.000 |  |
| **GTPBP8** | 3.69114 | CE:1.000 |  |
| **MEN1** | 3.690042 | LC:0.535;HT:0.465 | |
| **RPL36A** | 3.68917 | CC:1.000 |  |
| **TARS2** | 3.689165 | CE:1.000 |  |
| **MAGI2** | 3.687808 | CC:1.000 |  |
| **PNMA1** | 3.686623 | LC:1.000 |  |
| **TATDN1** | 3.682875 | GN:1.000 |  |
| **CSNK1A1** | 3.669674 | DB:0.560;PG:0.440 | |
| **MRPL46** | 3.667814 | CE:1.000 |  |
| **TATDN2** | 3.667657 | GN:1.000 |  |
| **USP39** | 3.656698 | CC:0.628;HT:0.372 | |
| **PSPC1** | 3.653804 | CC:1.000 |  |
| **CBX2** | 3.652958 | LC:1.000 |  |
| **AARS2** | 3.646027 | CE:1.000 |  |
| **VARS2** | 3.646027 | CE:1.000 |  |
| **POLG** | 3.646027 | CE:1.000 |  |
| **CALM2** | 3.6402 | DB:0.514;CX:0.486 | |
| **MRPS2** | 3.638373 | CE:1.000 |  |
| **CAND1** | 3.630158 | LC:1.000 |  |
| **RPS23** | 3.629717 | CX:1.000 |  |
| **HNRNPR** | 3.62316 | CC:1.000 |  |
| **MRPS24** | 3.622446 | CE:1.000 |  |
| **PFDN5** | 3.618439 | HT:0.599;CX:0.401 | |
| **MRM1** | 3.617251 | DB:1.000 |  |
| **MAGI1** | 3.616597 | CC:1.000 |  |
| **AQR** | 3.615073 | LC:0.529;CC:0.471 | |
| **RPS27** | 3.61406 | CX:1.000 |  |
| **GTPBP10** | 3.609938 | CE:1.000 |  |
| **NDUFS8** | 3.60947 | CE:1.000 |  |
| **DDX5** | 3.608545 | DB:0.603;LC:0.397 | |
| **PIGR** | 3.592236 | CX:1.000 |  |
| **RPLP1** | 3.586427 | CX:1.000 |  |
| **YWHAH** | 3.583717 | CC:0.544;LC:0.456 | |
| **GATAD2B** | 3.583546 | HT:1.000 |  |
| **DPF2** | 3.581923 | HT:1.000 |  |
| **SYNGAP1** | 3.58024 | CC:1.000 |  |
| **HRAS** | 3.573862 | DB:0.518;LC:0.482 | |
| **DDX28** | 3.569329 | CE:1.000 |  |
| **HNRNPA2B1** | 3.565471 | CC:0.662;CX:0.338 | |
| **RPL6** | 3.561602 | CC:0.605;CX:0.395 | |
| **DDX3X** | 3.561483 | CC:0.591;LC:0.409 | |
| **FZD3** | 3.560439 | LC:1.000 |  |
| **SYF2** | 3.560419 | CC:1.000 |  |
| **ARF1** | 3.552227 | LC:0.504;HT:0.496 | |
| **MTPAP** | 3.544626 | CE:1.000 |  |
| **MRPS21** | 3.544462 | CE:1.000 |  |
| **CWC15** | 3.543373 | CC:1.000 |  |
| **OSTM1** | 3.542208 | LC:1.000 |  |
| **DLG4** | 3.540151 | DB:0.649;LC:0.351 | |
| **DPF1** | 3.538593 | HT:1.000 |  |
| **MBOAT4** | 3.531676 | DB:1.000 |  |
| **PLA2G7** | 3.531676 | DB:1.000 |  |
| **PHF21B** | 3.530383 | HT:1.000 |  |
| **HNRNPL** | 3.52764 | CC:1.000 |  |
| **HUWE1** | 3.516069 | CX:1.000 |  |
| **FRAT1** | 3.512926 | DB:1.000 |  |
| **ZP1** | 3.503765 | CC:1.000 |  |
| **MRPS33** | 3.501642 | CE:1.000 |  |
| **RNPS1** | 3.501591 | CC:0.654;CE:0.346 | |
| **ZDHHC17** | 3.491625 | LC:1.000 |  |
| **MIPEP** | 3.490192 | CE:1.000 |  |
| **CARS2** | 3.484358 | CE:1.000 |  |
| **LSM6** | 3.478531 | CC:1.000 |  |
| **RGS20** | 3.47691 | CC:0.512;DP:0.488 | |
| **NDUFB7** | 3.476333 | CE:1.000 |  |
| **CHD7** | 3.47233 | LC:1.000 |  |
| **CDK1** | 3.472108 | DB:0.532;LC:0.468 | |
| **RPS24** | 3.467151 | CX:1.000 |  |
| **ETFB** | 3.464003 | LC:1.000 |  |
| **RPL8** | 3.45544 | CC:0.621;CX:0.379 | |
| **MORF4L2** | 3.454838 | HT:0.594;CX:0.406 | |
| **ERBIN** | 3.453506 | CC:1.000 |  |
| **MRPS23** | 3.449821 | CE:1.000 |  |
| **ANKRD36B** | 3.445582 | CX:1.000 |  |
| **MAPK3** | 3.44008 | DB:0.530;LC:0.470 | |
| **TXN2** | 3.439132 | DB:0.582;CE:0.418 | |
| **RPL15** | 3.438794 | CC:0.639;CX:0.361 | |
| **NLGN1** | 3.431927 | DB:0.356;CC:0.330;LC:0.313 | |
| **LSM7** | 3.431283 | CC:1.000 |  |
| **HNRNPF** | 3.428465 | CC:0.569;LC:0.431 | |
| **SLC25A26** | 3.42842 | CE:1.000 |  |
| **PAIP1** | 3.427737 | DB:0.580;LC:0.420 | |
| **RPS4X** | 3.424331 | CX:1.000 |  |
| **RPS14** | 3.420135 | CX:1.000 |  |
| **LSM2** | 3.416439 | CC:0.810;CE:0.190 | |
| **LARGE1** | 3.416429 | DP:1.000 |  |
| **GYG2** | 3.416429 | DP:1.000 |  |
| **LARGE2** | 3.416429 | DP:1.000 |  |
| **GXYLT1** | 3.416429 | DP:1.000 |  |
| **GXYLT2** | 3.416429 | DP:1.000 |  |
| **GLT8D2** | 3.416429 | DP:1.000 |  |
| **C17orf49** | 3.41601 | HT:1.000 |  |
| **MRPL17** | 3.415117 | CE:1.000 |  |
| **NLGN2** | 3.412656 | DB:0.364;CC:0.321;LC:0.315 | |
| **PGGT1B** | 3.409402 | CC:1.000 |  |
| **OLIG1** | 3.408512 | CC:0.545;CX:0.455 | |
| **MTERF3** | 3.408342 | CE:1.000 |  |
| **PTX3** | 3.40606 | LC:1.000 |  |
| **HMGCS1** | 3.403969 | DB:1.000 |  |
| **SYN2** | 3.39819 | CC:1.000 |  |
| **CRIPT** | 3.394839 | CC:0.551;LC:0.449 | |
| **PHF10** | 3.394202 | HT:1.000 |  |
| **PRPF40A** | 3.393622 | CC:1.000 |  |
| **ABLIM2** | 3.388986 | CC:1.000 |  |
| **SMAD5** | 3.386531 | DB:0.540;LC:0.460 | |
| **GRIN2B** | 3.385239 | CC:0.610;LC:0.390 | |
| **RPL32** | 3.384016 | CX:1.000 |  |
| **RPL36** | 3.383179 | CC:1.000 |  |
| **MALSU1** | 3.380403 | CE:1.000 |  |
| **BCAS2** | 3.373823 | CC:0.760;HT:0.240 | |
| **DDX39B** | 3.373238 | CC:1.000 |  |
| **SNW1** | 3.3669 | CC:0.616;LC:0.384 | |
| **NDUFA11** | 3.347594 | CE:1.000 |  |
| **DNAJC1** | 3.345757 | LC:1.000 |  |
| **POLG2** | 3.337793 | CC:0.397;DB:0.359;CE:0.244 | |
| **LIN7A** | 3.335657 | CC:0.572;LC:0.428 | |
| **FZD9** | 3.334649 | LC:1.000 |  |
| **COX7C** | 3.331212 | CE:0.566;CX:0.434 | |
| **MAGI3** | 3.325383 | CC:1.000 |  |
| **WIF1** | 3.323291 | LC:0.574;DB:0.426 | |
| **NOTCH1** | 3.31808 | DB:0.567;LC:0.433 | |
| **ARID4A** | 3.317727 | LC:1.000 |  |
| **MYBL2** | 3.312134 | LC:0.549;HT:0.451 | |
| **PET100** | 3.311829 | CE:1.000 |  |
| **GDF9** | 3.311722 | CC:1.000 |  |
| **CBFA2T3** | 3.308712 | LC:1.000 |  |
| **CWC25** | 3.307561 | CC:1.000 |  |
| **MRPL48** | 3.304414 | CE:1.000 |  |
| **DLGAP2** | 3.298582 | DB:0.505;CC:0.495 | |
| **MTFMT** | 3.296313 | CE:1.000 |  |
| **PRPF38A** | 3.285715 | CC:1.000 |  |
| **MAPK12** | 3.282966 | LC:1.000 |  |
| **NDUFB9** | 3.281424 | CE:1.000 |  |
| **MRPL57** | 3.279829 | CE:1.000 |  |
| **DROSHA** | 3.27462 | LC:1.000 |  |
| **UCN** | 3.269331 | DB:1.000 |  |
| **GAPDH** | 3.264123 | CX:0.551;CE:0.449 | |
| **GATAD1** | 3.256265 | HT:0.619;LC:0.381 | |
| **MRPL32** | 3.254386 | CE:1.000 |  |
| **MRPL30** | 3.254386 | CE:1.000 |  |
| **LSM8** | 3.249667 | CC:1.000 |  |
| **DIABLO** | 3.248041 | DB:1.000 |  |
| **FNIP1** | 3.2386 | LC:1.000 |  |
| **PCSK1** | 3.237547 | DB:1.000 |  |
| **CYC1** | 3.235868 | CE:0.535;LC:0.465 | |
| **NDE1** | 3.233464 | HT:1.000 |  |
| **RGS19** | 3.230265 | DP:0.569;CC:0.431 | |
| **GPKOW** | 3.224254 | CC:1.000 |  |
| **GH1** | 3.221995 | DB:1.000 |  |
| **TNRC18** | 3.216978 | DP:1.000 |  |
| **ZMAT2** | 3.2165 | CC:0.759;CE:0.241 | |
| **NDUFB10** | 3.212481 | CE:1.000 |  |
| **THRAP3** | 3.212209 | CC:0.557;HT:0.443 | |
| **RPUSD4** | 3.210033 | CE:1.000 |  |
| **GGA3** | 3.187585 | CC:0.550;LC:0.450 | |
| **PCBP2** | 3.187051 | CC:1.000 |  |
| **EIF3A** | 3.185162 | DB:0.606;HT:0.394 | |
| **NEK1** | 3.18382 | CC:1.000 |  |
| **GATC** | 3.182226 | CE:1.000 |  |
| **RBBP7** | 3.177333 | HT:0.548;DB:0.452 | |
| **POC1B** | 3.17501 | LC:1.000 |  |
| **LSM3** | 3.171671 | CC:1.000 |  |
| **EID3** | 3.167498 | CC:0.601;HT:0.399 | |
| **PLRG1** | 3.16459 | CC:1.000 |  |
| **PRKAR2B** | 3.153626 | DB:0.556;CX:0.444 | |
| **RPS15** | 3.152843 | CX:1.000 |  |
| **PTPRD** | 3.149765 | CC:1.000 |  |
| **MRPL34** | 3.14373 | CE:1.000 |  |
| **HSPE1** | 3.141966 | LC:1.000 |  |
| **GGA1** | 3.141047 | CC:0.552;LC:0.448 | |
| **PMVK** | 3.140533 | DB:1.000 |  |
| **MVK** | 3.140533 | DB:1.000 |  |
| **CAMKK2** | 3.139993 | LC:1.000 |  |
| **GIPC3** | 3.139759 | CC:1.000 |  |
| **RAB3D** | 3.134743 | DP:1.000 |  |
| **RAB39A** | 3.134743 | DP:1.000 |  |
| **RAB3B** | 3.134743 | DP:1.000 |  |
| **RAB17** | 3.134743 | DP:1.000 |  |
| **RAB31** | 3.134743 | DP:1.000 |  |
| **RAB2B** | 3.134743 | DP:1.000 |  |
| **RAB15** | 3.134743 | DP:1.000 |  |
| **RAB39B** | 3.134743 | DP:1.000 |  |
| **RAB29** | 3.134743 | DP:1.000 |  |
| **RAB28** | 3.134743 | DP:1.000 |  |
| **RAB34** | 3.134743 | DP:1.000 |  |
| **MRPL41** | 3.133849 | CE:1.000 |  |
| **SSBP3** | 3.133573 | LC:1.000 |  |
| **MRPL40** | 3.123865 | CE:1.000 |  |
| **UGGT2** | 3.123314 | DP:1.000 |  |
| **NEK9** | 3.115398 | CC:1.000 |  |
| **FIGLA** | 3.115347 | CC:1.000 |  |
| **DISC1** | 3.109278 | LC:1.000 |  |
| **LRP5** | 3.109186 | LC:0.532;DB:0.468 | |
| **NDUFA6** | 3.108173 | CE:1.000 |  |
| **MCAT** | 3.103584 | CE:1.000 |  |
| **COX4I2** | 3.102486 | LC:1.000 |  |
| **RPLP0** | 3.09908 | CX:1.000 |  |
| **TEKT1** | 3.09883 | CC:1.000 |  |
| **KIAA1549** | 3.096107 | LC:1.000 |  |
| **NGRN** | 3.093287 | CE:1.000 |  |
| **ACHE** | 3.092451 | DB:1.000 |  |
| **BCHE** | 3.092451 | DB:1.000 |  |
| **KRT1** | 3.088154 | CX:1.000 |  |
| **ESR1** | 3.087268 | DB:0.565;CX:0.435 | |
| **RFX5** | 3.086488 | LC:1.000 |  |
| **RAB21** | 3.085255 | DP:1.000 |  |
| **RAB9B** | 3.085255 | DP:1.000 |  |
| **RAB2A** | 3.085255 | DP:1.000 |  |
| **RAB5A** | 3.085255 | DP:1.000 |  |
| **RAB41** | 3.085255 | DP:1.000 |  |
| **RAB5B** | 3.085255 | DP:1.000 |  |
| **RAB23** | 3.085255 | DP:1.000 |  |
| **RAB22A** | 3.085255 | DP:1.000 |  |
| **RAB4B** | 3.085255 | DP:1.000 |  |
| **RABL2A** | 3.085255 | DP:1.000 |  |
| **RAB19** | 3.085255 | DP:1.000 |  |
| **GATA4** | 3.078585 | DB:0.564;LC:0.436 | |
| **RPS19** | 3.075612 | CX:1.000 |  |
| **PPIH** | 3.074907 | CC:1.000 |  |
| **IFT57** | 3.07396 | HT:1.000 |  |
| **GRIA4** | 3.071142 | CC:1.000 |  |
| **NDUFB5** | 3.063633 | CE:1.000 |  |
| **EIF5** | 3.060378 | DB:1.000 |  |
| **DTL** | 3.059612 | LC:1.000 |  |
| **RAB37** | 3.058459 | DP:1.000 |  |
| **RAB36** | 3.058459 | DP:1.000 |  |
| **ANKRD27** | 3.053886 | LC:1.000 |  |
| **SFRP1** | 3.053886 | LC:1.000 |  |
| **PORCN** | 3.053886 | LC:1.000 |  |
| **ZP2** | 3.052524 | CC:1.000 |  |
| **GRIK2** | 3.051305 | CC:1.000 |  |
| **MRPL22** | 3.05105 | CE:1.000 |  |
| **CDCA3** | 3.048429 | HT:1.000 |  |
| **CNNM1** | 3.045938 | CC:1.000 |  |
| **MAFK** | 3.041573 | HT:0.561;LC:0.439 | |
| **ALDH2** | 3.037837 | LC:1.000 |  |
| **SPHAR** | 3.034202 | DP:1.000 |  |
| **RAB10** | 3.034202 | DP:1.000 |  |
| **RAB26** | 3.034202 | DP:1.000 |  |
| **RAB35** | 3.034202 | DP:1.000 |  |
| **RAB18** | 3.034202 | DP:1.000 |  |
| **RAB4A** | 3.034202 | DP:1.000 |  |
| **RAB33B** | 3.030993 | DP:1.000 |  |
| **RAB33A** | 3.030993 | DP:1.000 |  |
| **RAB12** | 3.030993 | DP:1.000 |  |
| **ATPAF2** | 3.022956 | CC:0.704;CE:0.296 | |
| **GJA4** | 3.020686 | LC:1.000 |  |
| **MXD1** | 3.020623 | LC:1.000 |  |
| **NANOS3** | 3.00899 | CC:1.000 |  |
| **SF3B5** | 2.99657 | CC:1.000 |  |
| **MRPS5** | 2.995873 | CE:1.000 |  |
| **ECSIT** | 2.995873 | CE:1.000 |  |
| **RAB13** | 2.995841 | DP:1.000 |  |
| **RAB8B** | 2.995841 | DP:1.000 |  |
| **RAB1B** | 2.995841 | DP:1.000 |  |
| **NANOS1** | 2.995255 | CC:1.000 |  |
| **TUFM** | 2.992721 | CC:0.564;CE:0.436 | |
| **CUL3** | 2.989521 | LC:1.000 |  |
| **STAU1** | 2.97929 | LC:1.000 |  |
| **RASSF8** | 2.971712 | LC:1.000 |  |
| **RPSA** | 2.971298 | CC:0.582;CX:0.418 | |
| **PIP5K1A** | 2.961017 | LC:1.000 |  |
| **RGS10** | 2.95774 | DP:1.000 |  |
| **AKAP10** | 2.95774 | DP:1.000 |  |
| **RGS22** | 2.95774 | DP:1.000 |  |
| **NUCKS1** | 2.954259 | CC:1.000 |  |
| **SMNDC1** | 2.953009 | CC:1.000 |  |
| **SF3A1** | 2.952848 | CC:1.000 |  |
| **ARL1** | 2.952564 | LC:0.504;DB:0.496 | |
| **APBA2** | 2.95137 | CC:1.000 |  |
| **METTL17** | 2.94982 | CE:1.000 |  |
| **LRRTM4** | 2.949088 | DB:1.000 |  |
| **NRXN2** | 2.949088 | DB:1.000 |  |
| **EPB41L2** | 2.949088 | DB:1.000 |  |
| **HOMER2** | 2.949088 | DB:1.000 |  |
| **LRRTM1** | 2.949088 | DB:1.000 |  |
| **NLGN4Y** | 2.949088 | DB:1.000 |  |
| **NLGN4X** | 2.949088 | DB:1.000 |  |
| **SHANK2** | 2.949088 | DB:1.000 |  |
| **DLGAP4** | 2.949088 | DB:1.000 |  |
| **LRRTM2** | 2.949088 | DB:1.000 |  |
| **EPB41L5** | 2.949088 | DB:1.000 |  |
| **DLGAP3** | 2.949088 | DB:1.000 |  |
| **LRRTM3** | 2.949088 | DB:1.000 |  |
| **EPB41L3** | 2.949088 | DB:1.000 |  |
| **MRPS22** | 2.948052 | CE:0.552;CC:0.448 | |
| **CSTF2** | 2.938829 | CC:1.000 |  |
| **N4BP2** | 2.938233 | LC:1.000 |  |
| **PROSER1** | 2.938233 | LC:1.000 |  |
| **PABPC4** | 2.936856 | CC:1.000 |  |
| **FUBP1** | 2.93577 | CC:1.000 |  |
| **COPZ2** | 2.935307 | CC:1.000 |  |
| **NDOR1** | 2.933986 | LC:1.000 |  |
| **RPL19** | 2.929352 | CX:1.000 |  |
| **ARFGEF2** | 2.918122 | CC:1.000 |  |
| **PATJ** | 2.915781 | CC:1.000 |  |
| **NEK10** | 2.914898 | CC:0.627;PG:0.373 | |
| **NDUFA2** | 2.901726 | CE:1.000 |  |
| **RGS12** | 2.896708 | DP:0.514;CC:0.486 | |
| **MRPL14** | 2.889865 | CE:1.000 |  |
| **EIF1AX** | 2.882168 | DB:1.000 |  |
| **EIF1** | 2.882168 | DB:1.000 |  |
| **APAF1** | 2.882112 | LC:1.000 |  |
| **MARK3** | 2.88138 | LC:0.382;PG:0.368;GN:0.250 | |
| **RPAP3** | 2.86165 | LC:1.000 |  |
| **RPS12** | 2.859175 | CX:0.581;CC:0.419 | |
| **DRD2** | 2.858009 | LC:1.000 |  |
| **RPS6** | 2.856857 | CX:1.000 |  |
| **UNC93B1** | 2.854355 | LC:1.000 |  |
| **PLPPR4** | 2.854355 | LC:1.000 |  |
| **COP1** | 2.852929 | LC:1.000 |  |
| **C1QBP** | 2.852625 | CE:1.000 |  |
| **RBFA** | 2.852625 | CE:1.000 |  |
| **TRMT10C** | 2.852625 | CE:1.000 |  |
| **RPS28** | 2.850997 | CX:1.000 |  |
| **ACTL6B** | 2.850347 | LC:0.522;DB:0.478 | |
| **CUL4A** | 2.849512 | LC:1.000 |  |
| **GOLGA3** | 2.841872 | CC:1.000 |  |
| **GCC2** | 2.841612 | DB:0.553;CC:0.447 | |
| **RAC2** | 2.840573 | DP:1.000 |  |
| **RAB24** | 2.840573 | DP:1.000 |  |
| **RAB6C** | 2.840573 | DP:1.000 |  |
| **RHOF** | 2.840573 | DP:1.000 |  |
| **RAN** | 2.840573 | DP:1.000 |  |
| **RHOG** | 2.840573 | DP:1.000 |  |
| **LDHB** | 2.838962 | LC:0.504;CX:0.496 | |
| **CUL4B** | 2.830117 | LC:1.000 |  |
| **BPTF** | 2.830078 | HT:1.000 |  |
| **MBP** | 2.827456 | HT:0.507;CX:0.493 | |
| **MAT1A** | 2.825018 | CX:1.000 |  |
| **RBM39** | 2.822378 | CC:0.743;HT:0.257 | |
| **MBD3L1** | 2.822153 | LC:0.637;HT:0.363 | |
| **BCAS4** | 2.820288 | CC:1.000 |  |
| **KCNN4** | 2.81941 | LC:1.000 |  |
| **DIAPH2** | 2.81941 | LC:1.000 |  |
| **SNAI1** | 2.81941 | LC:1.000 |  |
| **RPL31** | 2.814477 | CX:1.000 |  |
| **CYB5R1** | 2.812746 | LC:1.000 |  |
| **TRIP11** | 2.811285 | CC:1.000 |  |
| **SYCP1** | 2.810614 | CC:1.000 |  |
| **HNRNPC** | 2.810148 | CC:1.000 |  |
| **DLG1** | 2.807558 | DB:0.590;LC:0.410 | |
| **ECM2** | 2.807489 | CC:1.000 |  |
| **KCNF1** | 2.806182 | LC:1.000 |  |
| **EIF2S3** | 2.803697 | DB:1.000 |  |
| **NDN** | 2.801747 | LC:0.583;HT:0.417 | |
| **ROR2** | 2.799717 | LC:1.000 |  |
| **USP2** | 2.799717 | LC:1.000 |  |
| **GRID1** | 2.790195 | LC:1.000 |  |
| **SCRIB** | 2.788741 | CC:1.000 |  |
| **ULK1** | 2.787254 | LC:1.000 |  |
| **A1BG** | 2.784609 | CX:1.000 |  |
| **EIF2S2** | 2.782758 | DB:1.000 |  |
| **CDK14** | 2.781793 | LC:1.000 |  |
| **APOC1** | 2.765985 | LC:0.527;CX:0.473 | |
| **ARHGAP29** | 2.762825 | LC:1.000 |  |
| **ARL2BP** | 2.760567 | DB:1.000 |  |
| **SLC25A5** | 2.760567 | DB:1.000 |  |
| **UPF2** | 2.760402 | LC:1.000 |  |
| **SMURF2** | 2.754523 | LC:1.000 |  |
| **NEK6** | 2.752471 | CC:1.000 |  |
| **KDM3A** | 2.746366 | LC:1.000 |  |
| **ZKSCAN7** | 2.746366 | LC:1.000 |  |
| **CCDC158** | 2.746366 | LC:1.000 |  |
| **DIS3L2** | 2.746366 | LC:1.000 |  |
| **RASGRP1** | 2.743837 | CC:0.522;CX:0.478 | |
| **ACACA** | 2.733558 | DB:0.536;LC:0.464 | |
| **KDM1A** | 2.73306 | HT:1.000 |  |
| **IGSF21** | 2.731563 | LC:1.000 |  |
| **CXXC1** | 2.730957 | HT:0.526;LC:0.474 | |
| **HMGCS2** | 2.728479 | DB:1.000 |  |
| **DYNLL1** | 2.728069 | HT:0.508;LC:0.492 | |
| **TAF15** | 2.726809 | CC:0.733;HT:0.267 | |
| **MRPL37** | 2.721761 | CE:1.000 |  |
| **EPB41L1** | 2.719622 | DB:1.000 |  |
| **NUPR1** | 2.718067 | LC:1.000 |  |
| **CASP9** | 2.716493 | LC:1.000 |  |
| **CCDC12** | 2.716077 | CC:1.000 |  |
| **RGS9** | 2.712 | DP:1.000 |  |
| **RGS11** | 2.712 | DP:1.000 |  |
| **COX16** | 2.708118 | CE:1.000 |  |
| **BRMS1L** | 2.705976 | HT:0.584;LC:0.416 | |
| **NDUFA9** | 2.705444 | CE:1.000 |  |
| **ADAM22** | 2.700325 | CC:1.000 |  |
| **SFRP2** | 2.697315 | LC:1.000 |  |
| **MRPS26** | 2.694377 | CE:1.000 |  |
| **MRPL11** | 2.694377 | CE:1.000 |  |
| **RGS16** | 2.692386 | CC:1.000 |  |
| **LRP6** | 2.678556 | DB:0.503;LC:0.497 | |
| **GOLT1B** | 2.678084 | LC:1.000 |  |
| **PIGN** | 2.678084 | LC:1.000 |  |
| **LPGAT1** | 2.678084 | LC:1.000 |  |
| **TMCO1** | 2.678084 | LC:1.000 |  |
| **YIPF3** | 2.678084 | LC:1.000 |  |
| **GRK4** | 2.677325 | DP:1.000 |  |
| **GRK7** | 2.677325 | DP:1.000 |  |
| **GRK5** | 2.677325 | DP:1.000 |  |
| **GRK6** | 2.677325 | DP:1.000 |  |
| **PUM1** | 2.676647 | CC:1.000 |  |
| **CFHR1** | 2.674828 | CC:1.000 |  |
| **GRK2** | 2.674137 | DP:1.000 |  |
| **NDUFB8** | 2.67173 | CE:1.000 |  |
| **WASHC3** | 2.669814 | HT:1.000 |  |
| **RHOU** | 2.669604 | DP:1.000 |  |
| **DMD** | 2.667948 | LC:1.000 |  |
| **STX6** | 2.664511 | DB:0.538;CC:0.462 | |
| **DOK5** | 2.663243 | LC:1.000 |  |
| **MYOM1** | 2.663243 | LC:1.000 |  |
| **MAGEF1** | 2.658588 | LC:1.000 |  |
| **PANK3** | 2.657329 | CC:1.000 |  |
| **TIMMDC1** | 2.652725 | CE:1.000 |  |
| **RPS29** | 2.650207 | CX:1.000 |  |
| **CLU** | 2.649507 | LC:0.522;CX:0.478 | |
| **RPL7** | 2.64414 | CX:1.000 |  |
| **NEK11** | 2.64254 | CC:1.000 |  |
| **YAF2** | 2.641579 | LC:1.000 |  |
| **NUBPL** | 2.632992 | CE:0.665;GN:0.335 | |
| **H2AFY2** | 2.624406 | LC:1.000 |  |
| **SIN3B** | 2.620815 | HT:0.514;DB:0.486 | |
| **CNBD1** | 2.620092 | DP:1.000 |  |
| **CNGB3** | 2.620092 | DP:1.000 |  |
| **INHBE** | 2.614535 | LC:0.528;CX:0.472 | |
| **RALBP1** | 2.613002 | HT:1.000 |  |
| **PCID2** | 2.608402 | LC:1.000 |  |
| **SUN1** | 2.606347 | LC:1.000 |  |
| **FUBP3** | 2.604997 | CC:1.000 |  |
| **COPS2** | 2.604117 | HT:1.000 |  |
| **HCFC2** | 2.602728 | HT:1.000 |  |
| **ATP5F1B** | 2.602505 | DB:0.663;CE:0.337 | |
| **RPL27** | 2.599877 | CX:1.000 |  |
| **MRPL38** | 2.595494 | CE:1.000 |  |
| **TIA1** | 2.59086 | CC:1.000 |  |
| **RGS14** | 2.590525 | DP:1.000 |  |
| **MYCN** | 2.589574 | LC:0.720;CE:0.280 | |
| **CABIN1** | 2.588944 | LC:0.523;DB:0.477 | |
| **PHF8** | 2.583017 | LC:1.000 |  |
| **SLC25A4** | 2.580273 | DB:1.000 |  |
| **PDZD2** | 2.579096 | CC:1.000 |  |
| **FRAT2** | 2.577314 | DB:1.000 |  |
| **GUK1** | 2.569068 | DB:1.000 |  |
| **SPTBN1** | 2.569068 | DB:1.000 |  |
| **ADD1** | 2.569068 | DB:1.000 |  |
| **ANK1** | 2.569068 | DB:1.000 |  |
| **PCLO** | 2.569068 | DB:1.000 |  |
| **NFASC** | 2.569068 | DB:1.000 |  |
| **NCAM1** | 2.569068 | DB:1.000 |  |
| **HIST1H3C** | 2.568873 | LC:1.000 |  |
| **FUS** | 2.56669 | LC:0.506;HT:0.494 | |
| **RABEPK** | 2.5646 | DB:1.000 |  |
| **VPS53** | 2.5646 | DB:1.000 |  |
| **SCOC** | 2.5646 | DB:1.000 |  |
| **USP6NL** | 2.5646 | DB:1.000 |  |
| **VPS54** | 2.5646 | DB:1.000 |  |
| **RHOBTB3** | 2.5646 | DB:1.000 |  |
| **VPS51** | 2.5646 | DB:1.000 |  |
| **TMF1** | 2.5646 | DB:1.000 |  |
| **SYS1** | 2.5646 | DB:1.000 |  |
| **ARFRP1** | 2.5646 | DB:1.000 |  |
| **GCC1** | 2.5646 | DB:1.000 |  |
| **STX10** | 2.5646 | DB:1.000 |  |
| **VPS52** | 2.5646 | DB:1.000 |  |
| **SLC25A6** | 2.56343 | DB:1.000 |  |
| **RPL17** | 2.563347 | CX:1.000 |  |
| **UTY** | 2.558883 | HT:1.000 |  |
| **RPL13A** | 2.558697 | CX:1.000 |  |
| **SIRT3** | 2.5556 | DB:1.000 |  |
| **GABPB1** | 2.5556 | DB:1.000 |  |
| **GABPA** | 2.5556 | DB:1.000 |  |
| **UTP4** | 2.547456 | CX:0.530;HT:0.470 | |
| **MIA2** | 2.54579 | DB:1.000 |  |
| **MIA3** | 2.54579 | DB:1.000 |  |
| **TUBA8** | 2.541209 | HT:1.000 |  |
| **MOG** | 2.538143 | CX:1.000 |  |
| **HSPD1** | 2.534318 | LC:1.000 |  |
| **ABCC1** | 2.530412 | LC:1.000 |  |
| **MBOAT7** | 2.530412 | LC:1.000 |  |
| **SAP30** | 2.525205 | HT:1.000 |  |
| **CRTC3** | 2.524763 | DB:1.000 |  |
| **CRTC1** | 2.524763 | DB:1.000 |  |
| **CRTC2** | 2.524763 | DB:1.000 |  |
| **PLXNA1** | 2.524548 | LC:1.000 |  |
| **RPL3** | 2.52006 | CX:1.000 |  |
| **TBC1D19** | 2.51794 | LC:1.000 |  |
| **CKAP2** | 2.51794 | LC:1.000 |  |
| **UNC79** | 2.51794 | LC:1.000 |  |
| **GTPBP3** | 2.51794 | LC:1.000 |  |
| **GDF10** | 2.51794 | LC:1.000 |  |
| **CALML5** | 2.517837 | LC:1.000 |  |
| **MTREX** | 2.509991 | CC:1.000 |  |
| **UQCRFS1** | 2.509109 | CE:0.706;HT:0.294 | |
| **MPDZ** | 2.509002 | CC:1.000 |  |
| **DNAJA1** | 2.507708 | CC:1.000 |  |
| **YIPF5** | 2.504747 | LC:1.000 |  |
| **PUM2** | 2.503186 | CC:1.000 |  |
| **RCOR1** | 2.502906 | HT:1.000 |  |
| **PLG** | 2.502817 | LC:1.000 |  |
| **RPL7A** | 2.501711 | CX:1.000 |  |
| **PLIN3** | 2.499455 | DB:1.000 |  |
| **GRIN1** | 2.499429 | DB:0.552;LC:0.448 | |
| **MRPL13** | 2.498709 | CC:0.520;CE:0.480 | |
| **KRT14** | 2.496766 | CX:1.000 |  |
| **U2SURP** | 2.494797 | CC:1.000 |  |
| **C1orf198** | 2.488049 | HT:1.000 |  |
| **CNTNAP1** | 2.486152 | HT:1.000 |  |
| **MAGEA1** | 2.485786 | LC:1.000 |  |
| **RPL28** | 2.485124 | CX:1.000 |  |
| **BUD31** | 2.482871 | CC:0.740;CE:0.260 | |
| **BCAS3** | 2.481789 | CC:1.000 |  |
| **ACAD9** | 2.477031 | CE:1.000 |  |
| **SEC63** | 2.4765 | DB:1.000 |  |
| **SEC62** | 2.4765 | DB:1.000 |  |
| **METTL21C** | 2.47212 | LC:1.000 |  |
| **CXorf56** | 2.47212 | LC:1.000 |  |
| **MUSK** | 2.470629 | LC:1.000 |  |
| **RHOH** | 2.469555 | DP:1.000 |  |
| **RERG** | 2.469555 | DP:1.000 |  |
| **DIRAS1** | 2.469555 | DP:1.000 |  |
| **RAC1** | 2.469555 | DP:1.000 |  |
| **RAC3** | 2.469555 | DP:1.000 |  |
| **ERAS** | 2.469555 | DP:1.000 |  |
| **RHOQ** | 2.469555 | DP:1.000 |  |
| **RAB20** | 2.469555 | DP:1.000 |  |
| **RND2** | 2.469555 | DP:1.000 |  |
| **RASL12** | 2.469555 | DP:1.000 |  |
| **DIRAS3** | 2.469555 | DP:1.000 |  |
| **CYB5R3** | 2.467662 | LC:1.000 |  |
| **NCBP2** | 2.466504 | CC:1.000 |  |
| **RPL24** | 2.464172 | CX:1.000 |  |
| **YBX1** | 2.46238 | LC:0.405;CX:0.400;CE:0.195 | |
| **AXIN2** | 2.458653 | DP:1.000 |  |
| **ATP5PB** | 2.457521 | CE:0.591;CX:0.409 | |
| **PYGO2** | 2.454562 | LC:1.000 |  |
| **MAGEH1** | 2.451706 | LC:1.000 |  |
| **PHC1** | 2.451593 | DB:0.573;LC:0.427 | |
| **ANKRD26** | 2.451068 | LC:1.000 |  |
| **ARFIP1** | 2.450285 | LC:1.000 |  |
| **HIST1H4I** | 2.448025 | LC:1.000 |  |
| **ATP5ME** | 2.446571 | CE:1.000 |  |
| **MRPS35** | 2.446571 | CE:1.000 |  |
| **PI4KA** | 2.446392 | DB:1.000 |  |
| **PI4KB** | 2.446392 | DB:1.000 |  |
| **TPTE2** | 2.446392 | DB:1.000 |  |
| **TPTE** | 2.446392 | DB:1.000 |  |
| **SACM1L** | 2.446392 | DB:1.000 |  |
| **METTL21A** | 2.444648 | LC:1.000 |  |
| **FOXA1** | 2.444016 | LC:1.000 |  |
| **EED** | 2.443793 | DB:0.581;HT:0.419 | |
| **LPAR1** | 2.442746 | DB:1.000 |  |
| **AKAP13** | 2.442746 | DB:1.000 |  |
| **LEPR** | 2.442746 | DB:1.000 |  |
| **LPAR3** | 2.442746 | DB:1.000 |  |
| **AKAP8** | 2.442746 | DB:1.000 |  |
| **NCAPD2** | 2.442746 | DB:1.000 |  |
| **LPAR2** | 2.442746 | DB:1.000 |  |
| **RRM2B** | 2.442507 | CC:1.000 |  |
| **AHCYL1** | 2.442098 | CC:1.000 |  |
| **RBM3** | 2.439269 | CC:1.000 |  |
| **CLPTM1L** | 2.433583 | LC:1.000 |  |
| **HTATSF1** | 2.431581 | CC:1.000 |  |
| **NOA1** | 2.431223 | CE:1.000 |  |
| **BLM** | 2.426639 | GN:1.000 |  |
| **FDX1** | 2.42283 | LC:1.000 |  |
| **BUD13** | 2.419374 | CC:1.000 |  |
| **GFAP** | 2.417264 | CX:1.000 |  |
| **SUPV3L1** | 2.415796 | CE:1.000 |  |
| **MRPS18A** | 2.415796 | CE:1.000 |  |
| **COQ7** | 2.415796 | CE:1.000 |  |
| **CRHR2** | 2.413998 | DB:1.000 |  |
| **IGF1** | 2.411842 | DB:1.000 |  |
| **DDX1** | 2.411144 | CC:1.000 |  |
| **TMSB10** | 2.406296 | CX:1.000 |  |
| **INPP5E** | 2.406103 | DB:1.000 |  |
| **RPL23** | 2.405464 | CX:1.000 |  |
| **SERPINC1** | 2.405102 | CX:1.000 |  |
| **RPS16** | 2.400994 | CX:1.000 |  |
| **DOK4** | 2.39957 | LC:1.000 |  |
| **SUN2** | 2.39957 | LC:1.000 |  |
| **KRT10** | 2.395727 | CX:1.000 |  |
| **CHMP4C** | 2.39351 | LC:1.000 |  |
| **BMP1** | 2.392436 | HT:1.000 |  |
| **BEGAIN** | 2.388439 | CC:1.000 |  |
| **SLC2A2** | 2.387859 | DB:1.000 |  |
| **SLC2A1** | 2.387859 | DB:1.000 |  |
| **NDUFB6** | 2.386981 | CE:1.000 |  |
| **HNRNPDL** | 2.385566 | CC:1.000 |  |
| **RALY** | 2.385566 | CC:1.000 |  |
| **SNX25** | 2.384718 | DP:1.000 |  |
| **SNX14** | 2.384718 | DP:1.000 |  |
| **COA3** | 2.384716 | CE:1.000 |  |
| **KDM4B** | 2.383542 | LC:1.000 |  |
| **GHRL** | 2.382362 | DB:1.000 |  |
| **PTS** | 2.382334 | LC:1.000 |  |
| **STX16** | 2.378798 | DB:1.000 |  |
| **VTI1A** | 2.378798 | DB:1.000 |  |
| **CNKSR1** | 2.372796 | LC:1.000 |  |
| **SEMA4C** | 2.372796 | LC:1.000 |  |
| **HMGCR** | 2.370902 | DB:1.000 |  |
| **NCBP1** | 2.36481 | CC:1.000 |  |
| **TIGD2** | 2.363054 | HT:1.000 |  |
| **BIRC6** | 2.358887 | LC:1.000 |  |
| **CALB1** | 2.358308 | CC:1.000 |  |
| **RECQL5** | 2.355135 | GN:1.000 |  |
| **PCNT** | 2.353347 | DB:1.000 |  |
| **CPT1A** | 2.353347 | DB:1.000 |  |
| **MAP2** | 2.353347 | DB:1.000 |  |
| **AKAP9** | 2.353347 | DB:1.000 |  |
| **NUP85** | 2.353347 | DB:1.000 |  |
| **PLXNB1** | 2.352103 | LC:1.000 |  |
| **PRPF18** | 2.350888 | CC:1.000 |  |
| **ERMN** | 2.346975 | CX:1.000 |  |
| **AGTPBP1** | 2.345428 | LC:1.000 |  |
| **RNF213** | 2.343765 | LC:1.000 |  |
| **GNB4** | 2.343467 | CC:1.000 |  |
| **FAM193A** | 2.343391 | LC:1.000 |  |
| **MTMR3** | 2.343391 | LC:1.000 |  |
| **TRIM41** | 2.343391 | LC:1.000 |  |
| **NFATC4** | 2.343391 | LC:1.000 |  |
| **CEP162** | 2.343391 | LC:1.000 |  |
| **DNAJB4** | 2.338859 | LC:1.000 |  |
| **TOMM22** | 2.338838 | CC:1.000 |  |
| **RECQL** | 2.337503 | GN:1.000 |  |
| **TMEFF2** | 2.336579 | CX:1.000 |  |
| **VAC14** | 2.334401 | DB:1.000 |  |
| **FIG4** | 2.334401 | DB:1.000 |  |
| **PIKFYVE** | 2.334401 | DB:1.000 |  |
| **LRP2BP** | 2.329197 | LC:1.000 |  |
| **LUC7L2** | 2.328137 | LC:1.000 |  |
| **EEF2** | 2.322065 | DB:1.000 |  |
| **EIF6** | 2.322065 | DB:1.000 |  |
| **EEF2K** | 2.322065 | DB:1.000 |  |
| **MRPL4** | 2.321693 | CE:1.000 |  |
| **DDB2** | 2.321007 | LC:1.000 |  |
| **TMSB4X** | 2.317689 | CX:1.000 |  |
| **MARK4** | 2.315979 | LC:1.000 |  |
| **RPS25** | 2.312029 | CX:1.000 |  |
| **SH3BP5** | 2.31154 | LC:1.000 |  |
| **RPS3** | 2.306173 | CX:1.000 |  |
| **CTNND2** | 2.301502 | CC:1.000 |  |
| **PAN3** | 2.300297 | DB:1.000 |  |
| **PAN2** | 2.300297 | DB:1.000 |  |
| **SNX13** | 2.299767 | DP:1.000 |  |
| **ZP4** | 2.299274 | CC:1.000 |  |
| **ATRN** | 2.297193 | CC:1.000 |  |
| **RPS7** | 2.293854 | CX:1.000 |  |
| **ZBTB26** | 2.291663 | HT:1.000 |  |
| **CCNH** | 2.291596 | HT:1.000 |  |
| **PPP6R3** | 2.291112 | DB:1.000 |  |
| **SEC23IP** | 2.291112 | DB:1.000 |  |
| **PPP6R1** | 2.291112 | DB:1.000 |  |
| **STX17** | 2.291112 | DB:1.000 |  |
| **SEC22A** | 2.291112 | DB:1.000 |  |
| **PPP6C** | 2.291112 | DB:1.000 |  |
| **TFG** | 2.291112 | DB:1.000 |  |
| **SEC22C** | 2.291112 | DB:1.000 |  |
| **SEC16B** | 2.291112 | DB:1.000 |  |
| **SEC16A** | 2.291112 | DB:1.000 |  |
| **GADD45GIP1** | 2.289774 | CE:1.000 |  |
| **MCFD2** | 2.284521 | DB:1.000 |  |
| **CNIH1** | 2.284521 | DB:1.000 |  |
| **CNIH2** | 2.284521 | DB:1.000 |  |
| **AREG** | 2.284521 | DB:1.000 |  |
| **CNIH3** | 2.284521 | DB:1.000 |  |
| **TMEM144** | 2.28448 | CX:1.000 |  |
| **NAF1** | 2.280227 | LC:1.000 |  |
| **RAB43** | 2.279886 | DB:1.000 |  |
| **NEK7** | 2.279532 | CC:1.000 |  |
| **DAPK3** | 2.278347 | LC:1.000 |  |
| **GLS2** | 2.278347 | LC:1.000 |  |
| **SNX18** | 2.2778 | LC:1.000 |  |
| **GOLGA5** | 2.274769 | CC:1.000 |  |
| **ATF7IP** | 2.272338 | DB:1.000 |  |
| **PRDM16** | 2.272338 | DB:1.000 |  |
| **SMYD3** | 2.272338 | DB:1.000 |  |
| **SETD6** | 2.272338 | DB:1.000 |  |
| **SRSF10** | 2.267664 | CC:1.000 |  |
| **RCL1** | 2.267363 | CC:1.000 |  |
| **GGA2** | 2.264515 | LC:1.000 |  |
| **PPL** | 2.261804 | LC:1.000 |  |
| **STK38** | 2.261804 | LC:1.000 |  |
| **NFYC** | 2.260864 | CC:1.000 |  |
| **UNC119** | 2.260006 | LC:1.000 |  |
| **PTN** | 2.260006 | LC:1.000 |  |
| **TOP1MT** | 2.259813 | CC:1.000 |  |
| **HOXC13** | 2.258217 | LC:1.000 |  |
| **HLA-A** | 2.255175 | CX:1.000 |  |
| **RAPGEF3** | 2.251659 | DB:1.000 |  |
| **RAPGEF4** | 2.251659 | DB:1.000 |  |
| **PARD6A** | 2.251404 | LC:1.000 |  |
| **MAML1** | 2.251135 | LC:1.000 |  |
| **EIF3F** | 2.250704 | HT:1.000 |  |
| **TGFA** | 2.247935 | DB:1.000 |  |
| **GCG** | 2.24669 | DB:1.000 |  |
| **ANKFY1** | 2.245904 | LC:1.000 |  |
| **AMER1** | 2.24583 | DB:1.000 |  |
| **HECW2** | 2.244878 | LC:1.000 |  |
| **STRADB** | 2.243422 | DB:1.000 |  |
| **STRADA** | 2.243422 | DB:1.000 |  |
| **CAB39L** | 2.243422 | DB:1.000 |  |
| **ACSM1** | 2.242741 | DB:1.000 |  |
| **MLXIPL** | 2.242741 | DB:1.000 |  |
| **ACSBG1** | 2.242741 | DB:1.000 |  |
| **FZD6** | 2.239832 | DB:1.000 |  |
| **PARD6G** | 2.239238 | LC:1.000 |  |
| **SNX3** | 2.238933 | DB:1.000 |  |
| **GUCY1A2** | 2.23818 | LC:1.000 |  |
| **KCNJ4** | 2.23818 | LC:1.000 |  |
| **GSN** | 2.23818 | LC:1.000 |  |
| **EEF1G** | 2.237646 | CC:0.530;CX:0.470 | |
| **CREB5** | 2.236935 | CC:1.000 |  |
| **CAV1** | 2.236082 | DB:1.000 |  |
| **CNOT8** | 2.233649 | DB:1.000 |  |
| **CNOT6** | 2.233649 | DB:1.000 |  |
| **CNOT1** | 2.233649 | DB:1.000 |  |
| **CNOT4** | 2.233649 | DB:1.000 |  |
| **CNOT9** | 2.233649 | DB:1.000 |  |
| **CNOT2** | 2.233649 | DB:1.000 |  |
| **CNOT7** | 2.233649 | DB:1.000 |  |
| **TNKS1BP1** | 2.233649 | DB:1.000 |  |
| **CNOT6L** | 2.233649 | DB:1.000 |  |
| **CNOT3** | 2.233649 | DB:1.000 |  |
| **CNOT10** | 2.233649 | DB:1.000 |  |
| **CNOT11** | 2.233649 | DB:1.000 |  |
| **INS** | 2.233441 | DB:1.000 |  |
| **PRMT7** | 2.233441 | DB:1.000 |  |
| **PRMT6** | 2.233441 | DB:1.000 |  |
| **COPRS** | 2.233441 | DB:1.000 |  |
| **ERH** | 2.232615 | CX:0.518;CE:0.482 | |
| **UBA52** | 2.232548 | CX:1.000 |  |
| **CASP7** | 2.230994 | DB:1.000 |  |
| **SOX7** | 2.23072 | DB:1.000 |  |
| **SOX13** | 2.23072 | DB:1.000 |  |
| **SOX17** | 2.23072 | DB:1.000 |  |
| **SOX9** | 2.23072 | DB:1.000 |  |
| **SOX3** | 2.23072 | DB:1.000 |  |
| **SOX4** | 2.23072 | DB:1.000 |  |
| **SOX6** | 2.23072 | DB:1.000 |  |
| **CTNNBIP1** | 2.23072 | DB:1.000 |  |
| **CBY1** | 2.23072 | DB:1.000 |  |
| **SRY** | 2.23072 | DB:1.000 |  |
| **CHD8** | 2.23072 | DB:1.000 |  |
| **MACF1** | 2.2306 | LC:1.000 |  |
| **CDK9** | 2.2306 | LC:1.000 |  |
| **EPS8** | 2.229794 | LC:1.000 |  |
| **SPEF2** | 2.228857 | LC:1.000 |  |
| **SERTAD3** | 2.228857 | LC:1.000 |  |
| **RPL11** | 2.228842 | CX:1.000 |  |
| **HPX** | 2.227464 | CX:1.000 |  |
| **TSC1** | 2.225686 | DB:1.000 |  |
| **RHEB** | 2.225686 | DB:1.000 |  |
| **DIRAS2** | 2.225685 | DP:1.000 |  |
| **RHOV** | 2.225685 | DP:1.000 |  |
| **IFT27** | 2.225685 | DP:1.000 |  |
| **RASL11A** | 2.225685 | DP:1.000 |  |
| **MRPL44** | 2.225195 | CE:1.000 |  |
| **MRPL53** | 2.225195 | CE:1.000 |  |
| **CYB5A** | 2.223979 | LC:1.000 |  |
| **RNF144B** | 2.223979 | LC:1.000 |  |
| **NRF1** | 2.220688 | DB:1.000 |  |
| **PPARGC1B** | 2.220688 | DB:1.000 |  |
| **COG5** | 2.219919 | DB:1.000 |  |
| **COG2** | 2.219919 | DB:1.000 |  |
| **COG4** | 2.219919 | DB:1.000 |  |
| **COG6** | 2.219919 | DB:1.000 |  |
| **COG7** | 2.219919 | DB:1.000 |  |
| **COG8** | 2.219919 | DB:1.000 |  |
| **COG1** | 2.219919 | DB:1.000 |  |
| **COG3** | 2.219919 | DB:1.000 |  |
| **SLC7A8** | 2.219844 | PG:1.000 |  |
| **RPS17** | 2.217555 | CX:1.000 |  |
| **SOD3** | 2.217053 | DB:1.000 |  |
| **NOX5** | 2.217053 | DB:1.000 |  |
| **CCS** | 2.217053 | DB:1.000 |  |
| **GPX8** | 2.217053 | DB:1.000 |  |
| **GPX7** | 2.217053 | DB:1.000 |  |
| **PRDX3** | 2.217053 | DB:1.000 |  |
| **GPX6** | 2.217053 | DB:1.000 |  |
| **GPX3** | 2.217053 | DB:1.000 |  |
| **NOX4** | 2.217053 | DB:1.000 |  |
| **NUDT2** | 2.217053 | DB:1.000 |  |
| **TXNRD2** | 2.217053 | DB:1.000 |  |
| **GPX5** | 2.217053 | DB:1.000 |  |
| **GTPBP6** | 2.216743 | GN:1.000 |  |
| **WNT5A** | 2.216207 | DB:1.000 |  |
| **RGP1** | 2.21443 | DB:1.000 |  |
| **RIC1** | 2.21443 | DB:1.000 |  |
| **ALAS1** | 2.211138 | DB:1.000 |  |
| **YY1** | 2.209032 | DB:1.000 |  |
| **RING1** | 2.209032 | DB:1.000 |  |
| **GRSF1** | 2.208905 | CE:1.000 |  |
| **GRM5** | 2.208264 | DB:1.000 |  |
| **GRM1** | 2.208264 | DB:1.000 |  |
| **ABCC8** | 2.207985 | DB:1.000 |  |
| **PAQR6** | 2.206193 | CX:1.000 |  |
| **CLIP1** | 2.202703 | HT:1.000 |  |
| **ATP2B4** | 2.201619 | LC:1.000 |  |
| **DYNLL2** | 2.201619 | LC:1.000 |  |
| **CEP63** | 2.196984 | LC:1.000 |  |
| **NACA** | 2.196304 | CX:1.000 |  |
| **PHF12** | 2.194689 | HT:1.000 |  |
| **COX10** | 2.192562 | CE:1.000 |  |
| **SSBP4** | 2.189233 | LC:1.000 |  |
| **CBFA2T2** | 2.189233 | LC:1.000 |  |
| **6-Sep** | 2.188513 | CC:1.000 |  |
| **ORM1** | 2.187849 | CC:1.000 |  |
| **NEK8** | 2.18783 | CC:1.000 |  |
| **HEY2** | 2.187739 | DB:1.000 |  |
| **HES1** | 2.187739 | DB:1.000 |  |
| **HES7** | 2.187739 | DB:1.000 |  |
| **FAM49B** | 2.186255 | CC:1.000 |  |
| **LMTK3** | 2.183862 | PG:1.000 |  |
| **ATP5F1E** | 2.180739 | CX:0.513;CE:0.487 | |
| **PRDM14** | 2.1757 | CC:1.000 |  |
| **RUNX1T1** | 2.174447 | LC:1.000 |  |
| **TGM4** | 2.173301 | LC:1.000 |  |
| **1-Dec** | 2.171678 | LC:1.000 |  |
| **SNAI3** | 2.171678 | LC:1.000 |  |
| **DDX53** | 2.171678 | LC:1.000 |  |
| **PRH2** | 2.170731 | LC:1.000 |  |
| **MAP3K15** | 2.170731 | LC:1.000 |  |
| **NET1** | 2.170731 | LC:1.000 |  |
| **FZD4** | 2.168087 | LC:1.000 |  |
| **ATP2B2** | 2.168087 | LC:1.000 |  |
| **PKN1** | 2.166932 | DB:1.000 |  |
| **RTN1** | 2.165336 | CC:1.000 |  |
| **E2F2** | 2.161703 | LC:1.000 |  |
| **RCOR3** | 2.159013 | HT:0.530;LC:0.470 | |
| **PDE2A** | 2.146331 | CC:1.000 |  |
| **TDP1** | 2.145858 | LC:1.000 |  |
| **BAZ2A** | 2.145858 | LC:1.000 |  |
| **LGI1** | 2.145602 | CC:1.000 |  |
| **CNTN4** | 2.145602 | CC:1.000 |  |
| **NFATC1** | 2.14493 | DB:0.568;LC:0.432 | |
| **OLIG2** | 2.143528 | CX:1.000 |  |
| **TOMM34** | 2.143437 | CC:1.000 |  |
| **SCO1** | 2.143225 | CE:1.000 |  |
| **RBM42** | 2.139126 | LC:1.000 |  |
| **KCNJ2** | 2.137135 | LC:1.000 |  |
| **CNKSR2** | 2.137135 | LC:1.000 |  |
| **SERPINA6** | 2.135954 | CC:1.000 |  |
| **MIER2** | 2.134249 | LC:0.554;HT:0.446 | |
| **PUF60** | 2.134056 | CC:1.000 |  |
| **DNAJC22** | 2.133185 | PG:1.000 |  |
| **CDK15** | 2.133183 | LC:1.000 |  |
| **CAMK1D** | 2.131788 | CC:1.000 |  |
| **ETV3** | 2.12621 | DB:1.000 |  |
| **DDX20** | 2.12621 | DB:1.000 |  |
| **DEDD2** | 2.124752 | LC:1.000 |  |
| **GNA15** | 2.124111 | LC:1.000 |  |
| **PGLYRP2** | 2.12241 | CX:1.000 |  |
| **STIM2** | 2.118004 | LC:0.583;HT:0.417 | |
| **NKIRAS1** | 2.116503 | DP:1.000 |  |
| **NKIRAS2** | 2.116503 | DP:1.000 |  |
| **LRP1B** | 2.114147 | CC:1.000 |  |
| **DIAPH1** | 2.113989 | LC:1.000 |  |
| **REL** | 2.110878 | LC:1.000 |  |
| **SLC2A4** | 2.108406 | LC:1.000 |  |
| **SURF4** | 2.107808 | LC:1.000 |  |
| **OPRD1** | 2.107791 | LC:1.000 |  |
| **LAMTOR5** | 2.107791 | LC:1.000 |  |
| **OGT** | 2.107791 | LC:1.000 |  |
| **ELOB** | 2.105338 | LC:1.000 |  |
| **GNA12** | 2.10503 | DB:1.000 |  |
| **TRRAP** | 2.103102 | DB:1.000 |  |
| **KAT5** | 2.103102 | DB:1.000 |  |
| **PITX2** | 2.103102 | DB:1.000 |  |
| **CCT5** | 2.099276 | CC:1.000 |  |
| **MEI1** | 2.095399 | CC:1.000 |  |
| **DNAJC21** | 2.094665 | PG:1.000 |  |
| **NDUFAF4** | 2.093471 | CE:1.000 |  |
| **FETUB** | 2.092909 | CC:1.000 |  |
| **POR** | 2.090933 | LC:1.000 |  |
| **ATP1A1** | 2.090796 | CC:1.000 |  |
| **SLC4A2** | 2.08947 | LC:1.000 |  |
| **PRNP** | 2.086245 | LC:1.000 |  |
| **HIST1H2BB** | 2.085663 | LC:1.000 |  |
| **TRIM24** | 2.084826 | HT:1.000 |  |
| **SERPIND1** | 2.082478 | LC:1.000 |  |
| **VBP1** | 2.081684 | HT:1.000 |  |
| **LYL1** | 2.079505 | LC:1.000 |  |
| **SSBP2** | 2.079505 | LC:1.000 |  |
| **HSPA5** | 2.077333 | DB:1.000 |  |
| **MRPL54** | 2.076803 | CE:1.000 |  |
| **E2F6** | 2.076464 | LC:1.000 |  |
| **NEK5** | 2.076337 | CC:1.000 |  |
| **BRD9** | 2.074531 | HT:1.000 |  |
| **EDEM3** | 2.074479 | CC:1.000 |  |
| **RPIA** | 2.074099 | CC:1.000 |  |
| **COPS8** | 2.070291 | HT:1.000 |  |
| **KIF20A** | 2.06709 | CC:1.000 |  |
| **SEC61A1** | 2.066383 | DB:1.000 |  |
| **SEC61A2** | 2.066383 | DB:1.000 |  |
| **SEC61B** | 2.066383 | DB:1.000 |  |
| **SEC61G** | 2.066383 | DB:1.000 |  |
| **DLL1** | 2.062299 | DB:1.000 |  |
| **RBPJ** | 2.062299 | DB:1.000 |  |
| **NDUFB2** | 2.060096 | CE:1.000 |  |
| **PNPT1** | 2.060096 | CE:1.000 |  |
| **NOL6** | 2.058644 | CE:1.000 |  |
| **RPL14** | 2.057743 | CX:1.000 |  |
| **ELAVL2** | 2.055438 | LC:1.000 |  |
| **RPL21** | 2.053908 | CX:1.000 |  |
| **PLCB1** | 2.052911 | CC:1.000 |  |
| **C17orf67** | 2.052033 | HT:1.000 |  |
| **TARS** | 2.051685 | LC:1.000 |  |
| **LBP** | 2.051489 | DB:1.000 |  |
| **ADAM17** | 2.051489 | DB:1.000 |  |
| **GJA1** | 2.051489 | DB:1.000 |  |
| **PPP1CB** | 2.048495 | LC:1.000 |  |
| **SPTAN1** | 2.041767 | DB:1.000 |  |
| **RBM17** | 2.038598 | CC:1.000 |  |
| **PLLP** | 2.036984 | CC:1.000 |  |
| **GNB5** | 2.036984 | CC:1.000 |  |
| **TRAF3IP1** | 2.035991 | LC:1.000 |  |
| **TSC2** | 2.033539 | DB:1.000 |  |
| **KCNJ12** | 2.032926 | LC:1.000 |  |
| **PGK1** | 2.032926 | LC:1.000 |  |
| **RABL3** | 2.032297 | DP:1.000 |  |
| **SPEN** | 2.029862 | DB:1.000 |  |
| **HIST2H3C** | 2.029862 | DB:1.000 |  |
| **NR0B1** | 2.029862 | DB:1.000 |  |
| **PHB2** | 2.029862 | DB:1.000 |  |
| **RPS20** | 2.028577 | CX:1.000 |  |
| **MRPS34** | 2.026576 | CE:1.000 |  |
| **PDK2** | 2.022101 | DB:1.000 |  |
| **PLD2** | 2.02189 | LC:1.000 |  |
| **CNP** | 2.021297 | CC:1.000 |  |
| **FDPS** | 2.019096 | DB:1.000 |  |
| **RPS3A** | 2.018993 | CX:1.000 |  |
| **ELOC** | 2.017952 | LC:1.000 |  |
| **NLK** | 2.017806 | DB:1.000 |  |
| **TLE1** | 2.017806 | DB:1.000 |  |
| **PELP1** | 2.017174 | DB:1.000 |  |
| **CSF1R** | 2.017174 | DB:1.000 |  |
| **ADGRL1** | 2.014111 | CC:1.000 |  |
| **MRPL19** | 2.009766 | CE:1.000 |  |
| **TELO2** | 2.008877 | LC:1.000 |  |
| **BPNT1** | 2.008603 | DB:1.000 |  |
| **ETHE1** | 2.008603 | DB:1.000 |  |
| **SUOX** | 2.008603 | DB:1.000 |  |
| **SQOR** | 2.008603 | DB:1.000 |  |
| **EIF4H** | 2.003531 | LC:1.000 |  |
| **C16orf87** | 2.003531 | LC:1.000 |  |
| **ZNF461** | 2.003531 | LC:1.000 |  |
| **OVOL1** | 2.003531 | LC:1.000 |  |
| **MIER3** | 2.00353 | LC:1.000 |  |
| **DHX35** | 2.002348 | CC:1.000 |  |
| **UBC** | 1.999162 | CX:1.000 |  |
| **GRIK1** | 1.997237 | CC:1.000 |  |
| **TAL1** | 1.996811 | LC:1.000 |  |
| **GPR37** | 1.996499 | CX:1.000 |  |
| **GRIA3** | 1.993651 | CC:1.000 |  |
| **VDAC2** | 1.99145 | LC:1.000 |  |
| **EEF1D** | 1.990844 | CX:1.000 |  |
| **BTRC** | 1.989883 | DB:1.000 |  |
| **TAX1BP3** | 1.989625 | LC:1.000 |  |
| **HDAC6** | 1.988884 | DB:1.000 |  |
| **HDAC4** | 1.988884 | DB:1.000 |  |
| **CAPN2** | 1.988884 | DB:1.000 |  |
| **CACNA1G** | 1.9875 | CC:1.000 |  |
| **SNRNP25** | 1.987455 | CE:1.000 |  |
| **MYB** | 1.985098 | LC:1.000 |  |
| **KAT8** | 1.984968 | LC:1.000 |  |
| **UGT8** | 1.983418 | CC:1.000 |  |
| **DTNA** | 1.982002 | CC:1.000 |  |
| **TAF6** | 1.978177 | LC:1.000 |  |
| **RBL1** | 1.975554 | DB:1.000 |  |
| **TP53RK** | 1.973569 | LC:1.000 |  |
| **CFHR2** | 1.972823 | CC:1.000 |  |
| **SYAP1** | 1.971351 | CC:1.000 |  |
| **GTF2A1** | 1.970031 | DB:1.000 |  |
| **MYBL1** | 1.968124 | LC:1.000 |  |
| **GHR** | 1.965152 | DB:1.000 |  |
| **TAF9** | 1.964808 | LC:1.000 |  |
| **EIF2S1** | 1.96385 | DB:1.000 |  |
| **CHRD** | 1.962656 | DB:1.000 |  |
| **NOG** | 1.962656 | DB:1.000 |  |
| **BMP7** | 1.962656 | DB:1.000 |  |
| **BMPR1A** | 1.962656 | DB:1.000 |  |
| **RFC1** | 1.962656 | DB:1.000 |  |
| **SMAD6** | 1.962656 | DB:1.000 |  |
| **BMP5** | 1.962656 | DB:1.000 |  |
| **BMP4** | 1.962656 | DB:1.000 |  |
| **BMP10** | 1.962656 | DB:1.000 |  |
| **NPPB** | 1.962656 | DB:1.000 |  |
| **BMP2** | 1.962656 | DB:1.000 |  |
| **BMPR2** | 1.962656 | DB:1.000 |  |
| **RPL29** | 1.961899 | CX:1.000 |  |
| **DDX18** | 1.961775 | CC:1.000 |  |
| **ESS2** | 1.959415 | LC:1.000 |  |
| **GRIP1** | 1.958693 | DB:1.000 |  |
| **TBP** | 1.95677 | DB:1.000 |  |
| **PFAS** | 1.954961 | LC:1.000 |  |
| **IRS1** | 1.954753 | DB:1.000 |  |
| **GNAO1** | 1.951495 | LC:1.000 |  |
| **PTEN** | 1.949185 | DB:1.000 |  |
| **CSF1** | 1.947045 | DB:1.000 |  |
| **E2F3** | 1.946983 | LC:1.000 |  |
| **ACTA1** | 1.946091 | DB:1.000 |  |
| **PRKCE** | 1.945411 | DB:1.000 |  |
| **TAF1** | 1.945263 | LC:1.000 |  |
| **MRPL49** | 1.942247 | CE:1.000 |  |
| **CNTNAP4** | 1.942136 | CX:1.000 |  |
| **CCNB1** | 1.941686 | DB:1.000 |  |
| **AUP1** | 1.940915 | LC:1.000 |  |
| **GNAS** | 1.939672 | DB:1.000 |  |
| **ETS1** | 1.939672 | DB:1.000 |  |
| **ETS2** | 1.939672 | DB:1.000 |  |
| **APPL1** | 1.936867 | HT:1.000 |  |
| **RBM7** | 1.936128 | LC:1.000 |  |
| **WDR26** | 1.935118 | LC:1.000 |  |
| **NR0B2** | 1.932577 | LC:1.000 |  |
| **IER5** | 1.93207 | CC:1.000 |  |
| **PPARD** | 1.931919 | DB:1.000 |  |
| **HDAC5** | 1.930771 | DB:1.000 |  |
| **DTNBP1** | 1.930618 | LC:1.000 |  |
| **PPP1R9A** | 1.930399 | LC:1.000 |  |
| **EIF2AK2** | 1.930182 | DB:1.000 |  |
| **CAPNS2** | 1.930051 | DB:1.000 |  |
| **CAPNS1** | 1.930051 | DB:1.000 |  |
| **MKNK1** | 1.929439 | DB:1.000 |  |
| **SRA1** | 1.928683 | DB:1.000 |  |
| **NRIP1** | 1.928683 | DB:1.000 |  |
| **CARM1** | 1.928034 | DB:1.000 |  |
| **BOD1L1** | 1.927455 | HT:1.000 |  |
| **BRCA1** | 1.927406 | DB:1.000 |  |
| **ACTG1** | 1.926547 | CX:1.000 |  |
| **TGFBR3** | 1.926409 | DB:1.000 |  |
| **UBAC2** | 1.925572 | LC:1.000 |  |
| **C4BPA** | 1.924072 | CC:1.000 |  |
| **MAPK15** | 1.922882 | LC:1.000 |  |
| **MYD88** | 1.921675 | DB:1.000 |  |
| **VAPA** | 1.920591 | LC:1.000 |  |
| **ADCY1** | 1.919794 | DB:1.000 |  |
| **PSEN1** | 1.919317 | DB:1.000 |  |
| **RHOA** | 1.918476 | DB:1.000 |  |
| **TAB1** | 1.918476 | DB:1.000 |  |
| **CCSER2** | 1.918229 | HT:1.000 |  |
| **NCOR2** | 1.917863 | DB:1.000 |  |
| **LUC7L** | 1.91784 | CC:1.000 |  |
| **NKX2-5** | 1.916241 | DB:1.000 |  |
| **NPTX1** | 1.915397 | CC:1.000 |  |
| **KCNA4** | 1.915299 | LC:1.000 |  |
| **POLR2C** | 1.915299 | LC:1.000 |  |
| **TGFBR2** | 1.914654 | DB:1.000 |  |
| **EIF4E2** | 1.914174 | LC:1.000 |  |
| **NPPA** | 1.914079 | DB:1.000 |  |
| **E2F1** | 1.912611 | DB:1.000 |  |
| **HNF1A** | 1.912525 | DB:1.000 |  |
| **CSNK2A1** | 1.912289 | DB:1.000 |  |
| **CWC27** | 1.911322 | CC:1.000 |  |
| **RPS6KB1** | 1.910565 | DB:1.000 |  |
| **MYL2** | 1.9104 | DB:1.000 |  |
| **DVL1** | 1.910214 | DB:1.000 |  |
| **NFATC2** | 1.909648 | DB:1.000 |  |
| **PRKAR1B** | 1.908635 | DB:1.000 |  |
| **PRKAR1A** | 1.908558 | DB:1.000 |  |
| **PPP3CC** | 1.907613 | DB:1.000 |  |
| **PPP3CB** | 1.907613 | DB:1.000 |  |
| **PRKACG** | 1.907569 | DB:1.000 |  |
| **PRKACB** | 1.907569 | DB:1.000 |  |
| **PRKAR2A** | 1.907569 | DB:1.000 |  |
| **BRAF** | 1.907128 | LC:1.000 |  |
| **PPP3CA** | 1.906788 | DB:1.000 |  |
| **ATF2** | 1.905822 | DB:1.000 |  |
| **RGS5** | 1.905122 | CC:1.000 |  |
| **RPL13** | 1.905021 | CX:1.000 |  |
| **CALM1** | 1.904214 | DB:1.000 |  |
| **GSK3B** | 1.902663 | DB:1.000 |  |
| **MAPK1** | 1.902445 | DB:1.000 |  |
| **CTR9** | 1.902419 | LC:1.000 |  |
| **MAP3K7** | 1.901908 | DB:1.000 |  |
| **TRPM3** | 1.90174 | CC:1.000 |  |
| **TGFB1** | 1.90149 | DB:1.000 |  |
| **TGFB2** | 1.90149 | DB:1.000 |  |
| **TGFB3** | 1.90149 | DB:1.000 |  |
| **FOS** | 1.901123 | DB:1.000 |  |
| **MYC** | 1.900829 | DB:1.000 |  |
| **HOXD4** | 1.900209 | LC:1.000 |  |
| **PAFAH1B1** | 1.89959 | HT:1.000 |  |
| **NFKB1** | 1.899249 | DB:1.000 |  |
| **JUN** | 1.899225 | DB:1.000 |  |
| **RELA** | 1.898982 | DB:1.000 |  |
| **RASL10A** | 1.898754 | DP:1.000 |  |
| **MAP3K6** | 1.896633 | LC:1.000 |  |
| **STIM1** | 1.896633 | LC:1.000 |  |
| **HIST1H2AB** | 1.889886 | LC:1.000 |  |
| **WDR61** | 1.888143 | LC:1.000 |  |
| **ZFP1** | 1.886391 | LC:1.000 |  |
| **CLPTM1** | 1.883394 | LC:1.000 |  |
| **WBP4** | 1.882927 | LC:1.000 |  |
| **ACACB** | 1.881577 | LC:1.000 |  |
| **PHC2** | 1.879765 | LC:1.000 |  |
| **PRMT2** | 1.879352 | CX:1.000 |  |
| **MAFF** | 1.879229 | HT:1.000 |  |
| **BACH1** | 1.879229 | HT:1.000 |  |
| **GLDC** | 1.878319 | LC:1.000 |  |
| **RSL1D1** | 1.877813 | CE:1.000 |  |
| **FKBP4** | 1.876772 | LC:1.000 |  |
| **SUDS3** | 1.872384 | HT:0.524;LC:0.476 | |
| **CFHR5** | 1.872313 | CC:1.000 |  |
| **FAM83D** | 1.871389 | HT:1.000 |  |
| **HAUS2** | 1.867391 | LC:1.000 |  |
| **ASXL1** | 1.866869 | LC:1.000 |  |
| **LIN52** | 1.86616 | LC:1.000 |  |
| **TFDP2** | 1.86616 | LC:1.000 |  |
| **LIN54** | 1.86616 | LC:1.000 |  |
| **GPRASP1** | 1.865411 | LC:1.000 |  |
| **RPS21** | 1.864114 | CX:1.000 |  |
| **PPIA** | 1.864073 | CX:1.000 |  |
| **HESX1** | 1.860517 | LC:1.000 |  |
| **NANOS2** | 1.859442 | CC:1.000 |  |
| **SLC41A1** | 1.858208 | CC:1.000 |  |
| **NDUFAF7** | 1.857377 | CE:1.000 |  |
| **NIT1** | 1.857121 | GN:1.000 |  |
| **RALB** | 1.856646 | CC:1.000 |  |
| **AHCY** | 1.854446 | HT:1.000 |  |
| **CCDC9** | 1.854446 | HT:1.000 |  |
| **C1orf61** | 1.853487 | CX:1.000 |  |
| **MAGED2** | 1.851516 | LC:1.000 |  |
| **CSNK1G1** | 1.850324 | PG:1.000 |  |
| **RPL22** | 1.847657 | CX:1.000 |  |
| **PIH1D1** | 1.846284 | LC:1.000 |  |
| **ALYREF** | 1.845389 | HT:1.000 |  |
| **SUB1** | 1.845389 | HT:1.000 |  |
| **PARD3** | 1.843047 | LC:1.000 |  |
| **HIPK3** | 1.842362 | CC:1.000 |  |
| **RSF1** | 1.840305 | LC:1.000 |  |
| **RBM23** | 1.837199 | LC:1.000 |  |
| **SAFB** | 1.836894 | LC:1.000 |  |
| **TUBA1C** | 1.835631 | CX:1.000 |  |
| **CLN3** | 1.834461 | LC:1.000 |  |
| **GLMN** | 1.83276 | HT:1.000 |  |
| **PTGS1** | 1.832347 | CX:1.000 |  |
| **NCAPG** | 1.830386 | CC:1.000 |  |
| **AP3D1** | 1.828385 | CC:1.000 |  |
| **DZIP3** | 1.827109 | HT:1.000 |  |
| **KIF11** | 1.826346 | LC:1.000 |  |
| **RPS5** | 1.824525 | CX:1.000 |  |
| **MRPL24** | 1.823337 | CE:1.000 |  |
| **MME** | 1.821173 | CX:1.000 |  |
| **SNRPB2** | 1.820539 | CC:1.000 |  |
| **DDX21** | 1.820539 | CC:1.000 |  |
| **EIF2A** | 1.820186 | LC:1.000 |  |
| **PDE10A** | 1.819398 | CC:1.000 |  |
| **KRT6C** | 1.814482 | LC:1.000 |  |
| **S1PR5** | 1.814482 | LC:1.000 |  |
| **ZNF676** | 1.814482 | LC:1.000 |  |
| **ZBTB4** | 1.814482 | LC:1.000 |  |
| **SH3RF2** | 1.813249 | LC:1.000 |  |
| **ZYX** | 1.809828 | LC:1.000 |  |
| **TK2** | 1.807006 | CC:1.000 |  |
| **BCL11A** | 1.805773 | HT:0.523;LC:0.477 | |
| **SURF2** | 1.803441 | HT:1.000 |  |
| **SIAH2** | 1.802522 | LC:1.000 |  |
| **PTPN13** | 1.79852 | CC:1.000 |  |
| **YEATS4** | 1.798365 | LC:1.000 |  |
| **ANTXR1** | 1.796998 | LC:1.000 |  |
| **KPNB1** | 1.796603 | CC:1.000 |  |
| **RPL18** | 1.792779 | CX:1.000 |  |
| **AMER2** | 1.792367 | CX:1.000 |  |
| **ATP5PD** | 1.789271 | CE:1.000 |  |
| **ABRAXAS2** | 1.788106 | LC:1.000 |  |
| **OGG1** | 1.788019 | PG:1.000 |  |
| **KIF1B** | 1.787525 | LC:1.000 |  |
| **MAP1A** | 1.787525 | LC:1.000 |  |
| **FAF2** | 1.783538 | LC:1.000 |  |
| **VDAC1** | 1.782483 | LC:1.000 |  |
| **SKIL** | 1.78234 | LC:1.000 |  |
| **ACTC1** | 1.78143 | LC:1.000 |  |
| **PSEN2** | 1.77881 | LC:1.000 |  |
| **PIK3R4** | 1.778333 | CC:1.000 |  |
| **SH3BP4** | 1.777335 | HT:1.000 |  |
| **ABCA2** | 1.776302 | HT:1.000 |  |
| **SGCZ** | 1.775442 | CC:1.000 |  |
| **GOLGA7** | 1.774958 | CC:1.000 |  |
| **OTUB1** | 1.774658 | LC:1.000 |  |
| **SCHIP1** | 1.774142 | LC:1.000 |  |
| **ATN1** | 1.77183 | LC:1.000 |  |
| **PLEKHG1** | 1.770963 | LC:1.000 |  |
| **HSBP1** | 1.766712 | LC:1.000 |  |
| **NOS1** | 1.764227 | LC:1.000 |  |
| **TUBA1B** | 1.764101 | CX:1.000 |  |
| **MASTL** | 1.760712 | PG:1.000 |  |
| **HSD17B8** | 1.759682 | GN:1.000 |  |
| **PA2G4** | 1.757515 | LC:1.000 |  |
| **MOV10** | 1.757323 | CC:1.000 |  |
| **GPHN** | 1.754333 | CC:1.000 |  |
| **GRIN2D** | 1.753108 | LC:1.000 |  |
| **RPS13** | 1.748377 | CX:1.000 |  |
| **VIM** | 1.747572 | CX:1.000 |  |
| **SH3KBP1** | 1.747526 | CX:1.000 |  |
| **RNF7** | 1.742554 | LC:1.000 |  |
| **MCM2** | 1.740197 | LC:1.000 |  |
| **CBR4** | 1.733752 | GN:1.000 |  |
| **PHOX2A** | 1.733245 | CC:1.000 |  |
| **APOF** | 1.730775 | CX:1.000 |  |
| **CSNK2B** | 1.729547 | LC:1.000 |  |
| **PRPF19** | 1.72927 | CC:1.000 |  |
| **TJP3** | 1.72817 | CC:1.000 |  |
| **ANKRD44** | 1.727413 | LC:1.000 |  |
| **RCOR2** | 1.725011 | LC:1.000 |  |
| **NUP50** | 1.724107 | HT:1.000 |  |
| **RPA2** | 1.72322 | HT:1.000 |  |
| **CSTB** | 1.722317 | LC:1.000 |  |
| **MPHOSPH9** | 1.722317 | LC:1.000 |  |
| **SLC5A11** | 1.722258 | CX:1.000 |  |
| **TCF4** | 1.722035 | LC:1.000 |  |
| **RARA** | 1.721647 | LC:1.000 |  |
| **CSTF3** | 1.721489 | CC:1.000 |  |
| **HMG20A** | 1.721388 | LC:0.555;HT:0.445 | |
| **ELMSAN1** | 1.721131 | LC:0.521;HT:0.479 | |
| **MRPL35** | 1.721126 | CE:1.000 |  |
| **MRPL36** | 1.721126 | CE:1.000 |  |
| **MRPL51** | 1.721126 | CE:1.000 |  |
| **POLR3D** | 1.718314 | LC:1.000 |  |
| **NCOA2** | 1.718093 | LC:1.000 |  |
| **UTP15** | 1.715944 | CE:1.000 |  |
| **ANXA2** | 1.713598 | CX:1.000 |  |
| **PSRC1** | 1.713457 | CX:1.000 |  |
| **EIF4A3** | 1.713305 | HT:1.000 |  |
| **SLCO1B1** | 1.712818 | CX:1.000 |  |
| **STOM** | 1.71098 | CC:1.000 |  |
| **FGA** | 1.708652 | LC:1.000 |  |
| **SPTB** | 1.707151 | HT:1.000 |  |
| **PDZK1** | 1.702252 | CC:1.000 |  |
| **WDR81** | 1.702066 | LC:1.000 |  |
| **CACNA1H** | 1.702066 | LC:1.000 |  |
| **GDI2** | 1.702045 | CX:1.000 |  |
| **DNAJC30** | 1.701473 | PG:1.000 |  |
| **CSNK1G2** | 1.700237 | PG:1.000 |  |
| **CHD1L** | 1.699771 | LC:1.000 |  |
| **CSNK1D** | 1.699272 | PG:1.000 |  |
| **4-Sep** | 1.696326 | CX:1.000 |  |
| **NPC1** | 1.692855 | LC:1.000 |  |
| **ARPP21** | 1.689715 | CC:1.000 |  |
| **SERPINA7** | 1.688862 | CX:1.000 |  |
| **ZC3HAV1** | 1.687782 | LC:1.000 |  |
| **COA5** | 1.687083 | CE:1.000 |  |
| **ERAL1** | 1.687083 | CE:1.000 |  |
| **PPP6R2** | 1.6867 | LC:1.000 |  |
| **SERPINA11** | 1.686527 | CX:1.000 |  |
| **PCNX1** | 1.686489 | CC:1.000 |  |
| **APOA5** | 1.685754 | CX:1.000 |  |
| **ANKS1B** | 1.683867 | CX:1.000 |  |
| **SIAH1** | 1.680536 | LC:1.000 |  |
| **GRWD1** | 1.679025 | LC:1.000 |  |
| **BCLAF1** | 1.678219 | CC:1.000 |  |
| **TBKBP1** | 1.672094 | LC:1.000 |  |
| **GNAI2** | 1.670322 | LC:1.000 |  |
| **AURKAIP1** | 1.670076 | CE:1.000 |  |
| **RASAL2** | 1.669197 | CC:1.000 |  |
| **APP** | 1.668581 | LC:1.000 |  |
| **EMSY** | 1.667908 | LC:0.514;HT:0.486 | |
| **ZNF512B** | 1.667621 | LC:1.000 |  |
| **TBX3** | 1.66494 | LC:1.000 |  |
| **SAP30L** | 1.66494 | LC:1.000 |  |
| **PRDM4** | 1.664939 | LC:1.000 |  |
| **PARP12** | 1.661649 | LC:1.000 |  |
| **TRIB3** | 1.660768 | LC:1.000 |  |
| **PRSS3** | 1.659351 | LC:1.000 |  |
| **DPYSL3** | 1.657833 | CC:1.000 |  |
| **AP4S1** | 1.656914 | HT:1.000 |  |
| **BAX** | 1.655527 | LC:1.000 |  |
| **ENTR1** | 1.65314 | LC:1.000 |  |
| **DNAJB2** | 1.648437 | PG:1.000 |  |
| **LIMD1** | 1.647415 | HT:1.000 |  |
| **VAT1L** | 1.644887 | CC:1.000 |  |
| **NEDD8** | 1.642035 | LC:1.000 |  |
| **CCT7** | 1.6419 | CC:1.000 |  |
| **ACTR2** | 1.6419 | CC:1.000 |  |
| **MAG** | 1.637791 | CX:1.000 |  |
| **EAPP** | 1.637255 | HT:1.000 |  |
| **IFIT2** | 1.636886 | LC:1.000 |  |
| **ECPAS** | 1.636709 | LC:1.000 |  |
| **NDUFAF3** | 1.636101 | CE:1.000 |  |
| **ILF2** | 1.63586 | CC:1.000 |  |
| **USP50** | 1.635545 | HT:1.000 |  |
| **RPLP2** | 1.633055 | CX:1.000 |  |
| **CPLX1** | 1.632732 | CC:1.000 |  |
| **DSP** | 1.631363 | CX:1.000 |  |
| **ATP13A5** | 1.631139 | CX:1.000 |  |
| **TTBK2** | 1.630481 | PG:1.000 |  |
| **CSNK1G3** | 1.626235 | PG:1.000 |  |
| **SLAIN1** | 1.626011 | CX:1.000 |  |
| **TRIM27** | 1.624583 | LC:1.000 |  |
| **COPS6** | 1.624243 | LC:1.000 |  |
| **PNMA2** | 1.623469 | LC:1.000 |  |
| **PRR3** | 1.623284 | CC:1.000 |  |
| **ITGA2B** | 1.622133 | CX:1.000 |  |
| **TARBP2** | 1.620352 | LC:1.000 |  |
| **LOR** | 1.620126 | CX:1.000 |  |
| **DMAC1** | 1.619137 | CE:1.000 |  |
| **HSP90AA1** | 1.617427 | CX:1.000 |  |
| **USP19** | 1.616669 | LC:1.000 |  |
| **HBS1L** | 1.616299 | LC:1.000 |  |
| **INHBC** | 1.615426 | CX:1.000 |  |
| **LGALS1** | 1.614984 | CX:1.000 |  |
| **HOXA11** | 1.613519 | LC:1.000 |  |
| **PIMREG** | 1.613519 | LC:1.000 |  |
| **ESCO1** | 1.613519 | LC:1.000 |  |
| **ABCA1** | 1.610234 | LC:1.000 |  |
| **OPHN1** | 1.608908 | CX:1.000 |  |
| **GSR** | 1.608373 | GN:1.000 |  |
| **UBE2O** | 1.606979 | LC:1.000 |  |
| **RBFOX2** | 1.606979 | LC:1.000 |  |
| **RPS27L** | 1.606368 | CC:1.000 |  |
| **CAPN1** | 1.606008 | LC:1.000 |  |
| **DHRS4** | 1.604908 | CC:1.000 |  |
| **OXSM** | 1.602192 | CE:1.000 |  |
| **KRI1** | 1.599541 | LC:1.000 |  |
| **QTRT2** | 1.599194 | LC:1.000 |  |
| **LRP2** | 1.595819 | LC:1.000 |  |
| **KAT2A** | 1.590987 | LC:1.000 |  |
| **TRAF2** | 1.589288 | LC:1.000 |  |
| **BICRA** | 1.589276 | HT:1.000 |  |
| **CTSL** | 1.588543 | LC:1.000 |  |
| **RANBP9** | 1.585612 | LC:1.000 |  |
| **ZNF488** | 1.585545 | CX:1.000 |  |
| **MRPS7** | 1.585268 | CE:1.000 |  |
| **RPL10** | 1.584041 | CX:1.000 |  |
| **AEBP2** | 1.583905 | LC:1.000 |  |
| **SRSF4** | 1.583664 | CC:1.000 |  |
| **CTTN** | 1.582845 | CX:1.000 |  |
| **PHLDB3** | 1.582555 | LC:1.000 |  |
| **MAL** | 1.579247 | CC:1.000 |  |
| **QKI** | 1.578372 | LC:1.000 |  |
| **MANSC1** | 1.575363 | HT:1.000 |  |
| **LLGL1** | 1.575179 | LC:1.000 |  |
| **TTBK1** | 1.574049 | PG:1.000 |  |
| **MMS19** | 1.57084 | LC:1.000 |  |
| **CD3D** | 1.569196 | CX:1.000 |  |
| **ARID5B** | 1.568656 | LC:1.000 |  |
| **SALL1** | 1.568656 | LC:1.000 |  |
| **ZNF318** | 1.568655 | LC:1.000 |  |
| **NACC2** | 1.568655 | LC:1.000 |  |
| **SNX4** | 1.568424 | CC:1.000 |  |
| **URI1** | 1.566795 | LC:1.000 |  |
| **PPP1CC** | 1.564471 | LC:1.000 |  |
| **SF3B6** | 1.561756 | CC:1.000 |  |
| **PPARG** | 1.561472 | LC:1.000 |  |
| **TCERG1** | 1.560499 | CC:1.000 |  |
| **MAP3K5** | 1.559606 | LC:1.000 |  |
| **SGK3** | 1.556692 | PG:0.572;GN:0.428 | |
| **C8A** | 1.555943 | CX:1.000 |  |
| **STT3B** | 1.555506 | LC:1.000 |  |
| **HIPK4** | 1.554796 | HT:1.000 |  |
| **WDR24** | 1.554796 | HT:1.000 |  |
| **FSTL1** | 1.554796 | HT:1.000 |  |
| **PYROXD2** | 1.554796 | HT:1.000 |  |
| **RRS1** | 1.554796 | HT:1.000 |  |
| **EIF3H** | 1.554107 | HT:1.000 |  |
| **BCL2L1** | 1.553044 | LC:1.000 |  |
| **ZBTB20** | 1.552291 | CX:1.000 |  |
| **MRPL9** | 1.551491 | CE:1.000 |  |
| **SLC38A1** | 1.549669 | CC:1.000 |  |
| **MAP1LC3A** | 1.548874 | LC:1.000 |  |
| **HIST2H2AC** | 1.548631 | LC:1.000 |  |
| **UBE3A** | 1.547537 | LC:1.000 |  |
| **TFDP1** | 1.546327 | LC:1.000 |  |
| **F13B** | 1.546129 | CX:1.000 |  |
| **CCNA1** | 1.543059 | LC:1.000 |  |
| **SEC14L1** | 1.542369 | CC:1.000 |  |
| **GNG12** | 1.541997 | CC:1.000 |  |
| **CDK2** | 1.541936 | PG:0.591;GN:0.409 | |
| **SEL1L** | 1.541684 | LC:1.000 |  |
| **ARHGAP45** | 1.541597 | CX:1.000 |  |
| **SP100** | 1.540712 | HT:1.000 |  |
| **NPR1** | 1.540156 | GN:1.000 |  |
| **NPR2** | 1.540156 | GN:1.000 |  |
| **IGF2BP1** | 1.539221 | CC:1.000 |  |
| **FARSA** | 1.538957 | LC:1.000 |  |
| **ERBB4** | 1.537442 | LC:1.000 |  |
| **CSNK1E** | 1.536138 | PG:1.000 |  |
| **PHACTR2** | 1.535648 | CC:1.000 |  |
| **MRPL50** | 1.534641 | CE:1.000 |  |
| **MRPS15** | 1.534641 | CE:1.000 |  |
| **ATP5F1D** | 1.534641 | CE:1.000 |  |
| **NEK3** | 1.533423 | CC:1.000 |  |
| **IFIT3** | 1.530094 | LC:1.000 |  |
| **MIER1** | 1.528927 | LC:1.000 |  |
| **ZNF217** | 1.528927 | LC:1.000 |  |
| **HOPX** | 1.528927 | LC:1.000 |  |
| **PADI4** | 1.528927 | LC:1.000 |  |
| **RBM10** | 1.528023 | LC:1.000 |  |
| **IKBKG** | 1.525323 | LC:1.000 |  |
| **DUSP3** | 1.525232 | CC:1.000 |  |
| **TTR** | 1.525094 | LC:1.000 |  |
| **IGF2BP2** | 1.522163 | CC:1.000 |  |
| **ACP1** | 1.519799 | LC:1.000 |  |
| **FTL** | 1.518758 | CX:1.000 |  |
| **ATP5PF** | 1.51782 | CE:1.000 |  |
| **EIF3B** | 1.514237 | LC:1.000 |  |
| **TRAP1** | 1.514237 | LC:1.000 |  |
| **MECP2** | 1.513746 | LC:1.000 |  |
| **RUNX2** | 1.513618 | LC:1.000 |  |
| **CSK** | 1.51174 | HT:1.000 |  |
| **MARK1** | 1.511143 | LC:1.000 |  |
| **MDH2** | 1.510942 | LC:1.000 |  |
| **COPS7B** | 1.510798 | HT:1.000 |  |
| **HNRNPH2** | 1.506637 | LC:1.000 |  |
| **HECTD1** | 1.504682 | LC:1.000 |  |
| **RNF113A** | 1.504395 | CC:1.000 |  |
| **RPUSD3** | 1.50103 | CE:1.000 |  |
| **CUL2** | 1.499677 | LC:1.000 |  |
| **MSH5** | 1.499439 | CC:1.000 |  |
| **EMD** | 1.498492 | LC:1.000 |  |
| **RPL5** | 1.497661 | CX:1.000 |  |
| **GABRA2** | 1.497154 | CC:1.000 |  |
| **RPAP2** | 1.496142 | LC:1.000 |  |
| **HR** | 1.493324 | LC:1.000 |  |
| **IFRD1** | 1.493324 | LC:1.000 |  |
| **ESCO2** | 1.493323 | LC:1.000 |  |
| **TP73** | 1.492393 | LC:1.000 |  |
| **ARID4B** | 1.492207 | LC:0.637;HT:0.363 | |
| **JADE2** | 1.491615 | HT:1.000 |  |
| **APOA1** | 1.491192 | LC:1.000 |  |
| **ZFYVE26** | 1.491127 | HT:1.000 |  |
| **IVNS1ABP** | 1.486973 | LC:1.000 |  |
| **RPS4Y2** | 1.484272 | LC:1.000 |  |
| **TOP1** | 1.483445 | LC:1.000 |  |
| **HMG20B** | 1.483269 | LC:0.651;HT:0.349 | |
| **SERPINA1** | 1.482478 | CX:1.000 |  |
| **GRB2** | 1.481863 | LC:1.000 |  |
| **CDC5L** | 1.480514 | LC:1.000 |  |
| **TUBA1A** | 1.480411 | CX:1.000 |  |
| **KRT5** | 1.478307 | CX:1.000 |  |
| **NASP** | 1.47726 | CC:1.000 |  |
| **FCHO2** | 1.47507 | PG:1.000 |  |
| **HMGB1** | 1.474303 | CX:1.000 |  |
| **PHB** | 1.474066 | LC:1.000 |  |
| **SMAD9** | 1.472418 | LC:1.000 |  |
| **EIF3D** | 1.471216 | HT:1.000 |  |
| **SYN3** | 1.471162 | CC:1.000 |  |
| **SYNRG** | 1.471037 | CX:1.000 |  |
| **SCAF4** | 1.470575 | LC:1.000 |  |
| **HDAC9** | 1.470561 | LC:1.000 |  |
| **HMMR** | 1.468105 | HT:1.000 |  |
| **DOCK7** | 1.467223 | LC:1.000 |  |
| **ANKHD1** | 1.464813 | LC:1.000 |  |
| **RNF2** | 1.464074 | LC:1.000 |  |
| **RPH3AL** | 1.462967 | LC:1.000 |  |
| **CARD6** | 1.462967 | LC:1.000 |  |
| **LCORL** | 1.462967 | LC:1.000 |  |
| **HOXA3** | 1.462967 | LC:1.000 |  |
| **TLX3** | 1.462967 | LC:1.000 |  |
| **GAS2L2** | 1.462967 | LC:1.000 |  |
| **NPAS3** | 1.462967 | LC:1.000 |  |
| **PAK6** | 1.462967 | LC:1.000 |  |
| **PATZ1** | 1.462967 | LC:1.000 |  |
| **AMHR2** | 1.462967 | LC:1.000 |  |
| **ARHGAP26** | 1.462966 | LC:1.000 |  |
| **P3H4** | 1.462966 | LC:1.000 |  |
| **SLC5A12** | 1.462966 | LC:1.000 |  |
| **ZNF787** | 1.462966 | LC:1.000 |  |
| **PARP14** | 1.461105 | LC:1.000 |  |
| **RREB1** | 1.461105 | LC:1.000 |  |
| **SSX2B** | 1.461105 | LC:1.000 |  |
| **CIR1** | 1.461105 | LC:1.000 |  |
| **EGFR** | 1.461104 | LC:1.000 |  |
| **GNAI1** | 1.460797 | HT:1.000 |  |
| **RPL4** | 1.456225 | CX:1.000 |  |
| **SHKBP1** | 1.455545 | LC:1.000 |  |
| **PRKD1** | 1.454453 | LC:1.000 |  |
| **SDHC** | 1.450863 | CE:1.000 |  |
| **HLA-C** | 1.445635 | CX:1.000 |  |
| **TCF12** | 1.443646 | LC:1.000 |  |
| **POLR2E** | 1.442727 | CE:1.000 |  |
| **PIK3CG** | 1.441952 | LC:1.000 |  |
| **KDM5A** | 1.43952 | LC:0.527;HT:0.473 | |
| **DNAJC11** | 1.438078 | PG:1.000 |  |
| **HIST2H2BE** | 1.437386 | LC:1.000 |  |
| **BCL2** | 1.437386 | LC:1.000 |  |
| **FN1** | 1.436104 | LC:1.000 |  |
| **FASLG** | 1.435123 | LC:1.000 |  |
| **MBTPS1** | 1.43171 | LC:1.000 |  |
| **ZMYM3** | 1.43171 | LC:1.000 |  |
| **PHF21A** | 1.43171 | LC:1.000 |  |
| **ZNF827** | 1.43171 | LC:1.000 |  |
| **ARHGAP6** | 1.430306 | CX:1.000 |  |
| **UBE2I** | 1.429293 | LC:1.000 |  |
| **TERF2IP** | 1.429197 | LC:1.000 |  |
| **PPP1CA** | 1.429197 | LC:1.000 |  |
| **USP10** | 1.429187 | LC:1.000 |  |
| **MAPK13** | 1.425714 | LC:1.000 |  |
| **SMARCAD1** | 1.421959 | LC:1.000 |  |
| **CDC20** | 1.421959 | LC:1.000 |  |
| **PMS2** | 1.419084 | PG:1.000 |  |
| **FLNA** | 1.407399 | LC:1.000 |  |
| **SLCO1B3** | 1.406565 | CX:1.000 |  |
| **DNTTIP1** | 1.404705 | LC:1.000 |  |
| **RUNX1** | 1.402833 | LC:1.000 |  |
| **MRPS28** | 1.401041 | CE:1.000 |  |
| **FAU** | 1.397067 | CX:1.000 |  |
| **CENPA** | 1.394494 | LC:1.000 |  |
| **CEP250** | 1.394148 | LC:1.000 |  |
| **ZGPAT** | 1.39411 | LC:1.000 |  |
| **WDR3** | 1.392619 | HT:1.000 |  |
| **CBX8** | 1.387259 | LC:1.000 |  |
| **SF1** | 1.386024 | LC:1.000 |  |
| **PLEC** | 1.385489 | PG:1.000 |  |
| **AKAP6** | 1.383398 | DP:1.000 |  |
| **JARID2** | 1.37975 | LC:1.000 |  |
| **IKZF2** | 1.379749 | LC:1.000 |  |
| **PRR18** | 1.379297 | CX:1.000 |  |
| **FLCN** | 1.374744 | LC:1.000 |  |
| **ACVR2A** | 1.374491 | PG:1.000 |  |
| **MEPCE** | 1.371891 | LC:1.000 |  |
| **TPM1** | 1.371326 | CX:1.000 |  |
| **DNAJC25** | 1.36899 | PG:1.000 |  |
| **PFDN2** | 1.368051 | HT:1.000 |  |
| **PTP4A2** | 1.365763 | HT:1.000 |  |
| **PRKACA** | 1.364297 | LC:1.000 |  |
| **HMGA1** | 1.364139 | LC:1.000 |  |
| **PINK1** | 1.363426 | LC:1.000 |  |
| **HTT** | 1.361308 | LC:1.000 |  |
| **NRGN** | 1.357125 | CX:1.000 |  |
| **RANBP1** | 1.356736 | CX:1.000 |  |
| **NR2E1** | 1.356568 | LC:1.000 |  |
| **HELLS** | 1.356568 | LC:1.000 |  |
| **IKZF4** | 1.356568 | LC:1.000 |  |
| **GSE1** | 1.356567 | LC:1.000 |  |
| **ZFPM2** | 1.356567 | LC:1.000 |  |
| **REPIN1** | 1.356567 | LC:1.000 |  |
| **OXCT2** | 1.355819 | GN:1.000 |  |
| **EIF3E** | 1.355543 | HT:1.000 |  |
| **PLXNB2** | 1.354823 | HT:1.000 |  |
| **CSNK1A1L** | 1.353676 | PG:1.000 |  |
| **BARD1** | 1.352504 | LC:1.000 |  |
| **U2AF2** | 1.349622 | LC:1.000 |  |
| **DHX36** | 1.348227 | CX:1.000 |  |
| **TBL3** | 1.346649 | CE:1.000 |  |
| **PYCR3** | 1.343835 | LC:1.000 |  |
| **NPM1** | 1.341126 | CX:1.000 |  |
| **SNCA** | 1.339733 | LC:1.000 |  |
| **LIN28A** | 1.337326 | LC:1.000 |  |
| **SRC** | 1.335875 | LC:1.000 |  |
| **GLRX5** | 1.335232 | CE:1.000 |  |
| **SNAI2** | 1.334936 | LC:1.000 |  |
| **RBP1** | 1.334936 | LC:1.000 |  |
| **RIOX1** | 1.334876 | HT:1.000 |  |
| **RBMXL1** | 1.334384 | CE:1.000 |  |
| **RERE** | 1.333433 | LC:0.607;HT:0.393 | |
| **VCL** | 1.332779 | LC:1.000 |  |
| **SH3GL2** | 1.332578 | PG:1.000 |  |
| **CFL1** | 1.332326 | CX:1.000 |  |
| **NSFL1C** | 1.332214 | LC:1.000 |  |
| **TMPRSS15** | 1.331008 | CX:1.000 |  |
| **AGO2** | 1.328791 | LC:1.000 |  |
| **TBK1** | 1.328758 | LC:1.000 |  |
| **GBP7** | 1.327616 | CX:1.000 |  |
| **TIAM1** | 1.325105 | LC:1.000 |  |
| **KIF21A** | 1.320968 | CX:1.000 |  |
| **PTBP1** | 1.320617 | LC:1.000 |  |
| **PCDHGC5** | 1.320075 | CX:1.000 |  |
| **MAP4K4** | 1.318755 | CX:1.000 |  |
| **RACK1** | 1.314713 | CX:1.000 |  |
| **BCL11B** | 1.314671 | LC:1.000 |  |
| **HMGN2** | 1.313145 | CX:1.000 |  |
| **MAP6D1** | 1.312949 | CX:1.000 |  |
| **COIL** | 1.307531 | LC:1.000 |  |
| **CBX1** | 1.306331 | LC:1.000 |  |
| **PPIC** | 1.302688 | PG:1.000 |  |
| **COX14** | 1.302625 | CE:1.000 |  |
| **NDUFV1** | 1.302625 | CE:1.000 |  |
| **CHD5** | 1.301843 | HT:1.000 |  |
| **CCDC8** | 1.301409 | LC:1.000 |  |
| **HLA-B** | 1.301147 | CX:1.000 |  |
| **XRCC6** | 1.30089 | LC:1.000 |  |
| **SH3GL1** | 1.297887 | PG:1.000 |  |
| **A2M** | 1.296059 | CX:1.000 |  |
| **TSC22D3** | 1.295617 | LC:1.000 |  |
| **BLOC1S1** | 1.295617 | LC:1.000 |  |
| **LMNA** | 1.293837 | LC:1.000 |  |
| **RB1** | 1.29352 | LC:1.000 |  |
| **SLCO1A2** | 1.292975 | CX:1.000 |  |
| **TRAF3** | 1.29213 | LC:1.000 |  |
| **SERPING1** | 1.291937 | HT:1.000 |  |
| **METAP1** | 1.291454 | GN:1.000 |  |
| **METAP1D** | 1.291454 | GN:1.000 |  |
| **NDUFA3** | 1.286402 | CE:1.000 |  |
| **DHX30** | 1.286402 | CE:1.000 |  |
| **CNDP2** | 1.28515 | GN:1.000 |  |
| **HLA-DRA** | 1.284226 | CX:1.000 |  |
| **HBB** | 1.281266 | CX:1.000 |  |
| **SMURF1** | 1.280248 | LC:1.000 |  |
| **KDM5C** | 1.277647 | LC:1.000 |  |
| **GFI1** | 1.277647 | LC:1.000 |  |
| **PRDX1** | 1.276843 | CX:1.000 |  |
| **SLC22A9** | 1.276757 | CX:1.000 |  |
| **DNAJC24** | 1.274772 | PG:1.000 |  |
| **DDX17** | 1.2734 | LC:1.000 |  |
| **AKR1C4** | 1.271697 | CX:1.000 |  |
| **GALNT7** | 1.27084 | CX:1.000 |  |
| **COX19** | 1.270234 | CE:1.000 |  |
| **ATP5F1A** | 1.270234 | CE:1.000 |  |
| **CNDP1** | 1.268869 | GN:1.000 |  |
| **PMS1** | 1.266524 | PG:1.000 |  |
| **MLH1** | 1.266524 | PG:1.000 |  |
| **TADA2A** | 1.266466 | LC:1.000 |  |
| **KHDRBS1** | 1.264092 | LC:1.000 |  |
| **NOLC1** | 1.263842 | CX:1.000 |  |
| **TXNDC5** | 1.26065 | LC:1.000 |  |
| **HDAC10** | 1.26065 | LC:1.000 |  |
| **CDYL** | 1.26065 | LC:1.000 |  |
| **RECQL4** | 1.260244 | GN:1.000 |  |
| **TNS1** | 1.255979 | CX:1.000 |  |
| **ANLN** | 1.25482 | CX:1.000 |  |
| **PAK1** | 1.254717 | LC:1.000 |  |
| **EIF5B** | 1.253939 | CX:1.000 |  |
| **NDUFB1** | 1.251319 | CX:1.000 |  |
| **GRM3** | 1.248397 | CX:1.000 |  |
| **ADAM21** | 1.246259 | CX:1.000 |  |
| **TERF2** | 1.245072 | LC:1.000 |  |
| **PRDM1** | 1.24453 | LC:1.000 |  |
| **IKZF3** | 1.24453 | LC:1.000 |  |
| **GLI1** | 1.24453 | LC:1.000 |  |
| **NCR2** | 1.243837 | CX:1.000 |  |
| **LAGE3** | 1.241995 | HT:1.000 |  |
| **ABCE1** | 1.241469 | CX:1.000 |  |
| **PDLIM5** | 1.240891 | PG:1.000 |  |
| **ABCB6** | 1.239645 | GN:1.000 |  |
| **ITGA1** | 1.235494 | HT:1.000 |  |
| **SEMA4D** | 1.231387 | CX:1.000 |  |
| **HIF1AN** | 1.229207 | LC:1.000 |  |
| **SPARC** | 1.228998 | CX:1.000 |  |
| **PTPRO** | 1.228961 | CX:1.000 |  |
| **PSME3** | 1.223807 | CX:1.000 |  |
| **TRAF3IP3** | 1.221748 | CX:1.000 |  |
| **CPN1** | 1.221047 | CX:1.000 |  |
| **CD48** | 1.219486 | CX:1.000 |  |
| **DCLK2** | 1.217347 | CX:1.000 |  |
| **FOXP3** | 1.21461 | LC:1.000 |  |
| **ZEB2** | 1.21461 | LC:1.000 |  |
| **RPS9** | 1.21418 | CX:1.000 |  |
| **CCDC136** | 1.211739 | HT:1.000 |  |
| **LIG4** | 1.20896 | LC:1.000 |  |
| **ZNF420** | 1.208639 | HT:1.000 |  |
| **ITGA4** | 1.208044 | LC:1.000 |  |
| **EIF3J** | 1.206837 | HT:1.000 |  |
| **PRRC2B** | 1.206837 | HT:1.000 |  |
| **SLC25A47** | 1.20652 | CX:1.000 |  |
| **HIST3H3** | 1.206078 | LC:1.000 |  |
| **ARHGAP19** | 1.204165 | HT:1.000 |  |
| **SAA4** | 1.201369 | CX:1.000 |  |
| **PABPC3** | 1.201263 | CX:1.000 |  |
| **MAD1L1** | 1.200676 | LC:1.000 |  |
| **MAF** | 1.200676 | LC:1.000 |  |
| **PLAC8** | 1.199771 | CX:1.000 |  |
| **CAMK4** | 1.197677 | CX:1.000 |  |
| **PRMT1** | 1.195277 | LC:1.000 |  |
| **MTURN** | 1.191901 | CX:1.000 |  |
| **MTF2** | 1.191203 | HT:1.000 |  |
| **NME6** | 1.190281 | CE:1.000 |  |
| **NARS** | 1.187352 | LC:1.000 |  |
| **SENP1** | 1.187352 | LC:1.000 |  |
| **LAMP1** | 1.183813 | CX:1.000 |  |
| **FLG** | 1.182508 | CX:1.000 |  |
| **ATXN7** | 1.179361 | CX:1.000 |  |
| **TSPAN8** | 1.177647 | CX:1.000 |  |
| **C9** | 1.175345 | CX:1.000 |  |
| **LUC7L3** | 1.17521 | CX:1.000 |  |
| **ZBTB7A** | 1.174589 | LC:1.000 |  |
| **KLF5** | 1.174589 | LC:1.000 |  |
| **MRPL18** | 1.174478 | CE:1.000 |  |
| **CAMK2N1** | 1.172272 | CX:1.000 |  |
| **MRFAP1** | 1.171722 | CX:1.000 |  |
| **NOP53** | 1.170902 | CX:1.000 |  |
| **DALRD3** | 1.170421 | CX:1.000 |  |
| **TERF1** | 1.17031 | LC:1.000 |  |
| **NOP16** | 1.167889 | CX:1.000 |  |
| **S100A8** | 1.167537 | CX:1.000 |  |
| **HLA-E** | 1.165384 | CX:1.000 |  |
| **CUL1** | 1.16538 | LC:1.000 |  |
| **MDM2** | 1.165171 | LC:1.000 |  |
| **SUMO2** | 1.162965 | LC:1.000 |  |
| **CIITA** | 1.162345 | LC:1.000 |  |
| **SAP25** | 1.162344 | LC:1.000 |  |
| **MYLK** | 1.160616 | CX:1.000 |  |
| **NUTF2** | 1.160198 | CX:1.000 |  |
| **ING3** | 1.158631 | CX:1.000 |  |
| **TIMM23** | 1.156771 | CX:1.000 |  |
| **KRTAP4-2** | 1.155588 | CX:1.000 |  |
| **NCOA4** | 1.155508 | CX:1.000 |  |
| **KRTDAP** | 1.154687 | CX:1.000 |  |
| **RGS2** | 1.154501 | CX:1.000 |  |
| **TRAF6** | 1.154182 | LC:1.000 |  |
| **DHX9** | 1.154102 | LC:1.000 |  |
| **CYP2E1** | 1.153274 | CX:1.000 |  |
| **FCGR3B** | 1.152891 | CX:1.000 |  |
| **GNG11** | 1.151501 | CX:1.000 |  |
| **RUNX3** | 1.15058 | LC:1.000 |  |
| **NR2C1** | 1.15058 | LC:1.000 |  |
| **SATB1** | 1.15058 | LC:1.000 |  |
| **CD24** | 1.149289 | CX:1.000 |  |
| **SERP1** | 1.148303 | CX:1.000 |  |
| **PGD** | 1.146642 | GN:1.000 |  |
| **PPP1R8** | 1.139262 | LC:1.000 |  |
| **DCLRE1C** | 1.13735 | HT:1.000 |  |
| **HNRNPK** | 1.129877 | LC:1.000 |  |
| **RLIM** | 1.128359 | LC:1.000 |  |
| **WIZ** | 1.128359 | LC:1.000 |  |
| **CBX7** | 1.128359 | LC:1.000 |  |
| **HIGD2A** | 1.12747 | CE:1.000 |  |
| **UCHL5** | 1.122005 | LC:1.000 |  |
| **ARHGAP44** | 1.1208 | PG:1.000 |  |
| **VCP** | 1.120779 | LC:1.000 |  |
| **RPA3** | 1.120564 | LC:1.000 |  |
| **LMO4** | 1.117844 | LC:1.000 |  |
| **SAP130** | 1.117844 | LC:1.000 |  |
| **TMEM206** | 1.113084 | HT:1.000 |  |
| **LOXL2** | 1.104893 | HT:1.000 |  |
| **DNAJC4** | 1.101457 | PG:1.000 |  |
| **CHD3** | 1.100117 | HT:1.000 |  |
| **DOCK8** | 1.098236 | HT:1.000 |  |
| **ISCA2** | 1.096477 | CE:1.000 |  |
| **FBXO21** | 1.094559 | HT:1.000 |  |
| **GNAI3** | 1.090145 | HT:1.000 |  |
| **DHX37** | 1.090145 | HT:1.000 |  |
| **TRIM11** | 1.086262 | HT:1.000 |  |
| **C1QTNF2** | 1.083587 | HT:1.000 |  |
| **XRCC4** | 1.076292 | HT:1.000 |  |
| **JMJD8** | 1.071135 | HT:1.000 |  |
| **CAMK2D** | 1.063295 | HT:1.000 |  |
| **IDH3A** | 1.046513 | GN:1.000 |  |
| **MMS22L** | 1.038031 | HT:1.000 |  |
| **IRAK1** | 1.032914 | PG:1.000 |  |
| **COX15** | 1.02028 | CE:1.000 |  |
| **ETNPPL** | 1.019333 | GN:1.000 |  |
| **SIRT6** | 1.012779 | HT:1.000 |  |
| **PYCR2** | 1.009607 | GN:1.000 |  |
| **MRPL45** | 1.005269 | CE:1.000 |  |
| **PYCR1** | 0.991679 | GN:1.000 |  |
| **SERPINB13** | 0.99131 | HT:1.000 |  |
| **COX18** | 0.990337 | CE:1.000 |  |
| **AP1G2** | 0.990247 | HT:1.000 |  |
| **PCSK5** | 0.985902 | HT:1.000 |  |
| **FAM92A** | 0.985902 | HT:1.000 |  |
| **SLC22A6** | 0.985902 | HT:1.000 |  |
| **CHMP5** | 0.985902 | HT:1.000 |  |
| **ZCCHC12** | 0.985902 | HT:1.000 |  |
| **KIF6** | 0.985902 | HT:1.000 |  |
| **MUL1** | 0.985902 | HT:1.000 |  |
| **GART** | 0.982924 | GN:1.000 |  |
| **DNAJC14** | 0.978444 | PG:1.000 |  |
| **SDHB** | 0.975485 | CE:1.000 |  |
| **ZNF219** | 0.963485 | HT:1.000 |  |
| **EIF3L** | 0.958779 | HT:1.000 |  |
| **EIF3I** | 0.958779 | HT:1.000 |  |
| **RTF1** | 0.957917 | CE:1.000 |  |
| **SNRPE** | 0.954689 | HT:1.000 |  |
| **TXNDC15** | 0.945844 | HT:1.000 |  |
| **CBS** | 0.943387 | HT:1.000 |  |
| **RAD51C** | 0.943271 | HT:1.000 |  |
| **LRG1** | 0.939427 | HT:1.000 |  |
| **UBAC1** | 0.935147 | HT:1.000 |  |
| **AES** | 0.931828 | HT:1.000 |  |
| **MRPL58** | 0.931416 | CE:1.000 |  |
| **DNAJA4** | 0.930956 | PG:1.000 |  |
| **SNRPF** | 0.930419 | CE:0.504;HT:0.496 | |
| **KBTBD7** | 0.921014 | HT:1.000 |  |
