# Supplementary material for: An integrative analysis of genome-wide association study and regulatory SNP annotation datasets identified candidate genes for bipolar disorder
Source: Int J Bipolar Disord. 2020 Feb 3;8:6. doi: 10.1186/s40345-019-0170-z (PMC6995798; doi:10.1186/s40345-019-0170-z)
Supplement: Supplementary file 7 — Additional file 7: Table S6. List of the guide genes identified by HumanNet-XC analysis [file 40345_2019_170_MOESM7_ESM.docx]

Table S6. List of the guide genes identified by HumanNet-XC analysis

| **Gene** | **Score** | **Evidences** | **Connected Queried Genes** |
| --- | --- | --- | --- |
| RBM4B | 5.843771 | DP:0.552;CC:0.235;HT:0.213 | RBM4;RBM14-RBM4;RBM14 |
| RBM4 | 5.787791 | DP:0.555;LC:0.231;HT:0.214 | EIF4A1;RBM14-RBM4;RBM4B |
| ITIH3 | 4.374088 | DP:0.568;CC:0.432 | ITIH4 |
| ITIH4 | 4.374088 | DP:0.568;CC:0.432 | ITIH3 |
| RBM14-RBM4 | 4.064204 | DP:1.000 | RBM4;RBM4B |
| KMT2E | 3.656399 | CC:0.549;DB:0.451 | KMT2D |
| KMT2D | 3.656399 | CC:0.549;DB:0.451 | KMT2E |
| RBM14 | 3.312884 | CC:1.000 | HNRNPAB;RBM4B |
| WNT1 | 2.674488 | DB:1.000 | WNT10B |
| WNT10B | 2.674488 | DB:1.000 | WNT1 |
| SPCS1 | 2.356615 | CC:1.000 | GLT8D1 |
| GLT8D1 | 2.356615 | CC:1.000 | SPCS1 |
| CACNA2D2 | 2.32388 | DB:1.000 | CACNA2D3 |
| CACNA2D3 | 2.32388 | DB:1.000 | CACNA2D2 |
| HNRNPAB | 1.776838 | CC:1.000 | RBM14 |
| EIF4A1 | 1.480065 | LC:1.000 | RBM4 |
| CTDSPL | 0 | - | - |
| MOBP | 0 | - | - |
| GOLGA4 | 0 | - | - |
| TRANK1 | 0 | - | - |
| LMAN2L | 0 | - | - |
| NEK4 | 0 | - | - |
| DLG2 | 0 | - | - |
| JRKL | 0 |  |  |
| TENM4 | 0 |  |  |
| ANKRD36 | 0 |  |  |
| APPBP2 | 0 |  |  |
| CNNM4 | 0 |  |  |
| PBRM1 | 0 |  |  |
| CCDC88A | 0 |  |  |
| EID1 | 0 |  |  |
| TM9SF3 | 0 |  |  |
| PATL2 | 0 |  |  |
| WDR76 | 0 |  |  |
| CYCS | 0 |  |  |
| RPL41 | 0 |  |  |
| CADPS | 0 |  |  |
| FHIT | 0 |  |  |
| RHEBL1 | 0 |  |  |
| RCE1 | 0 |  |  |
| PRKAG1 | 0 |  |  |
| CCSER1 | 0 |  |  |
| CCDC170 | 0 |  |  |
| IPCEF1 | 0 |  |  |
| OPRM1 | 0 |  |  |
| SYNE1 | 0 |  |  |
| GIPC2 | 0 |  |  |
| ARF3 | 0 |  |  |
| DDX23 | 0 |  |  |
| RGS17 | 0 |  |  |
| TFB1M | 0 |  |  |
| HDAC2 | 0 |  |  |
| RHOD | 0 |  |  |
