# Supplementary material for: An integrative analysis of genome-wide association study and regulatory SNP annotation datasets identified candidate genes for bipolar disorder
Source: Int J Bipolar Disord. 2020 Feb 3;8:6. doi: 10.1186/s40345-019-0170-z (PMC6995798; doi:10.1186/s40345-019-0170-z)
Supplement: Supplementary file 8 — Additional file 8: Table S7. HumanNet-XC gene-set analysis results. [file 40345_2019_170_MOESM8_ESM.docx]

Table S7. HumanNet-XC gene-set analysis results

| **Rank** | **Term ID** | **Term Description** | **P value** |
| --- | --- | --- | --- |
| ***DisGeNET*** | | | |
| 1 | C0005586 | Bipolar Disorder | 7.41×10^-5^ |
| 2 | C0349204 | Nonorganic psychosis | 9.18×10^-5^ |
| 3 | C0033975 | Psychotic Disorders | 8.41×10^-4^ |
| 4 | C0023470 | Myeloid Leukemia | 1.03×10^-3^ |
| 5 | C0236733 | Amphetamine-Related Disorders | 1.10×10^-3^ |
| 6 | C0036341 | Schizophrenia | 1.61×10^-3^ |
| 7 | C0542514 | Blue sclera | 3.44×10^-3^ |
| 8 | C0236969 | Substance-Related Disorders | 3.88×10^-3^ |
| 9 | C0025500 | Mesothelioma | 5.40×10^-3^ |
| ***DISEAASE*** |  |  |  |
| 1 | DOID:3324 | Mood disorder | 7.75×10^-5^ |
| 2 | DOID:3312 | Bipolar disorder | 5.88×10^-3^ |
| 3 | DOID:5419 | Schizophrenia | 7.38×10^-3^ |
| 4 | DOID:302 | Substance abuse | 9.10×10^-3^ |

Note: with a 5.90 in thresholding the prediction score.
